# Supplementary material for: The methodologies to assess the effectiveness of non-pharmaceutical interventions during COVID-19: a systematic review
Source: Eur J Epidemiol. 2022 Sep 24;37(10):1003–24. doi: 10.1007/s10654-022-00908-y (PMC9510554; doi:10.1007/s10654-022-00908-y)
Supplement: Supplementary file 1 — (pdf 1672 KB) [file 10654_2022_908_MOESM1_ESM.pdf]

The methodologies to assess the effectiveness of non-pharmaceutical interventions during COVID-19: a systematic review

Nicolas Banholzer[1*‡], Adrian Lison[2*‡], Dennis Özcelik[3], Tanja Stadler[2], Stefan Feuerriegel[1,4], and Werner Vach[5,6]

[1]ETH Zurich, Department of Management, Technology, and Economics, Zurich, Switzerland

[2]ETH Zurich, Department of Biosystems Science and Engineering, Zurich, Switzerland

[3]ETH Zurich, Chemistry | Biology | Pharmacy Information Center, Zurich, Switzerland

[4]LMU Munich, LMU Munich School of Management, Munich, Germany

[5]Basel Academy for Quality and Research in Medicine, Basel, Switzerland

[6]University of Basel, Department of Environmental Sciences, Basel, Switzerland

[*]Corresponding author: nbanholzer@ethz.ch

[‡]These authors contributed equally to this work.

Appendices

A Remarks on results **2**

B Literature search **4**

C Additional results **9**

D Data extraction manual **12**

E Data extraction report **35**

F Excluded studies **650**

G PRISMA checklist **692**

References **696**

A Remarks on results

Analyses inferring transmission dynamics

Almost all of the analyses in our review sample that used an unobserved outcome representing transmission dynamics (e. g. the reproduction number or transmission rate) inferred it from one or several observed outcomes (e. g. cases or deaths). For this, non-parametric methods or transmission models were typically used; however, these methods were not always correctly applied.

First, many analyses partially or completely neglected the stochastic time lags in the process from infection to reporting, including disease progression (time from infection to symptom onset, hospitalization, or death) and delays in ascertainment (testing, forwarding to health authority). That is, to estimate the reproduction number or transmission rate, non-parametric methods were often directly applied to the time series of confirmed cases, hospitalizations, or deaths, without accounting for the delays between infection and reporting (e. g.[1–3]). Similarly, analyses using transmission models sometimes equated confirmed cases with infections (e. g.[4–6]). This can lead to overconfident estimates of the reproduction number or transmission rate that may be both shifted in time (e. g. by the mean incubation period and reporting delay) and blurred (e. g. if reports of cases infected on a given day are spread over successive days)[7].

Second, authors must be aware that there exist different versions of the reproduction number R, which have been developed for particular use cases and require different interpretations. Aside from the method by Cori et al.[8], some authors also used other approaches for estimation of the instantaneous reproduction number[9,10], basic reproduction number[11,12], or case reproduction number[13]. However, some analyses did not use methods suitable for the research question at hand or the data analyzed. For example, the methods by White et al.[11] or Wallinga and Lipsitch[12] were designed for estimating the basic reproduction number during the early period of exponential growth in an epidemic, but some analyses applied them also to the time after interventions were implemented[14–16], which was typically a period of subexponential growth. Similarly, the case reproduction number as estimated with the method by Wallinga and Teunis[13] is a forward-looking reproduction number that does not describe transmission at one specific point in time, and is thus not ideal for assessing the population-level effectiveness of an intervention on a specific date.

Overall, differences in the methods used for inferring transmission dynamics could, to some

extent, also explain different findings regarding the effectiveness of non-pharmaceutical interventions.

Careful coding of interventions

Systematic coding of intervention data is necessary to compare the effectiveness of a comparable set of non-pharmaceutical interventions across multiple populations. However, the process of such systematic coding involves subjective decisions regarding if and when non-pharmaceutical interventions have been implemented[17]. These decisions are sometimes unavoidable, and they are also inherent to the development of public databases[18]. As a result, different coding of interventions could impact the results, and, therefore, coding decisions should be made transparent and documented carefully. In addition, we recommend to examine empirically the impact when using a different coding of interventions, e. g. by comparing the results across different input data.

Data availability for comparisons across studies

Important for comparisons across different studies is giving access to the preprocessed data, yet our results revealed that most study authors accessed these data directly from national authorities or external data providers without making the preprocessed data additionally available. To promote reproducibility, study authors should always make the preprocessed data available for two reasons. First, future access to the data via the original source may be restricted (this has already been observed for corporate mobility data). Second, originally analyzed and recently accessed data may be different as publicly available epidemiological data and coded intervention data can be subject to updates, revisions, and corrections. In addition, publication of computer code is crucial to support comparisons of methodological approaches, specific modeling choices, and input data.

B Literature search

Database search queries

We composed our search queries of four components to be contained in the publication title or abstract: (1) a synonym for "non-pharmaceutical intervention", (2) a synonym for "estimation" or "assessment", (3) a synonym for "effect", and (4) a synonym for "COVID-19". In the following, we provide the detailed string of our search query for each database searched, i. e. Embase, PubMed, Scopus, and Web of Science. The differences in query strings between databases are mostly of syntactical nature and result from the specific conventions of each search interface.

Embase

```
SEARCH QUERY:=
        ('non pharmac*':ab,ti OR 'non-pharmac*':ab,ti OR 'nonpharmac*':ab,ti OR
        'anti contagion':ab,ti OR 'anti-contagion':ab,ti OR 'anticontagion':ab,ti
        OR 'distancing':ab,ti OR 'contact restriction*':ab,ti OR 'containment
        strateg*':ab,ti OR 'containment measure*':ab,ti OR 'containment
        polic*':ab,ti OR 'confinement strateg*':ab,ti OR 'confinement
        measure*':ab,ti OR 'confinement polic*':ab,ti OR 'quarantine
        strateg*':ab,ti OR 'quarantine measure*':ab,ti OR 'quarantine polic*':ab,ti
        OR 'control strateg*':ab,ti OR 'control measure*':ab,ti OR 'control
        polic*':ab,ti OR 'mitigation measure*':ab,ti OR 'mitigation strateg*':ab,ti
        OR 'mitigation polic*':ab,ti OR 'government action*':ab,ti OR 'government
        measure*':ab,ti OR 'government response*':ab,ti OR 'government
        intervention*':ab,ti OR 'public health action*':ab,ti OR 'public health
        measure*':ab,ti OR 'public health response*':ab,ti OR 'public health
        polic*':ab,ti OR 'public health intervention*':ab,ti)
   AND
        (estimat*:ab,ti OR evaluat*:ab,ti OR assess*:ab,ti OR quantify*:ab,ti OR
        calculat*:ab,ti OR infer*:ab,ti OR rank*:ab,ti OR explain*:ab,ti)
   AND
```

(effect*:ab,ti OR effic*:ab,ti OR impact*:ab,ti OR associ*:ab,ti OR link*:ab,ti OR relat*:ab,ti OR chang*:ab,ti)

AND

'coronavirus infection'/exp (#ncov* already included)

PubMed

SEARCH QUERY:=

((non pharmac*[Title/Abstract]) OR (nonpharmac*[Title/Abstract]) OR (non-pharmac*[Title/Abstract]) OR (anti contagion[Title/Abstract]) OR (anticontagion[Title/Abstract]) OR (anti-contagion[Title/Abstract]) OR (distancing[Title/Abstract]) OR (contact restriction*[Title/Abstract]) OR (containment strateg*[Title/Abstract]) OR (containment measure*[Title/Abstract]) OR (containment polic*[Title/Abstract]) OR (confinement strateg*[Title/Abstract]) OR (confinement measure*[Title/Abstract]) OR (confinement polic*[Title/Abstract]) OR (quarantine strateg*[Title/Abstract]) OR (quarantine measure*[Title/Abstract]) OR (quarantine polic*[Title/Abstract]) OR (control strateg*[Title/Abstract]) OR (control measure*[Title/Abstract]) OR (control polic*[Title/Abstract]) OR (mitigation measure*[Title/Abstract]) OR (mitigation strateg*[Title/Abstract]) OR (mitigation polic*[Title/Abstract]) OR (government action*[Title/Abstract]) OR (government measure*[Title/Abstract]) OR (government response*[Title/Abstract]) OR (government intervention*[Title/Abstract]) OR (public health action*[Title/Abstract]) OR (public health measure*[Title/Abstract]) OR (public health response*[Title/Abstract]) OR (public health polic*[Title/Abstract]) OR (public health intervention*[Title/Abstract]))

AND

((estimat*[Title/Abstract]) OR (evaluat*[Title/Abstract]) OR (assess*[Title/Abstract]) OR (quantify*[Title/Abstract]) OR

```
    (calculat*[Title/Abstract]) OR (infer*[Title/Abstract]) OR
    (rank*[Title/Abstract]) OR (explain*[Title/Abstract]))
  AND
    ((effect*[Title/Abstract]) OR (effic*[Title/Abstract]) OR
    (impact*[Title/Abstract]) OR (associ*[Title/Abstract]) OR
    (link*[Title/Abstract]) OR (relat*[Title/Abstract]) OR
    (chang*[Title/Abstract]))
  AND
    (("SARS-CoV-2"[Mesh]) OR ("COVID-19"[Mesh]))
```

Scopus

```
SEARCH QUERY:=
  TITLE-ABS(
    ((non W/0 pharmac*) OR (non-pharmac*) OR (nonpharmac*) OR (anti W/0
    contagion) OR (anti-contagion) OR (anticontagion) OR (distancing) OR
    (contact W/0 restriction*) OR (containment W/0 strateg*) OR (containment
    W/0 measure*) OR (containment W/0 polic*) OR (confinement W/0 strateg*)
    OR (confinement W/0 measure*) OR (confinement W/0 polic*) OR (quarantine
    W/0 strateg*) OR (quarantine W/0 measure*) OR (quarantine W/0 polic*) OR
    (control W/0 strateg*) OR (control W/0 measure*) OR (control W/0 polic*)
    OR (mitigation W/0 measure*) OR (mitigation W/0 strateg*) OR (mitigation
    W/0 polic*) OR (government W/0 action*) OR (government W/0 measure*) OR
    (government W/0 response*) OR (government W/0 intervention*) OR (public
    W/0 health W/0 action*) OR (public W/0 health W/0 measure*) OR (public
    W/0 health W/0 response*) OR (public W/0 health W/0 polic*) OR (public w/0
    health w/0 intervention*))
  AND
    ((estimat*) OR (evaluat*) OR (assess*) OR (calculat*) OR (infer*) OR (rank*)
    OR (explain*))
  AND
```

```
    ((effect*) OR (effic*) OR (impact*) OR (associ*) OR (link*) OR (relat*) OR
    (chang*))
  AND
    ((ncov*) OR (covid*) OR (sars*) OR (coronavir*) OR (corona W/0 vir*))
  )
```

Web of Science

We independently searched by TI and AB (title and abstract) for non-pharmaceutical interventions, and then combined both searches using the Boolean operator OR.

```
SEARCH QUERY:=
    AB/TI=('non pharmac*' OR 'non-pharmac*' OR 'nonpharmac*' OR 'anti contagion'
    OR 'anti-contagion' OR 'anticontagion' OR 'distancing' OR 'contact
    restriction*' OR 'containment strateg*' OR 'containment measure*' OR
    'containment polic*' OR 'confinement strateg*' OR 'confinement measure*' OR
    'confinement polic*' OR 'quarantine strateg*' OR 'quarantine measure*' OR
    'quarantine polic*' OR 'control strateg*' OR 'control measure*' OR 'control
    polic*' OR 'mitigation measure*' OR 'mitigation strateg*' OR 'mitigation
    polic*' OR 'government action*' OR 'government measure*' OR 'government
    response*' OR 'government intervention*' OR 'public health action*' OR
    'public health measure*' OR 'public health response*' OR 'public health
    polic*' OR 'public health intervention*')
  AND
    ALL=(estimat* OR evaluat* OR assess* OR quantify* OR calculat* OR infer* OR
    rank* OR explain*)
  AND
    ALL=(effect* OR effic* OR impact* OR associ* OR link* OR relat* OR chang*)
  AND
    ALL=(ncov* OR covid* OR sars* OR corona$vir*)
```

Initial filtering of studies based on keywords

We screened the titles of the studies retrieved from the database search for keywords clearly suggesting that the study would not meet our predefined eligibility criteria. The following set of keywords was compiled:

`air, alcohol, attitudes, clinical, consumption, depression, distress, eating, education, environmental, experience, food, habits, knowledge, medical, mental, nursing, orthopaedic, perceptions, pollution, psychological, psychosocial, quality, remote, services, sleep, students, surgery, telehealth, telemedicine, training, transplant, trauma, virtual, wellbeing, workers`

The above keywords were thoroughly checked in order to minimize the risk of erroneous exclusion of studies. The compiled set of keywords was then used to identify cases for exclusion via the publication title. That is, studies containing one or more exclusion keywords in the publication title (abstract or full text not considered) were excluded.

C Additional results

Detailed breakdown of methods by empirical approach

For better readability of the manuscript, the frequencies of methods at the lower level were not broken down by empirical approach to ensure conciseness. The detailed breakdown of the methods by empirical approach is shown in Table S1.

Table S1. Detailed breakdown of method frequencies by empirical approach.

**Method**	**(D)**	**(P)**	**(C)**	Total freq.
Description of change over time	136	—	—	136 (48 %)
↳ Description of time course	↳ 49	↳ —	↳ —	↳ 49 (36 %)
↳ Comparison of time periods	↳ 87	↳ —	↳ —	↳ 87 (64 %)
Comparison of populations	8	—	—	8 (3 %)
Comparison of change points with intervention dates	7	—	—	7 (2 %)
Non-mechanistic model	—	61	17	78 (27 %)
↳ Generalized linear model	↳ —	↳ 49	↳ 2	↳ 51 (65 %)
↳ Exponential growth model	↳ —	↳ 0	↳ 11	↳ 11 (14 %)
↳ Other	↳ —	↳ 12	↳ 4	↳ 16 (21 %)
Mechanistic model	—	30	13	43 (15 %)
↳ Compartmental single-population transmission model	↳ —	↳ 21	↳ 8	↳ 29 (67 %)
↳ Compartmental meta-population transmission model	↳ —	↳ 1	↳ 3	↳ 4 (9 %)
↳ Semi-mechanistic Bayesian transmission model	↳ —	↳ 5	↳ 0	↳ 5 (12 %)
↳ Other	↳ —	↳ 3	↳ 2	↳ 5 (12 %)
Synthetic controls	—	—	6	6 (2 %)
Other	0	3	4	7 (2 %)

Empirical approach: **(D)** descriptive, **(P)** parametric, and **(C)** counterfactual

Comparison of citation counts by methodological approach and analysis type

We compared the average number of citations per study by methodological approach and across the four main types of analyses that we identified. The number of citations for each study until June 21, 2022 was retrieved using the semantic scholar search engine. For each category, we computed the mean and median number of citations per study (Table S2). To check for potential confounding by date of publication (where earlier studies had more time to accumulate citations), we also report citation statistics separately for the first and second half of studies in our review sample, defined by the date after which half of the studies have been published.

Overall, we find that studies using an analysis of type (2), where a computed, unobserved outcome is linked to interventions, and of type (3), where an observed outcome is linked to interventions via an unobserved outcome in a mechanistic model, had a higher mean number of citations than studies using other analysis types. Moreover, studies using variation in the exposure both over time and between populations had a consistently higher mean number of citations and a higher or similar median number of citations. Our results show that studies with above average number of citations often assessed the effectiveness of interventions on unobserved outcomes (measures of epidemic trend or epidemiological parameters) and exploited both variation over time and between populations. We also find that studies making the code used for their analysis publicly available had a consistently higher mean and median number of citations.

Table S2. Mean and median number of citations per study (rounded) until June 21, 2022 for different types of analyses and methodological approaches. Statistics are shown for (i) all studies, (ii) studies published before August 26, 2020 (1st half), and (iii) studies published after August 26, 2020 (2nd half).

	Citations overall			Citations 1st half			Citations 2nd half		
**Type of analysis**	Mean	Med.	n	Mean	Med.	n	Mean	Med.	n
**(1)** Observed outcome	82	25	119	142	34	54	33	20	65
**(2)** Computed, unobserved outcome	115	25	128	177	31	69	41	13	59
**(3)** Unobserved outcome in mechanistic model	172	22	31	306	41	15	46	18	16
**(4)** Change points in outcome	20	21	7	22	21	4	16	12	3
**Empirical approach**									
**D:** Descriptive	117	25	151	191	31	79	35	14	72
**P:** Parametric	110	23	94	192	39	42	44	20	52
**C:** Counterfactual	49	22	40	70	24	21	26	18	19
**Use of exposure variation**									
Only variation over time for a single population	95	24	125	171	31	62	21	12	63
Only variation over time for multiple populations	100	23	95	148	22	50	46	26	45
Only variation between populations	81	22	18	129	39	10	21	18	8
Both variation over time and between populations	152	29	47	266	64	20	67	22	27
**Method**									
Description of change over time	124	26	136	200	32	72	38	16	64
Comparison of populations	84	19	8	202	41	3	14	12	5
Comparison of change points with intervention dates	20	21	7	22	21	4	16	12	3
Non-mechanistic model	73	23	78	109	36	36	42	19	42
Mechanistic model	139	23	43	225	34	23	41	18	20
Synthetic controls	28	30	6	22	22	1	29	38	5
Other	71	21	7	139	21	3	20	13	4
**Code availability**									
None (not available)	70	23	220	109	28	115	27	14	105
Publicly available	224	38	65	448	160	27	66	30	38

D Data extraction manual

The following data extraction manual describes the items that we extracted for each dimension. The data extraction manual was used throughout the data extraction process.

Summary of the data extraction process

The data extraction manual was developed along with the data collection and analysis process. We started with a pre-defined list of items. For each item, we built a pre-defined set of categories from the first 20 studies that we extracted. Later, if necessary, more categories were added during extraction. Some items were not categorized (see, for example, Items D.3.1 and D.3.2), and some items were merged or removed if they became redundant (i. e. if the entries from one item could easily be determined from the entries of other items). The final list of items is shown below in the table of contents.

Studies with multiple entries

Some studies assessed the effectiveness of multiple types of non-pharmaceutical interventions with different methodological approaches. As such, these studies can have multiple entries, that is, one for each analysis. No additional entry was added if only different outcomes were analyzed but with respect to the same interventions and methodological approaches. Different outcomes were separated by comma.

Reporting

In the data extraction report, there is one row for each item. Multiple values are separated by comma (see for example an entry with values "`cases, deaths, recovered`" for Item D.2.1). Specific information for the entered data are given in parentheses, and further details are written after a colon, e. g. "`behavioral outcome (mobility: population outflow from Wuhan)`".

D.0 Study information 15
D.0.1 Author 15
D.0.2 Title 15
D.0.3 Year 15
D.0.4 Journal 15
D.0.5 DOI 15

D.0.6 Date received 15

D.0.7 Date accepted 15

D.1 Study setting 15

D.1.1 Number of populations included 15

D.1.2 Level of populations included 15

D.1.3 Geographic areas covered 16

D.1.4 Number of countries covered 16

D.1.5 Study period 16

D.2 Outcome 17

D.2.1 Raw outcome 18

D.2.2 Time resolution of raw outcome 19

D.2.3 Computed outcome 19

D.2.4 Method to obtain the computed outcome 20

D.2.5 Data source 21

D.2.6 Data availability 22

D.3 Intervention 23

D.3.1 Terminology for interventions 23

D.3.2 Terminology for the specific type of non-pharmaceutical interventions . . . 23

D.3.3 Exposure types 24

D.3.4 Types of single interventions 25

D.3.5 Coding of interventions 26

D.3.6 Source of intervention data 26

D.3.7 Availability of data on exposure 27

D.4 Methodological approach 27

D.4.1 Empirical approach 28

D.4.2 Use of exposure variation 28

D.4.3 Method 29

D.4.4 Code availability 31

D.5 Effectiveness assessment 31

D.5.1 Reporting of effectiveness 31
D.5.2 Measure of effectiveness 32
D.5.3 Interpretation of results 32
D.5.4 Reporting of uncertainty 32
D.5.5 Sensitivity analysis 33
D.5.6 Subgroup assessment 33

D.0 Study information

D.0.1 Author

The author(s) of the study.

D.0.2 Title

The title of the study.

D.0.3 Year

The year the study was published.

D.0.4 Journal

The journal where the study was published.

D.0.5 DOI

The digital object identifier (DOI).

D.0.6 Date received

The date the study was received.

D.0.7 Date accepted

The date the study was accepted. If not available, then the date when the paper was first published in print or online (depending on what came first), as noted in parentheses.

D.1 Study setting

D.1.1 Number of populations included

Whether a single or multiple populations (countries, states, cities) are analyzed.

- **Single**: A single population, e. g. a single country or subnational region is analyzed.
- **Multiple**: Multiple populations are analyzed, e. g. multiple countries or multiple subnational regions.

D.1.2 Level of populations included

Whether populations are studied at the national or a subnational level. Can be both, e. g. if a country as a whole and its subnational regions are studied. The specific population is given in parentheses.

- **National**: Populations are studied at the national level, i. e. countries.

- **Subnational**: Populations are studied at the subnational level, e. g. state or city.

D.1.3 Geographic areas covered

The geographic areas of the populations that are studied.

- **Asia**

- **Europe**

- **North America**

- **Middle East & Africa**

- **Central & South America**

- **Oceania**

D.1.4 Number of countries covered

Whether the analysis covers populations from multiple countries or from a single country (e. g. China, United States, India, Italy, etc).

- **Multiple countries**: Populations from multiple countries, i. e. multiple countries or subnational regions from multiple countries.

- **Single country**: Populations from a single country, i. e. a single country or subnational regions from a single country. If applicable, then the name of that country.

D.1.5 Study period

Any specifics regarding the start or end dates of the study period in relation to the course of the epidemic in which the intervention(s) were assessed. We checked if analyses covered both a rise and decline in cases to assess the effectiveness of interventions. Any observed deviations from this setup are noted. If the raw outcome is behavioral, then only deviations of the study period from the timing of interventions are noted. As the vast majority of studies analyzed the first wave in the respective population, it is also noted when a study included data from a subsequent wave.

- **Nothing specific (Start and end date span first epidemic wave)**: Study period covers rise and decline in new cases (with respect to confirmed cases as reported by "Our World in Data"[19]) during the first epidemic wave in the analyzed population, and starts before and ends after the analyzed interventions were implemented.
- **Late start**: The study period starts comparatively late for one or several of the populations analyzed, either with respect to the wave (e. g. only after the peak) or with respect to the intervention (e. g. only after the intervention was implemented).
- **End date in growth phase of wave**: The study period has a comparatively early end date compared to the epidemic wave in one or several of the populations analyzed, i. e. cases are still clearly rising.
- **End date at peak of wave**: The study period ends (roughly) at the peak of the first wave in one or several of the populations analyzed, i. e. new cases are stabilizing or start declining.
- **Same end date for several populations with diverse epidemic trajectories**: Several populations are included in the analysis, and, during the study period, the course of the epidemic varies significantly across the populations. Typically, this would mean that the end date of the study period was still within the epidemic growth phase for some populations and already in the control phase for other populations.
- **Start date could not be evaluated**: The start of the study period could not be evaluated.
- **End date could not be evaluated**: The end of the study period could not be evaluated.
- **Includes second wave**: Study includes data from a second wave in one or several of the populations analyzed. This can either mean that the study analyses several waves (separately or jointly) or only focuses on the second wave. Details noted in parentheses.
- **Other**: Anything else noteworthy about the study period, details noted in parentheses.

D.2 Outcome

During the data extraction process, we observed that some analyses directly used the raw data from the outcome to assess the association with an intervention, while other analyses involved an

intermediate step in which an unobserved outcome was computed from the raw outcome. In the latter case, the computed outcome was then analyzed instead of the raw outcome, or sometimes in addition to it. Therefore, we subsequently distinguish between the raw outcome (Item D.2.1) and the computed outcome (Item D.2.3).

D.2.1 Raw outcome

The raw outcome from the data that was available to the study.

- **Epidemiological population-level outcome**: Cases (documented number of cumulative or new cases), deaths, recovered cases, hospitalizations, intensive care unit (ICU) hospitalizations, ICU beds occupation, emergency room visits, severe cases (people severely ill from COVID-19), active cases (number of active cases as obtained from external data), basic or effective reproduction number (as obtained from external data), exported cases (cases exported from one population to another, as obtained from external data), imported cases (cases imported from one population to another, as obtained from external data), tests (number of COVID-19 tests conducted, or number of positive tests), test positivity rate (ratio of positive tests to total tests), date of first reported case in a country, or surrogate disease (a similar epidemiological outcome as for COVID-19, but for a different disease, e. g. cases of influenza, with exact outcome and disease noted in parentheses).

- **Epidemiological individual-level outcome**: (1) Individual cases, i. e. cases with case ID, demographics, clinical characteristics, and epidemiological characteristics (e. g. the date of symptom onset, travel history, potentially close contacts), which can used to estimate the serial interval; (2) individual cases and transmission chains, i. e. cases with case ID and the link between index and secondary case that allow inference of transmission chains, in order to compute the analyzed outcome (e. g. estimating the serial interval and/or the reproduction number); or (3) genome sequence data, i. e. genome sequence data of clinical SARS-CoV-2 samples.

- **Behavioral outcome**: (1) Mobility (mobility data, e. g. time spent in various locations or distance travelled) or (2) survey responses (survey response data, e. g. on social distancing or hygiene behavior).

D.2.2 Time resolution of raw outcome

Time resolution of data for the raw outcome (Item D.2.1), e. g. daily, weekly, monthly, etc.

D.2.3 Computed outcome

Outcomes computed in an intermediate step from a raw outcome and then used in the main effectiveness assessment. Detailed computed outcome in parentheses.

- **None**: No intermediate step, i. e. the raw outcome is used in the main assessment.
- **Measure of epidemic trend**: Doubling time, growth rate, or other. Usually all of these measures are derived from confirmed cases. If otherwise, the respective outcome is noted after a colon in the data extraction report.
- **Epidemiological parameter**: Reproduction number (basic or effective reproduction number, which is usually estimated from the confirmed number of cases; if not, the respective outcome is noted after a colon)*, transmission rate (also known as effective contact rate), contact rate, probability of transmission upon contact, hospitalization rate, recovery rate, death rate, case fatality rate, serial interval, time from symptom onset to testing or to isolation, length of infectiousness period, or other (details noted after a colon in the data extraction report).
- **Summary statistic**: Any statistic summarizing the state, course, or severity of the epidemic in a population. Examples are the time until a certain number of cases are reached, the time until the peak of the epidemic is reached, or the time until the reproduction number fell below 1.
- **Change points**: Points in time where there is a structural change in the outcome, estimated using a change point detection method. The respective outcome used for change point detection is noted after a colon.
- **Other**: Any other computed outcome which does not fit into the above categories. Details noted in parentheses.

*Note that the reproduction number is also often used as a measure of epidemic trend (e. g. a value of $R > 1$ is interpreted as exponential spreading). We have classified it as epidemiological parameter here because of its foundation in infectious disease modeling and its frequent use in mechanistic models.

D.2.4 Method to obtain the computed outcome

If applicable, the method which is used to obtain the computed outcome (Item D.2.3). If different methods are used for different computed outcomes, the corresponding outcome is noted in parentheses.

- **Not applicable**: No computed outcome; instead, the raw outcome is used for analysis.
- **Simple computation**: Simple computation, e. g. computing the growth in cases between two consecutive time periods.
- **Exponential growth model**: Fitting of an exponential growth curve to the raw outcome (e. g. confirmed cases) in order to estimate epidemiological parameters such as the exponential growth rate or doubling time.
- **Compartmental transmission model**: (1) Compartmental single-population transmission model (no spatial structure) or (2) compartmental meta-population transmission model (several spatially distinguished meta-populations which interact to some degree, e. g. by infection across meta-populations and/or migration between meta-populations). Meta-population models usually imply a network structure between populations, and mobility data are often used to inform the interaction parameters.
- **Individual-based transmission model**: Model for transmission dynamics at the individual level, oftentimes with a network structure or via simulation of individual behavior and contact patterns.
- **Phylodynamic model**: Phylogenetic model with population dynamics that is fitted using genome sequence data.
- **Semi-mechanistic Bayesian transmission model**: Bayesian hierarchical model with a time-discrete, stochastic renewal process of transmission.
- **Statistical estimation of reproduction number**: Estimation of the reproduction number based on the time series of confirmed cases, deaths, hospitalized, etc. The exact method used to estimate the reproduction number is noted in parentheses: (1) Cori et al.[8] (estimates the instantaneous effective reproduction number); (2) Thompson et al.[10] (extension of the method by Cori et al., so that it integrates data on known pairs of index and secondary cases to

directly estimate the serial interval); (3) an der Heiden and Hamouda[9] (simplification of the method by Cori et al., assumes a fixed serial interval); (4) Wallinga and Teunis[13] (estimates the case/cohort effective reproductive number, i. e. the expected number of secondary infections that will be caused by an individual infected at time t); (5) White et al.[11] (maximum-likelihood based method for estimating the basic reproduction number); or (6) Wallinga and Lipsitch[12] (uses the exponential growth rate for estimating the basic reproduction number).

- **Statistical estimation of reproduction number from individual data**: Estimation of R based on individual case data with information to infer transmission chains. The exact method used to estimate the reproduction number from individual case data is noted in parentheses: (1) Wallinga and Teunis[13] (same method as above, but using individual data); (2) Lloyd and Smith[20] (estimates the reproduction number as the population-average of individual reproduction numbers); or (3) Diekmann et al.[21] (estimates R from the dominant eigenvalue of a next generation matrix).
- **Back projection**: Backward computation of a time series of cases by one epidemiological event (e. g. infection) from a time series of cases by another event (e. g. confirmation) using an empirical distribution of the delay between the two events.
- **Change point analysis**: Change point detection method applied to the raw outcome.
- **Other**: Other less frequent approaches that are used to estimate or compute the analyzed outcome. Details given in parentheses.

D.2.5 Data source

A categorization of the data source for the raw outcome (Item D.2.1). The specific source is given in parentheses. Note that, for (sub)national data, this is usually a governmental health authority, e. g. the department or ministry of health, the center for disease control, the health commission, or a committee. The terminology varies; e. g. sometimes the name of the authority is translated, sometimes the higher- or lower-level authority name is provided, or sometimes only the name of the database hosted by the authority is provided. Therefore, all kinds of governmental health authorities and their databases are summarized under “health authority”.

- **Could not be evaluated**: Data source for the outcome not mentioned in the manuscript.

- **Data from (sub)national authorities**: The data are accessed from a (sub)national authority, e. g. the national center for disease control.
- **Data from publicly available cross-country selections**: Examples of publicly available cross-country selections include (1) the John Hopkins University (JHU)[22]; (2) the European Center for Disease Prevention and Control (ECDC)[23]; (3) Our World In Data[19], (4) the World Health Organization (WHO)[24]; and (5) Worldometer[25].
- **Mobility data from corporate organizations**: Private/corporate organization sharing mobility data, e. g. Google COVID-19 Community Mobility Reports[26], Apple COVID-19 Mobility Trends Reports[27], or SafeGraph Places Data and Foot Traffic Insight[28].
- **Data from (sub)national public organizations**: The data are accessed directly from a (sub)national public (or not-for-profit) organization, e. g. the national statistics institute or transport organization.
- **Data from public media outlets**: Data from media outlets, e. g. the New York Times.
- **Data from other research projects**: Data from research projects, e. g. a published study or preprint.
- **Data from publicly available open-source projects**: Open-source projects where volunteers collected data from government and/or news websites and/or other sources, which they share via a publicly available repository.
- **Data collected by authors**: The study authors collected the data themselves, e. g. behavioral outcomes via surveys, seroprevalence studies, or data collected at local facilities, e. g. emergency room visits at hospitals.

D.2.6 Data availability

Data availability for the raw outcome.

- **Data not accessible**: Data are not available, i.e. no access is provided by the authors and the source of the data is not documented.

- **Data access via source**: Data accessible from original source. Of note, this does not imply that the data are easily accessible or that access is granted. In particular, we labeled raw data on epidemiological individual-level outcomes as *not accessible*, unless the study authors provided a link to the exact source of their raw data in the manuscript.
- **Data made available by the authors**: Authors make the data available, e.g. by depositing the data they used for analysis into a publicly accessible repository.

D.3 Intervention

D.3.1 Terminology for interventions

The term that is predominantly used by the authors to refer to "interventions".

- **Not applicable**: Only a term for the specific type of non-pharmaceutical interventions is used.
- **Measures**
- **Interventions**
- **Policies**
- **Strategies**
- **Orders**
- **Actions**

D.3.2 Terminology for the specific type of non-pharmaceutical interventions

The term that is predominantly used by the authors to refer to the specific type of non-pharmaceutical interventions.

- **Not applicable**: Only a term for "interventions" is used.
- **Non-pharmaceutical**
- **Social distancing**
- **Control**

- **Public health**
- **Containment**
- **Other**: Other less frequently used terms (e. g. “mitigation”, “preventive”, “government”) or terms describing particular non-pharmaceutical interventions (e. g. “stay-at-home orders” and “travel restrictions”).

D.3.3 Exposure types

Categorization of exposure types. The specific interventions are given in parentheses, e. g. “school closure” or “confinement of the population by decree of the Spanish Government on March 14”. Variations of the “Wuhan City Lockdown” or the “Indian National Lockdown” occurred frequently and are thus consistently labeled.

- **One single intervention**: One single intervention implemented on a specific date. Example: school closures implemented on March 15.
- **Multiple separate interventions**: Multiple separate interventions implemented at different dates. Example: school closures implemented on March 15 and stay-at-home orders implemented on April 1.
- **One combination of interventions**: One combination of multiple interventions implemented at the same date. Example: a lockdown on April 1 which included the closure of schools, the closure of all non-essential businesses, and a stay-at-home order.
- **Multiple combinations of interventions**: Multiple combinations of single interventions implemented at different dates, e. g. school closures and gathering bans implemented on March 15 as well as workplace closures and stay-at-home orders implemented on April 1. Of note, this category applies if there is at least one combination of multiple interventions. Example: school closures on March 15 and lockdown including workplace closures and stay-at-home orders on April 1.
- **All interventions together**: No specific interventions are analyzed but rather whether all interventions together were effective. As a result, one observes whether transmission was reduced over time as interventions were implemented.

- **Phases of interventions**: The public health response is divided into phases of interventions and the effectiveness of interventions are assessed within the phases rather than assessing the effectiveness of specific interventions implemented at a specific date. Example: the public health response is divided into two phases, namely (1) from March 15 and April 1 where school closures, bans of large gatherings, and venue closures were implemented, and (2) from April 2 to April 15 where workplace closures and stay-at-home orders were implemented.

- **Start of interventions**: Usually represented as the first date any intervention was implemented.

- **Categories of interventions**: Categorizing interventions into groups, e. g. quarantine and containment measures.

- **Stringency index**: Summarizing the stringency of interventions into a numerical index, e. g. the stringency index from the Oxford Covid-19 Government Response Tracker[29]

D.3.4 Types of single interventions

When single interventions are analyzed, the following interventions are consistently labeled:

- **Stay-at-home order**: Order to stay at home and otherwise confinement of the population, i. e. being allowed to leave the house only with few exceptions, such as going shopping or to the pharmacy.

- **School closure**: Closure of primary or secondary schools, or universities.

- **Workplace closure**: Closure of workplaces, i. e. closure of (most) non-essential businesses.

- **Venue closure**: Closure of venues, e. g. bars, restaurants, theaters, religious places, etc.

- **Declaration of a state of emergency**: Declaration of a state of emergency. Of note, in some cases, it could not be evaluated based on the manuscript if the emergency declaration was just a declaration or if it involved additional interventions.

- **Bans of large gatherings**: Ban of large gatherings of people, often the cancellation of large public events involving hundreds or thousands of people.

- **Bans of small gatherings**: Ban of small gatherings of people, often the cancellation of small private gatherings involving only a dozen of people.
- **Internal movement restrictions**: Restrictions on internal movement between subnational regions.
- **Public transport closure**: Closure of public transport.
- **International travel restrictions**: Restrictions on free international travel, i. e. restricting travel from or to some or all international destinations.
- **Mask mandate**: Compulsory order to wear masks in public indoor settings.

D.3.5 Coding of interventions

Whether coding of intervention data is necessary, i. e. the interventions and dates need to be categorized in a systematic way so that the interventions are comparable across multiple populations. For instance, one country has closed secondary schools while another has closed both primary and secondary schools. To jointly assess their effectiveness across populations, the data are reconciled by coding school closures as the closure of primary schools. Coding of data is usually necessary when multiple populations are analyzed jointly, whereas the data are usually not further reconciled when a few populations and specific interventions are analyzed separately.

- **Not applicable**: No specific interventions are analyzed.
- **Not necessary**: Coding of interventions is not necessary because the data are not analyzed jointly, i. e. interventions remain specific to the populations and no attempts are made to compare interventions between multiple populations by analyzing them jointly.
- **Necessary**: Coding of interventions is necessary because the data are analyzed jointly, i. e. interventions need to be coded so that they can be compared across multiple populations.

D.3.6 Source of intervention data

Source of intervention data. Specific source is provided in parentheses.

- **Not applicable**: No specific interventions are analyzed.
- **Could not be evaluated**: Source could not be evaluated.

- **Government or news websites**: Date and type of interventions for raw (non-coded) data collected from government or news websites.
- **Another study**: Date and type of interventions for raw (non-coded) data collected from another study.
- **Coding done by the authors**: Coding of interventions done by the authors based on data collected from government or news websites.
- **Use of externally coded data**: Coded intervention data are obtained from an external provider or another published study or preprint.

D.3.7 Availability of data on exposure

Statement regarding data availability for the exposure data.

- **Not applicable**: No specific interventions are analyzed.
- **Raw data documented in the manuscript**: Coding of data is not necessary, and the raw data are documented in the manuscript (i. e. exposure type and intervention dates).
- **Access to externally coded data via source**: Coding of data is necessary, externally coded data are used, and it is stated in the manuscript where the data can be accessed.
- **Coded data made available by the authors**: Coding of data is necessary, coding is done by the authors or externally coded data are used, and the data are made publicly available by the authors, e. g. by depositing the data into a public repository or providing a structured table of the coded data in the manuscript or supplements.
- **Coded data not available**: Coding of data is necessary, but the data are not available; i. e. the data source could not be evaluated, and the data are not made available by the authors.

D.4 Methodological approach

The methodological approach describes the stage of the effectiveness assessment. Possible intermediate steps in the analysis, which may also involve substantial methodology, are described in Section D.2.

D.4.1 Empirical approach

The general empirical approach used to assess the associations of outcomes with interventions.

- **Descriptive**: The association is assessed descriptively without explicit formulation of an outcome-intervention relationship within a mathematical model. Nevertheless, mechanistic or non-mechanistic modeling may be involved in other parts of the analysis, e. g. for computing an unobserved outcome (see Section D.2).
- **Parametric**: The association is represented by a parameter in a mechanistic or non-mechanistic model. By fitting the model to data and estimating the parameter, the association of the outcome with the intervention is quantified.
- **Counterfactual**: A counterfactual outcome representing an explicit scenario in which the intervention was not implemented is computed. This is either done by projecting the outcome from the pre-intervention period or by using outcomes in other populations. The association with the intervention is then assessed by comparing the observed outcome with the counterfactual outcome during the post-intervention period.

D.4.2 Use of exposure variation

The variation in exposure to interventions that is exploited to assess the effectiveness of interventions.

- **Only variation over time for a single population**: A single population is analyzed. Here, only variation in the exposure over time can be exploited to assess the effectiveness of interventions.
- **Only variation over time for multiple populations**: Multiple populations are analyzed, but only the variation over time is exploited, i. e. the effectiveness of interventions are assessed within each population separately.
- **Only variation between populations**: Multiple populations are analyzed, but only the variation between populations is exploited, not the variation in each population over time.
- **Both variation over time and between populations**: Multiple populations are analyzed and both variation over time and between populations are exploited.

D.4.3 Method

A categorization of the method used to assess the associations of outcomes with interventions.

- **Description of change over time**: (1) Description of time course (reported outcome or changes in the reported outcome are qualitatively described over time, sometimes by providing values for the outcome at representative time points), or (2) comparison of time periods (comparison of the outcome along time periods of different interventions, typically before and after an intervention is implemented).

- **Comparison of populations**: Comparison of populations subject to different interventions, usually comparing a population subject to an intervention with a population not subject to this intervention.

- **Comparison of change points with intervention dates**: Comparison of inferred change points in the outcome with observed dates of the interventions. If both time points are concurrent, this would be seen as evidence for the effectiveness of an intervention.

- **Non-mechanistic model**: Non-mechanistic model with a direct relationship between intervention and outcome. For example, the outcome may be an epidemiological variable such as the number of new cases, and the intervention is represented by a binary variable that equals one if the intervention is implemented and zero otherwise. Can either be (1) exponential growth model (fitting of an exponential growth equation); (2) time series model (time series model such as AR(I)MA, exponential smoothing etc.); (3) generalized linear (regression) model (including ordinary linear regression); (4) correlation (correlation coefficient computed between outcome and intervention, where intervention is usually a continuous variable representing a social distancing index); or (5) other.

- **Mechanistic model**: A model that has a structure that makes, to some extent, explicit assumptions about the mechanisms that drive infection dynamics. Can be either (1) compartmental single-population transmission model (single population, i. e. no spatial structure); (2) compartmental meta-population transmission model (several spatially distinguished meta-populations which interact to some degree, e. g. by infection across meta-populations and/or

migration between meta-populations; this typically has a network structure between meta-populations, for which often mobility data are used to inform interaction parameters); (3) semi-mechanistic Bayesian transmission model (Bayesian hierarchical model with time-discrete, stochastic renewal process); (4) individual-based transmission model (model of transmission dynamics at individual level, often with network structure or simulation of individual behavior and contact patterns); or (5) phylodynamic model (phylogenetic model with population dynamics that is fitted using genome sequence data).

- **Synthetic controls**: A method based on synthetic controls is used to compare the outcome of a target population with a synthetic scenario constructed from on a pool of "control" populations.
- **Other**: Any other method that does not fit in one of the above categories.

Addendum for mechanistic models in parametric approaches If a parametric approach is used and the method is a mechanistic model, then the parameter representing the association with an intervention must be functionally linked to some latent variable representing disease dynamics. This makes explicit how the intervention is assumed to influence disease dynamics in the model. The corresponding latent variable is recorded as follows.

- **Not applicable**: No model with a latent variable that is functionally linked to the intervention is used.
- **Reproduction number**: Intervention changes the reproduction number.
- **Transmission rate**: Intervention changes the transmission rate, i. e. effective contact rate.
- **Contact rate**: Intervention changes the contact rate.
- **Probability of transmission upon contact**: Intervention changes the probability of transmission upon contact.
- **Contact matrix**: Intervention changes individual entries of the contact matrix, representing scaled contact rates between different subpopulations (e. g. age groups).
- **Quarantine rate**: Intervention changes the rate at which individuals are quarantined.

- **Susceptible decay**: Intervention removes susceptible individuals without adding infected individuals.
- **Other**: Other type of latent variable, details noted in parentheses.

D.4.4 Code availability

Statement regarding the availability of the code. We acknowledge that many analyses, in particular descriptive analyses, would not require code to reproduce the results. Thus, this item should be analyzed within specific categories, e. g. analyses that use a parametric approach.

- **None**: No code is made publicly available.
- **Publicly available**: The code is made publicly available.

D.5 Effectiveness assessment

D.5.1 Reporting of effectiveness

How the effectiveness of interventions is reported.

- **Qualitative statement**: Effectiveness is reported without quantification, usually by qualitatively stating changes in the outcome that are likely related to the implemented interventions.
- **Comparison of outcome values**: Effectiveness is reported via a comparison of outcome values, usually by comparing the outcome value before the intervention with the outcome value after the intervention.
- **Quantification of change in outcome values**: Effectiveness is reported by quantifying changes in outcome values, usually by computing or estimating the difference in the outcome values with and without the intervention, e. g. in the form of a model parameter. Of note, in some cases, the difference between quantification of the change in and comparison of outcome values can be subtle. For instance, let B be the outcome value before an intervention and A be the outcome after an intervention. A comparison would be to note that A is lower than B, whereas a quantification would be to compute the effect of the intervention as the difference $A - B$.

D.5.2 Measure of effectiveness

On which outcome scale the effectiveness of interventions are measured.

- Change in one of the outcomes of Item D.2.1, Item D.2.3, or Item D.4.3.
- Change in any other outcome scale derived from Item D.2.1, Item D.2.3, or Item D.4.3.

D.5.3 Interpretation of results

Whether the interpretation of the results in the Abstract, Discussion and Conclusion section of the corresponding study is associative or (implicitly or explicitly) causal.

- **Associative: No causal interpretation of results is made. Findings are only expressed in terms of statistical or temporal association.**
- **Implicitly causal**: Results are not explicitly interpreted as causal effect estimates, but use of causal language suggests a causal relationship between interventions and outcomes. Causal language was classified based on a list of terms by Thapa et al.[30]
- **Explicitly causal**: Results are explicitly interpreted as estimates of the causal effects of interventions.

D.5.4 Reporting of uncertainty

Whether uncertainty regarding the effectiveness of interventions is reported. If several outcomes are analyzed, we report if uncertainty is reported for the effect of interventions on at least one outcome.

- **Not applicable**: Effectiveness is not quantified, thus uncertainty reporting is not applicable.
- **Yes**: Uncertainty is reported by providing information on imprecision, e. g. by reporting the standard deviation, standard error, confidence interval, p-value, credible interval, etc. of the estimated effectiveness of interventions. For comparison of outcome values, uncertainty is reported for the outcome values. For quantification of the change in outcome values, uncertainty is reported for the quantified change or estimated parameter.
- **No**: Effectiveness is quantified, but uncertainty is not reported.

D.5.5 Sensitivity analysis

Categorization of analyses that are conducted for assessing sensitivity with respect to the effectiveness of interventions. Detailed categories in parentheses. Note that we also considered sensitivity analyses for models used in descriptive approaches (to obtain a computed outcome) if the sensitivity was assessed with regard to the effectiveness of interventions.

- **None**: No sensitivity analyses are conducted.
- **Different coding of interventions used**: Assessing sensitivity with respect to the use of differently coded intervention data. Details in parentheses.
- **Different or modified outcome used**: Assessing sensitivity with respect to the use of different or modified outcomes. Details in parentheses.
- **Same analysis with (sub)population(s) excluded**: Assessing sensitivity with respect to the exclusion of specific (sub)populations for the same analysis.
- **Epidemiological parameters varied**: Assessing sensitivity with respect to varying assumptions regarding epidemiological parameters, e. g. serial interval or delay from infection to reporting of a new case.
- **Start or end date of study period varied**: Assessing sensitivity with respect to variations of the start and end date of the study period.
- **Model specification varied**: Assessing sensitivity with respect to changes in the model structure or specification, e. g. extending the model or adjusting the effects of interventions for additional variables.

D.5.6 Subgroup assessment

The subgroups within which the effectiveness of interventions are assessed. These subgroups are usually created by grouping the populations based on the values of some other variable.

- **None**: No subgroup assessments.
- **Socioeconomic indicators**: Subgroups based on age, gender, income (e. g. household income at the individual level or by national income at the population level), nationality, population

density, household size, health index (e. g. score for the global health security index), or occupation (i. e. workers with and without health care occupation at the individual level).

- **Epidemiological indicators**: Subgroups based on infection setting (e. g. workplace, household, school or community), pre-intervention epidemic trend (e. g. similar epidemic trends before the implementation of interventions), source of infection (whether the infection can be traced back to a local case or an imported case), isolation delay (e. g. varying delay until a person was isolated after symptom onset at the individual level), or disease severity (disease severity with COVID-19 at the individual level).
- **Public health response**: Subgroups based on response time (time till governments responded to the pandemic with an intervention) or whether being subject to other measures (e. g. whether being subject to contact tracing at the individual level or regions with/without other interventions implemented at the population level).
- **Geographic areas**: Subgroups based on regions (e. g. Europe, Asia, Middle East).

E Data extraction report

The following section provides a detailed report of all information extracted from the studies in our review sample. The corresponding items are described in the Data extraction manual. The extracted information is presented for each individual analysis, where some studies contain multiple analyses (referred to as Analysis 1, 2, etc.). Of note, some studies did not only assess the associations of mobility with non-pharmaceutical interventions, but also of population-level epidemiological outcomes with changes in mobility. We also extracted information on such complementary analyses that use "mobility as an exposure" and present them along with their main analyses of the effectiveness of intervention in the following.

Abdalla et al. 2021 . . . . **48**

Analysis 1 48

Analysis 2 49

Analysis 3, mobility as exposure 51

Abouk & Heydari 2021 . . . . **53**

Abuhasel et al. 2020 . . . . **55**

Adekunle et al. 2020 . . . . **57**

Aguiar et al. 2020 . . . . **59**

Analysis 1 59

Analysis 2 61

Akimkin et al. 2020 . . . . **63**

Aldila et al. 2020 . . . . **65**

Ali et al. 2020 . . . . **67**

Alimohamadi et al. 2020 . . . . **69**

Aloi et al. 2020 . . . . **71**

Alumran 2020 . . . . **73**

Anderson et al. 2020 . . . . **75**

Anzai et al. 2020 . . . . **77**

Aravindakshan et al. 2020 . . . . **79**

Analysis 1 . . . 79

Analysis 2, mobility as exposure . . . 81

Arshed et al. 2020 . . . 83

Asongu et al. 2020 . . . 85

Auger et al. 2020 . . . 87

Badr et al. 2020 . . . 89

Analysis 1 . . . 89

Analysis 2, mobility as exposure . . . 91

Baker et al. 2020 . . . 93

Analysis 1 . . . 93

Analysis 2 . . . 95

Banerjee & Nayak 2020 . . . 97

Bemanian et al. 2020 . . . 99

Bennett 2021 . . . 101

Bo et al. 2021 . . . 103

Bönisch et al. 2020 . . . 105

Analysis 1 . . . 105

Analysis 2, mobility as exposure . . . 107

Braithwaite et al. 2020 . . . 109

Brauner et al. 2021 . . . 111

Candido et al. 2020 . . . 113

Analysis 1 . . . 113

Analysis 2, mobility as exposure . . . 115

Cao et al. 2020 . . . 118

Castaneda & Saygili 2020 . . . 120

Cento et al. 2020 . . . 122

Chakraborty & Ghosh 2020 . . . 124

Chatterjee et al. 2020 . . . 126

Chaudhry et al. 2020 . . . 128

Chaves et al. 2020 . . . . **130**

Cheema et al. 2021 . . . . **132**

Chen et al. 2020a **134**

Chen et al. 2020b **135**

Chen et al. 2020c **137**

Cheng et al. 2020a **139**

Cheng et al. 2020b **141**

Cheng et al. 2020c **143**

Analysis 1 143

Analysis 2 145

Analysis 3, mobility as exposure 147

Cheng et al. 2020d **149**

Chernozhukov et al. 2021 . . . . **151**

Analysis 1 151

Analysis 2, mobility as exposure 153

Choi & Ki 2020 . . . . **155**

Choi et al. 2020 . . . . **157**

Cobb & Seale 2020 . . . . **159**

Coletti et al. 2020 . . . . **161**

Collins & Duffy 2020 . . . . **163**

Cooper et al. 2020 . . . . **165**

Courtemanche et al. 2020 . . . . **167**

Cowling et al. 2020 . . . . **169**

Analysis 1 169

Analysis 2 171

Crokidakis 2020 . . . . **173**

Czerwińska & Szenborn 2020 . . . . **175**

da Silva & Tsigaris 2020 . . . . **177**

Dai et al. 2020 . . . . **179**

Dandekar et al. 2020 . . . . **181**

de Brito Cruz 2020 . . . . **183**

Analysis 1 183

Analysis 2, mobility as exposure 185

Dehning et al. 2020 . . . . **187**

Dickson et al. 2020 . . . . **189**

Dikid et al. 2020 . . . . **191**

Djurović 2020 . . . . **193**

Domenico et al. 2020 . . . . **195**

Donsimoni et al. 2020 . . . . **197**

Du et al. 2020 . . . . **199**

Analysis 1 199

Analysis 2 200

Duhon et al. 2021 . . . . **202**

Džiugys et al. 2020 . . . . **204**

Edjoc et al. 2020 . . . . **206**

Eguchi et al. 2020 . . . . **208**

Fatmi 2020 . . . . **210**

Feng et al. 2020 . . . . **212**

Fernández-Recio 2020 . . . . **214**

Flaxman et al. 2020 . . . . **216**

Frank 2020 . . . . **218**

Friedrich et al. 2020 . . . . **220**

Friedson et al. 2020 . . . . **222**

Analysis 1 222

Analysis 2 224

Gallaway et al. 2020 . . . . **226**

Gao et al. 2020 . . . . **228**

Analysis 1 228

Analysis 2 230

Gatto et al. 2020 . . . . **232**

Ge et al. 2020 . . . . **234**

Ghosal et al. 2020 . . . . **236**

Gill et al. 2020 . . . . **238**

Analysis 1 238

Analysis 2 240

Gregori et al. 2020 . . . . **242**

Guirao 2020 . . . . **244**

Guo & Xiao 2020 . . . . **246**

Gupta et al. 2021 . . . . **248**

Analysis 1 248

Analysis 2 249

Hao et al. 2020 . . . . **251**

Haug et al. 2020 . . . . **253**

He et al. 2020a **255**

He et al. 2020b **257**

Henríquez et al. 2020 . . . . **259**

Analysis 1 259

Analysis 2 261

Holtz et al. 2020 . . . . **263**

Hsiang et al. 2020 . . . . **265**

Huang et al. 2020 . . . . **267**

Huber & Langen 2020 . . . . **269**

Analysis 1 269

Analysis 2 271

Analysis 3 273

Hyafil & Moriña 2021 . . . . **275**

Islam et al. 2020 . . . . **277**

Jardine et al. 2020 . . . . **279**

Jarvis et al. 2020 . . . . **281**

Jefferies et al. 2020 . . . . **283**

Jeffrey et al. 2020 . . . . **285**

Analysis 1 285

Analysis 2, mobility as exposure 287

Ji et al. 2020 . . . . **289**

Jia et al. 2020 . . . . **291**

Jüni et al. 2020 . . . . **293**

Kang & Kim 2020 . . . . **295**

Karnakov et al. 2020 . . . . **297**

Analysis 1 297

Analysis 2 299

Kaufman et al. 2020 . . . . **301**

Kendall et al. 2020 . . . . **303**

Analysis 1 303

Analysis 2 305

Khan et al. 2020 . . . . **307**

Kharroubi & Saleh 2020 . . . . **309**

Kim et al. 2020a **311**

Kim et al. 2020b **313**

Klimek-Tulwin & Tulwin 2020 . . . . **315**

Koh et al. 2020 . . . . **317**

Analysis 1 317

Analysis 2 319

Analysis 3, mobility as exposure 321

Kraemer et al. 2020 . . . . **323**

Analysis 1 323

Analysis 2 325

Analysis 3, mobility as exposure 327
Krishna 2020 329
Kucharski et al. 2020 330
Kumar et al. 2020 332
Kuo & Fu 2021 334
Lau et al. 2020 336
Analysis 1 336
Analysis 2 338
Lee et al. 2020 340
Lei et al. 2020 342
Lemaitre et al. 2020 344
Analysis 1 344
Analysis 2 346
Analysis 3, mobility as exposure 348
Li et al. 2020a 350
Li et al. 2020b 352
Li et al. 2020c 354
Analysis 1 354
Analysis 2 356
Li et al. 2021 358
Lim et al. 2020a 360
Analysis 1 360
Analysis 2, mobility as exposure 362
Lim et al. 2020b 364
Linka et al. 2020 366
Analysis 1 366
Analysis 2, mobility as exposure 368
Liu et al. 2020a 370
Liu et al. 2020b 372

Analysis 1 372
Analysis 2, mobility as exposure 373
Loeffler-Wirth et al. 2020 . . . . . **375**
Lurie et al. 2020 . . . . . **377**
Analysis 1 377
Analysis 2 379
Maier & Brockmann 2020 . . . . . **381**
Malheiro et al. 2020 . . . . . **383**
Manevski et al. 2020 . . . . . **385**
Marschner 2020 . . . . . **387**
McCarthy et al. 2020 . . . . . **389**
McGrail et al. 2020 . . . . . **392**
McKee et al. 2020 . . . . . **393**
McKenzie & Adams 2020 . . . . . **395**
Medline et al. 2020 . . . . . **397**
Miller et al. 2020 . . . . . **399**
Mitra et al. 2020 . . . . . **401**
Moon et al. 2020 . . . . . **403**
Moreno et al. 2020 . . . . . **405**
Moshammer et al. 2020 . . . . . **407**
Mukandavire et al. 2020 . . . . . **409**
Munayco et al. 2020 . . . . . **411**
Murillo-Zamora et al. 2020 . . . . . **413**
Ng et al. 2020 . . . . . **415**
Niazkar et al. 2020 . . . . . **417**
Analysis 1 417
Analysis 2 419
Nie et al. 2020 . . . . . **421**
Niwa et al. 2020 . . . . . **423**

Odagaki 2020 . . . . **425**

Ouchetto et al. 2020 . . . . **427**

Pan et al. 2020a **429**

Pan et al. 2020b **431**

Pang et al. 2020 . . . . **433**

Pei et al. 2020 . . . . **435**

Peixoto et al. 2020 . . . . **437**

Peng et al. 2020 . . . . **438**

Pépin et al. 2020 . . . . **440**

Piovani et al. 2021 . . . . **442**

Price et al. 2020 . . . . **444**

Pullano et al. 2020 . . . . **446**

Analysis 1 446

Analysis 2 448

Analysis 3 450

Pung et al. 2020 . . . . **452**

Qiu et al. 2020 . . . . **454**

Analysis 1 454

Analysis 2 456

Quaife et al. 2020 . . . . **458**

Rahman et al. 2020 . . . . **461**

Analysis 1 461

Analysis 2, mobility as exposure 462

Reis et al. 2020 . . . . **464**

Riccardo et al. 2020 . . . . **466**

Rieger & Wang 2020 . . . . **468**

Ryu et al. 2020 . . . . **470**

Saez et al. 2020 . . . . **472**

Salje et al. 2020 . . . . **474**

Salvatore et al. 2020 476

Santamaria et al. 2020 478

Saurabh et al. 2020 480

Scarabel et al. 2020 482

Sebastian et al. 2020 484

Analysis 1 484

Analysis 2 486

Sebastiani et al. 2020 488

Seemann et al. 2020 490

Sehra et al. 2020 492

Analysis 1 492

Analysis 2, mobility as exposure 494

Sekine et al. 2020 496

Sewell & And 2020 498

Analysis 1 498

Analysis 2 500

Shanmugam 2020 502

Shi et al. 2020a 504

Analysis 1 504

Analysis 2 506

Shi et al. 2020b 508

Shim et al. 2021 510

Siedner et al. 2020 512

Signorelli et al. 2020 514

Silva et al. 2020 516

Singh & Singh 2020 518

Singh et al. 2020 519

Sinha 2020 521

Spaccaferri et al. 2020 523

Sun et al. 2020 . . . . **525**

Supino et al. 2020 . . . . **527**

Suraya et al. 2020 . . . . **529**

Analysis 1 529

Analysis 2 531

Talmoudi et al. 2020 . . . . **533**

Tamagusko & Ferreira 2020 . . . . **536**

Analysis 1 536

Analysis 2 537

Tchole et al. 2020 . . . . **539**

Thai et al. 2020 . . . . **541**

Tian et al. 2020 . . . . **544**

Analysis 1 544

Analysis 2 546

Tian et al. 2021 . . . . **547**

Tiwari et al. 2020 . . . . **549**

Tsai et al. 2020 . . . . **551**

Turk et al. 2020 . . . . **553**

Tutsoy et al. 2020 . . . . **555**

Utamura et al. 2020 . . . . **557**

Valcarcel et al. 2020 . . . . **559**

Valencia et al. 2020 . . . . **561**

Analysis 1 561

Analysis 2 563

Van Dyke et al. 2020 . . . . **565**

Vannoni et al. 2020 . . . . **567**

Verma et al. 2020 . . . . **569**

Vicentini et al. 2020 . . . . **571**

Wagner et al. 2020 . . . . **573**

Wahaibi et al. 2020 . . . . **575**

Wan et al. 2020 . . . . **577**

Wang et al. 2020a **579**

Wang et al. 2020b **581**

Wang et al. 2020c **583**

Analysis 1 583

Analysis 2 585

Wang et al. 2020d **587**

Wang et al. 2020e **589**

Wells et al. 2020 . . . . **591**

Wibbens et al. 2020 . . . . **593**

Wieland 2020 . . . . **595**

Analysis 1 595

Analysis 2 597

Wielechowski et al. 2020 . . . . **599**

Analysis 1 599

Analysis 2, mobility as exposure 601

Wong et al. 2020a **603**

Wong et al. 2020b **605**

Analysis 1 605

Analysis 2, mobility as exposure 607

Wong et al. 2020c **609**

Analysis 1 609

Analysis 2 611

Wu et al. 2020 . . . . **613**

Analysis 1 613

Analysis 2 615

Xia et al. 2020 . . . . **616**

Xiao et al. 2020 . . . . **618**

Xu et al. 2020 . . . . **620**

Analysis 1 620

Analysis 2 622

Analysis 3, mobility as exposure 624

Yang et al. 2020 . . . . **626**

Ye & Hu 2020 . . . . **628**

Yehya et al. 2020 . . . . **630**

You 2020 . . . . **632**

You et al. 2020 . . . . **634**

Yuan et al. 2020 . . . . **636**

Zhang et al. 2020a **638**

Zhang et al. 2020b **639**

Zhang et al. 2020c **641**

Zhang et al. 2020d **643**

Zhao & Chen 2020 . . . . **645**

Zhao et al. 2020 . . . . **647**

Abdalla et al. 2021 (Analysis 1)	
**Study information**	
Author	Abdalla M, Abar A, Beiter ER, Saad M
Title	Asynchrony between Individual and Government Actions Accounts for Disproportionate Impact of COVID-19 on Vulnerable Communities
Year	2021
Journal	American Journal of Preventive Medicine
DOI	10.1016/j.amepre.2020.10.012
Date received	– could not be evaluated –
Date accepted	2020-11-13 (published)
**Study setting**	
Number of populations included	multiple
Level of populations included	both national and subnational (1124 US counties in 42 US states)
Geographic areas covered	North America
Number of countries covered	single country (US)
Study period	end date could not be evaluated
**Outcome**	
Raw outcome	epidemiological population-level outcome (cases)
Time resolution of raw outcome	daily
Computed outcome	none
Method to obtain the computed outcome	– not applicable –
Data source	data from public media outlets (New York Times)
Data availability	data access via source

– continued on next page –

**Intervention**	
Terminology for interventions	actions
Terminology for the specific type of non-pharmaceutical interventions	government
Exposure types	one single intervention (stay-at-home order)
Types of single interventions	stay-at-home order
Coding of interventions	necessary
Source of intervention data	– could not be evaluated –
Availability of data on exposure	coded data not available
**Methodological approach**	
Empirical approach	parametric
Use of exposure variation	both variation over time and between populations
Method	non-mechanistic model (generalized linear model)
Code availability	none
**Effectiveness assessment**	
Reporting of intervention effectiveness	quantification of change in outcome values
Measure of effectiveness	cases
Interpretation of results	implicitly causal
Reporting of uncertainty	yes
Sensitivity analysis	epidemiological parameters varied
Subgroup assessment	none

Abdalla et al. 2021 (Analysis 2)	
**Study information**	
Author	Abdalla M, Abar A, Beiter ER, Saad M
Title	Asynchrony between Individual and Government Actions Accounts for Disproportionate Impact of COVID-19 on Vulnerable Communities
Year	2021
Journal	American Journal of Preventive Medicine
DOI	10.1016/j.amepre.2020.10.012
Date received	– could not be evaluated –
Date accepted	2020-11-13 (published)
**Study setting**	
Number of populations included	multiple
Level of populations included	both national and subnational (1124 US counties in 42 US states)
Geographic areas covered	North America
Number of countries covered	single country (US)
Study period	end date could not be evaluated
**Outcome**	
Raw outcome	behavioral outcome (mobility: time spent at home)
Time resolution of raw outcome	daily
Computed outcome	change points (change points: mobility)
Method to obtain the computed outcome	change point analysis
Data source	mobility data from corporate organizations (Google)
Data availability	data access via source
**Intervention**	
Terminology for interventions	actions

– continued on next page –

**Abdalla et al. 2021 (Analysis 2)** – continued from previous page	
Terminology for the specific type of non-pharmaceutical interventions	government
Exposure types	one single intervention (stay-at-home order)
Types of single interventions	stay-at-home order
Coding of interventions	necessary
Source of intervention data	– could not be evaluated –
Availability of data on exposure	coded data not available
**Methodological approach**	
Empirical approach	descriptive
Use of exposure variation	both variation over time and between populations
Method	comparison of change points with intervention
Code availability	none
**Effectiveness assessment**	
Reporting of intervention effectiveness	quantification of change in outcome values
Measure of effectiveness	change points: mobility
Interpretation of results	associative
Reporting of uncertainty	no
Sensitivity analysis	none
Subgroup assessment	none

**Abdalla et al. 2021 (Analysis 3, mobility as exposure)**	
**Study information**	
Author	Abdalla M, Abar A, Beiter ER, Saad M

– continued on next page –

Title	Asynchrony between Individual and Government Actions Accounts for Disproportionate Impact of COVID-19 on Vulnerable Communities
Year	2021
Journal	American Journal of Preventive Medicine
DOI	10.1016/j.amepre.2020.10.012
Date received	– could not be evaluated –
Date accepted	13.11.2020 (published)
**Study setting**	
Number of populations included	multiple
Level of populations included	both national and subnational (1124 US counties in 42 US states)
Geographic areas covered	North America
Number of countries covered	single country (US)
Study period	end date could not be evaluated
**Outcome**	
Raw outcome	epidemiological population-level outcome (cases)
Time resolution of raw outcome	daily
Computed outcome	none
Method to obtain the computed outcome	– not applicable –
Data source	data from public media outlets (New York Times)
Data availability	data access via source
**Intervention**	
Terminology for interventions	actions
Terminology for the specific type of non-pharmaceutical interventions	government
Exposure types	change points (mobility: time spent at home)

– continued on next page –

Types of single interventions	– not applicable –
Coding of interventions	– not applicable –
Source of intervention data	mobility data by corporate organizations (Google)
Availability of data on exposure	data access via source
**Methodological approach**	
Empirical approach	parametric
Use of exposure variation	both variation over time and between populations
Method	non-mechanistic model (generalized linear model)
Code availability	none
**Effectiveness assessment**	
Reporting of intervention effectiveness	quantification of change in outcome values
Measure of effectiveness	cases
Interpretation of results	implicitly causal
Reporting of uncertainty	yes
Sensitivity analysis	epidemiological parameters varied
Subgroup assessment	none

Abouk & Heydari 2021

**Study information**	
Author	Abouk R, Heydari B
Title	The Immediate Effect of COVID-19 Policies on Social-Distancing Behavior in the United States
Year	2021

– continued on next page –

Journal	Public Health Reports
DOI	10.1177/0033354920976575
Date received	– could not be evaluated –
Date accepted	2020-01-05 (published)
**Study setting**	
Number of populations included	multiple
Level of populations included	both national and subnational (50 US states and the District of Columbia)
Geographic areas covered	North America
Number of countries covered	single country (US)
Study period	start and end date span first epidemic wave: behavioral
**Outcome**	
Raw outcome	behavioral outcome (mobility: time spent in various locations)
Time resolution of raw outcome	– not applicable –
Computed outcome	none
Method to obtain the computed outcome	– not applicable –
Data source	mobility data from corporate organizations (Google)
Data availability	data access via source
**Intervention**	
Terminology for interventions	policies
Terminology for the specific type of non-pharmaceutical interventions	social distancing
Exposure types	multiple separate interventions (stay- at-home order, limited stay- at-home order, workplace closure, bans of large gatherings, school closure, venue closure)

– continued on next page –

Types of single interventions	stay-at-home order, workplace closure, bans of large gatherings, school closure, venue closure
Coding of interventions	necessary
Source of intervention data	use of externally coded data (Kaiser Family Foundation)
Availability of data on exposure	access to externally coded data via source
**Methodological approach**	
Empirical approach	parametric
Use of exposure variation	both variation over time and between populations
Method	non-mechanistic model (generalized linear model)
Code availability	none
**Effectiveness assessment**	
Reporting of intervention effectiveness	quantification of change in outcome values
Measure of effectiveness	mobility: time spent in various locations
Interpretation of results	implicitly causal
Reporting of uncertainty	yes
Sensitivity analysis	same analysis with (sub)population(s) excluded, model specification varied, different coding of interventions used
Subgroup assessment	none

Abuhasel et al. 2020

**Study information**	
Author	Abuhasel KA, Khadr M, Alquraish MM
Title	Analyzing and Forecasting COVID -19 Pandemic in the Kingdom of Saudi Arabia Using ARIMA and SIR Models

– continued on next page –

Year	2020
Journal	Computational Intelligence
DOI	10.1111/coin.12407
Date received	2020-06-01
Date accepted	2020-09-06
**Study setting**	
Number of populations included	single
Level of populations included	national (Saudi Arabia)
Geographic areas covered	Middle East and Africa
Number of countries covered	single country (Saudi Ariba)
Study period	end date at peak of wave
**Outcome**	
Raw outcome	epidemiological population-level outcome (cases, deaths, recovered cases)
Time resolution of raw outcome	daily
Computed outcome	none
Method to obtain the computed outcome	– not applicable –
Data source	data from (sub)national authorities
Data availability	data access via source
**Intervention**	
Terminology for interventions	strategies
Terminology for the specific type of non-pharmaceutical interventions	containment, control
Exposure types	all interventions together
Types of single interventions	– not applicable –
Coding of interventions	– not applicable –

– continued on next page –

Source of intervention data	– not applicable –
Availability of data on exposure	– not applicable –
**Methodological approach**	
Empirical approach	counterfactual
Use of exposure variation	only variation over time for a single population
Method	mechanistic model (compartmental single-population transmission model)
Code availability	none
**Effectiveness assessment**	
Reporting of intervention effectiveness	comparison of outcome values
Measure of effectiveness	infections
Interpretation of results	implicitly causal
Reporting of uncertainty	no
Sensitivity analysis	none
Subgroup assessment	none

Adekunle et al. 2020

**Study information**	
Author	Adekunle A, Meehan M, Rojas-Alvarez D, Trauer J, McBryde E
Title	Delaying the COVID-19 Epidemic in Australia: Evaluating the Effectiveness of International Travel Bans
Year	2020
Journal	Australian and New Zealand Journal of Public Health
DOI	10.1111/1753-6405.13016

– continued on next page –

Date received	2020-04-01
Date accepted	2020-05-01
**Study setting**	
Number of populations included	single
Level of populations included	national (Australia)
Geographic areas covered	Oceania
Number of countries covered	single country (Australia)
Study period	end date at peak of wave
**Outcome**	
Raw outcome	epidemiological population-level outcome (imported cases)
Time resolution of raw outcome	daily
Computed outcome	none
Method to obtain the computed outcome	– not applicable –
Data source	data from other research projects (EpiForecasts project by the London School of Hygiene & Tropical Medicine or study by Abbott et al.)
Data availability	data access via source
**Intervention**	
Terminology for interventions	interventions
Terminology for the specific type of non-pharmaceutical interventions	– not applicable –
Exposure types	one single intervention (travel restrictions (Australian travel bans on travellers from Wuhan, China, Iran, South Korea, Italy))
Types of single interventions	international travel restrictions
Coding of interventions	not necessary
Source of intervention data	– could not be evaluated –

– continued on next page –

Availability of data on exposure	raw data documented in the manuscript
**Methodological approach**	
Empirical approach	counterfactual
Use of exposure variation	only variation over time for a single population
Method	mechanistic model (compartmental metapopulation transmission model)
Code availability	none
**Effectiveness assessment**	
Reporting of intervention effectiveness	quantification of change in outcome values
Measure of effectiveness	imported cases
Interpretation of results	implicitly causal
Reporting of uncertainty	no
Sensitivity analysis	none
Subgroup assessment	none

Aguiar et al. 2020 (Analysis 1)

**Study information**	
Author	Aguiar M, Ortuondo EM, Van-Dierdonck JB, Mar J, Stollenwerk N
Title	Modelling COVID 19 in the Basque Country from Introduction to Control Measure Response
Year	2020
Journal	Scientific Reports
DOI	10.1038/s41598-020-74386-1

– continued on next page –

Date received	2020-07-20
Date accepted	2020-09-28
**Study setting**	
Number of populations included	single
Level of populations included	subnational (Basque)
Geographic areas covered	Europe
Number of countries covered	single country (Spain)
Study period	start and end date span first epidemic wave
**Outcome**	
Raw outcome	epidemiological population-level outcome (cases, hospitalizations, ICU hospitalizations, recovered cases, deaths)
Time resolution of raw outcome	daily
Computed outcome	none
Method to obtain the computed outcome	– not applicable –
Data source	data from (sub)national authorities
Data availability	data access via source
**Intervention**	
Terminology for interventions	measures
Terminology for the specific type of non-pharmaceutical interventions	control
Exposure types	all interventions together
Types of single interventions	– not applicable –
Coding of interventions	– not applicable –
Source of intervention data	– not applicable –
Availability of data on exposure	– not applicable –

– continued on next page –

**Methodological approach**	
Empirical approach	counterfactual
Use of exposure variation	only variation over time for a single population
Method	mechanistic model (compartmental single-population transmission model)
Code availability	none
**Effectiveness assessment**	
Reporting of intervention effectiveness	qualitative statement
Measure of effectiveness	cases, hospitalizations, ICU hospitalizations, recovered, deaths
Interpretation of results	implicitly causal
Reporting of uncertainty	– not applicable –
Sensitivity analysis	none
Subgroup assessment	none

Aguiar et al. 2020 (Analysis 2)

**Study information**	
Author	Aguiar M, Ortuondo EM, Van-Dierdonck JB, Mar J, Stollenwerk N
Title	Modelling COVID 19 in the Basque Country from Introduction to Control Measure Response
Year	2020
Journal	Scientific Reports
DOI	10.1038/s41598-020-74386-1
Date received	2020-07-20
Date accepted	2020-09-28
**Study setting**	

– continued on next page –

Number of populations included	single
Level of populations included	subnational (Basque)
Geographic areas covered	Europe
Number of countries covered	single country (Spain)
Study period	start and end date span first epidemic wave
**Outcome**	
Raw outcome	epidemiological population-level outcome (cases, hospitalizations, ICU hospitalizations, recovered cases, deaths)
Time resolution of raw outcome	daily
Computed outcome	measure of epidemic trend (growth rate, growth rate: hospitalized, growth rate: ICU hospitalized, growth rate: recovered, growth rate: deaths), epidemiological parameter (reproduction number)
Method to obtain the computed outcome	simple computation (growth rate), compartmental transmission model (compartmental single-population transmission model: reproduction number)
Data source	data from (sub)national authorities
Data availability	data access via source
**Intervention**	
Terminology for interventions	measures
Terminology for the specific type of non-pharmaceutical interventions	control
Exposure types	all interventions together
Types of single interventions	– not applicable –
Coding of interventions	– not applicable –
Source of intervention data	– not applicable –
Availability of data on exposure	– not applicable –
**Methodological approach**	

– continued on next page –

Empirical approach	descriptive
Use of exposure variation	only variation over time for a single population
Method	description of change over time (description of time course)
Code availability	none
**Effectiveness assessment**	
Reporting of intervention effectiveness	qualitative statement
Measure of effectiveness	reproduction number, growth rate, growth rate: hospitalizations, growth rate: ICU hospitalizations, growth rate: recovered, growth rate: deaths
Interpretation of results	implicitly causal
Reporting of uncertainty	– not applicable –
Sensitivity analysis	none
Subgroup assessment	none

Akimkin et al. 2020

**Study information**	
Author	Akimkin VG, Kuzin SN, Semenenko TA, Shipulina OY, Yatsyshina SB, Tivanova EV, Kalenskaya AV, Solovyova IV, Vershinina MA, Kvasova OA, Ploskireva AA, Mamoshina MV, Elkina MA, Klushkina VV, Andreeva EE, Ivanenko AV
Title	Patterns of the SARS-CoV-2 Epidemic Spread in a Megacity
Year	2020
Journal	Problems of Virology
DOI	10.36233/0507-4088-2020-65-4-203-211
Date received	2020-07-14

– continued on next page –

Date accepted	2020-08-04
**Study setting**	
Number of populations included	single
Level of populations included	subnational (Moscow)
Geographic areas covered	Asia, Europe
Number of countries covered	single country (Russia)
Study period	start and end date span first epidemic wave
**Outcome**	
Raw outcome	epidemiological population-level outcome (cases)
Time resolution of raw outcome	daily
Computed outcome	measure of epidemic trend (growth rate)
Method to obtain the computed outcome	simple computation
Data source	data from (sub)national authorities (Government of the Russian Federation)
Data availability	data access via source
**Intervention**	
Terminology for interventions	measures
Terminology for the specific type of non-pharmaceutical interventions	prevention
Exposure types	all interventions together (timeline of measures)
Types of single interventions	– not applicable –
Coding of interventions	– not applicable –
Source of intervention data	– not applicable –
Availability of data on exposure	– not applicable –
**Methodological approach**	
Empirical approach	descriptive

– continued on next page –

Use of exposure variation	only variation over time for a single population
Method	description of change over time (comparison of time periods)
Code availability	none
**Effectiveness assessment**	
Reporting of intervention effectiveness	comparison of outcome values
Measure of effectiveness	growth rate, cases
Interpretation of results	implicitly causal
Reporting of uncertainty	yes
Sensitivity analysis	none
Subgroup assessment	none

Aldila et al. 2020

**Study information**	
Author	Aldila D, Khoshnaw SH, Safitri E, Anwar YR, Bakry AR, Samiadji BM, Anugerah DA, Gh MF, Ayulani ID, Salim SN
Title	A Mathematical Study on the Spread of COVID-19 Considering Social Distancing and Rapid Assessment: The Case of Jakarta, Indonesia
Year	2020
Journal	Chaos, Solitons & Fractals
DOI	10.1016/j.chaos.2020.110042
Date received	2020-05-22
Date accepted	2020-06-18
**Study setting**	
Number of populations included	single

– continued on next page –

Level of populations included	subnational (Jakarta)
Geographic areas covered	Asia
Number of countries covered	single country (Indonesia)
Study period	end date in growth phase of wave
**Outcome**	
Raw outcome	epidemiological population-level outcome (hospitalizations, cases)
Time resolution of raw outcome	daily
Computed outcome	epidemiological parameter (reproduction number, transmission rate, hospitalization rate)
Method to obtain the computed outcome	compartmental transmission model (single-population)
Data source	– could not be evaluated –
Data availability	data made available by the authors
**Intervention**	
Terminology for interventions	– not applicable –
Terminology for the specific type of non-pharmaceutical interventions	social distancing
Exposure types	one combination of interventions (closure of schools and venues)
Types of single interventions	– not applicable –
Coding of interventions	not necessary
Source of intervention data	government or news websites
Availability of data on exposure	raw data documented in the manuscript
**Methodological approach**	
Empirical approach	descriptive
Use of exposure variation	only variation over time for a single population
Method	description of change over time (comparison of time periods)

– continued on next page –

Code availability	none
**Effectiveness assessment**	
Reporting of intervention effectiveness	quantification of change in outcome values
Measure of effectiveness	reproduction number, transmission rate, hospitalization rate
Interpretation of results	implicitly causal
Reporting of uncertainty	no
Sensitivity analysis	none
Subgroup assessment	none

Ali et al. 2020

**Study information**	
Author	Ali ST, Wang L, Lau EH, Xu XK, Du Z, Wu Y, Leung GM, Cowling BJ
Title	Serial Interval of SARS-CoV-2 Was Shortened over Time by Nonpharmaceutical Interventions
Year	2020
Journal	Science
DOI	10.1126/science.abc9004
Date received	2020-05-20
Date accepted	2020-07-13
**Study setting**	
Number of populations included	single
Level of populations included	national (China)
Geographic areas covered	Asia

– continued on next page –

Number of countries covered	single country (China)
Study period	start and end date span first epidemic wave
**Outcome**	
Raw outcome	epidemiological individual-level outcome (individual cases and transmission chains)
Time resolution of raw outcome	daily
Computed outcome	epidemiological parameter (serial interval)
Method to obtain the computed outcome	simple computation
Data source	data from (sub)national authorities
Data availability	data made available by the authors
**Intervention**	
Terminology for interventions	interventions
Terminology for the specific type of non-pharmaceutical interventions	non-pharmaceutical
Exposure types	all interventions together
Types of single interventions	– not applicable –
Coding of interventions	– not applicable –
Source of intervention data	– not applicable –
Availability of data on exposure	– not applicable –
**Methodological approach**	
Empirical approach	descriptive
Use of exposure variation	only variation over time for a single population
Method	description of change over time (description of time course)
Code availability	publicly available
**Effectiveness assessment**	

– continued on next page –

Reporting of intervention effectiveness	quantification of change in outcome values
Measure of effectiveness	serial interval
Interpretation of results	implicitly causal
Reporting of uncertainty	yes
Sensitivity analysis	none
Subgroup assessment	socioeconomic indicators (age, gender), epidemiological indicators (infection setting, isolation delay)

Alimohamadi et al. 2020

**Study information**	
Author	Alimohamadi Y, Holakouie-Naieni K, Sepandi M, Taghdir M
Title	Effect of Social Distancing on COVID-19 Incidence and Mortality in Iran Since February 20 to May 13, 2020: An Interrupted Time Series Analysis
Year	2020
Journal	Risk Management and Healthcare Policy
DOI	10.2147/rmhp.s265079
Date received	2020-06-08
Date accepted	2020-08-29
**Study setting**	
Number of populations included	single
Level of populations included	national (Iran)
Geographic areas covered	Middle East and Africa
Number of countries covered	single country (Iran)

– continued on next page –

Study period	start and end date span first epidemic wave
**Outcome**	
Raw outcome	epidemiological population-level outcome (cases, deaths)
Time resolution of raw outcome	daily
Computed outcome	none
Method to obtain the computed outcome	– not applicable –
Data source	data from publicly available cross-country selections (OurWorldInData)
Data availability	data access via source
**Intervention**	
Terminology for interventions	measures
Terminology for the specific type of non-pharmaceutical interventions	social distancing
Exposure types	one single intervention (venue closure)
Types of single interventions	venue closure
Coding of interventions	not necessary
Source of intervention data	– could not be evaluated –
Availability of data on exposure	raw data documented in the manuscript
**Methodological approach**	
Empirical approach	parametric
Use of exposure variation	only variation over time for a single population
Method	non-mechanistic model (generalized linear model)
Code availability	none
**Effectiveness assessment**	
Reporting of intervention effectiveness	quantification of change in outcome values

– continued on next page –

Measure of effectiveness	cases, deaths
Interpretation of results	implicitly causal
Reporting of uncertainty	yes
Sensitivity analysis	none
Subgroup assessment	none

Aloi et al. 2020

**Study information**	
Author	Aloi A, Alonso B, Benavente J, Cordera R, Echániz E, González F, Ladisa C, Lezama-Romanelli R, López-Parra Á, Mazzei V, Perrucci L, Prieto-Quintana D, Rodríguez A, Sañudo R
Title	Effects of the COVID-19 Lockdown on Urban Mobility: Empirical Evidence from the City of Santander (Spain)
Year	2020
Journal	Sustainability
DOI	10.3390/su12093870
Date received	2020-04-14
Date accepted	2020-05-05
**Study setting**	
Number of populations included	single
Level of populations included	subnational (Santander (Spain))
Geographic areas covered	Europe
Number of countries covered	single country (Spain)
Study period	start and end date span first epidemic wave: behavioral
**Outcome**	

– continued on next page –

Raw outcome	behavioral outcome (mobility: public transport data & sensors and surveillance cams)
Time resolution of raw outcome	daily
Computed outcome	none
Method to obtain the computed outcome	– not applicable –
Data source	data from (sub)national authorities (Santander City Council)
Data availability	data not accessible
**Intervention**	
Terminology for interventions	measures
Terminology for the specific type of non-pharmaceutical interventions	confinement, quarantine
Exposure types	one combination of interventions (confinement of the population imposed by the Spanish Government on March 15)
Types of single interventions	– not applicable –
Coding of interventions	not necessary
Source of intervention data	– could not be evaluated –
Availability of data on exposure	raw data documented in the manuscript
**Methodological approach**	
Empirical approach	descriptive
Use of exposure variation	only variation over time for a single population
Method	description of change over time (comparison of time periods)
Code availability	none
**Effectiveness assessment**	
Reporting of intervention effectiveness	quantification of change in outcome values

– continued on next page –

Measure of effectiveness	mobility: various measures of within-city mobility for motor vehicles & public transport & walking
Interpretation of results	associative
Reporting of uncertainty	no
Sensitivity analysis	none
Subgroup assessment	none

Alumran 2020

**Study information**	
Author	Alumran A
Title	Role of Precautionary Measures in Containing the Natural Course of Novel Coronavirus Disease
Year	2020
Journal	Journal of Multidisciplinary Healthcare
DOI	10.2147/jmdh.s261643
Date received	2020-05-12
Date accepted	2020-07-02
**Study setting**	
Number of populations included	single
Level of populations included	national (Saudi Arabia)
Geographic areas covered	Middle East and Africa
Number of countries covered	single country (Saudi Ariba)
Study period	end date in growth phase of wave
**Outcome**	

– continued on next page –

Raw outcome	epidemiological population-level outcome (cases, deaths)
Time resolution of raw outcome	daily
Computed outcome	none
Method to obtain the computed outcome	– not applicable –
Data source	data from (sub)national authorities (cases and deaths), data from publicly available cross-country selections (Worldometer: cases and deaths)
Data availability	data access via source
**Intervention**	
Terminology for interventions	measures
Terminology for the specific type of non-pharmaceutical interventions	prevention
Exposure types	all interventions together (timeline of measures)
Types of single interventions	– not applicable –
Coding of interventions	– not applicable –
Source of intervention data	– not applicable –
Availability of data on exposure	– not applicable –
**Methodological approach**	
Empirical approach	counterfactual
Use of exposure variation	only variation over time for a single population
Method	non-mechanistic model (exponential growth model)
Code availability	none
**Effectiveness assessment**	
Reporting of intervention effectiveness	quantification of change in outcome values
Measure of effectiveness	cases, deaths

– continued on next page –

Interpretation of results	implicitly causal
Reporting of uncertainty	no
Sensitivity analysis	none
Subgroup assessment	none

Anderson et al. 2020

**Study information**	
Author	Anderson SC, Edwards AM, Yerlanov M, Mulberry N, Stockdale JE, Iyaniwura SA, Falcao RC, Otterstatter MC, Irvine MA, Janjua NZ, Coombs D, Colijn C
Title	Quantifying the Impact of COVID-19 Control Measures Using a Bayesian Model of Physical Distancing
Year	2020
Journal	PLOS Computational Biology
DOI	10.1371/journal.pcbi.1008274
Date received	2020-05-21
Date accepted	2020-08-19
**Study setting**	
Number of populations included	multiple
Level of populations included	both national and subnational (British Columbia, New York, Washington, Florida, California, New Zealand)
Geographic areas covered	North America
Number of countries covered	single country (Canada)
Study period	same end date for several populations with diverse epidemic trajectories
**Outcome**	

– continued on next page –

Raw outcome	epidemiological population-level outcome (cases)
Time resolution of raw outcome	daily
Computed outcome	none
Method to obtain the computed outcome	– not applicable –
Data source	data from (sub)national authorities (British Columbia and New Zealand), data from public media outlets (COVID Tracking Project by The Atlantic: US)
Data availability	data made available by the authors
**Intervention**	
Terminology for interventions	measures
Terminology for the specific type of non-pharmaceutical interventions	physical distancing
Exposure types	all interventions together
Types of single interventions	– not applicable –
Coding of interventions	– not applicable –
Source of intervention data	– not applicable –
Availability of data on exposure	– not applicable –
**Methodological approach**	
Empirical approach	parametric
Use of exposure variation	only variation over time for multiple populations
Method	mechanistic model (compartmental single-population transmission model)
Latent variable functionally linked to intervention	contact rate
Code availability	publicly available
**Effectiveness assessment**	

– continued on next page –

Reporting of intervention effectiveness	quantification of change in outcome values
Measure of effectiveness	contact rate
Interpretation of results	associative
Reporting of uncertainty	yes
Sensitivity analysis	epidemiological parameters varied
Subgroup assessment	none

Anzai et al. 2020

**Study information**	
Author	Anzai A, Kobayashi T, Linton NM, Kinoshita R, Hayashi K, Suzuki A, Yang Y, Jung SM, Miyama T, Akhmetzhanov AR, Nishiura H
Title	Assessing the Impact of Reduced Travel on Exportation Dynamics of Novel Coronavirus Infection (COVID-19)
Year	2020
Journal	Journal of Clinical Medicine
DOI	10.3390/jcm9020601
Date received	2020-02-13
Date accepted	2020-02-20
**Study setting**	
Number of populations included	multiple
Level of populations included	national (Japan (imported cases from China))
Geographic areas covered	Asia
Number of countries covered	single country (Japan)

– continued on next page –

Study period	end date in growth phase of wave
**Outcome**	
Raw outcome	epidemiological population-level outcome (cases)
Time resolution of raw outcome	daily
Computed outcome	none
Method to obtain the computed outcome	– not applicable –
Data source	data collected by authors (from data from (sub)national authorities and news websites quoting official outbreak reports)
Data availability	data made available by the authors
**Intervention**	
Terminology for interventions	– not applicable –
Terminology for the specific type of non-pharmaceutical interventions	travel restrictions
Exposure types	one combination of interventions (Wuhan City Lockdown)
Types of single interventions	– not applicable –
Coding of interventions	not necessary
Source of intervention data	– could not be evaluated –
Availability of data on exposure	raw data documented in the manuscript
**Methodological approach**	
Empirical approach	counterfactual
Use of exposure variation	both variation over time and between populations
Method	non-mechanistic model (generalized linear model)
Code availability	none
**Effectiveness assessment**	

– continued on next page –

Reporting of intervention effectiveness	quantification of change in outcome values
Measure of effectiveness	cases
Interpretation of results	implicitly causal
Reporting of uncertainty	yes
Sensitivity analysis	epidemiological parameters varied
Subgroup assessment	none

Aravindakshan et al. 2020 (Analysis 1)

**Study information**	
Author	Aravindakshan A, Boehnke J, Gholami E, Nayak A
Title	Preparing for a Future COVID-19 Wave: Insights and Limitations from a Data-Driven Evaluation of Non-Pharmaceutical Interventions in Germany
Year	2020
Journal	Scientific Reports
DOI	10.1038/s41598-020-76244-6
Date received	2020-07-01
Date accepted	2020-10-26
**Study setting**	
Number of populations included	multiple
Level of populations included	both national and subnational (Germanys states)
Geographic areas covered	Europe
Number of countries covered	single country (Germany)

– continued on next page –

Study period	start and end date span first epidemic wave: behavioral
**Outcome**	
Raw outcome	behavioral outcome (mobility: time spent in retail and recreation)
Time resolution of raw outcome	daily
Computed outcome	none
Method to obtain the computed outcome	– not applicable –
Data source	mobility data from corporate organizations (Google)
Data availability	data made available by the authors
**Intervention**	
Terminology for interventions	interventions
Terminology for the specific type of non-pharmaceutical interventions	non-pharmaceutical
Exposure types	multiple separate interventions (retail outlets closure (e.g. restaurants), contact restriction (movement in public places restricted to just two people), stay-at-home order, non-essential services closure (e.g. trade shows), educational facilities closure (e.g. school and university closure), initial business closure (e.g. restaurants), international border closure)
Types of single interventions	workplace closure, venue closure, bans of small gatherings, stay-at-home order, international travel restrictions
Coding of interventions	necessary
Source of intervention data	use of externally coded data (Insitute for Health Metrics and Evaluation, ACAPS Government Measures Dataset)
Availability of data on exposure	coded data made available by the authors
**Methodological approach**	
Empirical approach	parametric
Use of exposure variation	both variation over time and between populations

– continued on next page –

Method	non-mechanistic model (generalized linear model)
Code availability	publicly available
**Effectiveness assessment**	
Reporting of intervention effectiveness	quantification of change in outcome values
Measure of effectiveness	mobility: time spent in retail and recreation
Interpretation of results	associative
Reporting of uncertainty	yes
Sensitivity analysis	none
Subgroup assessment	none

Aravindakshan et al. 2020 (Analysis 2, mobility as exposure)

**Study information**	
Author	Aravindakshan A, Boehnke J, Gholami E, Nayak A
Title	Preparing for a Future COVID-19 Wave: Insights and Limitations from a Data-Driven Evaluation of Non-Pharmaceutical Interventions in Germany
Year	2020
Journal	Scientific Reports
DOI	10.1038/s41598-020-76244-6
Date received	01.07.2020
Date accepted	26.10.2020
**Study setting**	
Number of populations included	multiple
Level of populations included	both national and subnational (Germanys states)
Geographic areas covered	Europe

– continued on next page –

Number of countries covered	single country (Germany)
Study period	start and end date span first epidemic wave
**Outcome**	
Raw outcome	epidemiological population-level outcome (cases)
Time resolution of raw outcome	daily
Computed outcome	none
Method to obtain the computed outcome	– not applicable –
Data source	data from (sub)national authorities
Data availability	data made available by the authors
**Intervention**	
Terminology for interventions	interventions
Terminology for the specific type of non-pharmaceutical interventions	non-pharmaceutical
Exposure types	mobility (time spent in retail and recreation, predicted from regression model on NPIs)
Types of single interventions	– not applicable –
Coding of interventions	– not applicable –
Source of intervention data	mobility data by corporate organizations (Google)
Availability of data on exposure	data access via source
**Methodological approach**	
Empirical approach	counterfactual
Use of exposure variation	only variation over time for multiple populations
Method	mechanistic model (compartmental single-population transmission model)
Code availability	publicly available
**Effectiveness assessment**	

– continued on next page –

Reporting of intervention effectiveness	quantification of change in outcome values
Measure of effectiveness	cases
Interpretation of results	associative
Reporting of uncertainty	yes
Sensitivity analysis	none
Subgroup assessment	none

Arshed et al. 2020

**Study information**	
Author	Arshed N, Meo MS, Farooq F
Title	Empirical Assessment of Government Policies and Flattening of the COVID 19 Curve
Year	2020
Journal	Journal of Public Affairs
DOI	10.1002/pa.2333
Date received	2020-06-22
Date accepted	2020-07-26
**Study setting**	
Number of populations included	multiple
Level of populations included	national (190 countries worldwide)
Geographic areas covered	Europe, Middle East and Africa, Asia, Oceania, Central and South America, North America
Number of countries covered	multiple countries

– continued on next page –

Study period	same end date for several populations with diverse epidemic trajectories
**Outcome**	
Raw outcome	epidemiological population-level outcome (cases)
Time resolution of raw outcome	daily
Computed outcome	summary statistic (number of days until flattening of the curve)
Method to obtain the computed outcome	simple computation
Data source	data from publicly available cross-country selections (JHU)
Data availability	data access via source
**Intervention**	
Terminology for interventions	policies
Terminology for the specific type of non-pharmaceutical interventions	government
Exposure types	multiple separate interventions (school closure, bans of large gatherings, workplace closure, stay-at-home order, international travel restrictions, internal movement restrictions, public transport closure)
Types of single interventions	school closure, bans of large gatherings, workplace closure, stay-at-home order, international travel restrictions, internal movement restrictions, public transport closure
Coding of interventions	necessary
Source of intervention data	use of externally coded data (OxCGRT)
Availability of data on exposure	access to externally coded data via source
**Methodological approach**	
Empirical approach	parametric
Use of exposure variation	only variation between populations
Method	non-mechanistic model (other: generalized additive model)

– continued on next page –

Code availability	none
**Effectiveness assessment**	
Reporting of intervention effectiveness	quantification of change in outcome values
Measure of effectiveness	number of days until flattening of the curve
Interpretation of results	implicitly causal
Reporting of uncertainty	yes
Sensitivity analysis	none
Subgroup assessment	none

Asongu et al. 2020

**Study information**	
Author	Asongu SA, Diop S, Nnanna J
Title	The Geography of the Effectiveness and Consequences of Covid-19 Measures: Global Evidence
Year	2020
Journal	Journal of Public Affairs
DOI	10.1002/pa.2483
Date received	2020-07-21
Date accepted	2020-09-11
**Study setting**	
Number of populations included	multiple
Level of populations included	national (186 countries worldwide)
Geographic areas covered	Europe, Middle East and Africa, Asia, Oceania, Central and South America, North America

– continued on next page –

Number of countries covered	multiple countries
Study period	same end date for several populations with diverse epidemic trajectories
**Outcome**	
Raw outcome	epidemiological population-level outcome (cases)
Time resolution of raw outcome	daily
Computed outcome	summary statistic (peak reached or not)
Method to obtain the computed outcome	other (visual inspection)
Data source	data from publicly available cross-country selections (ECDC)
Data availability	data access via source
**Intervention**	
Terminology for interventions	measures
Terminology for the specific type of non-pharmaceutical interventions	prevention, mitigation
Exposure types	categories of interventions (lockdown, movement restrictions, social distancing, governance/economic measures, public health measures (further detailed in manuscript))
Types of single interventions	– not applicable –
Coding of interventions	necessary
Source of intervention data	use of externally coded data (ACAPS Government Measures Dataset)
Availability of data on exposure	access to externally coded data via source
**Methodological approach**	
Empirical approach	descriptive
Use of exposure variation	only variation between populations
Method	comparison of populations
Code availability	none

– continued on next page –

**Effectiveness assessment**	
Reporting of intervention effectiveness	quantification of change in outcome values
Measure of effectiveness	peak reached or not
Interpretation of results	implicitly causal
Reporting of uncertainty	yes
Sensitivity analysis	none
Subgroup assessment	geographic areas (regions)

Auger et al. 2020

**Study information**	
Author	Auger KA, Shah SS, Richardson T, Hartley D, Hall M, Warniment A, Timmons K, Bosse D, Ferris SA, Brady PW, Schondelmeyer AC, Thomson JE
Title	Association between Statewide School Closure and COVID-19 Incidence and Mortality in the US
Year	2020
Journal	JAMA
DOI	10.1001/jama.2020.14348
Date received	– could not be evaluated –
Date accepted	2020-07-17
**Study setting**	
Number of populations included	multiple
Level of populations included	both national and subnational (50 US states)
Geographic areas covered	North America

– continued on next page –

Number of countries covered	single country (US)
Study period	same end date for several populations with diverse epidemic trajectories
**Outcome**	
Raw outcome	epidemiological population-level outcome (cases, deaths)
Time resolution of raw outcome	daily
Computed outcome	none
Method to obtain the computed outcome	– not applicable –
Data source	data from publicly available cross-country selections (JHU)
Data availability	data access via source
**Intervention**	
Terminology for interventions	interventions
Terminology for the specific type of non-pharmaceutical interventions	non-pharmaceutical
Exposure types	single individual intervention (school closure (other controls: stay-at-home or shelter-in-place order, nonessential business closure, restaurant and bar closure, and prohibition of gatherings with more than 10 people))
Types of single interventions	school closure
Coding of interventions	– not applicable –
Source of intervention data	coding done by authors
Availability of data on exposure	coded data made available by the authors
**Methodological approach**	
Empirical approach	parametric
Use of exposure variation	both variation over time and between populations
Method	non-mechanistic model (generalized linear model)
Code availability	none

– continued on next page –

**Effectiveness assessment**	
Reporting of intervention effectiveness	quantification of change in outcome values
Measure of effectiveness	cases, deaths
Interpretation of results	associative
Reporting of uncertainty	yes
Sensitivity analysis	epidemiological parameters varied
Subgroup assessment	epidemiological indicators (pre-intervention epidemic trend), socioeconomic indicators (population density, age, health care occupation), public health response (subject to other measures)

Badr et al. 2020 (Analysis 1)

**Study information**	
Author	Badr HS, Du H, Marshall M, Dong E, Squire MM, Gardner LM
Title	Association between Mobility Patterns and COVID-19 Transmission in the USA: A Mathematical Modelling Study
Year	2020
Journal	The Lancet Infectious Diseases
DOI	10.1016/s1473-3099(20)30553-3
Date received	– could not be evaluated –
Date accepted	2020-07-01 (published)
**Study setting**	
Number of populations included	multiple
Level of populations included	subnational (25 US counties)

– continued on next page –

Geographic areas covered	North America
Number of countries covered	single country (US)
Study period	start and end date span first epidemic wave: behavioral
**Outcome**	
Raw outcome	behavioral outcome (mobility: number of trips between and within counties, relative to baseline)
Time resolution of raw outcome	daily
Computed outcome	none
Method to obtain the computed outcome	– not applicable –
Data source	mobility data from corporate organizations (Teralytics)
Data availability	data not accessible
**Intervention**	
Terminology for interventions	measures
Terminology for the specific type of non-pharmaceutical interventions	social distancing
Exposure types	one single intervention (stay-at-home order (state- and county-level))
Types of single interventions	stay-at-home order
Coding of interventions	necessary
Source of intervention data	coding done by authors
Availability of data on exposure	coded data made available by the authors
**Methodological approach**	
Empirical approach	descriptive
Use of exposure variation	only variation over time for multiple populations
Method	description of change over time (description of time course)
Code availability	none

– continued on next page –

**Effectiveness assessment**	
Reporting of intervention effectiveness	qualitative statement
Measure of effectiveness	mobility: smoothed mobility ratio
Interpretation of results	associative
Reporting of uncertainty	– not applicable –
Sensitivity analysis	none
Subgroup assessment	none

**Badr et al. 2020 (Analysis 2, mobility as exposure)**	
**Study information**	
Author	Badr HS, Du H, Marshall M, Dong E, Squire MM, Gardner LM
Title	Association between Mobility Patterns and COVID-19 Transmission in the USA: A Mathematical Modelling Study
Year	2020
Journal	The Lancet Infectious Diseases
DOI	10.1016/s1473-3099(20)30553-3
Date received	– could not be evaluated –
Date accepted	01.07.2020 (published)
**Study setting**	
Number of populations included	multiple
Level of populations included	subnational (25 US counties)
Geographic areas covered	North America
Number of countries covered	single country (US)
Study period	same end date for several populations with diverse epidemic trajectories

– continued on next page –

**Outcome**	
Raw outcome	epidemiological population-level outcome (cases)
Time resolution of raw outcome	daily
Computed outcome	measure of epidemic trend (growth rate ratio)
Method to obtain the computed outcome	simple computation
Data source	data from publicly available cross-country selections (JHU)
Data availability	data access via source
**Intervention**	
Terminology for interventions	measures
Terminology for the specific type of non-pharmaceutical interventions	social distancing
Exposure types	mobility (number of trips between and within counties, relative to baseline)
Types of single interventions	– not applicable –
Coding of interventions	– not applicable –
Source of intervention data	mobility data by corporate organizations (Teralytics)
Availability of data on exposure	data not accessible
**Methodological approach**	
Empirical approach	parametric
Use of exposure variation	both variation over time and between populations
Method	non-mechanistic model (generalized linear model, correlation)
Code availability	none
**Effectiveness assessment**	
Reporting of intervention effectiveness	quantification of change in outcome values
Measure of effectiveness	growth rate ratio

– continued on next page –

Interpretation of results	associative
Reporting of uncertainty	yes
Sensitivity analysis	different or modified outcome used (mobility aggregated by state)
Subgroup assessment	none

Baker et al. 2020 (Analysis 1)

**Study information**	
Author	Baker RE, Park SW, Yang W, Vecchi GA, Metcalf CJ, Grenfell BT
Title	The Impact of COVID-19 Nonpharmaceutical Interventions on the Future Dynamics of Endemic Infections
Year	2020
Journal	Proceedings of the National Academy of Sciences
DOI	10.1073/pnas.2013182117
Date received	2020-06-24
Date accepted	2020-10-13
**Study setting**	
Number of populations included	multiple
Level of populations included	subnational (4 US states: Florinda, Minnesota, Oregan & SW Washingston, Texas)
Geographic areas covered	North America
Number of countries covered	single country (US)
Study period	end date in growth phase of wave
**Outcome**	

– continued on next page –

Raw outcome	epidemiological population-level outcome (surrogate disease: test positivity rate for RSV and influenza)
Time resolution of raw outcome	weekly
Computed outcome	none
Method to obtain the computed outcome	– not applicable –
Data source	data from (sub)national authorities
Data availability	data made available by the authors
**Intervention**	
Terminology for interventions	interventions
Terminology for the specific type of non-pharmaceutical interventions	non-pharmaceutical
Exposure types	all interventions together
Types of single interventions	– not applicable –
Coding of interventions	– not applicable –
Source of intervention data	– not applicable –
Availability of data on exposure	– not applicable –
**Methodological approach**	
Empirical approach	descriptive
Use of exposure variation	only variation over time for multiple populations
Method	description of change over time (comparison of time periods)
Code availability	publicly available
**Effectiveness assessment**	
Reporting of intervention effectiveness	qualitative statement
Measure of effectiveness	test positivity rate: RSV & influenza

– continued on next page –

Interpretation of results	associative
Reporting of uncertainty	– not applicable –
Sensitivity analysis	none
Subgroup assessment	none

Baker et al. 2020 (Analysis 2)

**Study information**	
Author	Baker RE, Park SW, Yang W, Vecchi GA, Metcalf CJ, Grenfell BT
Title	The Impact of COVID-19 Nonpharmaceutical Interventions on the Future Dynamics of Endemic Infections
Year	2020
Journal	Proceedings of the National Academy of Sciences
DOI	10.1073/pnas.2013182117
Date received	2020-06-24
Date accepted	2020-10-13
**Study setting**	
Number of populations included	multiple
Level of populations included	both national and subnational (majority of US states)
Geographic areas covered	North America
Number of countries covered	single country (US)
Study period	same end date for several populations with diverse epidemic trajectories
**Outcome**	
Raw outcome	epidemiological population-level outcome (surrogate disease: test positivity rate for influenza)
Time resolution of raw outcome	weekly

– continued on next page –

Computed outcome	none
Method to obtain the computed outcome	– not applicable –
Data source	data from (sub)national authorities
Data availability	data made available by the authors
**Intervention**	
Terminology for interventions	interventions
Terminology for the specific type of non-pharmaceutical interventions	non-pharmaceutical
Exposure types	all interventions together
Types of single interventions	– not applicable –
Coding of interventions	– not applicable –
Source of intervention data	– not applicable –
Availability of data on exposure	– not applicable –
**Methodological approach**	
Empirical approach	descriptive
Use of exposure variation	only variation over time for multiple populations
Method	description of change over time (comparison of time periods)
Code availability	publicly available
**Effectiveness assessment**	
Reporting of intervention effectiveness	quantification of change in outcome values
Measure of effectiveness	test positivity rate: influenza
Interpretation of results	associative
Reporting of uncertainty	no

– continued on next page –

Sensitivity analysis	none
Subgroup assessment	none

**Banerjee & Nayak 2020**	
**Study information**	
Author	Banerjee T, Nayak A
Title	U.S. County Level Analysis to Determine If Social Distancing Slowed the Spread of COVID-19
Year	2020
Journal	Revista Panamericana de Salud Pública
DOI	10.26633/rpsp.2020.90
Date received	2020-04-23
Date accepted	2020-07-03
**Study setting**	
Number of populations included	multiple
Level of populations included	both national and subnational (US and all counties)
Geographic areas covered	North America
Number of countries covered	single country (US)
Study period	end date in growth phase of wave
**Outcome**	
Raw outcome	epidemiological population-level outcome (cases)
Time resolution of raw outcome	daily
Computed outcome	none

– continued on next page –

Method to obtain the computed outcome	– not applicable –
Data source	data from (sub)national authorities
Data availability	data access via source
**Intervention**	
Terminology for interventions	interventions
Terminology for the specific type of non-pharmaceutical interventions	non-pharmaceutical
Exposure types	start of interventions (first NPI out of potentially several NPIs taken in each country)
Types of single interventions	– not applicable –
Coding of interventions	necessary
Source of intervention data	use of externally coded data (New York Times, Keystone)
Availability of data on exposure	access to externally coded data via source
**Methodological approach**	
Empirical approach	parametric
Use of exposure variation	both variation over time and between populations
Method	non-mechanistic model (generalized linear model)
Code availability	none
**Effectiveness assessment**	
Reporting of intervention effectiveness	quantification of change in outcome values
Measure of effectiveness	cases
Interpretation of results	implicitly causal
Reporting of uncertainty	yes

– continued on next page –

Sensitivity analysis	model specification varied
Subgroup assessment	none

**Bemanian et al. 2020**	
**Study information**	
Author	Bemanian A, Ahn KW, O'brien M, Rausch DJ, Weston B, Beyer KMM
Title	Investigating the Trajectory of the COVID-19 Outbreak in Milwaukee County and Projected Effects of Relaxed Distancing
Year	2020
Journal	Wisconsin Medical Journal
DOI	– not applicable –
Date received	– could not be evaluated –
Date accepted	2020-06-01 (published)
**Study setting**	
Number of populations included	single
Level of populations included	subnational (Milwaukee county (Wisconsin, USA))
Geographic areas covered	North America
Number of countries covered	single country (US)
Study period	end date in growth phase of wave
**Outcome**	
Raw outcome	epidemiological population-level outcome (cases)
Time resolution of raw outcome	daily
Computed outcome	epidemiological parameter (reproduction number)

– continued on next page –

Method to obtain the computed outcome	statistical estimation of reproduction number (Cori et al.)
Data source	data from (sub)national authorities
Data availability	data access via source
**Intervention**	
Terminology for interventions	measures
Terminology for the specific type of non-pharmaceutical interventions	physical distancing
Exposure types	multiple combinations of interventions (initial social distancing measures (school closure, gathering bans), stay-at-home order)
Types of single interventions	– not applicable –
Coding of interventions	not necessary
Source of intervention data	– could not be evaluated –
Availability of data on exposure	raw data documented in the manuscript
**Methodological approach**	
Empirical approach	descriptive
Use of exposure variation	only variation over time for a single population
Method	description of change over time (description of time course)
Code availability	none
**Effectiveness assessment**	
Reporting of intervention effectiveness	qualitative statement
Measure of effectiveness	reproduction number
Interpretation of results	associative
Reporting of uncertainty	– not applicable –

– continued on next page –

Sensitivity analysis	none
Subgroup assessment	none

**Bennett 2021**	
**Study information**	
Author	Bennett M
Title	All Things Equal? Heterogeneity in Policy Effectiveness against COVID-19 Spread in Chile
Year	2021
Journal	World Development
DOI	10.1016/j.worlddev.2020.105208
Date received	– could not be evaluated –
Date accepted	2020-09-15
**Study setting**	
Number of populations included	multiple
Level of populations included	both national and subnational (municipalities in Santiago and Chile where COVID was spread, number unclear)
Geographic areas covered	Central and South America
Number of countries covered	single country (Chile)
Study period	end date in growth phase of wave
**Outcome**	
Raw outcome	epidemiological population-level outcome (cases), behavioral outcome (mobility: transit station mobility index & subway validations)
Time resolution of raw outcome	daily
Computed outcome	none

– continued on next page –

Method to obtain the computed outcome	– not applicable –
Data source	data from (sub)national authorities (health authority: cases, Ministry of Transportation: subway validations), mobility data from corporate organizations (Google: transit station mobility index)
Data availability	data access via source
**Intervention**	
Terminology for interventions	– not applicable –
Terminology for the specific type of non-pharmaceutical interventions	lockdown
Exposure types	one combination of interventions (lockdown)
Types of single interventions	– not applicable –
Coding of interventions	necessary
Source of intervention data	– could not be evaluated –
Availability of data on exposure	coded data made available by the authors
**Methodological approach**	
Empirical approach	counterfactual
Use of exposure variation	both variation over time and between populations
Method	synthetic controls
Code availability	none
**Effectiveness assessment**	
Reporting of intervention effectiveness	quantification of change in outcome values
Measure of effectiveness	cases, mobility: transit station mobility index, mobility: subway validations
Interpretation of results	explicitly causal
Reporting of uncertainty	yes

– continued on next page –

Sensitivity analysis	same analysis with (sub)population(s) excluded
Subgroup assessment	socioeconomic indicators (income)

**Bo et al. 2021**	
**Study information**	
Author	Bo Y, Guo C, Lin C, Zeng Y, Li HB, Zhang Y, Hossain MS, Chan JW, Yeung DW, Kwok KO, Wong SY, Lau AK, Lao XQ
Title	Effectiveness of Non-Pharmaceutical Interventions on COVID-19 Transmission in 190 Countries from 23 January to 13 April 2020
Year	2021
Journal	International Journal of Infectious Diseases
DOI	10.1016/j.ijid.2020.10.066
Date received	2020-07-27
Date accepted	2020-10-21
**Study setting**	
Number of populations included	multiple
Level of populations included	both national and subnational (235 cities from 10 countries + 180 countries worldwide)
Geographic areas covered	Europe, Middle East and Africa, Asia, Oceania, Central and South America, North America
Number of countries covered	multiple countries
Study period	end date could not be evaluated
**Outcome**	
Raw outcome	epidemiological population-level outcome (cases)
Time resolution of raw outcome	daily

– continued on next page –

Computed outcome	epidemiological parameter (reproduction number)
Method to obtain the computed outcome	statistical estimation of reproduction number (Cori et al.)
Data source	data from publicly available cross-country selections (JHU: cases), mobility data from corporate organizations (Wind financial database: cases)
Data availability	data access via source
**Intervention**	
Terminology for interventions	interventions
Terminology for the specific type of non-pharmaceutical interventions	non-pharmaceutical
Exposure types	start of interventions (the first day an NPI was implemented within a group (NPI groups: travel restriction, social distancing, mask wearing mandate, isolation or quarantine))
Types of single interventions	– not applicable –
Coding of interventions	necessary
Source of intervention data	coding done by authors
Availability of data on exposure	coded data not available
**Methodological approach**	
Empirical approach	parametric
Use of exposure variation	both variation over time and between populations
Method	non-mechanistic model (generalized linear model)
Code availability	none
**Effectiveness assessment**	
Reporting of intervention effectiveness	quantification of change in outcome values
Measure of effectiveness	reproduction number
Interpretation of results	associative

– continued on next page –

Reporting of uncertainty	yes
Sensitivity analysis	epidemiological parameters varied, model specification varied, same analysis with (sub)population(s) excluded
Subgroup assessment	geographic areas (regions), socioeconomic indicators (population density, age, health index: global health security index)

**Bönisch et al. 2020 (Analysis 1)**	
**Study information**	
Author	Bönisch S, Wegscheider K, Krause L, Sehner S, Wiegel S, Zapf A, Moser S, Becher H
Title	Effects of Coronavirus Disease (COVID-19) Related Contact Restrictions in Germany, March to May 2020, on the Mobility and Relation to Infection Patterns
Year	2020
Journal	Frontiers in Public Health
DOI	10.3389/fpubh.2020.568287
Date received	2020-05-31
Date accepted	2020-09-18
**Study setting**	
Number of populations included	single
Level of populations included	national (Germany)
Geographic areas covered	Europe
Number of countries covered	single country (Germany)
Study period	start and end date span first epidemic wave: behavioral

– continued on next page –

**Outcome**	
Raw outcome	behavioral outcome (mobility: median daily distance)
Time resolution of raw outcome	daily
Computed outcome	none
Method to obtain the computed outcome	– not applicable –
Data source	mobility data from corporate organizations (GapFish)
Data availability	data not accessible
**Intervention**	
Terminology for interventions	– not applicable –
Terminology for the specific type of non-pharmaceutical interventions	contact restrictions
Exposure types	one combination of interventions (lockdown period from 23.03 to 05.04.2020 following the closure of schools and non-essential public services, TV adress by Angela Merkel, contact restrictions)
Types of single interventions	– not applicable –
Coding of interventions	not necessary
Source of intervention data	government or news websites
Availability of data on exposure	raw data documented in the manuscript
**Methodological approach**	
Empirical approach	counterfactual
Use of exposure variation	only variation over time for a single population
Method	non-mechanistic model (time series model)
Code availability	none
**Effectiveness assessment**	

– continued on next page –

Reporting of intervention effectiveness	quantification of change in outcome values
Measure of effectiveness	mobility: median daily distance
Interpretation of results	associative
Reporting of uncertainty	yes
Sensitivity analysis	none
Subgroup assessment	socioeconomic indicators (age, gender)

**Bönisch et al. 2020 (Analysis 2, mobility as exposure)**	
**Study information**	
Author	Bönisch S, Wegscheider K, Krause L, Sehner S, Wiegel S, Zapf A, Moser S, Becher H
Title	Effects of Coronavirus Disease (COVID-19) Related Contact Restrictions in Germany, March to May 2020, on the Mobility and Relation to Infection Patterns
Year	2020
Journal	Frontiers in Public Health
DOI	10.3389/fpubh.2020.568287
Date received	31.05.2020
Date accepted	18.09.2020
**Study setting**	
Number of populations included	single
Level of populations included	national (Germany)
Geographic areas covered	Europe
Number of countries covered	single country (Germany)

– continued on next page –

Study period	start and end date span first epidemic wave
**Outcome**	
Raw outcome	epidemiological population-level outcome (cases, reproduction number)
Time resolution of raw outcome	daily
Computed outcome	none
Method to obtain the computed outcome	– not applicable –
Data source	data from (sub)national authorities
Data availability	data access via source
**Intervention**	
Terminology for interventions	– not applicable –
Terminology for the specific type of non-pharmaceutical interventions	contact restrictions
Exposure types	mobility (relative reduction in median daily distance in the study period)
Types of single interventions	– not applicable –
Coding of interventions	– not applicable –
Source of intervention data	mobility data by corporate organizations (GapFish)
Availability of data on exposure	data not accessible
**Methodological approach**	
Empirical approach	descriptive
Use of exposure variation	only variation over time for a single population
Method	description of change over time (description of time course)
Code availability	none
**Effectiveness assessment**	
Reporting of intervention effectiveness	qualitative statement

– continued on next page –

**Bönisch et al. 2020 (Analysis 2, mobility as exposure)** – continued from previous page	
Measure of effectiveness	cases, reproduction number
Interpretation of results	associative
Reporting of uncertainty	– not applicable –
Sensitivity analysis	none
Subgroup assessment	none

**Braithwaite et al. 2020**	
**Study information**	
Author	Braithwaite J, Tran Y, Ellis LA, Westbrook J
Title	The 40 Health Systems, COVID-19 (40HS, C-19) Study
Year	2020
Journal	International Journal for Quality in Health Care
DOI	10.1093/intqhc/mzaa113
Date received	2020-06-24
Date accepted	2020-09-08
**Study setting**	
Number of populations included	multiple
Level of populations included	national (40 mostly OECD countries)
Geographic areas covered	Europe, Middle East and Africa, Asia, Oceania, Central and South America, North America
Number of countries covered	multiple countries
Study period	same end date for several populations with diverse epidemic trajectories
**Outcome**	
Raw outcome	epidemiological population-level outcome (cases, deaths)

– continued on next page –

Time resolution of raw outcome	daily
Computed outcome	none
Method to obtain the computed outcome	– not applicable –
Data source	data from publicly available cross-country selections (OurWorldInData)
Data availability	data access via source
**Intervention**	
Terminology for interventions	measures
Terminology for the specific type of non-pharmaceutical interventions	public health
Exposure types	stringency index (OxCGRT Stringency Index (at 3 time points))
Types of single interventions	– not applicable –
Coding of interventions	necessary
Source of intervention data	use of externally coded data (OxCGRT)
Availability of data on exposure	access to externally coded data via source
**Methodological approach**	
Empirical approach	descriptive
Use of exposure variation	only variation between populations
Method	comparison of populations
Code availability	none
**Effectiveness assessment**	
Reporting of intervention effectiveness	quantification of change in outcome values
Measure of effectiveness	cases, deaths
Interpretation of results	associative
Reporting of uncertainty	yes

– continued on next page –

Sensitivity analysis	none
Subgroup assessment	none

**Brauner et al. 2021**	
**Study information**	
Author	Brauner JM, Mindermann S, Sharma M, Johnston D, Salvatier J, Gavenčiak T, Stephenson AB, Leech G, Altman G, Mikulik V, Norman AJ, Monrad JT, Besiroglu T, Ge H, Hartwick MA, Teh YW, Chindelevitch L, Gal Y, Kulveit J
Title	Inferring the Effectiveness of Government Interventions against COVID-19
Year	2021
Journal	Science
DOI	10.1126/science.abd9338
Date received	2020-07-21
Date accepted	2020-12-08
**Study setting**	
Number of populations included	multiple
Level of populations included	national (34 European, 7 non-European countries)
Geographic areas covered	Asia, Central and South America, Europe, Middle East and Africa, Oceania
Number of countries covered	multiple countries
Study period	start and end date span first epidemic wave
**Outcome**	
Raw outcome	epidemiological population-level outcome (cases, deaths)
Time resolution of raw outcome	daily
Computed outcome	none

– continued on next page –

Method to obtain the computed outcome	– not applicable –
Data source	data from publicly available cross-country selections (JHU)
Data availability	data made available by the authors
**Intervention**	
Terminology for interventions	interventions
Terminology for the specific type of non-pharmaceutical interventions	non-pharmaceutical
Exposure types	multiple separate interventions (bans of small gatherings, bans of large gatherings, venue closure, workplace closure, stay-at-home order, school closure)
Types of single interventions	bans of small gatherings, bans of large gatherings, venue closure, workplace closure, stay-at-home order, school closure
Coding of interventions	necessary
Source of intervention data	coding done by authors
Availability of data on exposure	coded data made available by the authors
**Methodological approach**	
Empirical approach	parametric
Use of exposure variation	both variation over time and between populations
Method	mechanistic model (semi-mechanistic Bayesian transmission model)
Latent variable functionally linked to intervention	reproduction number
Code availability	publicly available
**Effectiveness assessment**	
Reporting of intervention effectiveness	quantification of change in outcome values
Measure of effectiveness	reproduction number

– continued on next page –

Interpretation of results	implicitly causal
Reporting of uncertainty	yes
Sensitivity analysis	different or modified outcome used (number of confirmed cases adjusted using time-varying ascertainment rate estimated by study), different coding of interventions used (specific NPIs left out), model specification varied, start or end date of study period varied, epidemiological parameters varied, same analysis with (sub)population(s) excluded
Subgroup assessment	none

Candido et al. 2020 (Analysis 1)

**Study information**	
Author	Candido DS, Claro IM, de Jesus JG, Souza WM, Moreira FR, Dellicour S, Mellan TA, du Plessis L, Pereira RH, Sales FC, Manuli ER, Thézé J, Almeida L, Menezes MT, Voloch CM, Fumagalli MJ, Coletti TM, da Silva CA, Ramundo MS, Amorim MR, Hoeltgebaum HH, Mishra S, Gill MS, Carvalho LM, Buss LF, Prete CA, Ashworth J, Nakaya HI, Peixoto PS, Brady OJ, Nicholls SM, Tanuri A, Rossi ÁD, Braga CK, Gerber AL, de C. Guimarães AP, Gaburo N, Alencar CS, Ferreira AC, Lima CX, Levi JE, Granato C, Ferreira GM, Francisco RS, Granja F, Garcia MT, Moretti ML, Perroud MW, Castiñeiras TM, Lazari CS, Hill SC, de Souza Santos AA, Simeoni CL, Forato J, Sposito AC, Schreiber AZ, Santos MN, de Sá CZ, Souza RP, Resende-Moreira LC, Teixeira MM, Hubner J, Leme PA, Moreira RG, Nogueira ML, Ferguson NM, Costa SF, Proenca-Modena JL, Vasconcelos AT, Bhatt S, Lemey P, Wu CH, Rambaut A, Loman NJ, Aguiar RS, Pybus OG, Sabino EC, And NR
Title	Evolution and Epidemic Spread of SARS-CoV-2 in Brazil
Year	2020

– continued on next page –

Journal	Science
DOI	10.1126/science.abd2161
Date received	2020-06-10
Date accepted	2020-07-16
**Study setting**	
Number of populations included	multiple
Level of populations included	both national and subnational (Brazil and its major cities)
Geographic areas covered	Central and South America
Number of countries covered	single country (Brazil)
Study period	end date in growth phase of wave
**Outcome**	
Raw outcome	epidemiological individual-level outcome (genome sequence data), behavioral outcome (mobility: air travel)
Time resolution of raw outcome	daily
Computed outcome	other (virus migration)
Method to obtain the computed outcome	phylodynamic model (phylogeographic analysis)
Data source	data from (sub)national authorities (Civil Aviation Agency: mobility), data collected by authors (viral sequence data)
Data availability	data made available by the authors
**Intervention**	
Terminology for interventions	interventions
Terminology for the specific type of non-pharmaceutical interventions	non-pharmaceutical
Exposure types	single combination of interventions (school closure, retail and service closure, emergency declaration)

– continued on next page –

Types of single interventions	– not applicable –
Coding of interventions	not necessary
Source of intervention data	– could not be evaluated –
Availability of data on exposure	raw data documented in the manuscript
**Methodological approach**	
Empirical approach	descriptive
Use of exposure variation	only variation over time for multiple populations
Method	description of change over time (description of time course)
Code availability	publicly available
**Effectiveness assessment**	
Reporting of intervention effectiveness	qualitative statement
Measure of effectiveness	mobility: air travel, virus migration
Interpretation of results	implicitly causal
Reporting of uncertainty	– not applicable –
Sensitivity analysis	none
Subgroup assessment	none

Candido et al. 2020 (Analysis 2, mobility as exposure)

Study information

– continued on next page –

Author	Candido DS, Claro IM, de Jesus JG, Souza WM, Moreira FR, Dellicour S, Mellan TA, du Plessis L, Pereira RH, Sales FC, Manuli ER, Thézé J, Almeida L, Menezes MT, Voloch CM, Fumagalli MJ, Coletti TM, da Silva CA, Ramundo MS, Amorim MR, Hoeltgebaum HH, Mishra S, Gill MS, Carvalho LM, Buss LF, Prete CA, Ashworth J, Nakaya HI, Peixoto PS, Brady OJ, Nicholls SM, Tanuri A, Rossi ÁD, Braga CK, Gerber AL, de C. Guimarães AP, Gaburo N, Alencar CS, Ferreira AC, Lima CX, Levi JE, Granato C, Ferreira GM, Francisco RS, Granja F, Garcia MT, Moretti ML, Perroud MW, Castiñeiras TM, Lazari CS, Hill SC, de Souza Santos AA, Simeoni CL, Forato J, Sposito AC, Schreiber AZ, Santos MN, de Sá CZ, Souza RP, Resende-Moreira LC, Teixeira MM, Hubner J, Leme PA, Moreira RG, Nogueira ML, Ferguson NM, Costa SF, Proenca-Modena JL, Vasconcelos AT, Bhatt S, Lemey P, Wu CH, Rambaut A, Loman NJ, Aguiar RS, Pybus OG, Sabino EC, And NR
Title	Evolution and Epidemic Spread of SARS-CoV-2 in Brazil
Year	2020
Journal	Science
DOI	10.1126/science.abd2161
Date received	10.06.2020
Date accepted	16.07.2020
**Study setting**	
Number of populations included	multiple
Level of populations included	both national and subnational (Brazil and its major cities)
Geographic areas covered	Central and South America
Number of countries covered	single country (Brazil)
Study period	end date in growth phase of wave
**Outcome**	
Raw outcome	epidemiological population-level outcome (cases, deaths)
Time resolution of raw outcome	daily

– continued on next page –

Computed outcome	none
Method to obtain the computed outcome	– not applicable –
Data source	data from (sub)national authorities
Data availability	data made available by the authors
**Intervention**	
Terminology for interventions	interventions
Terminology for the specific type of non-pharmaceutical interventions	non-pharmaceutical
Exposure types	mobility (time spent in various locations, social isolation index)
Types of single interventions	– not applicable –
Coding of interventions	– not applicable –
Source of intervention data	mobility data by corporate organizations (Google: time spent in various locations, InLoco: social isolation index)
Availability of data on exposure	data access via source
**Methodological approach**	
Empirical approach	parametric
Use of exposure variation	only variation over time for multiple populations
Method	mechanistic model (semi-mechanistic Bayesian transmission model)
Code availability	publicly available
**Effectiveness assessment**	
Reporting of intervention effectiveness	qualitative statement
Measure of effectiveness	reproduction number
Interpretation of results	implicitly causal
Reporting of uncertainty	– not applicable –

– continued on next page –

Sensitivity analysis	none
Subgroup assessment	none

Cao et al. 2020

**Study information**	
Author	Cao Y, Hiyoshi A, Montgomery S
Title	COVID-19 Case-Fatality Rate and Demographic and Socioeconomic Influencers: Worldwide Spatial Regression Analysis Based on Country-Level Data
Year	2020
Journal	BMJ Open
DOI	10.1136/bmjopen-2020-043560
Date received	2020-08-17
Date accepted	2020-09-16
**Study setting**	
Number of populations included	multiple
Level of populations included	national (209 countries/territories worldwide)
Geographic areas covered	Europe, Middle East and Africa, Asia, Oceania, Central and South America, North America
Number of countries covered	multiple countries
Study period	start and end date span first epidemic wave
**Outcome**	
Raw outcome	epidemiological population-level outcome (cases, deaths)
Time resolution of raw outcome	daily
Computed outcome	summary statistic (case-fatality rate)

– continued on next page –

Method to obtain the computed outcome	simple computation
Data source	data from publicly available cross-country selections (OurWorldInData)
Data availability	data access via source
**Intervention**	
Terminology for interventions	measures
Terminology for the specific type of non-pharmaceutical interventions	quarantine
Exposure types	stringency index (OxCGRT Stringency Index)
Types of single interventions	– not applicable –
Coding of interventions	necessary
Source of intervention data	use of externally coded data (OxCGRT)
Availability of data on exposure	access to externally coded data via source
**Methodological approach**	
Empirical approach	parametric
Use of exposure variation	only variation between populations
Method	non-mechanistic model (other: spatial regression model)
Code availability	none
**Effectiveness assessment**	
Reporting of intervention effectiveness	quantification of change in outcome values
Measure of effectiveness	case-fatality rate
Interpretation of results	associative
Reporting of uncertainty	yes

– continued on next page –

Sensitivity analysis	different or modified outcome used (crude cause-specific death rate instead of CFR)
Subgroup assessment	public health response (testing policy), socioeconomic indicators (income)

**Castaneda & Saygili 2020**	
**Study information**	
Author	Castaneda MA, Saygili M
Title	The Effect of Shelter-in-Place Orders on Social Distancing and the Spread of the COVID-19 Pandemic: A Study of Texas
Year	2020
Journal	Frontiers in Public Health
DOI	10.3389/fpubh.2020.596607
Date received	2020-08-27
Date accepted	2020-11-05
**Study setting**	
Number of populations included	multiple
Level of populations included	subnational (Counties in Texas)
Geographic areas covered	North America
Number of countries covered	single country (US)
Study period	end date in growth phase of wave
**Outcome**	
Raw outcome	epidemiological population-level outcome (cases, deaths), behavioral outcome (mobility: share of population sheltering in home)
Time resolution of raw outcome	daily
Computed outcome	measure of epidemic trend (growth rate, growth rate: deaths)

– continued on next page –

Method to obtain the computed outcome	simple computation
Data source	data from public media outlets (New York Times: cases), mobility data from corporate organizations (SafeGraph: mobility)
Data availability	data access via source
**Intervention**	
Terminology for interventions	measures
Terminology for the specific type of non-pharmaceutical interventions	social distancing
Exposure types	multiple separate interventions (emergency declaration, stay-at-home order (county- and statewide))
Types of single interventions	declaration of a state of emergency, stay-at-home order
Coding of interventions	necessary
Source of intervention data	use of externally coded data (National Association of Counties)
Availability of data on exposure	access to externally coded data via source
**Methodological approach**	
Empirical approach	parametric
Use of exposure variation	both variation over time and between populations
Method	non-mechanistic model (generalized linear model)
Code availability	none
**Effectiveness assessment**	
Reporting of intervention effectiveness	quantification of change in outcome values
Measure of effectiveness	growth rate, growth rate: deaths, mobility: share of population sheltering in home
Interpretation of results	implicitly causal
Reporting of uncertainty	yes

– continued on next page –

Sensitivity analysis	none
Subgroup assessment	public health response (subject to other measures)

**Cento et al. 2020**	
**Study information**	
Author	Cento V, Alteri C, Merli M, Ruscio FD, Tartaglione L, Rossotti R, Travi G, Vecchi M, Raimondi A, Nava A, Colagrossi L, Fumagalli R, Ughi N, Epis OM, Fanti D, Beretta A, Galbiati F, Scaglione F, Vismara C, Puoti M, Campisi D, Perno CF
Title	Effectiveness of Infection-Containment Measures on SARS-CoV-2 Seroprevalence and Circulation from May to July 2020, in Milan, Italy
Year	2020
Journal	PLOS ONE
DOI	10.1371/journal.pone.0242765
Date received	2020-08-10
Date accepted	2020-11-09
**Study setting**	
Number of populations included	single
Level of populations included	subnational (Milan (Italy))
Geographic areas covered	Europe
Number of countries covered	single country (Italy)
Study period	start and end date span first epidemic wave
**Outcome**	
Raw outcome	epidemiological population-level outcome (test positivity rate: anti-N IgG seroprevalence)

– continued on next page –

Time resolution of raw outcome	daily
Computed outcome	none
Method to obtain the computed outcome	– not applicable –
Data source	data collected by authors
Data availability	data access via source
**Intervention**	
Terminology for interventions	measures
Terminology for the specific type of non-pharmaceutical interventions	containment
Exposure types	one combination of interventions (lockdown of March 9)
Types of single interventions	– not applicable –
Coding of interventions	not necessary
Source of intervention data	– could not be evaluated –
Availability of data on exposure	raw data documented in the manuscript
**Methodological approach**	
Empirical approach	descriptive
Use of exposure variation	only variation over time for a single population
Method	description of change over time (comparison of time periods)
Code availability	none
**Effectiveness assessment**	
Reporting of intervention effectiveness	comparison of outcome values
Measure of effectiveness	test positivity rate: anti-N IgG seroprevalence
Interpretation of results	associative
Reporting of uncertainty	yes

– continued on next page –

Sensitivity analysis	none
Subgroup assessment	none

**Chakraborty & Ghosh 2020**	
**Study information**	
Author	Chakraborty T, Ghosh I
Title	Real-Time Forecasts and Risk Assessment of Novel Coronavirus (COVID-19) Cases: A Data-Driven Analysis
Year	2020
Journal	Chaos, Solitons & Fractals
DOI	10.1016/j.chaos.2020.109850
Date received	2020-04-10
Date accepted	2020-04-24
**Study setting**	
Number of populations included	multiple
Level of populations included	national (50 countries around the globe)
Geographic areas covered	Europe, Middle East and Africa, Asia, Oceania, Central and South America, North America
Number of countries covered	multiple countries
Study period	same end date for several populations with diverse epidemic trajectories
**Outcome**	
Raw outcome	epidemiological population-level outcome (cases, deaths)
Time resolution of raw outcome	daily
Computed outcome	epidemiological parameter (case-fatality rate)

– continued on next page –

Method to obtain the computed outcome	simple computation
Data source	data from publicly available cross-country selections (Worldometer)
Data availability	data made available by the authors
**Intervention**	
Terminology for interventions	– not applicable –
Terminology for the specific type of non-pharmaceutical interventions	lockdown
Exposure types	one combination of interventions (lockdown with specific date for each country (only days since lockdown are provided))
Types of single interventions	– not applicable –
Coding of interventions	necessary
Source of intervention data	– could not be evaluated –
Availability of data on exposure	coded data made available by the authors
**Methodological approach**	
Empirical approach	parametric
Use of exposure variation	only variation between populations
Method	non-mechanistic model (other: feature importance from machine learning model with lockdown period as predictor)
Code availability	publicly available
**Effectiveness assessment**	
Reporting of intervention effectiveness	quantification of change in outcome values
Measure of effectiveness	case-fatality rate
Interpretation of results	explicitly causal
Reporting of uncertainty	no

– continued on next page –

Sensitivity analysis	none
Subgroup assessment	none

**Chatterjee et al. 2020**	
**Study information**	
Author	Chatterjee S, Sarkar A, Chatterjee S, Karmakar M, Paul R
Title	Studying the Progress of COVID-19 Outbreak in India Using SIRD Model
Year	2020
Journal	Indian Journal of Physics
DOI	10.1007/s12648-020-01766-8
Date received	2020-05-15
Date accepted	2020-06-03
**Study setting**	
Number of populations included	multiple
Level of populations included	both national and subnational (India and 4 states, South Korea, USA, Germany, Spain)
Geographic areas covered	Asia
Number of countries covered	single country (India)
Study period	same end date for several populations with diverse epidemic trajectories
**Outcome**	
Raw outcome	epidemiological population-level outcome (cases, deaths)
Time resolution of raw outcome	daily
Computed outcome	none

– continued on next page –

Method to obtain the computed outcome	– not applicable –
Data source	data from publicly available open-source projects (covid19india.org: India), data from publicly available cross-country selections (Worldometer: other countries)
Data availability	data access via source
**Intervention**	
Terminology for interventions	measures
Terminology for the specific type of non-pharmaceutical interventions	containment
Exposure types	one combination of interventions (Indian National Lockdown)
Types of single interventions	– not applicable –
Coding of interventions	not necessary
Source of intervention data	– could not be evaluated –
Availability of data on exposure	raw data documented in the manuscript
**Methodological approach**	
Empirical approach	parametric
Use of exposure variation	only variation over time for multiple populations
Method	mechanistic model (compartmental single-population transmission model)
Latent variable functionally linked to intervention	transmission rate
Code availability	none
**Effectiveness assessment**	
Reporting of intervention effectiveness	quantification of change in outcome values
Measure of effectiveness	transmission rate, reproduction number
Interpretation of results	implicitly causal

– continued on next page –

Reporting of uncertainty	no
Sensitivity analysis	none
Subgroup assessment	none

**Chaudhry et al. 2020**	
**Study information**	
Author	Chaudhry R, Dranitsaris G, Mubashir T, Bartoszko J, Riazi S
Title	A Country Level Analysis Measuring the Impact of Government Actions, Country Preparedness and Socioeconomic Factors on COVID-19 Mortality and Related Health Outcomes
Year	2020
Journal	EClinicalMedicine
DOI	10.1016/j.eclinm.2020.100464
Date received	2020-05-05
Date accepted	2020-07-02
**Study setting**	
Number of populations included	multiple
Level of populations included	national (50 countries around the globe)
Geographic areas covered	Europe, Middle East and Africa, Asia, Oceania, Central and South America, North America
Number of countries covered	multiple countries
Study period	same end date for several populations with diverse epidemic trajectories
**Outcome**	
Raw outcome	epidemiological population-level outcome (cases, recovered cases, severe cases, deaths)

– continued on next page –

Time resolution of raw outcome	daily
Computed outcome	none
Method to obtain the computed outcome	– not applicable –
Data source	data from publicly available cross-country selections (JHU: other countries, WHO: other countries, Worldometer: other countries), data from (sub)national authorities (US)
Data availability	data access via source
**Intervention**	
Terminology for interventions	policies, measures
Terminology for the specific type of non-pharmaceutical interventions	health policies, containment
Exposure types	multiple separate interventions (travel restrictions (types of travel restrictions, partial border closures, complete border closures) stay-at-home order (partial lockdown, complete lockdown, stay-at-home order))
Types of single interventions	international travel restrictions, stay-at-home order
Coding of interventions	necessary
Source of intervention data	coding done by authors
Availability of data on exposure	coded data not available
**Methodological approach**	
Empirical approach	parametric
Use of exposure variation	only variation between populations
Method	non-mechanistic model (generalized linear model)
Code availability	none
**Effectiveness assessment**	
Reporting of intervention effectiveness	quantification of change in outcome values

– continued on next page –

Measure of effectiveness	cases, recovered, severe cases, deaths
Interpretation of results	associative
Reporting of uncertainty	yes
Sensitivity analysis	none
Subgroup assessment	none

Chaves et al. 2020

**Study information**	
Author	Chaves LF, Hurtado LA, Rojas MR, Friberg MD, Rodríguez RM, Avila-Aguero ML
Title	COVID-19 Basic Reproduction Number and Assessment of Initial Suppression Policies in Costa Rica
Year	2020
Journal	Mathematical Modelling of Natural Phenomena
DOI	10.1051/mmnp/2020019
Date received	2020-04-07
Date accepted	2020-05-07
**Study setting**	
Number of populations included	single
Level of populations included	national (Costa Rica)
Geographic areas covered	Central and South America
Number of countries covered	single country (Costa Rica)
Study period	end date at peak of wave
**Outcome**	

– continued on next page –

Raw outcome	epidemiological population-level outcome (cases)
Time resolution of raw outcome	daily
Computed outcome	epidemiological parameter (reproduction number)
Method to obtain the computed outcome	statistical estimation of reproduction number (Thompson et al.), compartmental transmission model (single-population)
Data source	data from (sub)national authorities
Data availability	data access via source
**Intervention**	
Terminology for interventions	policies
Terminology for the specific type of non-pharmaceutical interventions	suppression
Exposure types	phases of interventions (timeline of measures divided into three phases)
Types of single interventions	– not applicable –
Coding of interventions	not necessary
Source of intervention data	government or news websites
Availability of data on exposure	raw data documented in the manuscript
**Methodological approach**	
Empirical approach	descriptive
Use of exposure variation	only variation over time for a single population
Method	description of change over time (comparison of time periods)
Code availability	none
**Effectiveness assessment**	
Reporting of intervention effectiveness	comparison of outcome values
Measure of effectiveness	reproduction number
Interpretation of results	implicitly causal

– continued on next page –

Reporting of uncertainty	yes
Sensitivity analysis	none
Subgroup assessment	none

**Cheema et al. 2021**	
**Study information**	
Author	Cheema SA, Kifayat T, Rahman AR, Khan U, Zaib A, Khan I, Nisar KS
Title	Is Social Distancing, and Quarantine Effective in Restricting COVID-19 Outbreak? Statistical Evidences from Wuhan, China
Year	2021
Journal	Computers, Materials & Continua
DOI	10.32604/cmc.2020.012096
Date received	2020-06-14
Date accepted	2020-07-07
**Study setting**	
Number of populations included	single
Level of populations included	subnational (Wuhan)
Geographic areas covered	Asia
Number of countries covered	single country (China)
Study period	late start
**Outcome**	
Raw outcome	epidemiological population-level outcome (cases, deaths)
Time resolution of raw outcome	daily
Computed outcome	none

– continued on next page –

Method to obtain the computed outcome	– not applicable –
Data source	data from (sub)national authorities
Data availability	data access via source
**Intervention**	
Terminology for interventions	measures, policies
Terminology for the specific type of non-pharmaceutical interventions	social distancing
Exposure types	one combination of interventions (Wuhan City Lockdown)
Types of single interventions	– not applicable –
Coding of interventions	not necessary
Source of intervention data	– could not be evaluated –
Availability of data on exposure	raw data documented in the manuscript
**Methodological approach**	
Empirical approach	descriptive
Use of exposure variation	only variation over time for a single population
Method	description of change over time (comparison of time periods)
Code availability	none
**Effectiveness assessment**	
Reporting of intervention effectiveness	quantification of change in outcome values
Measure of effectiveness	cases, deaths
Interpretation of results	associative
Reporting of uncertainty	yes
Sensitivity analysis	none
Subgroup assessment	none

Chen et al. 2020a	
**Study information**	
Author	Chen SL, Yen AM, Lai CC, Hsu CY, Chan CC, Chen TH
Title	An Index for Lifting Social Distancing during the COVID-19 Pandemic: Algorithm Recommendation for Lifting Social Distancing
Year	2020
Journal	Journal of Medical Internet Research
DOI	10.2196/22469
Date received	2020-07-13
Date accepted	2020-09-04
**Study setting**	
Number of populations included	multiple
Level of populations included	national (186 countries worldwide)
Geographic areas covered	Europe, Middle East and Africa, Asia, Oceania, Central and South America, North America
Number of countries covered	multiple countries
Study period	same end date for several populations with diverse epidemic trajectories
**Outcome**	
Raw outcome	epidemiological population-level outcome (cases, deaths, recovered cases)
Time resolution of raw outcome	daily
Computed outcome	other (lifting social distancing index)
Method to obtain the computed outcome	simple computation
Data source	data from publicly available cross-country selections (JHU)
Data availability	data access via source

– continued on next page –

**Intervention**	
Terminology for interventions	measures
Terminology for the specific type of non-pharmaceutical interventions	social distancing
Exposure types	all interventions together
Types of single interventions	– not applicable –
Coding of interventions	– not applicable –
Source of intervention data	– not applicable –
Availability of data on exposure	– not applicable –
**Methodological approach**	
Empirical approach	descriptive
Use of exposure variation	only variation over time for multiple populations
Method	description of change over time (description of time course)
Code availability	none
**Effectiveness assessment**	
Reporting of intervention effectiveness	qualitative statement
Measure of effectiveness	lifting social distancing index
Interpretation of results	associative
Reporting of uncertainty	– not applicable –
Sensitivity analysis	none
Subgroup assessment	none

Chen et al. 2020b	
**Study information**	
Author	Chen S, Chen Q, Yang W, Xue L, Liu Y, Yang J, Wang C, Bärnighausen T
Title	Buying Time for an Effective Epidemic Response: The Impact of a Public Holiday for Outbreak Control on COVID-19 Epidemic Spread
Year	2020
Journal	Engineering
DOI	10.1016/j.eng.2020.07.018
Date received	2020-05-07
Date accepted	2020-07-13
**Study setting**	
Number of populations included	multiple
Level of populations included	both national and subnational (China Hubei province and other provinces)
Geographic areas covered	Asia
Number of countries covered	single country (China)
Study period	end date in growth phase of wave
**Outcome**	
Raw outcome	epidemiological population-level outcome (cases, deaths, recovered cases)
Time resolution of raw outcome	daily
Computed outcome	none
Method to obtain the computed outcome	– not applicable –
Data source	data from (sub)national authorities
Data availability	data access via source
**Intervention**	
Terminology for interventions	measures

– continued on next page –

Terminology for the specific type of non-pharmaceutical interventions	control
Exposure types	one combination of interventions (Wuhan City Lockdown)
Types of single interventions	– not applicable –
Coding of interventions	not necessary
Source of intervention data	government or news websites
Availability of data on exposure	raw data documented in the manuscript
**Methodological approach**	
Empirical approach	parametric
Use of exposure variation	both variation over time and between populations
Method	mechanistic model (compartmental single-population transmission model)
Latent variable functionally linked to intervention	transmission rate
Code availability	none
**Effectiveness assessment**	
Reporting of intervention effectiveness	quantification of change in outcome values
Measure of effectiveness	transmission rate
Interpretation of results	implicitly causal
Reporting of uncertainty	no
Sensitivity analysis	none
Subgroup assessment	none

Chen et al. 2020c	
**Study information**	
Author	Chen YT, Yen YF, Yu SH, Su EC
Title	An Examination on the Transmission of COVID-19 and the Effect of Response Strategies: A Comparative Analysis
Year	2020
Journal	International Journal of Environmental Research and Public Health
DOI	10.3390/ijerph17165687
Date received	2020-06-24
Date accepted	2020-07-31
**Study setting**	
Number of populations included	multiple
Level of populations included	national (six countries: China, Korea, Japan, Italy, the USA, Brazil)
Geographic areas covered	Asia, Central and South America, Europe, North America
Number of countries covered	multiple countries
Study period	start and end date span first epidemic wave
**Outcome**	
Raw outcome	epidemiological population-level outcome (cases)
Time resolution of raw outcome	daily
Computed outcome	summary statistic (time to peak, maximum percentage reduction relative to peak after peak)
Method to obtain the computed outcome	simple computation
Data source	data from publicly available cross-country selections (WHO)
Data availability	data access via source
**Intervention**	
Terminology for interventions	strategies

– continued on next page –

Terminology for the specific type of non-pharmaceutical interventions	response
Exposure types	all interventions together
Types of single interventions	– not applicable –
Coding of interventions	– not applicable –
Source of intervention data	– not applicable –
Availability of data on exposure	– not applicable –
**Methodological approach**	
Empirical approach	descriptive
Use of exposure variation	both variation over time and between populations
Method	comparison of populations
Code availability	none
**Effectiveness assessment**	
Reporting of intervention effectiveness	qualitative statement
Measure of effectiveness	time to peak, maximum percentage reduction relative to peak after peak
Interpretation of results	implicitly causal
Reporting of uncertainty	– not applicable –
Sensitivity analysis	none
Subgroup assessment	none

Cheng et al. 2020a

**Study information**	
Author	Cheng W, Yu Z, Liu S, Sun W, Ling F, Pan J, Chen E

– continued on next page –

Title	Successful Interruption of Seasonal Influenza Transmission under the COVID-19 Rapid Response in Zhejiang Province, China
Year	2020
Journal	Public Health
DOI	10.1016/j.puhe.2020.10.011
Date received	2020-08-04
Date accepted	2020-09-13
**Study setting**	
Number of populations included	single
Level of populations included	subnational (Zhejiang Province (China))
Geographic areas covered	Asia
Number of countries covered	single country (China)
Study period	start and end date span first epidemic wave
**Outcome**	
Raw outcome	epidemiological population-level outcome (surrogate disease: cases influenza & hospital visits with influenza-like illnesses & test positivity rate influenza)
Time resolution of raw outcome	weekly
Computed outcome	none
Method to obtain the computed outcome	– not applicable –
Data source	data from (sub)national authorities (National Notifiable Diseases Reporting System: influenza, National Sentinel Influenza Surveillance Network: influenza-like illnesses)
Data availability	data access via source
**Intervention**	
Terminology for interventions	interventions

– continued on next page –

Terminology for the specific type of non-pharmaceutical interventions	non-pharmaceutical
Exposure types	one combination of interventions (level-1 public health response with many different measures (including travel restrictions, gathering bans, school and venue closure))
Types of single interventions	– not applicable –
Coding of interventions	not necessary
Source of intervention data	another study (Tian et al.)
Availability of data on exposure	raw data documented in the manuscript
**Methodological approach**	
Empirical approach	descriptive
Use of exposure variation	only variation over time for a single population
Method	description of change over time (comparison of time periods)
Code availability	none
**Effectiveness assessment**	
Reporting of intervention effectiveness	quantification of change in outcome values
Measure of effectiveness	cases: influenza, emergency room visits: influenza-like illness, test positivity rate: influenza
Interpretation of results	associative
Reporting of uncertainty	yes
Sensitivity analysis	none
Subgroup assessment	none

Cheng et al. 2020b	
**Study information**	
Author	Cheng Q, Liu Z, Cheng G, Huang J
Title	Heterogeneity and Effectiveness Analysis of COVID-19 Prevention and Control in Major Cities in China through Time-Varying Reproduction Number Estimation
Year	2020
Journal	Scientific Reports
DOI	10.1038/s41598-020-79063-x
Date received	2020-02-14
Date accepted	2020-12-03
**Study setting**	
Number of populations included	multiple
Level of populations included	subnational (25 cities in China)
Geographic areas covered	Asia
Number of countries covered	single country (China)
Study period	end date at peak of wave
**Outcome**	
Raw outcome	epidemiological population-level outcome (cases)
Time resolution of raw outcome	daily
Computed outcome	epidemiological parameter (reproduction number)
Method to obtain the computed outcome	statistical estimation of reproduction number (Cori et al.)
Data source	data from (sub)national authorities
Data availability	data made available by the authors
**Intervention**	
Terminology for interventions	measures

– continued on next page –

Terminology for the specific type of non-pharmaceutical interventions	prevention, control
Exposure types	all interventions together
Types of single interventions	– not applicable –
Coding of interventions	– not applicable –
Source of intervention data	– not applicable –
Availability of data on exposure	– not applicable –
**Methodological approach**	
Empirical approach	descriptive
Use of exposure variation	only variation over time for multiple populations
Method	description of change over time (description of time course)
Code availability	none
**Effectiveness assessment**	
Reporting of intervention effectiveness	qualitative statement
Measure of effectiveness	reproduction number
Interpretation of results	implicitly causal
Reporting of uncertainty	– not applicable –
Sensitivity analysis	none
Subgroup assessment	none

Cheng et al. 2020c (Analysis 1)

Study information

– continued on next page –

Author	Cheng C, Zhang T, Song C, Shen S, Jiang Y, Zhang X
Title	The Coupled Impact of Emergency Responses and Population Flows on the COVID-19 Pandemic in China
Year	2020
Journal	GeoHealth
DOI	10.1029/2020gh000332
Date received	2020-10-08
Date accepted	2020-11-18
**Study setting**	
Number of populations included	multiple
Level of populations included	both national and subnational (China and its provinces)
Geographic areas covered	Asia
Number of countries covered	single country (China)
Study period	start and end date span first epidemic wave
**Outcome**	
Raw outcome	epidemiological population-level outcome (cases)
Time resolution of raw outcome	daily
Computed outcome	none
Method to obtain the computed outcome	– not applicable –
Data source	data from publicly available open-source projects (public repository aggregating data from health authorities and other sources)
Data availability	data access via source
**Intervention**	
Terminology for interventions	responses

– continued on next page –

Terminology for the specific type of non-pharmaceutical interventions	emergency
Exposure types	stringency index (computation of a index based on level and timing of the public health response in the provinces)
Types of single interventions	– not applicable –
Coding of interventions	necessary
Source of intervention data	coding done by authors
Availability of data on exposure	coded data made available by the authors
**Methodological approach**	
Empirical approach	parametric
Use of exposure variation	both variation over time and between populations
Method	non-mechanistic model (correlation)
Code availability	none
**Effectiveness assessment**	
Reporting of intervention effectiveness	quantification of change in outcome values
Measure of effectiveness	cases
Interpretation of results	implicitly causal
Reporting of uncertainty	yes
Sensitivity analysis	none
Subgroup assessment	none

Cheng et al. 2020c (Analysis 2)

**Study information**	
Author	Cheng C, Zhang T, Song C, Shen S, Jiang Y, Zhang X

– continued on next page –

Title	The Coupled Impact of Emergency Responses and Population Flows on the COVID-19 Pandemic in China
Year	2020
Journal	GeoHealth
DOI	10.1029/2020gh000332
Date received	2020-10-08
Date accepted	2020-11-18
**Study setting**	
Number of populations included	multiple
Level of populations included	both national and subnational (China and its provinces)
Geographic areas covered	Asia
Number of countries covered	single country (China)
Study period	start and end date span first epidemic wave
**Outcome**	
Raw outcome	epidemiological population-level outcome (cases)
Time resolution of raw outcome	daily
Computed outcome	none
Method to obtain the computed outcome	– not applicable –
Data source	data from publicly available open-source projects (public repository aggregating data from health authorities and other sources)
Data availability	data access via source
**Intervention**	
Terminology for interventions	responses
Terminology for the specific type of non-pharmaceutical interventions	emergency

– continued on next page –

Exposure types	stringency index (computation of a index based on level and timing of the public health response in the provinces)
Types of single interventions	– not applicable –
Coding of interventions	necessary
Source of intervention data	coding done by authors
Availability of data on exposure	coded data made available by the authors
**Methodological approach**	
Empirical approach	parametric
Use of exposure variation	both variation over time and between populations
Method	non-mechanistic model (other: weighted regression)
Code availability	none
**Effectiveness assessment**	
Reporting of intervention effectiveness	quantification of change in outcome values
Measure of effectiveness	cases
Interpretation of results	implicitly causal
Reporting of uncertainty	yes
Sensitivity analysis	none
Subgroup assessment	none

Cheng et al. 2020c (Analysis 3, mobility as exposure)

**Study information**	
Author	Cheng C, Zhang T, Song C, Shen S, Jiang Y, Zhang X
Title	The Coupled Impact of Emergency Responses and Population Flows on the COVID-19 Pandemic in China

– continued on next page –

Year	2020
Journal	GeoHealth
DOI	10.1029/2020gh000332
Date received	08.10.2020
Date accepted	18.12.2020
**Study setting**	
Number of populations included	multiple
Level of populations included	both national and subnational (China and its provinces)
Geographic areas covered	Asia
Number of countries covered	single country (China)
Study period	start and end date span first epidemic wave
**Outcome**	
Raw outcome	epidemiological population-level outcome (cases)
Time resolution of raw outcome	daily
Computed outcome	none
Method to obtain the computed outcome	– not applicable –
Data source	data from publicly available open-source projects (public repository aggregating data from health authorities and other sources)
Data availability	data access via source
**Intervention**	
Terminology for interventions	responses
Terminology for the specific type of non-pharmaceutical interventions	emergency
Exposure types	mobility (proportion of population moving out of Wuhan that moves to certain province, relative population inflow into province from across country)

– continued on next page –

Types of single interventions	– not applicable –
Coding of interventions	– not applicable –
Source of intervention data	mobility data by corporate organizations (Baidu)
Availability of data on exposure	data access via source
**Methodological approach**	
Empirical approach	descriptive
Use of exposure variation	both variation over time and between populations
Method	non-mechanistic model (correlation)
Code availability	none
**Effectiveness assessment**	
Reporting of intervention effectiveness	quantification of change in outcome values
Measure of effectiveness	cases
Interpretation of results	implicitly causal
Reporting of uncertainty	yes
Sensitivity analysis	none
Subgroup assessment	none

Cheng et al. 2020d

**Study information**	
Author	Cheng VC, Wong SC, Chuang VW, So SY, Chen JH, Sridhar S, To KK, Chan JF, Hung IF, Ho PL, Yuen KY
Title	The Role of Community-Wide Wearing of Face Mask for Control of Coronavirus Disease 2019 (COVID-19) Epidemic Due to SARS-CoV-2
Year	2020

– continued on next page –

Journal	Journal of Infection
DOI	10.1016/j.jinf.2020.04.024
Date received	– could not be evaluated –
Date accepted	2020-04-17
**Study setting**	
Number of populations included	multiple
Level of populations included	national (Hong Kong Special Administrative Region, Germany, France, Italy, Spain, UK, South Korea, Singapore, US)
Geographic areas covered	Asia, Europe, North America
Number of countries covered	multiple countries
Study period	same end date for several populations with diverse epidemic trajectories
**Outcome**	
Raw outcome	epidemiological population-level outcome (cases)
Time resolution of raw outcome	daily
Computed outcome	none
Method to obtain the computed outcome	– not applicable –
Data source	data from publicly available cross-country selections (WHO)
Data availability	data access via source
**Intervention**	
Terminology for interventions	interventions
Terminology for the specific type of non-pharmaceutical interventions	non-pharmaceutical
Exposure types	one single intervention (mask mandate)
Types of single interventions	mask mandate
Coding of interventions	not necessary

– continued on next page –

Source of intervention data	government or news websites
Availability of data on exposure	raw data documented in the manuscript
**Methodological approach**	
Empirical approach	descriptive
Use of exposure variation	only variation between populations
Method	comparison of populations
Code availability	none
**Effectiveness assessment**	
Reporting of intervention effectiveness	quantification of change in outcome values
Measure of effectiveness	cases
Interpretation of results	associative
Reporting of uncertainty	yes
Sensitivity analysis	none
Subgroup assessment	none

Chernozhukov et al. 2021 (Analysis 1)

**Study information**	
Author	Chernozhukov V, Kasahara H, Schrimpf P
Title	Causal Impact of Masks, Policies, Behavior on Early Covid-19 Pandemic in the U.S
Year	2021
Journal	Journal of Econometrics

– continued on next page –

DOI	10.1016/j.jeconom.2020.09.003
Date received	2020-07-03
Date accepted	2020-09-15
**Study setting**	
Number of populations included	multiple
Level of populations included	both national and subnational (US and its states)
Geographic areas covered	North America
Number of countries covered	single country (US)
Study period	same end date for several populations with diverse epidemic trajectories
**Outcome**	
Raw outcome	epidemiological population-level outcome (cases, deaths), behavioral outcome (mobility: time spent in various locations)
Time resolution of raw outcome	daily
Computed outcome	measure of epidemic trend (growth rate, growth rate: deaths)
Method to obtain the computed outcome	simple computation
Data source	data from publicly available cross-country selections (New York Times), mobility data from corporate organizations (Google)
Data availability	data made available by the authors
**Intervention**	
Terminology for interventions	policies
Terminology for the specific type of non-pharmaceutical interventions	social distancing
Exposure types	multiple separate interventions (stay-at-home, closed nonessential businesses, closed K-12 schools, closed restaurants except takeout, closed movie theaters, and face mask mandates for employees in public facing businesses)

– continued on next page –

Types of single interventions	stay-at-home order, workplace closure, school closure, venue closure, mask mandate
Coding of interventions	necessary
Source of intervention data	use of externally coded data (Boston University COVID-19 US State Policy Database)
Availability of data on exposure	coded data made available by the authors
**Methodological approach**	
Empirical approach	parametric
Use of exposure variation	both variation over time and between populations
Method	non-mechanistic model (generalized linear model)
Code availability	publicly available
**Effectiveness assessment**	
Reporting of intervention effectiveness	quantification of change in outcome values
Measure of effectiveness	growth rate, growth rate:deaths, mobility: time spent in various locations
Interpretation of results	explicitly causal
Reporting of uncertainty	yes
Sensitivity analysis	different coding of interventions used, same analysis with (sub)population(s) excluded, model specification varied
Subgroup assessment	none

Chernozhukov et al. 2021 (Analysis 2, mobility as exposure)

**Study information**	
Author	Chernozhukov V, Kasahara H, Schrimpf P
Title	Causal Impact of Masks, Policies, Behavior on Early Covid-19 Pandemic in the U.S

– continued on next page –

Year	2021
Journal	Journal of Econometrics
DOI	10.1016/j.jeconom.2020.09.003
Date received	2020-07-03
Date accepted	2020-09-15
**Study setting**	
Number of populations included	multiple
Level of populations included	both national and subnational (US and its states)
Geographic areas covered	North America
Number of countries covered	single country (US)
Study period	same end date for several populations with diverse epidemic trajectories
**Outcome**	
Raw outcome	epidemiological population-level outcome (cases, deaths)
Time resolution of raw outcome	daily
Computed outcome	measure of epidemic trend (growth rate, growth rate: deaths)
Method to obtain the computed outcome	simple computation
Data source	data from publicly available cross-country selections (New York Times)
Data availability	data made available by the authors
**Intervention**	
Terminology for interventions	policies
Terminology for the specific type of non-pharmaceutical interventions	social distancing
Exposure types	mobility (time spent in various locations)
Types of single interventions	– not applicable –
Coding of interventions	– not applicable –

– continued on next page –

Source of intervention data	mobility data by corporate organizations (Google)
Availability of data on exposure	data made available by the authors
**Methodological approach**	
Empirical approach	parametric
Use of exposure variation	both variation over time and between populations
Method	non-mechanistic model (generalized linear model)
Code availability	publicly available
**Effectiveness assessment**	
Reporting of intervention effectiveness	quantification of change in outcome values
Measure of effectiveness	growth rate, growth rate: deaths
Interpretation of results	explicitly causal
Reporting of uncertainty	yes
Sensitivity analysis	model specification varied
Subgroup assessment	none

**Choi & Ki 2020**	
**Study information**	
Author	Choi S, Ki M
Title	Analyzing the Effects of Social Distancing on the COVID-19 Pandemic in Korea Using Mathematical Modeling
Year	2020
Journal	Epidemiology and Health
DOI	10.4178/epih.e2020064

– continued on next page –

Date received	2020-06-16
Date accepted	2020-08-19
**Study setting**	
Number of populations included	single
Level of populations included	national (South Korea)
Geographic areas covered	Asia
Number of countries covered	single country (South Korea)
Study period	start and end date span first epidemic wave
**Outcome**	
Raw outcome	epidemiological population-level outcome (cases)
Time resolution of raw outcome	daily
Computed outcome	epidemiological parameter (reproduction number)
Method to obtain the computed outcome	compartmental transmission model (single-population)
Data source	data from (sub)national authorities
Data availability	data access via source
**Intervention**	
Terminology for interventions	measures
Terminology for the specific type of non-pharmaceutical interventions	social distancing
Exposure types	phases of interventions (social distancing policies of varying strictness divided into 5 phases but where the phases are manually derived based on observed changes in the outcome (the phases are guided by the NPI dates, but changepoints were defined manually by inspecting the 2nd order difference (second derivative) of the cumulative case time series))
Types of single interventions	– not applicable –
Coding of interventions	not necessary

– continued on next page –

Source of intervention data	– could not be evaluated –
Availability of data on exposure	raw data documented in the manuscript
**Methodological approach**	
Empirical approach	descriptive
Use of exposure variation	only variation over time for a single population
Method	description of change over time (comparison of time periods)
Code availability	none
**Effectiveness assessment**	
Reporting of intervention effectiveness	comparison of outcome values
Measure of effectiveness	reproduction number
Interpretation of results	implicitly causal
Reporting of uncertainty	no
Sensitivity analysis	none
Subgroup assessment	none

Choi et al. 2020

**Study information**	
Author	Choi Y, Kim JS, Choi H, Lee H, Lee CH
Title	Assessment of Social Distancing for Controlling COVID-19 in Korea: An Age-Structured Modeling Approach
Year	2020
Journal	International Journal of Environmental Research and Public Health
DOI	10.3390/ijerph17207474

– continued on next page –

Date received	2020-08-28
Date accepted	2020-10-12
**Study setting**	
Number of populations included	single
Level of populations included	subnational (Seougl and Gyenggi provinces in South Korea)
Geographic areas covered	Asia
Number of countries covered	single country (South Korea)
Study period	includes second wave (two waves)
**Outcome**	
Raw outcome	epidemiological population-level outcome (cases, deaths, recovered cases)
Time resolution of raw outcome	daily
Computed outcome	epidemiological parameter (reproduction number, transmission probability)
Method to obtain the computed outcome	compartmental transmission model (single-population)
Data source	data from (sub)national authorities
Data availability	data access via source
**Intervention**	
Terminology for interventions	measures
Terminology for the specific type of non-pharmaceutical interventions	social distancing
Exposure types	all interventions together (timeline of measures divided into three phases)
Types of single interventions	– not applicable –
Coding of interventions	– not applicable –
Source of intervention data	– not applicable –
Availability of data on exposure	– not applicable –
**Methodological approach**	

– continued on next page –

Empirical approach	descriptive
Use of exposure variation	only variation over time for a single population
Method	description of change over time (comparison of time periods)
Code availability	none
**Effectiveness assessment**	
Reporting of intervention effectiveness	comparison of outcome values
Measure of effectiveness	reproduction number, probability of transmission upon contact
Interpretation of results	implicitly causal
Reporting of uncertainty	no
Sensitivity analysis	none
Subgroup assessment	socioeconomic indicators (age)

Cobb & Seale 2020

**Study information**	
Author	Cobb JS, Seale MA
Title	Examining the Effect of Social Distancing on the Compound Growth Rate of COVID-19 at the County Level (United States) Using Statistical Analyses and a Random Forest Machine Learning Model
Year	2020
Journal	Public Health
DOI	10.1016/j.puhe.2020.04.016
Date received	2020-04-10
Date accepted	2020-04-13
**Study setting**	

– continued on next page –

Number of populations included	multiple
Level of populations included	both national and subnational (US counties)
Geographic areas covered	North America
Number of countries covered	single country (US)
Study period	end date in growth phase of wave
**Outcome**	
Raw outcome	epidemiological population-level outcome (cases)
Time resolution of raw outcome	daily
Computed outcome	measure of epidemic trend (growth rate)
Method to obtain the computed outcome	simple computation
Data source	data from (sub)national authorities (till March 25), data from public media outlets (New York Times: beyond March 25)
Data availability	data access via source
**Intervention**	
Terminology for interventions	measures
Terminology for the specific type of non-pharmaceutical interventions	social distancing
Exposure types	multiple separate interventions (presidential recommendation to limit gatherings, shelter-in-place order)
Types of single interventions	other, stay-at-home order
Coding of interventions	necessary
Source of intervention data	– could not be evaluated –
Availability of data on exposure	coded data made available by the authors
**Methodological approach**	
Empirical approach	descriptive

– continued on next page –

Use of exposure variation	only variation over time for a single population
Method	comparison of populations
Code availability	none
**Effectiveness assessment**	
Reporting of intervention effectiveness	quantification of change in outcome values
Measure of effectiveness	growth rate
Interpretation of results	implicitly causal
Reporting of uncertainty	yes
Sensitivity analysis	none
Subgroup assessment	none

Coletti et al. 2020

**Study information**	
Author	Coletti P, Wambua J, Gimma A, Willem L, Vercruysse S, Vanhoutte B, Jarvis CI, Van Zandvoort K, Edmunds J, Beutels P, Hens N
Title	CoMix: Comparing Mixing Patterns in the Belgian Population during and after Lockdown
Year	2020
Journal	Scientific Reports
DOI	10.1038/s41598-020-78540-7
Date received	2020-08-25
Date accepted	2020-12-14
**Study setting**	
Number of populations included	single

– continued on next page –

Level of populations included	national (Belgium)
Geographic areas covered	Europe
Number of countries covered	single country (Belgium)
Study period	start and end date span first epidemic wave: behavioral
**Outcome**	
Raw outcome	behavioral outcome (survey: number of contacts & face mask use)
Time resolution of raw outcome	– not applicable –
Computed outcome	epidemiological parameter (reproduction number)
Method to obtain the computed outcome	statistical estimation of reproduction number from individual data (Diekmann et al.)
Data source	data collected by authors
Data availability	data made available by the authors
**Intervention**	
Terminology for interventions	measures
Terminology for the specific type of non-pharmaceutical interventions	social distancing
Exposure types	all interventions together (timeline of measures)
Types of single interventions	– not applicable –
Coding of interventions	– not applicable –
Source of intervention data	– not applicable –
Availability of data on exposure	– not applicable –
**Methodological approach**	
Empirical approach	descriptive
Use of exposure variation	only variation over time for a single population
Method	description of change over time (comparison of time periods)

– continued on next page –

Code availability	none
**Effectiveness assessment**	
Reporting of intervention effectiveness	comparison of outcome values
Measure of effectiveness	number of contacts, face mask usage, reproduction number
Interpretation of results	associative
Reporting of uncertainty	no
Sensitivity analysis	none
Subgroup assessment	socioeconomic indicators (age, gender, household size)

**Collins & Duffy 2020**	
**Study information**	
Author	Collins OC, Duffy KJ
Title	Estimating the Impact of Lock-down, Quarantine and Sensitization in a COVID-19 Outbreak: Lessons from the COVID-19 Outbreak in China
Year	2020
Journal	PeerJ
DOI	10.7717/peerj.9933
Date received	2020-05-27
Date accepted	2020-08-23
**Study setting**	
Number of populations included	single
Level of populations included	national (China)
Geographic areas covered	Asia

– continued on next page –

Number of countries covered	single country (China)
Study period	start and end date span first epidemic wave
**Outcome**	
Raw outcome	epidemiological population-level outcome (active cases)
Time resolution of raw outcome	daily
Computed outcome	none
Method to obtain the computed outcome	– not applicable –
Data source	data from publicly available cross-country selections (Worldometer)
Data availability	data made available by the authors
**Intervention**	
Terminology for interventions	measures
Terminology for the specific type of non-pharmaceutical interventions	control
Exposure types	categories of interventions (sensitization, quarantine, lockdown and the combination of all three)
Types of single interventions	– not applicable –
Coding of interventions	– not applicable –
Source of intervention data	– not applicable –
Availability of data on exposure	– not applicable –
**Methodological approach**	
Empirical approach	parametric
Use of exposure variation	only variation over time for a single population
Method	mechanistic model (compartmental single-population transmission model)
Latent variable functionally linked to intervention	susceptible decay, transmission rate, quarantine rate

– continued on next page –

Code availability	publicly available
**Effectiveness assessment**	
Reporting of intervention effectiveness	quantification of change in outcome values
Measure of effectiveness	susceptible decay rate, transmission rate, quarantine rate
Interpretation of results	implicitly causal
Reporting of uncertainty	no
Sensitivity analysis	none
Subgroup assessment	none

Cooper et al. 2020

**Study information**	
Author	Cooper I, Mondal A, Antonopoulos CG
Title	A SIR Model Assumption for the Spread of COVID-19 in Different Communities
Year	2020
Journal	Chaos, Solitons & Fractals
DOI	10.1016/j.chaos.2020.110057
Date received	2020-06-18
Date accepted	2020-06-23
**Study setting**	
Number of populations included	multiple
Level of populations included	both national and subnational (China, South Korea, India, Australia, USA, Italy, Texas)
Geographic areas covered	Asia, Oceania, Europe, North America

– continued on next page –

Number of countries covered	multiple countries
Study period	same end date for several populations with diverse epidemic trajectories
**Outcome**	
Raw outcome	epidemiological population-level outcome (cases, deaths, recovered cases)
Time resolution of raw outcome	daily
Computed outcome	other (infections)
Method to obtain the computed outcome	compartmental transmission model (single-population)
Data source	data from publicly available cross-country selections (Worldometer)
Data availability	data access via source
**Intervention**	
Terminology for interventions	measures
Terminology for the specific type of non-pharmaceutical interventions	control
Exposure types	all interventions together
Types of single interventions	– not applicable –
Coding of interventions	– not applicable –
Source of intervention data	– not applicable –
Availability of data on exposure	– not applicable –
**Methodological approach**	
Empirical approach	descriptive
Use of exposure variation	only variation over time for multiple populations
Method	description of change over time (description of time course)
Code availability	none
**Effectiveness assessment**	

– continued on next page –

Reporting of intervention effectiveness	qualitative statement
Measure of effectiveness	infections
Interpretation of results	associative
Reporting of uncertainty	– not applicable –
Sensitivity analysis	none
Subgroup assessment	none

**Courtemanche et al. 2020**	
**Study information**	
Author	Courtemanche C, Garuccio J, Le A, Pinkston J, Yelowitz A
Title	Strong Social Distancing Measures in the United States Reduced the COVID-19 Growth Rate
Year	2020
Journal	Health Affairs
DOI	10.1377/hlthaff.2020.00608
Date received	– could not be evaluated –
Date accepted	2020-05-14 (published)
**Study setting**	
Number of populations included	multiple
Level of populations included	both national and subnational (US counties)
Geographic areas covered	North America
Number of countries covered	single country (US)
Study period	same end date for several populations with diverse epidemic trajectories

– continued on next page –

**Outcome**	
Raw outcome	epidemiological population-level outcome (cases)
Time resolution of raw outcome	daily
Computed outcome	measure of epidemic trend (growth rate)
Method to obtain the computed outcome	simple computation
Data source	data from publicly available cross-country selections (JHU)
Data availability	data access via source
**Intervention**	
Terminology for interventions	measures
Terminology for the specific type of non-pharmaceutical interventions	social distancing
Exposure types	multiple separate interventions (bans of large gatherings, school closure, venue closure, stay-at-home order)
Types of single interventions	bans of large gatherings, school closure, venue closure, stay-at-home order
Coding of interventions	necessary
Source of intervention data	use of externally coded data (same as in Killeen et al.)
Availability of data on exposure	coded data made available by the authors
**Methodological approach**	
Empirical approach	parametric
Use of exposure variation	both variation over time and between populations
Method	non-mechanistic model (generalized linear model)
Code availability	none
**Effectiveness assessment**	
Reporting of intervention effectiveness	quantification of change in outcome values

– continued on next page –

Measure of effectiveness	growth rate
Interpretation of results	implicitly causal
Reporting of uncertainty	yes
Sensitivity analysis	different coding of interventions used (gathering bans of 50 instead of 500, closure of restaurants AND gyms, not OR), same analysis with (sub)population(s) excluded, model specification varied
Subgroup assessment	none

	**Cowling et al. 2020 (Analysis 1)**
**Study information**	
Author	Cowling BJ, Ali ST, Ng TW, Tsang TK, Li JC, Fong MW, Liao Q, Kwan MY, Lee SL, Chiu SS, Wu JT, Wu P, Leung GM
Title	Impact Assessment of Non-Pharmaceutical Interventions against Coronavirus Disease 2019 and Influenza in Hong Kong: An Observational Study
Year	2020
Journal	The Lancet Public Health
DOI	10.1016/s2468-2667(20)30090-6
Date received	– could not be evaluated –
Date accepted	2020-04-17 (published)
**Study setting**	
Number of populations included	single
Level of populations included	national (Hong Kong)
Geographic areas covered	Asia
Number of countries covered	single country (Hong Kong)

– continued on next page –

Study period	start and end date span first epidemic wave
**Outcome**	
Raw outcome	epidemiological individual-level outcome (cases, surrogate disease: cases with influenza & hospitalizations children with influenza)
Time resolution of raw outcome	daily
Computed outcome	epidemiological parameter (reproduction number, reproduction number: influenza, reproduction number: influenza of hospitalized children)
Method to obtain the computed outcome	statistical estimation of reproduction number (Cori et al.)
Data source	data from (sub)national authorities
Data availability	data access via source
**Intervention**	
Terminology for interventions	interventions
Terminology for the specific type of non-pharmaceutical interventions	non-pharmaceutical
Exposure types	one single intervention (school closure)
Types of single interventions	school closure
Coding of interventions	not necessary
Source of intervention data	– could not be evaluated –
Availability of data on exposure	raw data documented in the manuscript
**Methodological approach**	
Empirical approach	descriptive
Use of exposure variation	only variation over time for a single population
Method	description of change over time (comparison of time periods)
Code availability	none
**Effectiveness assessment**	

– continued on next page –

Reporting of intervention effectiveness	quantification of change in outcome values
Measure of effectiveness	reproduction number: SARS-CoV-2, surrogate disease: influenza, surrogate disease: influenza in hospitalized children
Interpretation of results	associative
Reporting of uncertainty	yes
Sensitivity analysis	none
Subgroup assessment	none

	**Cowling et al. 2020 (Analysis 2)**
**Study information**	
Author	Cowling BJ, Ali ST, Ng TW, Tsang TK, Li JC, Fong MW, Liao Q, Kwan MY, Lee SL, Chiu SS, Wu JT, Wu P, Leung GM
Title	Impact Assessment of Non-Pharmaceutical Interventions against Coronavirus Disease 2019 and Influenza in Hong Kong: An Observational Study
Year	2020
Journal	The Lancet Public Health
DOI	10.1016/s2468-2667(20)30090-6
Date received	– could not be evaluated –
Date accepted	2020-04-17 (published)
**Study setting**	
Number of populations included	single
Level of populations included	national (Hong Kong)
Geographic areas covered	Asia
Number of countries covered	single country (Hong Kong)

– continued on next page –

Study period	start and end date span first epidemic wave: behavioral
**Outcome**	
Raw outcome	behavioral outcome (survey: responses to behavioral adherence to measures, e.g., use of face masks & avoidance of public places)
Time resolution of raw outcome	– not applicable –
Computed outcome	none
Method to obtain the computed outcome	– not applicable –
Data source	data collected by authors
Data availability	data not accessible
**Intervention**	
Terminology for interventions	interventions
Terminology for the specific type of non-pharmaceutical interventions	non-pharmaceutical
Exposure types	all interventions together (timeline of measures)
Types of single interventions	– not applicable –
Coding of interventions	– not applicable –
Source of intervention data	– not applicable –
Availability of data on exposure	– not applicable –
**Methodological approach**	
Empirical approach	descriptive
Use of exposure variation	only variation over time for a single population
Method	description of change over time (description of time course)
Code availability	none
**Effectiveness assessment**	

– continued on next page –

Reporting of intervention effectiveness	comparison of outcome values
Measure of effectiveness	face mask usage, avoidance of public places
Interpretation of results	associative
Reporting of uncertainty	yes
Sensitivity analysis	none
Subgroup assessment	none

**Crokidakis 2020**	
**Study information**	
Author	Crokidakis N
Title	COVID-19 Spreading in Rio de Janeiro, Brazil: Do the Policies of Social Isolation Really Work?
Year	2020
Journal	Chaos, Solitons & Fractals
DOI	10.1016/j.chaos.2020.109930
Date received	2020-05-05
Date accepted	2020-05-20
**Study setting**	
Number of populations included	single
Level of populations included	subnational (Rio de Janeiro)
Geographic areas covered	Central and South America
Number of countries covered	single country (Brazil)
Study period	end date in growth phase of wave

– continued on next page –

**Outcome**	
Raw outcome	epidemiological population-level outcome (cases)
Time resolution of raw outcome	daily
Computed outcome	none
Method to obtain the computed outcome	– not applicable –
Data source	data from (sub)national authorities
Data availability	data access via source
**Intervention**	
Terminology for interventions	policies
Terminology for the specific type of non-pharmaceutical interventions	social isolation
Exposure types	all interventions together
Types of single interventions	– not applicable –
Coding of interventions	– not applicable –
Source of intervention data	– not applicable –
Availability of data on exposure	– not applicable –
**Methodological approach**	
Empirical approach	parametric
Use of exposure variation	only variation over time for a single population
Method	mechanistic model (compartmental single-population transmission model)
Latent variable functionally linked to intervention	susceptible decay
Code availability	none
**Effectiveness assessment**	

– continued on next page –

Reporting of intervention effectiveness	quantification of change in outcome values
Measure of effectiveness	cases, quarantined
Interpretation of results	implicitly causal
Reporting of uncertainty	no
Sensitivity analysis	none
Subgroup assessment	none

**Czerwińska & Szenborn 2020**	
**Study information**	
Author	Czerwińska E, Szenborn L
Title	The COVID-19 Pandemic Lockdown Causes a Decrease in the Number of Cases of Other Infectious Diseases in Poland
Year	2020
Journal	Pediatria Polska
DOI	10.5114/polp.2020.97221
Date received	2020-06-04
Date accepted	2020-06-22
**Study setting**	
Number of populations included	single
Level of populations included	national (Poland)
Geographic areas covered	Europe
Number of countries covered	single country (Poland)
Study period	end date at peak of wave

– continued on next page –

**Outcome**	
Raw outcome	epidemiological population-level outcome (surrogate disease: cases of multiple other infectious diseases)
Time resolution of raw outcome	biweekly
Computed outcome	none
Method to obtain the computed outcome	– not applicable –
Data source	data from (sub)national authorities
Data availability	data access via source
**Intervention**	
Terminology for interventions	measures
Terminology for the specific type of non-pharmaceutical interventions	social distancing
Exposure types	all interventions together (stay-at-home order, school closure, bans of small gatherings, mask mandate)
Types of single interventions	– not applicable –
Coding of interventions	– not applicable –
Source of intervention data	– not applicable –
Availability of data on exposure	– not applicable –
**Methodological approach**	
Empirical approach	descriptive
Use of exposure variation	only variation over time for a single population
Method	description of change over time (comparison of time periods)
Code availability	none
**Effectiveness assessment**	

– continued on next page –

Reporting of intervention effectiveness	quantification of change in outcome values
Measure of effectiveness	hospitalizations: acute bronchiolitis in infants
Interpretation of results	implicitly causal
Reporting of uncertainty	no
Sensitivity analysis	none
Subgroup assessment	none

**da Silva & Tsigaris 2020**	
**Study information**	
Author	da Silva JA, Tsigaris P
Title	Policy Determinants of COVID-19 Pandemic–Induced Fatality Rates across Nations
Year	2020
Journal	Public Health
DOI	10.1016/j.puhe.2020.08.008
Date received	2020-05-09
Date accepted	2020-08-12
**Study setting**	
Number of populations included	multiple
Level of populations included	national (121 countries areound the world)
Geographic areas covered	Europe, Middle East and Africa, Asia, Oceania, Central and South America, North America
Number of countries covered	multiple countries

– continued on next page –

Study period	same end date for several populations with diverse epidemic trajectories
**Outcome**	
Raw outcome	epidemiological population-level outcome (cases, deaths)
Time resolution of raw outcome	daily
Computed outcome	epidemiological parameter (case-fatality rate)
Method to obtain the computed outcome	simple computation
Data source	data from publicly available cross-country selections (Worldometer)
Data availability	data access via source
**Intervention**	
Terminology for interventions	measures
Terminology for the specific type of non-pharmaceutical interventions	containment
Exposure types	start of interventions (days since first policy, delay for international travel restrictions, delay for public information campaign, delay for testing policy)
Types of single interventions	– not applicable –
Coding of interventions	necessary
Source of intervention data	use of externally coded data (OxCGRT)
Availability of data on exposure	access to externally coded data via source
**Methodological approach**	
Empirical approach	parametric
Use of exposure variation	only variation between populations
Method	non-mechanistic model (generalized linear model)
Code availability	none
**Effectiveness assessment**	

– continued on next page –

Reporting of intervention effectiveness	quantification of change in outcome values
Measure of effectiveness	case-fatality rate
Interpretation of results	associative
Reporting of uncertainty	yes
Sensitivity analysis	none
Subgroup assessment	socioeconomic indicators (income)

**Dai et al. 2020**	
**Study information**	
Author	Dai C, Yang AJ, Wang K, and
Title	Evaluation of Prevention and Control Interventions and Its Impact on the Epidemic of Coronavirus Disease 2019 in Chongqing and Guizhou Provinces
Year	2020
Journal	Mathematical Biosciences and Engineering
DOI	10.3934/mbe.2020152
Date received	2020-02-12
Date accepted	2020-03-11
**Study setting**	
Number of populations included	multiple
Level of populations included	subnational (Chongqing and Guizhou province in China)
Geographic areas covered	Asia
Number of countries covered	single country (China)
Study period	start and end date span first epidemic wave

– continued on next page –

**Outcome**	
Raw outcome	epidemiological population-level outcome (cases)
Time resolution of raw outcome	daily
Computed outcome	none
Method to obtain the computed outcome	– not applicable –
Data source	data from (sub)national authorities
Data availability	data access via source
**Intervention**	
Terminology for interventions	measures
Terminology for the specific type of non-pharmaceutical interventions	prevention, control
Exposure types	one combination of interventions (Wuhan City Lockdown)
Types of single interventions	– not applicable –
Coding of interventions	not necessary
Source of intervention data	– could not be evaluated –
Availability of data on exposure	raw data documented in the manuscript
**Methodological approach**	
Empirical approach	parametric
Use of exposure variation	only variation over time for multiple populations
Method	mechanistic model (compartmental single-population transmission model)
Latent variable functionally linked to intervention	contact rate
Code availability	none
**Effectiveness assessment**	

– continued on next page –

Reporting of intervention effectiveness	quantification of change in outcome values
Measure of effectiveness	contact rate
Interpretation of results	implicitly causal
Reporting of uncertainty	no
Sensitivity analysis	none
Subgroup assessment	none

**Dandekar et al. 2020**	
**Study information**	
Author	Dandekar R, Rackauckas C, Barbastathis G
Title	A Machine Learning-Aided Global Diagnostic and Comparative Tool to Assess Effect of Quarantine Control in COVID-19 Spread
Year	2020
Journal	Patterns
DOI	10.1016/j.patter.2020.100145
Date received	2020-07-22
Date accepted	2020-10-21
**Study setting**	
Number of populations included	multiple
Level of populations included	both national and subnational (70 countries worldwide, 20 major US states)
Geographic areas covered	Europe, Middle East and Africa, Asia, Oceania, Central and South America, North America
Number of countries covered	multiple countries

– continued on next page –

Study period	same end date for several populations with diverse epidemic trajectories
**Outcome**	
Raw outcome	epidemiological population-level outcome (cases, recovered cases)
Time resolution of raw outcome	daily
Computed outcome	none
Method to obtain the computed outcome	– not applicable –
Data source	data from publicly available cross-country selections (JHU)
Data availability	data made available by the authors
**Intervention**	
Terminology for interventions	policies
Terminology for the specific type of non-pharmaceutical interventions	quarantine
Exposure types	all interventions together
Types of single interventions	– not applicable –
Coding of interventions	– not applicable –
Source of intervention data	– not applicable –
Availability of data on exposure	– not applicable –
**Methodological approach**	
Empirical approach	parametric
Use of exposure variation	only variation over time for multiple populations
Method	mechanistic model (compartmental single-population transmission model)
Latent variable functionally linked to intervention	quarantine rate
Code availability	publicly available
**Effectiveness assessment**	

– continued on next page –

Reporting of intervention effectiveness	quantification of change in outcome values
Measure of effectiveness	quarantine rate
Interpretation of results	associative
Reporting of uncertainty	no
Sensitivity analysis	none
Subgroup assessment	none

**de Brito Cruz 2020 (Analysis 1)**	
**Study information**	
Author	de Brito Cruz CH
Title	Social Distancing in São Paulo State: Demonstrating the Reduction in Cases Using Time Series Analysis of Deaths Due to COVID-19
Year	2020
Journal	Revista Brasileira de Epidemiologia
DOI	10.1590/1980-549720200056
Date received	2020-05-12
Date accepted	2020-05-14
**Study setting**	
Number of populations included	multiple
Level of populations included	both national and subnational (Brazil, Sao Paulo State, Sao Paulo City)
Geographic areas covered	Central and South America
Number of countries covered	single country (Brazil)

– continued on next page –

Study period	end date in growth phase of wave
**Outcome**	
Raw outcome	epidemiological population-level outcome (deaths)
Time resolution of raw outcome	daily
Computed outcome	none
Method to obtain the computed outcome	– not applicable –
Data source	data from (sub)national authorities (National Association of the Registrars for Natural Persons)
Data availability	data access via source
**Intervention**	
Terminology for interventions	measures
Terminology for the specific type of non-pharmaceutical interventions	social distancing
Exposure types	all interventions together
Types of single interventions	– not applicable –
Coding of interventions	– not applicable –
Source of intervention data	– not applicable –
Availability of data on exposure	– not applicable –
**Methodological approach**	
Empirical approach	descriptive
Use of exposure variation	only variation over time for multiple populations
Method	description of change over time (description of time course)
Code availability	none
**Effectiveness assessment**	

– continued on next page –

Reporting of intervention effectiveness	qualitative statement
Measure of effectiveness	deaths
Interpretation of results	implicitly causal
Reporting of uncertainty	– not applicable –
Sensitivity analysis	none
Subgroup assessment	none

**de Brito Cruz 2020 (Analysis 2, mobility as exposure)**	
**Study information**	
Author	de Brito Cruz CH
Title	Social Distancing in São Paulo State: Demonstrating the Reduction in Cases Using Time Series Analysis of Deaths Due to COVID-19
Year	2020
Journal	Revista Brasileira de Epidemiologia
DOI	10.1590/1980-549720200056
Date received	12.05.2020
Date accepted	14.05.2020
**Study setting**	
Number of populations included	multiple
Level of populations included	both national and subnational (Brazil, Sao Paulo State, Sao Paulo City)
Geographic areas covered	Central and South America
Number of countries covered	single country (Brazil)
Study period	end date in growth phase of wave
**Outcome**	

– continued on next page –

Raw outcome	epidemiological population-level outcome (deaths)
Time resolution of raw outcome	daily
Computed outcome	measure of epidemic trend (growth rate: deaths)
Method to obtain the computed outcome	simple computation
Data source	data from (sub)national authorities (National Association of the Registrars for Natural Persons)
Data availability	data access via source
**Intervention**	
Terminology for interventions	measures
Terminology for the specific type of non-pharmaceutical interventions	social distancing
Exposure types	mobility (index based on São Paulo Intelligent Monitoring System)
Types of single interventions	– not applicable –
Coding of interventions	– not applicable –
Source of intervention data	data collected by authors
Availability of data on exposure	data access via source
**Methodological approach**	
Empirical approach	descriptive
Use of exposure variation	only variation over time for multiple populations
Method	non-mechanistic model (correlation)
Code availability	none
**Effectiveness assessment**	
Reporting of intervention effectiveness	comparison of outcome values
Measure of effectiveness	growth rate: deaths

– continued on next page –

Interpretation of results	implicitly causal
Reporting of uncertainty	no
Sensitivity analysis	none
Subgroup assessment	none

**Dehning et al. 2020**	
**Study information**	
Author	Dehning J, Zierenberg J, Spitzner FP, Wibral M, Neto JP, Wilczek M, Priesemann V
Title	Inferring Change Points in the Spread of COVID-19 Reveals the Effectiveness of Interventions
Year	2020
Journal	Science
DOI	10.1126/science.abb9789
Date received	2020-04-03
Date accepted	2020-05-12
**Study setting**	
Number of populations included	single
Level of populations included	national (Germany)
Geographic areas covered	Europe
Number of countries covered	single country (Germany)
Study period	start and end date span first epidemic wave
**Outcome**	
Raw outcome	epidemiological population-level outcome (cases)

– continued on next page –

Time resolution of raw outcome	daily
Computed outcome	none
Method to obtain the computed outcome	– not applicable –
Data source	data from publicly available cross-country selections (JHU)
Data availability	data made available by the authors
**Intervention**	
Terminology for interventions	interventions
Terminology for the specific type of non-pharmaceutical interventions	– not applicable –
Exposure types	one combination of interventions (stage 1: event ban, stage 2: school and venue closure, stage 3: contact ban, lockdown)
Types of single interventions	– not applicable –
Coding of interventions	not necessary
Source of intervention data	– could not be evaluated –
Availability of data on exposure	raw data documented in the manuscript
**Methodological approach**	
Empirical approach	parametric
Use of exposure variation	only variation over time for a single population
Method	mechanistic model (compartmental single-population transmission model)
Latent variable functionally linked to intervention	transmission rate
Code availability	publicly available
**Effectiveness assessment**	
Reporting of intervention effectiveness	quantification of change in outcome values

– continued on next page –

Measure of effectiveness	transmission rate
Interpretation of results	implicitly causal
Reporting of uncertainty	yes
Sensitivity analysis	epidemiological parameters varied, model specification varied
Subgroup assessment	none

**Dickson et al. 2020**	
**Study information**	
Author	Dickson MM, Espa G, Giuliani D, Santi F, Savadori L
Title	Assessing the Effect of Containment Measures on the Spatio-Temporal Dynamic of COVID-19 in Italy
Year	2020
Journal	Nonlinear Dynamics
DOI	10.1007/s11071-020-05853-7
Date received	2020-04-27
Date accepted	2020-07-28
**Study setting**	
Number of populations included	multiple
Level of populations included	both national and subnational (Italy and its 107 provinces)
Geographic areas covered	Europe
Number of countries covered	single country (Italy)
Study period	start and end date span first epidemic wave
**Outcome**	
Raw outcome	epidemiological population-level outcome (cases)

– continued on next page –

Time resolution of raw outcome	daily
Computed outcome	measure of epidemic trend (other: autoregressive parameters)
Method to obtain the computed outcome	other (endemic-epidemic regression model based on hhh4 framework with time-varying temporal and spatial autoregressive parameters representing disease spread within and across provinces)
Data source	data from (sub)national authorities
Data availability	data access via source
**Intervention**	
Terminology for interventions	measures
Terminology for the specific type of non-pharmaceutical interventions	containment
Exposure types	multiple combinations of interventions (national quarantine following DPCM of 11 March 2020, closure of non-essential businesses and lockdown on March 25)
Types of single interventions	– not applicable –
Coding of interventions	not necessary
Source of intervention data	– could not be evaluated –
Availability of data on exposure	raw data documented in the manuscript
**Methodological approach**	
Empirical approach	descriptive
Use of exposure variation	only variation over time for multiple populations
Method	description of change over time (description of time course)
Code availability	none
**Effectiveness assessment**	
Reporting of intervention effectiveness	qualitative statement
Measure of effectiveness	autoregressive parameters

– continued on next page –

Interpretation of results	implicitly causal
Reporting of uncertainty	– not applicable –
Sensitivity analysis	none
Subgroup assessment	none

**Dikid et al. 2020**	
**Study information**	
Author	Dikid T, Patel P, Athotra A, Vaisakh TP, Jain S, Covid N
Title	Impact of Nonpharmacological Interventions on COVID-19 Transmission Dynamics in India
Year	2020
Journal	Indian Journal of Public Health
DOI	10.4103/ijph.ijph__510__20
Date received	2020-05-05
Date accepted	2020-05-12
**Study setting**	
Number of populations included	single
Level of populations included	national (India)
Geographic areas covered	Asia
Number of countries covered	single country (India)
Study period	end date in growth phase of wave
**Outcome**	
Raw outcome	epidemiological population-level outcome (cases)
Time resolution of raw outcome	daily

– continued on next page –

Computed outcome	measure of epidemic trend (doubling time), epidemiological parameter (reproduction number)
Method to obtain the computed outcome	statistical estimation of reproduction number (White et al., Wallinga and Teunis), simple computation (doubling time)
Data source	data from (sub)national authorities
Data availability	data access via source
**Intervention**	
Terminology for interventions	interventions
Terminology for the specific type of non-pharmaceutical interventions	non-pharmacological
Exposure types	one combination of interventions (Indian National Lockdown)
Types of single interventions	– not applicable –
Coding of interventions	not necessary
Source of intervention data	government or news websites
Availability of data on exposure	raw data documented in the manuscript
**Methodological approach**	
Empirical approach	descriptive
Use of exposure variation	only variation over time for a single population
Method	description of change over time (comparison of time periods)
Code availability	none
**Effectiveness assessment**	
Reporting of intervention effectiveness	comparison of outcome values
Measure of effectiveness	reproduction number, doubling time
Interpretation of results	implicitly causal
Reporting of uncertainty	yes

– continued on next page –

Sensitivity analysis	none
Subgroup assessment	none

**Djurović 2020**	
**Study information**	
Author	Djurović I
Title	Epidemiological Control Measures and Predicted Number of Infections for SARS-CoV-2 Pandemic: Case Study Serbia March–April 2020
Year	2020
Journal	Heliyon
DOI	10.1016/j.heliyon.2020.e04238
Date received	2020-04-21
Date accepted	2020-06-15
**Study setting**	
Number of populations included	single
Level of populations included	national (Serbia)
Geographic areas covered	Europe
Number of countries covered	single country (Serbia)
Study period	end date at peak of wave
**Outcome**	
Raw outcome	epidemiological population-level outcome (cases)
Time resolution of raw outcome	daily
Computed outcome	none

– continued on next page –

Method to obtain the computed outcome	– not applicable –
Data source	data from (sub)national authorities
Data availability	data access via source
**Intervention**	
Terminology for interventions	measures
Terminology for the specific type of non-pharmaceutical interventions	control
Exposure types	all interventions together (timeline of measures)
Types of single interventions	– not applicable –
Coding of interventions	– not applicable –
Source of intervention data	– not applicable –
Availability of data on exposure	– not applicable –
**Methodological approach**	
Empirical approach	counterfactual
Use of exposure variation	only variation over time for a single population
Method	non-mechanistic model (exponential growth model)
Code availability	none
**Effectiveness assessment**	
Reporting of intervention effectiveness	qualitative statement
Measure of effectiveness	cases
Interpretation of results	implicitly causal
Reporting of uncertainty	– not applicable –

– continued on next page –

Sensitivity analysis	different coding of interventions used (model fit on different time periods with NPIs)
Subgroup assessment	none

**Domenico et al. 2020**	
**Study information**	
Author	Domenico LD, Pullano G, Sabbatini CE, Boëlle PY, Colizza V
Title	Impact of Lockdown on COVID-19 Epidemic in Île-de-France and Possible Exit Strategies
Year	2020
Journal	BMC Medicine
DOI	10.1186/s12916-020-01698-4
Date received	2020-04-28
Date accepted	2020-07-06
**Study setting**	
Number of populations included	single
Level of populations included	subnational (Ile de France)
Geographic areas covered	Europe
Number of countries covered	single country (France)
Study period	start and end date span first epidemic wave
**Outcome**	
Raw outcome	epidemiological population-level outcome (hospitalizations, ICU beds occupation)
Time resolution of raw outcome	daily
Computed outcome	none

– continued on next page –

Method to obtain the computed outcome	– not applicable –
Data source	data from (sub)national authorities
Data availability	data access via source
**Intervention**	
Terminology for interventions	measures
Terminology for the specific type of non-pharmaceutical interventions	social distancing
Exposure types	one combination of interventions (national lockdown per decree of March 18)
Types of single interventions	– not applicable –
Coding of interventions	not necessary
Source of intervention data	government or news websites
Availability of data on exposure	raw data documented in the manuscript
**Methodological approach**	
Empirical approach	counterfactual
Use of exposure variation	only variation over time for a single population
Method	mechanistic model (compartmental single-population transmission model)
Code availability	none
**Effectiveness assessment**	
Reporting of intervention effectiveness	qualitative statement
Measure of effectiveness	ICU beds occupation
Interpretation of results	implicitly causal
Reporting of uncertainty	– not applicable –

– continued on next page –

Sensitivity analysis	epidemiological parameters varied
Subgroup assessment	none

**Donsimoni et al. 2020**	
**Study information**	
Author	Donsimoni JR, Glawion R, Plachter B, Wälde K, Weiser C
Title	Should Contact Bans Have Been Lifted More in Germany?
Year	2020
Journal	CESifo Economic Studies
DOI	10.1093/cesifo/ifaa004
Date received	– could not be evaluated –
Date accepted	2020-07-03(published)
**Study setting**	
Number of populations included	single
Level of populations included	national (Germany)
Geographic areas covered	Europe
Number of countries covered	single country (Germany)
Study period	start and end date span first epidemic wave
**Outcome**	
Raw outcome	epidemiological population-level outcome (cases)
Time resolution of raw outcome	daily
Computed outcome	none
Method to obtain the computed outcome	– not applicable –

– continued on next page –

Data source	data from (sub)national authorities
Data availability	data access via source
**Intervention**	
Terminology for interventions	measures
Terminology for the specific type of non-pharmaceutical interventions	contact ban
Exposure types	one combination of interventions (social contact restrictions (including major public events suspended, schools closed, domestic movements banned))
Types of single interventions	– not applicable –
Coding of interventions	necessary
Source of intervention data	use of externally coded data (OxCGRT)
Availability of data on exposure	access to externally coded data via source
**Methodological approach**	
Empirical approach	counterfactual
Use of exposure variation	only variation over time for a single population
Method	mechanistic model (compartmental single-population transmission model)
Code availability	none
**Effectiveness assessment**	
Reporting of intervention effectiveness	qualitative statement
Measure of effectiveness	cases
Interpretation of results	implicitly causal
Reporting of uncertainty	– not applicable –
Sensitivity analysis	none
Subgroup assessment	none

Du et al. 2020 (Analysis 1)	
**Study information**	
Author	Du Z, Xu X, Wang L, Fox SJ, Cowling BJ, Galvani AP, Meyers LA
Title	Effects of Proactive Social Distancing on COVID-19 Outbreaks in 58 Cities, China
Year	2020
Journal	Emerging Infectious Diseases
DOI	10.3201/eid2609.201932
Date received	– could not be evaluated –
Date accepted	2020-06-09 (published)
**Study setting**	
Number of populations included	multiple
Level of populations included	subnational (58 cities in China outside of Wuhan)
Geographic areas covered	Asia
Number of countries covered	single country (China)
Study period	start and end date span first epidemic wave
**Outcome**	
Raw outcome	epidemiological population-level outcome (cases)
Time resolution of raw outcome	daily
Computed outcome	epidemiological parameter (reproduction number)
Method to obtain the computed outcome	statistical estimation of reproduction number (Cori et al.)
Data source	data from (sub)national authorities
Data availability	data access via source

– continued on next page –

**Intervention**	
Terminology for interventions	measures
Terminology for the specific type of non-pharmaceutical interventions	social distancing
Exposure types	start of interventions (first of many social distancing measures implemented (including venue closure, isolation, travel restrictions))
Types of single interventions	– not applicable –
Coding of interventions	necessary
Source of intervention data	coding done by authors
Availability of data on exposure	coded data made available by the authors
**Methodological approach**	
Empirical approach	descriptive
Use of exposure variation	only variation over time for multiple populations
Method	description of change over time (comparison of time periods)
Code availability	none
**Effectiveness assessment**	
Reporting of intervention effectiveness	quantification of change in outcome values
Measure of effectiveness	reproduction number
Interpretation of results	implicitly causal
Reporting of uncertainty	yes
Sensitivity analysis	none
Subgroup assessment	none

Du et al. 2020 (Analysis 2)	
**Study information**	
Author	Du Z, Xu X, Wang L, Fox SJ, Cowling BJ, Galvani AP, Meyers LA
Title	Effects of Proactive Social Distancing on COVID-19 Outbreaks in 58 Cities, China
Year	2020
Journal	Emerging Infectious Diseases
DOI	10.3201/eid2609.201932
Date received	– could not be evaluated –
Date accepted	2020-06-09 (published)
**Study setting**	
Number of populations included	multiple
Level of populations included	subnational (58 cities in China outside of Wuhan)
Geographic areas covered	Asia
Number of countries covered	single country (China)
Study period	start and end date span first epidemic wave
**Outcome**	
Raw outcome	epidemiological population-level outcome (cases)
Time resolution of raw outcome	daily
Computed outcome	summary statistic (time until reproduction number below 1)
Method to obtain the computed outcome	statistical estimation of reproduction number (Cori et al.)
Data source	data from (sub)national authorities
Data availability	data access via source
**Intervention**	
Terminology for interventions	measures

– continued on next page –

Terminology for the specific type of non-pharmaceutical interventions	social distancing
Exposure types	start of interventions (first of many social distancing measures implemnted (including venue closure, isolation, travel restrictions))
Types of single interventions	– not applicable –
Coding of interventions	necessary
Source of intervention data	coding done by authors
Availability of data on exposure	coded data made available by the authors
**Methodological approach**	
Empirical approach	parametric
Use of exposure variation	only variation between populations
Method	non-mechanistic model (generalized linear model)
Code availability	none
**Effectiveness assessment**	
Reporting of intervention effectiveness	quantification of change in outcome values
Measure of effectiveness	time until reproduction number below 1
Interpretation of results	implicitly causal
Reporting of uncertainty	yes
Sensitivity analysis	none
Subgroup assessment	none

Duhon et al. 2021

**Study information**	
Author	Duhon J, Bragazzi N, Kong JD

– continued on next page –

Title	The Impact of Non-Pharmaceutical Interventions, Demographic, Social, and Climatic Factors on the Initial Growth Rate of COVID-19: A Cross-Country Study
Year	2021
Journal	Science of The Total Environment
DOI	10.1016/j.scitotenv.2020.144325
Date received	2020-09-19
Date accepted	2020-12-05
**Study setting**	
Number of populations included	multiple
Level of populations included	national (– could not be evaluated – (probably many countries, over 100))
Geographic areas covered	Europe, Middle East and Africa, Asia, Oceania, Central and South America, North America
Number of countries covered	multiple countries
Study period	same end date for several populations with diverse epidemic trajectories
**Outcome**	
Raw outcome	epidemiological population-level outcome (cases)
Time resolution of raw outcome	daily
Computed outcome	measure of epidemic trend (growth rate)
Method to obtain the computed outcome	exponential growth model
Data source	data from publicly available cross-country selections (OurWorldInData)
Data availability	data access via source
**Intervention**	
Terminology for interventions	interventions

– continued on next page –

Terminology for the specific type of non-pharmaceutical interventions	non-pharmaceutical
Exposure types	multiple separate interventions (workplace closure, internal movement restrictions)
Types of single interventions	workplace closure, internal movement restrictions
Coding of interventions	necessary
Source of intervention data	use of externally coded data (OxCGRT)
Availability of data on exposure	access to externally coded data via source
**Methodological approach**	
Empirical approach	parametric
Use of exposure variation	both variation over time and between populations
Method	non-mechanistic model (generalized linear model)
Code availability	none
**Effectiveness assessment**	
Reporting of intervention effectiveness	quantification of change in outcome values
Measure of effectiveness	growth rate
Interpretation of results	associative
Reporting of uncertainty	yes
Sensitivity analysis	none
Subgroup assessment	none

Džiugys et al. 2020

**Study information**	
Author	Džiugys A, Bieliūnas M, Skarbalius G, Misiulis E, Navakas R

– continued on next page –

Title	Simplified Model of Covid-19 Epidemic Prognosis under Quarantine and Estimation of Quarantine Effectiveness
Year	2020
Journal	Chaos, Solitons & Fractals
DOI	10.1016/j.chaos.2020.110162
Date received	2020-06-18
Date accepted	2020-07-25
**Study setting**	
Number of populations included	multiple
Level of populations included	national (53 countries worldwide)
Geographic areas covered	Europe, Middle East and Africa, Asia, Oceania, Central and South America, North America
Number of countries covered	multiple countries
Study period	same end date for several populations with diverse epidemic trajectories
**Outcome**	
Raw outcome	epidemiological population-level outcome (cases)
Time resolution of raw outcome	daily
Computed outcome	measure of epidemic trend (growth rate)
Method to obtain the computed outcome	simple computation
Data source	data from publicly available cross-country selections (ECDC)
Data availability	data access via source
**Intervention**	
Terminology for interventions	measures
Terminology for the specific type of non-pharmaceutical interventions	quarantine

– continued on next page –

Exposure types	start of interventions (day of the first intervention leading to lockdown)
Types of single interventions	– not applicable –
Coding of interventions	necessary
Source of intervention data	use of externally coded data (Wikipedia)
Availability of data on exposure	access to externally coded data via source
**Methodological approach**	
Empirical approach	parametric
Use of exposure variation	only variation over time for multiple populations
Method	non-mechanistic model (generalized linear model)
Code availability	none
**Effectiveness assessment**	
Reporting of intervention effectiveness	quantification of change in outcome values
Measure of effectiveness	growth rate
Interpretation of results	associative
Reporting of uncertainty	no
Sensitivity analysis	none
Subgroup assessment	none

Edjoc et al. 2020

**Study information**	
Author	Edjoc R, Atchessi N, Lien A, Smith BA, Gabrani-Juma I, Abalos C, Heisz M
Title	Assessing the Progression of the COVID-19 Pandemic in Canada Using Testing Data and Time-Dependent Reproduction Numbers

– continued on next page –

Year	2020
Journal	Canadian Journal of Public Health
DOI	10.17269/s41997-020-00428-w
Date received	2020-06-23
Date accepted	2020-09-29
**Study setting**	
Number of populations included	multiple
Level of populations included	both national and subnational (Canada)
Geographic areas covered	North America
Number of countries covered	single country (Canada)
Study period	start and end date span first epidemic wave
**Outcome**	
Raw outcome	epidemiological population-level outcome (cases)
Time resolution of raw outcome	daily
Computed outcome	epidemiological parameter (reproduction number)
Method to obtain the computed outcome	statistical estimation of reproduction number (Walinga et al.)
Data source	data from (sub)national authorities
Data availability	data access via source
**Intervention**	
Terminology for interventions	interventions
Terminology for the specific type of non-pharmaceutical interventions	public health
Exposure types	all interventions together (gathering bans, closing non-essential businesses, implementation of telework, closing public venues, closing schools and universities)

– continued on next page –

Types of single interventions	– not applicable –
Coding of interventions	– not applicable –
Source of intervention data	– not applicable –
Availability of data on exposure	– not applicable –
**Methodological approach**	
Empirical approach	descriptive
Use of exposure variation	only variation over time for multiple populations
Method	description of change over time (description of time course)
Code availability	none
**Effectiveness assessment**	
Reporting of intervention effectiveness	qualitative statement
Measure of effectiveness	cases, reproduction number
Interpretation of results	associative
Reporting of uncertainty	– not applicable –
Sensitivity analysis	none
Subgroup assessment	none

Eguchi et al. 2020

**Study information**	
Author	Eguchi A, Yoneoka D, Shi S, Tanoue Y, Kawashima T, Nomura S, Matsuura K, Makiyama K, Ejima K, Gilmour S, Nishiura H, Miyata H
Title	Trend Change of the Transmission Route of COVID-19–Related Symptoms in Japan
Year	2020

– continued on next page –

Journal	Public Health
DOI	10.1016/j.puhe.2020.08.020
Date received	2020-06-16
Date accepted	2020-08-20
**Study setting**	
Number of populations included	single
Level of populations included	national (Japan)
Geographic areas covered	Asia
Number of countries covered	single country (Japan)
Study period	start and end date span first epidemic wave: behavioral
**Outcome**	
Raw outcome	behavioral outcome (survey: contacts with infected individuals and fever symptoms)
Time resolution of raw outcome	daily
Computed outcome	none
Method to obtain the computed outcome	– not applicable –
Data source	data from (sub)national authorities
Data availability	data not accessible
**Intervention**	
Terminology for interventions	– not applicable –
Terminology for the specific type of non-pharmaceutical interventions	emergency declaration
Exposure types	one single intervention (declaration of a state of emergency on April 7 in some prefectures and nationwide on April 16)
Types of single interventions	declaration of a state of emergency

– continued on next page –

Coding of interventions	not necessary
Source of intervention data	government or news websites
Availability of data on exposure	raw data documented in the manuscript
**Methodological approach**	
Empirical approach	descriptive
Use of exposure variation	only variation over time for a single population
Method	description of change over time (description of time course)
Code availability	none
**Effectiveness assessment**	
Reporting of intervention effectiveness	qualitative statement
Measure of effectiveness	contacts with infected individuals, fever symptoms
Interpretation of results	implicitly causal
Reporting of uncertainty	– not applicable –
Sensitivity analysis	none
Subgroup assessment	epidemiological indicators (infection setting)

Fatmi 2020

**Study information**	
Author	Fatmi MR
Title	COVID-19 Impact on Urban Mobility
Year	2020
Journal	Journal of Urban Management
DOI	10.1016/j.jum.2020.08.002

– continued on next page –

Date received	2020-07-22
Date accepted	2020-08-03
**Study setting**	
Number of populations included	single
Level of populations included	subnational (Kelowna region of British Columbia)
Geographic areas covered	North America
Number of countries covered	single country (Canada)
Study period	start and end date span first epidemic wave: behavioral
**Outcome**	
Raw outcome	behavioral outcome (survey: travel activities)
Time resolution of raw outcome	– not applicable –
Computed outcome	none
Method to obtain the computed outcome	– not applicable –
Data source	data collected by authors
Data availability	data not accessible
**Intervention**	
Terminology for interventions	– not applicable –
Terminology for the specific type of non-pharmaceutical interventions	travel restrictions
Exposure types	all interventions together
Types of single interventions	– not applicable –
Coding of interventions	– not applicable –
Source of intervention data	– not applicable –
Availability of data on exposure	– not applicable –
**Methodological approach**	

– continued on next page –

Empirical approach	descriptive
Use of exposure variation	only variation over time for a single population
Method	description of change over time (comparison of time periods)
Code availability	none
**Effectiveness assessment**	
Reporting of intervention effectiveness	comparison of outcome values
Measure of effectiveness	mobility: travel activities
Interpretation of results	associative
Reporting of uncertainty	no
Sensitivity analysis	none
Subgroup assessment	none

Feng et al. 2020

**Study information**	
Author	Feng X, Chen AJ, Wang K, Wang L, Zhang F, Jin Z, Zou L, Wang X, and, and, And
Title	Phase-Adjusted Estimation of the COVID-19 Outbreak in South Korea under Multi-Source Data and Adjustment Measures: A Modelling Study
Year	2020
Journal	Mathematical Biosciences and Engineering
DOI	10.3934/mbe.2020205
Date received	2020-03-30
Date accepted	2020-05-06
**Study setting**	

– continued on next page –

Number of populations included	single
Level of populations included	national (South Korea)
Geographic areas covered	Asia
Number of countries covered	single country (South Korea)
Study period	start and end date span first epidemic wave
**Outcome**	
Raw outcome	epidemiological population-level outcome (cases, deaths, recovered cases)
Time resolution of raw outcome	daily
Computed outcome	epidemiological parameter (reproduction number)
Method to obtain the computed outcome	compartmental transmission model (single-population)
Data source	data from (sub)national authorities
Data availability	data access via source
**Intervention**	
Terminology for interventions	measures
Terminology for the specific type of non-pharmaceutical interventions	lockdown, control
Exposure types	multiple combinations of interventions (second phase: people are encouraged to work at home and reduce the unnecessary gathering activities and so on, third phase: free tests to people in a less crowded manner)
Types of single interventions	– not applicable –
Coding of interventions	not necessary
Source of intervention data	– could not be evaluated –
Availability of data on exposure	raw data documented in the manuscript
**Methodological approach**	
Empirical approach	descriptive

– continued on next page –

Use of exposure variation	only variation over time for a single population
Method	description of change over time (comparison of time periods)
Code availability	none
**Effectiveness assessment**	
Reporting of intervention effectiveness	comparison of outcome values
Measure of effectiveness	reproduction number
Interpretation of results	implicitly causal
Reporting of uncertainty	yes
Sensitivity analysis	epidemiological parameters varied
Subgroup assessment	none

Fernández-Recio 2020

**Study information**	
Author	Fernández-Recio J
Title	Modelling the Evolution of COVID-19 in High-Incidence European Countries and Regions: Estimated Number of Infections and Impact of Past and Future Intervention Measures
Year	2020
Journal	Journal of Clinical Medicine
DOI	10.3390/jcm9061825
Date received	2020-05-18
Date accepted	2020-06-08
**Study setting**	
Number of populations included	multiple

– continued on next page –

Level of populations included	both national and subnational (Spain, Italy, UK, Germany, France, Iceland, La Rioja)
Geographic areas covered	Europe
Number of countries covered	multiple countries
Study period	start and end date span first epidemic wave
**Outcome**	
Raw outcome	epidemiological population-level outcome (cases, deaths)
Time resolution of raw outcome	daily
Computed outcome	none
Method to obtain the computed outcome	– not applicable –
Data source	data from publicly available cross-country selections (ECDC)
Data availability	data access via source
**Intervention**	
Terminology for interventions	measures
Terminology for the specific type of non-pharmaceutical interventions	intervention
Exposure types	one combination of interventions (lockdown (dates inferred from Flaxman study))
Types of single interventions	– not applicable –
Coding of interventions	necessary
Source of intervention data	use of externally coded data (same as in Flaxman et al.)
Availability of data on exposure	coded data made available by the authors
**Methodological approach**	
Empirical approach	parametric
Use of exposure variation	only variation over time for multiple populations

– continued on next page –

Method	mechanistic model (semi-mechanistic Bayesian transmission model)
Latent variable functionally linked to intervention	reproduction number
Code availability	none
**Effectiveness assessment**	
Reporting of intervention effectiveness	quantification of change in outcome values
Measure of effectiveness	reproduction number
Interpretation of results	implicitly causal
Reporting of uncertainty	yes
Sensitivity analysis	none
Subgroup assessment	none

Flaxman et al. 2020

**Study information**	
Author	Flaxman S, Mishra S, Gandy A, Unwin HJ, Mellan TA, Coupland H, Whittaker C, Zhu H, Berah T, Eaton JW, Monod M, Perez-Guzman PN, Schmit N, Cilloni L, Ainslie KE, Baguelin M, Boonyasiri A, Boyd O, Cattarino L, Cooper LV, Cucunubá Z, Cuomo-Dannenburg G, Dighe A, Djaafara B, Dorigatti I, van Elsland SL, FitzJohn RG, Gaythorpe KA, Geidelberg L, Grassly NC, Green WD, Hallett T, Hamlet A, Hinsley W, Jeffrey B, Knock E, Laydon DJ, Nedjati-Gilani G, Nouvellet P, Parag KV, Siveroni I, Thompson HA, Verity R, Volz E, Walters CE, Wang H, Wang Y, Watson OJ, Winskill P, Xi X, Walker PG, Ghani AC, Donnelly CA, Riley S, Vollmer MA, Ferguson NM, Okell LC, And SB
Title	Estimating the Effects of Non-Pharmaceutical Interventions on COVID-19 in Europe

– continued on next page –

Year	2020
Journal	Nature
DOI	10.1038/s41586-020-2405-7
Date received	2020-03-30
Date accepted	2020-05-22
**Study setting**	
Number of populations included	multiple
Level of populations included	national (11 European countries)
Geographic areas covered	Europe
Number of countries covered	multiple countries
Study period	start and end date span first epidemic wave
**Outcome**	
Raw outcome	epidemiological population-level outcome (deaths)
Time resolution of raw outcome	daily
Computed outcome	none
Method to obtain the computed outcome	– not applicable –
Data source	data from publicly available cross-country selections (ECDC)
Data availability	data made available by the authors
**Intervention**	
Terminology for interventions	interventions
Terminology for the specific type of non-pharmaceutical interventions	non-pharmaceutical
Exposure types	multiple separate interventions (lockdown (gathering bans, venue closure), public events banned, primary and secondary school closure, self isolaton, social distancing encouraged)

– continued on next page –

Types of single interventions	– not applicable –
Coding of interventions	necessary
Source of intervention data	coding done by authors
Availability of data on exposure	coded data made available by the authors
**Methodological approach**	
Empirical approach	parametric
Use of exposure variation	both variation over time and between populations
Method	mechanistic model (semi-mechanistic Bayesian transmission model)
Latent variable functionally linked to intervention	reproduction number
Code availability	publicly available
**Effectiveness assessment**	
Reporting of intervention effectiveness	quantification of change in outcome values
Measure of effectiveness	reproduction number
Interpretation of results	implicitly causal
Reporting of uncertainty	yes
Sensitivity analysis	start or end date of study period varied, same analysis with (sub)population(s) excluded, model specification varied, epidemiological parameters varied
Subgroup assessment	none

Frank 2020

**Study information**	
Author	Frank TD

– continued on next page –

Title	COVID-19 Interventions in Some European Countries Induced Bifurcations Stabilizing Low Death States against High Death States: An Eigenvalue Analysis Based on the Order Parameter Concept of Synergetics
Year	2020
Journal	Chaos, Solitons & Fractals
DOI	10.1016/j.chaos.2020.110194
Date received	2020-07-15
Date accepted	2020-08-06
**Study setting**	
Number of populations included	multiple
Level of populations included	national (20 European countries)
Geographic areas covered	Europe
Number of countries covered	multiple countries
Study period	start and end date span first epidemic wave
**Outcome**	
Raw outcome	epidemiological population-level outcome (cases)
Time resolution of raw outcome	daily
Computed outcome	other (order parameter eigenvalue)
Method to obtain the computed outcome	compartmental transmission model (single-population)
Data source	data from publicly available cross-country selections (JHU)
Data availability	data access via source
**Intervention**	
Terminology for interventions	interventions
Terminology for the specific type of non-pharmaceutical interventions	government

– continued on next page –

Exposure types	all interventions together
Types of single interventions	– not applicable –
Coding of interventions	– not applicable –
Source of intervention data	– not applicable –
Availability of data on exposure	– not applicable –
**Methodological approach**	
Empirical approach	descriptive
Use of exposure variation	only variation over time for multiple populations
Method	description of change over time (comparison of time periods)
Code availability	none
**Effectiveness assessment**	
Reporting of intervention effectiveness	qualitative statement
Measure of effectiveness	order parameter eigenvalue
Interpretation of results	implicitly causal
Reporting of uncertainty	– not applicable –
Sensitivity analysis	none
Subgroup assessment	none

Friedrich et al. 2020

**Study information**	
Author	Friedrich F, Ongaratto R, Scotta MC, Veras TN, Stein RT, Lumertz MS, Jones MH, Comaru T, Pinto LA
Title	Early Impact of Social Distancing in Response to Coronavirus Disease 2019 on Hospitalizations for Acute Bronchiolitis in Infants in Brazil

– continued on next page –

Year	2020
Journal	Clinical Infectious Diseases
DOI	10.1093/cid/ciaa1458
Date received	2020-08-12
Date accepted	2020-09-22
**Study setting**	
Number of populations included	multiple
Level of populations included	both national and subnational (Brazil and 5 of its macro-regions)
Geographic areas covered	Central and South America
Number of countries covered	single country (Brazil)
Study period	end date in growth phase of wave
**Outcome**	
Raw outcome	epidemiological population-level outcome (surrogate disease: hospitalizations with acute bronchiolitis in infants)
Time resolution of raw outcome	monthly
Computed outcome	none
Method to obtain the computed outcome	– not applicable –
Data source	data from (sub)national authorities
Data availability	data access via source
**Intervention**	
Terminology for interventions	measures
Terminology for the specific type of non-pharmaceutical interventions	social distancing
Exposure types	all interventions together
Types of single interventions	– not applicable –

– continued on next page –

Coding of interventions	– not applicable –
Source of intervention data	– not applicable –
Availability of data on exposure	– not applicable –
**Methodological approach**	
Empirical approach	descriptive
Use of exposure variation	only variation over time for multiple populations
Method	description of change over time (comparison of time periods)
Code availability	none
**Effectiveness assessment**	
Reporting of intervention effectiveness	quantification of change in outcome values
Measure of effectiveness	hospitalizations: acute bronchiolitis in infants
Interpretation of results	implicitly causal
Reporting of uncertainty	yes
Sensitivity analysis	none
Subgroup assessment	none

Friedson et al. 2020 (Analysis 1)

**Study information**	
Author	Friedson AI, McNichols D, Sabia JJ, Dave D
Title	Shelter-in-Place Orders and Public Health: Evidence from California during the COVID-19 Pandemic
Year	2020

– continued on next page –

Journal	Journal of Policy Analysis and Management
DOI	10.1002/pam.22267
Date received	– could not be evaluated –
Date accepted	2020-12-11
**Study setting**	
Number of populations included	single
Level of populations included	subnational (California)
Geographic areas covered	North America
Number of countries covered	single country (California)
Study period	end date in growth phase of wave
**Outcome**	
Raw outcome	epidemiological population-level outcome (cases, deaths), behavioral outcome (mobility: shelter-in-place index)
Time resolution of raw outcome	daily
Computed outcome	none
Method to obtain the computed outcome	– not applicable –
Data source	data from public media outlets (New York Times: cases), mobility data from corporate organizations (SafeGraph: mobility)
Data availability	data access via source
**Intervention**	
Terminology for interventions	– not applicable –
Terminology for the specific type of non-pharmaceutical interventions	shelter-in-place order
Exposure types	one single intervention (stay-at-home order)
Types of single interventions	stay-at-home order

– continued on next page –

Coding of interventions	not necessary
Source of intervention data	– could not be evaluated –
Availability of data on exposure	raw data documented in the manuscript
**Methodological approach**	
Empirical approach	counterfactual
Use of exposure variation	only variation over time for a single population
Method	synthetic controls
Code availability	none
**Effectiveness assessment**	
Reporting of intervention effectiveness	quantification of change in outcome values
Measure of effectiveness	cases, deaths, mobility: shelter-in-place index
Interpretation of results	explicitly causal
Reporting of uncertainty	yes
Sensitivity analysis	same analysis with (sub)population(s) excluded, model specification varied
Subgroup assessment	none

**Friedson et al. 2020 (Analysis 2)**	
**Study information**	
Author	Friedson AI, McNichols D, Sabia JJ, Dave D
Title	Shelter-in-Place Orders and Public Health: Evidence from California during the COVID-19 Pandemic
Year	2020
Journal	Journal of Policy Analysis and Management
DOI	10.1002/pam.22267

– continued on next page –

Date received	– could not be evaluated –
Date accepted	2020-12-11
**Study setting**	
Number of populations included	multiple
Level of populations included	both national and subnational (US states)
Geographic areas covered	North America
Number of countries covered	single country (US)
Study period	end date in growth phase of wave
**Outcome**	
Raw outcome	epidemiological population-level outcome (cases, deaths)
Time resolution of raw outcome	daily
Computed outcome	none
Method to obtain the computed outcome	– not applicable –
Data source	data from public media outlets (New York Times)
Data availability	data access via source
**Intervention**	
Terminology for interventions	– not applicable –
Terminology for the specific type of non-pharmaceutical interventions	shelter-in-place order
Exposure types	one single intervention (stay-at-home order)
Types of single interventions	stay-at-home order
Coding of interventions	not necessary
Source of intervention data	– could not be evaluated –
Availability of data on exposure	raw data documented in the manuscript
**Methodological approach**	

– continued on next page –

Empirical approach	parametric
Use of exposure variation	only variation over time for a single population
Method	non-mechanistic model (generalized linear model)
Code availability	none
**Effectiveness assessment**	
Reporting of intervention effectiveness	quantification of change in outcome values
Measure of effectiveness	cases, deaths
Interpretation of results	explicitly causal
Reporting of uncertainty	yes
Sensitivity analysis	same analysis with (sub)population(s) excluded, model specification varied
Subgroup assessment	none

Gallaway et al. 2020

**Study information**	
Author	Gallaway MS, Rigler J, Robinson S, Herrick K, Livar E, Komatsu KK, Brady S, Cunico J, Christ CM
Title	Trends in COVID-19 Incidence after Implementation of Mitigation Measures —Arizona, January 22–August 7, 2020
Year	2020
Journal	Morbidity and Mortality Weekly Report
DOI	10.15585/mmwr.mm6940e3
Date received	– could not be evaluated –
Date accepted	2020-08-07 (published)
**Study setting**	

– continued on next page –

Number of populations included	single
Level of populations included	subnational (Arizona)
Geographic areas covered	North America
Number of countries covered	single country (US)
Study period	start and end date span first epidemic wave
**Outcome**	
Raw outcome	epidemiological population-level outcome (cases)
Time resolution of raw outcome	daily
Computed outcome	none
Method to obtain the computed outcome	– not applicable –
Data source	data from (sub)national authorities
Data availability	data access via source
**Intervention**	
Terminology for interventions	measures
Terminology for the specific type of non-pharmaceutical interventions	mitigation
Exposure types	all interventions together (timeline of measures)
Types of single interventions	– not applicable –
Coding of interventions	– not applicable –
Source of intervention data	– not applicable –
Availability of data on exposure	– not applicable –
**Methodological approach**	
Empirical approach	descriptive
Use of exposure variation	only variation over time for a single population
Method	description of change over time (comparison of time periods)

– continued on next page –

Code availability	none
**Effectiveness assessment**	
Reporting of intervention effectiveness	comparison of outcome values
Measure of effectiveness	cases
Interpretation of results	associative
Reporting of uncertainty	no
Sensitivity analysis	none
Subgroup assessment	none

Gao et al. 2020 (Analysis 1)

**Study information**	
Author	Gao S, Rao J, Kang Y, Liang Y, Kruse J, Dopfer D, Sethi AK, Reyes JF, Yandell BS, Patz JA
Title	Association of Mobile Phone Location Data Indications of Travel and Stay-at-Home Mandates with COVID-19 Infection Rates in the US
Year	2020
Journal	JAMA Network Open
DOI	10.1001/jamanetworkopen.2020.20485
Date received	– could not be evaluated –
Date accepted	2020-07-31
**Study setting**	
Number of populations included	multiple
Level of populations included	both national and subnational (US and all states)

– continued on next page –

Geographic areas covered	North America
Number of countries covered	single country (US)
Study period	same end date for several populations with diverse epidemic trajectories
**Outcome**	
Raw outcome	epidemiological population-level outcome (cases)
Time resolution of raw outcome	daily
Computed outcome	measure of epidemic trend (doubling time)
Method to obtain the computed outcome	exponential growth model
Data source	data from publicly available open-source projects (Corona Data Scraper: cases), data from (sub)national authorities (health authority: cases)
Data availability	data access via source
**Intervention**	
Terminology for interventions	measures
Terminology for the specific type of non-pharmaceutical interventions	non-pharmacological
Exposure types	one single intervention (stay-at-home order)
Types of single interventions	stay-at-home order
Coding of interventions	necessary
Source of intervention data	coding done by authors
Availability of data on exposure	coded data made available by the authors
**Methodological approach**	
Empirical approach	descriptive
Use of exposure variation	only variation over time for multiple populations
Method	description of change over time (comparison of time periods)
Code availability	none

– continued on next page –

**Effectiveness assessment**	
Reporting of intervention effectiveness	quantification of change in outcome values
Measure of effectiveness	doubling time
Interpretation of results	associative
Reporting of uncertainty	yes
Sensitivity analysis	model specification varied
Subgroup assessment	none

Gao et al. 2020 (Analysis 2)

**Study information**	
Author	Gao S, Rao J, Kang Y, Liang Y, Kruse J, Dopfer D, Sethi AK, Reyes JF, Yandell BS, Patz JA
Title	Association of Mobile Phone Location Data Indications of Travel and Stay-at-Home Mandates with COVID-19 Infection Rates in the US
Year	2020
Journal	JAMA Network Open
DOI	10.1001/jamanetworkopen.2020.20485
Date received	– could not be evaluated –
Date accepted	2020-07-31
**Study setting**	
Number of populations included	multiple
Level of populations included	both national and subnational (US and all states)
Geographic areas covered	North America
Number of countries covered	single country (US)

– continued on next page –

Study period	start and end date span first epidemic wave: behavioral
**Outcome**	
Raw outcome	behavioral outcome (mobility: number of trips between and within counties relative to baseline)
Time resolution of raw outcome	daily
Computed outcome	none
Method to obtain the computed outcome	– not applicable –
Data source	mobility data from corporate organizations (Descartes Labs (mobility: travel distances), Safegraph (mobility: home dwell time))
Data availability	data access via source
**Intervention**	
Terminology for interventions	measures
Terminology for the specific type of non-pharmaceutical interventions	non-pharmacological
Exposure types	one single intervention (stay-at-home order)
Types of single interventions	stay-at-home order
Coding of interventions	necessary
Source of intervention data	coding done by authors
Availability of data on exposure	coded data made available by the authors
**Methodological approach**	
Empirical approach	descriptive
Use of exposure variation	only variation over time for multiple populations
Method	description of change over time (comparison of time periods)
Code availability	none
**Effectiveness assessment**	

– continued on next page –

Reporting of intervention effectiveness	quantification of change in outcome values
Measure of effectiveness	mobility: travel distance & home dwell time
Interpretation of results	associative
Reporting of uncertainty	yes
Sensitivity analysis	none
Subgroup assessment	none

Gatto et al. 2020

**Study information**	
Author	Gatto M, Bertuzzo E, Mari L, Miccoli S, Carraro L, Casagrandi R, Rinaldo A
Title	Spread and Dynamics of the COVID-19 Epidemic in Italy: Effects of Emergency Containment Measures
Year	2020
Journal	Proceedings of the National Academy of Sciences
DOI	10.1073/pnas.2004978117
Date received	2020-03-26 (sent for review)
Date accepted	2020-04-06
**Study setting**	
Number of populations included	multiple
Level of populations included	both national and subnational (Italy and 107 provinces)
Geographic areas covered	Europe
Number of countries covered	single country (Italy)

– continued on next page –

Study period	end date at peak of wave
**Outcome**	
Raw outcome	epidemiological population-level outcome (hospitalizations, deaths)
Time resolution of raw outcome	daily
Computed outcome	none
Method to obtain the computed outcome	– not applicable –
Data source	data from (sub)national authorities
Data availability	data access via source
**Intervention**	
Terminology for interventions	measures
Terminology for the specific type of non-pharmaceutical interventions	containment
Exposure types	multiple combinations of interventions (two sets of measures adopted at unspecific dates and to a varying extent in different subnational regions)
Types of single interventions	– not applicable –
Coding of interventions	not necessary
Source of intervention data	government or news websites
Availability of data on exposure	raw data documented in the manuscript
**Methodological approach**	
Empirical approach	parametric
Use of exposure variation	both variation over time and between populations
Method	mechanistic model (compartmental single-population transmission model)
Latent variable functionally linked to intervention	transmission rate
Code availability	none

– continued on next page –

**Effectiveness assessment**	
Reporting of intervention effectiveness	quantification of change in outcome values
Measure of effectiveness	transmission rate
Interpretation of results	implicitly causal
Reporting of uncertainty	yes
Sensitivity analysis	none
Subgroup assessment	none

Ge et al. 2020

**Study information**	
Author	Ge J, He D, Lin Z, Zhu H, Zhuang Z
Title	Four-Tier Response System and Spatial Propagation of COVID-19 in China by a Network Model
Year	2020
Journal	Mathematical Biosciences
DOI	10.1016/j.mbs.2020.108484
Date received	2020-08-16
Date accepted	2020-09-28
**Study setting**	
Number of populations included	multiple
Level of populations included	subnational (Wuhan and 18 other cities/regions)
Geographic areas covered	Asia
Number of countries covered	single country (China)

– continued on next page –

Study period	start and end date span first epidemic wave
**Outcome**	
Raw outcome	epidemiological population-level outcome (cases)
Time resolution of raw outcome	daily
Computed outcome	none
Method to obtain the computed outcome	– not applicable –
Data source	– could not be evaluated –
Data availability	data not accessible
**Intervention**	
Terminology for interventions	measures
Terminology for the specific type of non-pharmaceutical interventions	social distancing
Exposure types	phases of interventions (different response levels in Chinas response plan)
Types of single interventions	– not applicable –
Coding of interventions	not necessary
Source of intervention data	government or news websites
Availability of data on exposure	raw data documented in the manuscript
**Methodological approach**	
Empirical approach	parametric
Use of exposure variation	both variation over time and between populations
Method	mechanistic model (compartmental metapopulation transmission model)
Latent variable functionally linked to intervention	transmission rate
Code availability	none
**Effectiveness assessment**	

– continued on next page –

Reporting of intervention effectiveness	quantification of change in outcome values
Measure of effectiveness	transmission rate
Interpretation of results	implicitly causal
Reporting of uncertainty	yes
Sensitivity analysis	none
Subgroup assessment	none

Ghosal et al. 2020

**Study information**	
Author	Ghosal S, Bhattacharyya R, Majumder M
Title	Impact of Complete Lockdown on Total Infection and Death Rates: A Hierarchical Cluster Analysis
Year	2020
Journal	Diabetes & Metabolic Syndrome: Clinical Research & Reviews
DOI	10.1016/j.dsx.2020.05.026
Date received	2020-05-08
Date accepted	2020-05-11
**Study setting**	
Number of populations included	multiple
Level of populations included	national (Spain, Germany, Italy, UK, France, Belgium, Austria, New Zealand, India, Hungary, Poland, Malysia)
Geographic areas covered	Asia, Europe, Oceania
Number of countries covered	multiple countries

– continued on next page –

Study period	end date could not be evaluated
**Outcome**	
Raw outcome	epidemiological population-level outcome (growth rate, growth rate: deaths)
Time resolution of raw outcome	daily
Computed outcome	none
Method to obtain the computed outcome	– not applicable –
Data source	data from publicly available cross-country selections (Worldometer)
Data availability	data access via source
**Intervention**	
Terminology for interventions	strategies
Terminology for the specific type of non-pharmaceutical interventions	preventive
Exposure types	one combination of interventions (lockdown)
Types of single interventions	– not applicable –
Coding of interventions	necessary
Source of intervention data	coding done by authors
Availability of data on exposure	coded data made available by the authors
**Methodological approach**	
Empirical approach	descriptive
Use of exposure variation	only variation over time for multiple populations
Method	description of change over time (description of time course)
Code availability	none
**Effectiveness assessment**	
Reporting of intervention effectiveness	qualitative statement

– continued on next page –

Measure of effectiveness	growth rate, growth rate: deaths
Interpretation of results	associative
Reporting of uncertainty	– not applicable –
Sensitivity analysis	none
Subgroup assessment	none

Gill et al. 2020 (Analysis 1)

**Study information**	
Author	Gill BS, Jayaraj VJ, Singh S, Ghazali SM, Cheong YL, Iderus NH, Sundram BM, Aris TB, Ibrahim HM, Hong BH, Labadin J
Title	Modelling the Effectiveness of Epidemic Control Measures in Preventing the Transmission of COVID-19 in Malaysia
Year	2020
Journal	International Journal of Environmental Research and Public Health
DOI	10.3390/ijerph17155509
Date received	2020-04-28
Date accepted	2020-07-02
**Study setting**	
Number of populations included	single
Level of populations included	national (Malaysia)
Geographic areas covered	Asia
Number of countries covered	single country (Malaysia)
Study period	start and end date span first epidemic wave

– continued on next page –

**Outcome**	
Raw outcome	epidemiological population-level outcome (active cases)
Time resolution of raw outcome	daily
Computed outcome	none
Method to obtain the computed outcome	– not applicable –
Data source	data from (sub)national authorities
Data availability	data access via source
**Intervention**	
Terminology for interventions	measures
Terminology for the specific type of non-pharmaceutical interventions	control
Exposure types	one combination of interventions (movement control order decreed by the Malaysian government on March 18)
Types of single interventions	– not applicable –
Coding of interventions	not necessary
Source of intervention data	– could not be evaluated –
Availability of data on exposure	raw data documented in the manuscript
**Methodological approach**	
Empirical approach	counterfactual
Use of exposure variation	only variation over time for a single population
Method	mechanistic model (compartmental single-population transmission model)
Code availability	none
**Effectiveness assessment**	
Reporting of intervention effectiveness	quantification of change in outcome values

– continued on next page –

Measure of effectiveness	active cases
Interpretation of results	implicitly causal
Reporting of uncertainty	no
Sensitivity analysis	none
Subgroup assessment	none

**Gill et al. 2020 (Analysis 2)**	
**Study information**	
Author	Gill BS, Jayaraj VJ, Singh S, Ghazali SM, Cheong YL, Iderus NH, Sundram BM, Aris TB, Ibrahim HM, Hong BH, Labadin J
Title	Modelling the Effectiveness of Epidemic Control Measures in Preventing the Transmission of COVID-19 in Malaysia
Year	2020
Journal	International Journal of Environmental Research and Public Health
DOI	10.3390/ijerph17155509
Date received	2020-04-28
Date accepted	2020-07-02
**Study setting**	
Number of populations included	single
Level of populations included	national (Malaysia)
Geographic areas covered	Asia
Number of countries covered	single country (Malaysia)
Study period	start and end date span first epidemic wave
**Outcome**	
Raw outcome	epidemiological population-level outcome (active cases)

– continued on next page –

Time resolution of raw outcome	daily
Computed outcome	epidemiological parameter (contact rate)
Method to obtain the computed outcome	compartmental transmission model (single-population)
Data source	data from (sub)national authorities
Data availability	data access via source
**Intervention**	
Terminology for interventions	measures
Terminology for the specific type of non-pharmaceutical interventions	control
Exposure types	one combination of interventions (movement control order decreed by the Malaysian government on March 18)
Types of single interventions	– not applicable –
Coding of interventions	not necessary
Source of intervention data	– could not be evaluated –
Availability of data on exposure	raw data documented in the manuscript
**Methodological approach**	
Empirical approach	descriptive
Use of exposure variation	only variation over time for a single population
Method	description of change over time (comparison of time periods)
Code availability	none
**Effectiveness assessment**	
Reporting of intervention effectiveness	comparison of outcome values
Measure of effectiveness	contact rate
Interpretation of results	implicitly causal

– continued on next page –

Reporting of uncertainty	no
Sensitivity analysis	none
Subgroup assessment	none

**Gregori et al. 2020**	
**Study information**	
Author	Gregori D, Azzolina D, Lanera C, Prosepe I, Destro N, Lorenzoni G, Berchialla P
Title	A First Estimation of the Impact of Public Health Actions against COVID-19 in Veneto (Italy)
Year	2020
Journal	Journal of Epidemiology and Community Health
DOI	10.1136/jech-2020-214209
Date received	2020-03-28
Date accepted	2020-04-21
**Study setting**	
Number of populations included	single
Level of populations included	subnational (Venice (Italy))
Geographic areas covered	Europe
Number of countries covered	single country (Italy)
Study period	end date at peak of wave
**Outcome**	
Raw outcome	epidemiological population-level outcome (hospitalizations)
Time resolution of raw outcome	daily

– continued on next page –

Computed outcome	none
Method to obtain the computed outcome	– not applicable –
Data source	data from (sub)national authorities
Data availability	data access via source
**Intervention**	
Terminology for interventions	interventions
Terminology for the specific type of non-pharmaceutical interventions	public health
Exposure types	one combination of interventions (change point 17 days after the first decree law that established a lockdown)
Types of single interventions	– not applicable –
Coding of interventions	not necessary
Source of intervention data	government or news websites
Availability of data on exposure	raw data documented in the manuscript
**Methodological approach**	
Empirical approach	counterfactual
Use of exposure variation	only variation over time for a single population
Method	non-mechanistic model (other: generalized additive model)
Code availability	none
**Effectiveness assessment**	
Reporting of intervention effectiveness	quantification of change in outcome values
Measure of effectiveness	hospitalizations
Interpretation of results	implicitly causal
Reporting of uncertainty	yes

– continued on next page –

Sensitivity analysis	none
Subgroup assessment	none

**Guirao 2020**	
**Study information**	
Author	Guirao A
Title	The COVID-19 Outbreak in Spain. A Simple Dynamics Model, Some Lessons, and a Theoretical Framework for Control Response
Year	2020
Journal	Infectious Disease Modelling
DOI	10.1016/j.idm.2020.08.010
Date received	2020-07-02
Date accepted	2020-08-21
**Study setting**	
Number of populations included	multiple
Level of populations included	national (Spain, Italy, Germany)
Geographic areas covered	Europe
Number of countries covered	multiple countries
Study period	start and end date span first epidemic wave
**Outcome**	
Raw outcome	epidemiological population-level outcome (cases)
Time resolution of raw outcome	daily
Computed outcome	epidemiological parameter (reproduction number)

– continued on next page –

Method to obtain the computed outcome	compartmental transmission model (single-population)
Data source	data from (sub)national authorities (Spain, Italy, Germany), data from publicly available cross-country selections (WHO: Italy, Germany)
Data availability	data access via source
**Intervention**	
Terminology for interventions	measures
Terminology for the specific type of non-pharmaceutical interventions	control
Exposure types	one combination of interventions (state decrees establishing a system of work-from-home and the mandatory closure of non-emergency businesses and educational institutions throughout the state)
Types of single interventions	– not applicable –
Coding of interventions	not necessary
Source of intervention data	– could not be evaluated –
Availability of data on exposure	raw data documented in the manuscript
**Methodological approach**	
Empirical approach	descriptive
Use of exposure variation	only variation over time for multiple populations
Method	description of change over time (comparison of time periods)
Code availability	none
**Effectiveness assessment**	
Reporting of intervention effectiveness	comparison of outcome values
Measure of effectiveness	reproduction number
Interpretation of results	implicitly causal
Reporting of uncertainty	yes

– continued on next page –

Sensitivity analysis	none
Subgroup assessment	none

**Guo & Xiao 2020**	
**Study information**	
Author	Guo Z, Xiao D
Title	Analysis and Prediction of the Coronavirus Disease Epidemic in China Based on an Individual-Based Model
Year	2020
Journal	Scientific Reports
DOI	10.1038/s41598-020-76969-4
Date received	2020-04-06
Date accepted	2020-10-30
**Study setting**	
Number of populations included	single
Level of populations included	national (China)
Geographic areas covered	Asia
Number of countries covered	single country (China)
Study period	start and end date span first epidemic wave
**Outcome**	
Raw outcome	epidemiological population-level outcome (cases)
Time resolution of raw outcome	daily
Computed outcome	none

– continued on next page –

Method to obtain the computed outcome	– not applicable –
Data source	data from (sub)national authorities
Data availability	data made available by the authors
**Intervention**	
Terminology for interventions	measures
Terminology for the specific type of non-pharmaceutical interventions	prevention, control
Exposure types	one single intervention (traffic control restrictions to and from Hubei)
Types of single interventions	internal movement restrictions
Coding of interventions	not necessary
Source of intervention data	government or news websites
Availability of data on exposure	raw data documented in the manuscript
**Methodological approach**	
Empirical approach	counterfactual
Use of exposure variation	only variation over time for a single population
Method	mechanistic model (individual-based transmission model)
Code availability	none
**Effectiveness assessment**	
Reporting of intervention effectiveness	quantification of change in outcome values
Measure of effectiveness	cases
Interpretation of results	implicitly causal
Reporting of uncertainty	no
Sensitivity analysis	epidemiological parameters varied
Subgroup assessment	none

Gupta et al. 2021 (Analysis 1)	
**Study information**	
Author	Gupta M, Mohanta SS, Rao A, Parameswaran GG, Agarwal M, Arora M, Mazumder A, Lohiya A, Behera P, Bansal A, Kumar R, Meena VP, Tiwari P, Mohan A, Bhatnagar S
Title	Transmission Dynamics of the COVID-19 Epidemic in India and Modeling Optimal Lockdown Exit Strategies
Year	2021
Journal	International Journal of Infectious Diseases
DOI	10.1016/j.ijid.2020.11.206
Date received	2020-07-01
Date accepted	2020-11-28
**Study setting**	
Number of populations included	single
Level of populations included	national (India)
Geographic areas covered	Asia
Number of countries covered	single country (India)
Study period	end date in growth phase of wave
**Outcome**	
Raw outcome	epidemiological population-level outcome (cases)
Time resolution of raw outcome	daily
Computed outcome	epidemiological parameter (reproduction number)
Method to obtain the computed outcome	back-projection (infections), statistical estimation of reproduction number (White et al., Walinga et al., Cori et al.)
Data source	data from publicly available open-source projects (covid19India.org)

– continued on next page –

Data availability	data made available by the authors
**Intervention**	
Terminology for interventions	measures
Terminology for the specific type of non-pharmaceutical interventions	control
Exposure types	one combination of interventions (Indian National Lockdown)
Types of single interventions	– not applicable –
Coding of interventions	not necessary
Source of intervention data	– could not be evaluated –
Availability of data on exposure	raw data documented in the manuscript
**Methodological approach**	
Empirical approach	descriptive
Use of exposure variation	only variation over time for a single population
Method	description of change over time (description of time course)
Code availability	publicly available
**Effectiveness assessment**	
Reporting of intervention effectiveness	comparison of outcome values
Measure of effectiveness	reproduction number
Interpretation of results	implicitly causal
Reporting of uncertainty	yes
Sensitivity analysis	none
Subgroup assessment	none

Gupta et al. 2021 (Analysis 2)	
**Study information**	
Author	Gupta M, Mohanta SS, Rao A, Parameswaran GG, Agarwal M, Arora M, Mazumder A, Lohiya A, Behera P, Bansal A, Kumar R, Meena VP, Tiwari P, Mohan A, Bhatnagar S
Title	Transmission Dynamics of the COVID-19 Epidemic in India and Modeling Optimal Lockdown Exit Strategies
Year	2021
Journal	International Journal of Infectious Diseases
DOI	10.1016/j.ijid.2020.11.206
Date received	2020-07-01
Date accepted	2020-11-28
**Study setting**	
Number of populations included	single
Level of populations included	national (India)
Geographic areas covered	Asia
Number of countries covered	single country (India)
Study period	start and end date span first epidemic wave: behavioral
**Outcome**	
Raw outcome	behavioral outcome (mobility: time spent in various locations)
Time resolution of raw outcome	daily
Computed outcome	none
Method to obtain the computed outcome	– not applicable –
Data source	mobility data from corporate organizations (Google)
Data availability	data made available by the authors
**Intervention**	

– continued on next page –

Terminology for interventions	measures
Terminology for the specific type of non-pharmaceutical interventions	control
Exposure types	one combination of interventions (Indian National Lockdown)
Types of single interventions	– not applicable –
Coding of interventions	not necessary
Source of intervention data	– could not be evaluated –
Availability of data on exposure	raw data documented in the manuscript
**Methodological approach**	
Empirical approach	descriptive
Use of exposure variation	only variation over time for a single population
Method	description of change over time (description of time course)
Code availability	publicly available
**Effectiveness assessment**	
Reporting of intervention effectiveness	qualitative statement
Measure of effectiveness	mobility: time spent in various locations
Interpretation of results	implicitly causal
Reporting of uncertainty	– not applicable –
Sensitivity analysis	none
Subgroup assessment	none

Hao et al. 2020

**Study information**	

– continued on next page –

Author	Hao X, Cheng S, Wu D, Wu T, Lin X, Wang C
Title	Reconstruction of the Full Transmission Dynamics of COVID-19 in Wuhan
Year	2020
Journal	Nature
DOI	10.1038/s41586-020-2554-8
Date received	2020-04-14
Date accepted	2020-07-10
**Study setting**	
Number of populations included	single
Level of populations included	subnational (Wuhan (China))
Geographic areas covered	Asia
Number of countries covered	single country (China)
Study period	start and end date span first epidemic wave
**Outcome**	
Raw outcome	epidemiological individual-level outcome (individual cases)
Time resolution of raw outcome	daily
Computed outcome	epidemiological parameter (reproduction number)
Method to obtain the computed outcome	compartmental transmission model (single-population)
Data source	data from (sub)national authorities
Data availability	data made available by the authors
**Intervention**	
Terminology for interventions	interventions
Terminology for the specific type of non-pharmaceutical interventions	non-pharmaceutical

– continued on next page –

Exposure types	multiple combinations of interventions (Wuhan City Lockdown, centralized isolation and quarantine, community screening)
Types of single interventions	– not applicable –
Coding of interventions	not necessary
Source of intervention data	another study (Pan et al.)
Availability of data on exposure	raw data documented in the manuscript
**Methodological approach**	
Empirical approach	descriptive
Use of exposure variation	only variation over time for a single population
Method	description of change over time (comparison of time periods)
Code availability	publicly available
**Effectiveness assessment**	
Reporting of intervention effectiveness	quantification of change in outcome values
Measure of effectiveness	reproduction number, infections
Interpretation of results	implicitly causal
Reporting of uncertainty	yes
Sensitivity analysis	different or modified outcome used (smoothing of outliers), epidemiological parameters varied
Subgroup assessment	none

Haug et al. 2020

**Study information**	
Author	Haug N, Geyrhofer L, Londei A, Dervic E, Desvars-Larrive A, Loreto V, Pinior B, Thurner S, Klimek P

– continued on next page –

Title	Ranking the Effectiveness of Worldwide COVID-19 Government Interventions
Year	2020
Journal	Nature Human Behaviour
DOI	10.1038/s41562-020-01009-0
Date received	2020-10-15
Date accepted	2020-10-28
**Study setting**	
Number of populations included	multiple
Level of populations included	both national and subnational (79 territories around the globe)
Geographic areas covered	Europe, Middle East and Africa, Asia, Oceania, Central and South America, North America
Number of countries covered	multiple countries
Study period	same end date for several populations with diverse epidemic trajectories
**Outcome**	
Raw outcome	epidemiological population-level outcome (cases)
Time resolution of raw outcome	daily
Computed outcome	epidemiological parameter (reproduction number)
Method to obtain the computed outcome	statistical estimation of reproduction number (Cori et al.)
Data source	data from publicly available cross-country selections (JHU)
Data availability	data made available by the authors
**Intervention**	
Terminology for interventions	interventions
Terminology for the specific type of non-pharmaceutical interventions	non-pharmaceutical

– continued on next page –

Exposure types	multiple separate interventions (large list of hierarchically coded NPIs)
Types of single interventions	stay-at-home order, school closure, workplace closure, venue closure, declaration of a state of emergency, bans of large gatherings, bans of small gatherings, internal movement restrictions, public transport closure, international travel restrictions, mask mandate
Coding of interventions	necessary
Source of intervention data	use of externally coded data (Complexity Science Hub COVID-19 Control Strategies List)
Availability of data on exposure	coded data made available by the authors
**Methodological approach**	
Empirical approach	parametric
Use of exposure variation	both variation over time and between populations
Method	non-mechanistic model (other: several machine learning models of different complexity)
Code availability	publicly available
**Effectiveness assessment**	
Reporting of intervention effectiveness	quantification of change in outcome values
Measure of effectiveness	reproduction number
Interpretation of results	implicitly causal
Reporting of uncertainty	yes
Sensitivity analysis	different coding of interventions used (validation with other NPI datasets, implementation date varied)
Subgroup assessment	none

**He et al. 2020a**	
**Study information**	
Author	He J, Chen G, Jiang Y, Jin R, Shortridge A, Agusti S, He M, Wu J, Duarte CM, Christakos G
Title	Comparative Infection Modeling and Control of COVID-19 Transmission Patterns in China, South Korea, Italy and Iran
Year	2020
Journal	Science of The Total Environment
DOI	10.1016/j.scitotenv.2020.141447
Date received	2020-07-05
Date accepted	2020-08-01
**Study setting**	
Number of populations included	multiple
Level of populations included	both national and subnational (Chinese regions (Zhejiang, Guangdong, Xinjiang), South Korea, Italy, Iran)
Geographic areas covered	Asia, Europe, Middle East and Africa
Number of countries covered	multiple countries
Study period	start and end date span first epidemic wave
**Outcome**	
Raw outcome	epidemiological population-level outcome (cases, deaths, recovered cases)
Time resolution of raw outcome	daily
Computed outcome	none
Method to obtain the computed outcome	– not applicable –
Data source	data from (sub)national authorities
Data availability	data access via source
**Intervention**	

– continued on next page –

Terminology for interventions	measures
Terminology for the specific type of non-pharmaceutical interventions	control
Exposure types	all interventions together (timeline of measures)
Types of single interventions	– not applicable –
Coding of interventions	– not applicable –
Source of intervention data	– not applicable –
Availability of data on exposure	– not applicable –
**Methodological approach**	
Empirical approach	counterfactual
Use of exposure variation	only variation over time for multiple populations
Method	mechanistic model (compartmental single-population transmission model)
Code availability	none
**Effectiveness assessment**	
Reporting of intervention effectiveness	quantification of change in outcome values
Measure of effectiveness	cases
Interpretation of results	implicitly causal
Reporting of uncertainty	no
Sensitivity analysis	none
Subgroup assessment	none

He et al. 2020b

Study information

– continued on next page –

Author	He S, Tang AS, And LR
Title	A Discrete Stochastic Model of the COVID-19 Outbreak: Forecast and Control
Year	2020
Journal	Mathematical Biosciences and Engineering
DOI	10.3934/mbe.2020153
Date received	2020-02-22
Date accepted	2020-03-12
**Study setting**	
Number of populations included	single
Level of populations included	national (China)
Geographic areas covered	Asia
Number of countries covered	single country (China)
Study period	start and end date span first epidemic wave
**Outcome**	
Raw outcome	epidemiological population-level outcome (cases, deaths, recovered cases)
Time resolution of raw outcome	daily
Computed outcome	epidemiological parameter (reproduction number, contact rate)
Method to obtain the computed outcome	compartmental transmission model (single-population)
Data source	data from (sub)national authorities
Data availability	data access via source
**Intervention**	
Terminology for interventions	measures
Terminology for the specific type of non-pharmaceutical interventions	control

– continued on next page –

Exposure types	all interventions together
Types of single interventions	– not applicable –
Coding of interventions	– not applicable –
Source of intervention data	– not applicable –
Availability of data on exposure	– not applicable –
**Methodological approach**	
Empirical approach	descriptive
Use of exposure variation	only variation over time for a single population
Method	description of change over time (description of time course)
Code availability	none
**Effectiveness assessment**	
Reporting of intervention effectiveness	qualitative statement
Measure of effectiveness	reproduction number, contact rate
Interpretation of results	implicitly causal
Reporting of uncertainty	– not applicable –
Sensitivity analysis	none
Subgroup assessment	none

Henríquez et al. 2020 (Analysis 1)

**Study information**	
Author	Henríquez J, Gonzalo-Almorox E, García-Goñi M, Paolucci F
Title	The First Months of the COVID-19 Pandemic in Spain

– continued on next page –

Year	2020
Journal	Health Policy and Technology
DOI	10.1016/j.hlpt.2020.08.013
Date received	– could not be evaluated –
Date accepted	2020-08-27 (published)
**Study setting**	
Number of populations included	single
Level of populations included	national (Spain)
Geographic areas covered	Europe
Number of countries covered	single country (Spain)
Study period	start and end date span first epidemic wave
**Outcome**	
Raw outcome	epidemiological population-level outcome (cases)
Time resolution of raw outcome	daily
Computed outcome	none
Method to obtain the computed outcome	– not applicable –
Data source	data from (sub)national authorities
Data availability	data access via source
**Intervention**	
Terminology for interventions	measures
Terminology for the specific type of non-pharmaceutical interventions	containment, mitigation
Exposure types	stringency index (OxCGRT Stringency Index)
Types of single interventions	– not applicable –
Coding of interventions	necessary

– continued on next page –

Source of intervention data	use of externally coded data (OxCGRT)
Availability of data on exposure	access to externally coded data via source
**Methodological approach**	
Empirical approach	descriptive
Use of exposure variation	only variation over time for a single population
Method	description of change over time (description of time course)
Code availability	none
**Effectiveness assessment**	
Reporting of intervention effectiveness	qualitative statement
Measure of effectiveness	cases
Interpretation of results	implicitly causal
Reporting of uncertainty	– not applicable –
Sensitivity analysis	none
Subgroup assessment	none

Henríquez et al. 2020 (Analysis 2)

**Study information**	
Author	Henríquez J, Gonzalo-Almorox E, García-Goñi M, Paolucci F
Title	The First Months of the COVID-19 Pandemic in Spain
Year	2020
Journal	Health Policy and Technology
DOI	10.1016/j.hlpt.2020.08.013
Date received	– could not be evaluated –

– continued on next page –

Date accepted	2020-08-27 (published)
**Study setting**	
Number of populations included	multiple
Level of populations included	both national and subnational (Spain and all its autonomous communities)
Geographic areas covered	Europe
Number of countries covered	single country (Spain)
Study period	start and end date span first epidemic wave: behavioral
**Outcome**	
Raw outcome	behavioral outcome (mobility)
Time resolution of raw outcome	daily
Computed outcome	none
Method to obtain the computed outcome	– not applicable –
Data source	– could not be evaluated –
Data availability	data not accessible
**Intervention**	
Terminology for interventions	measures
Terminology for the specific type of non-pharmaceutical interventions	containment, mitigation
Exposure types	all interventions together
Types of single interventions	– not applicable –
Coding of interventions	– not applicable –
Source of intervention data	– not applicable –
Availability of data on exposure	– not applicable –
**Methodological approach**	
Empirical approach	descriptive

– continued on next page –

Use of exposure variation	only variation over time for a single population
Method	description of change over time (description of time course)
Code availability	none
**Effectiveness assessment**	
Reporting of intervention effectiveness	qualitative statement
Measure of effectiveness	mobility
Interpretation of results	implicitly causal
Reporting of uncertainty	– not applicable –
Sensitivity analysis	none
Subgroup assessment	none

Holtz et al. 2020

**Study information**	
Author	Holtz D, Zhao M, Benzell SG, Cao CY, Rahimian MA, Yang J, Allen J, Collis A, Moehring A, Sowrirajan T, Ghosh D, Zhang Y, Dhillon PS, Nicolaides C, Eckles D, Aral S
Title	Interdependence and the Cost of Uncoordinated Responses to COVID-19
Year	2020
Journal	Proceedings of the National Academy of Sciences
DOI	10.1073/pnas.2009522117
Date received	2020-05-12
Date accepted	2020-07-10
**Study setting**	
Number of populations included	multiple

– continued on next page –

Level of populations included	both national and subnational (2,502 US counties)
Geographic areas covered	North America
Number of countries covered	single country (US)
Study period	start and end date span first epidemic wave: behavioral
**Outcome**	
Raw outcome	behavioral outcome (mobility: fraction of mobile devices leaving home and mean number of locations visited per device)
Time resolution of raw outcome	daily
Computed outcome	none
Method to obtain the computed outcome	– not applicable –
Data source	mobility data from corporate organizations (Safegraph (fraction of mobile devices leaving home), Facebook (mean number of locations visited per device))
Data availability	data made available by the authors
**Intervention**	
Terminology for interventions	measures
Terminology for the specific type of non-pharmaceutical interventions	social distancing
Exposure types	multiple separate interventions (stay-at-home order, venue closure)
Types of single interventions	stay-at-home order, venue closure
Coding of interventions	necessary
Source of intervention data	use of externally coded data (same as in Killeen et al.)
Availability of data on exposure	access to externally coded data via source
**Methodological approach**	
Empirical approach	parametric

– continued on next page –

Use of exposure variation	both variation over time and between populations
Method	non-mechanistic model (generalized linear model)
Code availability	publicly available
**Effectiveness assessment**	
Reporting of intervention effectiveness	quantification of change in outcome values
Measure of effectiveness	mobility: fraction of mobile devices leaving home and mean number of locations visited per device
Interpretation of results	explicitly causal
Reporting of uncertainty	yes
Sensitivity analysis	different coding of interventions used (policy effects considered with lags and leads), model specification varied, same analysis with (sub)population(s) excluded
Subgroup assessment	none

Hsiang et al. 2020

**Study information**	
Author	Hsiang S, Allen D, Annan-Phan S, Bell K, Bolliger I, Chong T, Druckenmiller H, Huang LY, Hultgren A, Krasovich E, Lau P, Lee J, Rolf E, Tseng J, Wu T
Title	The Effect of Large-Scale Anti-Contagion Policies on the COVID-19 Pandemic
Year	2020
Journal	Nature
DOI	10.1038/s41586-020-2404-8
Date received	2020-03-22

– continued on next page –

Date accepted	2020-05-26
**Study setting**	
Number of populations included	multiple
Level of populations included	both national and subnational (China, South Korea, Italy, Iran, France, the US and the subnational regions of these countries)
Geographic areas covered	Asia, Europe, Middle East and Africa, North America
Number of countries covered	multiple countries
Study period	same end date for several populations with diverse epidemic trajectories
**Outcome**	
Raw outcome	epidemiological population-level outcome (cases, active cases, deaths, recovered cases)
Time resolution of raw outcome	daily
Computed outcome	measure of epidemic trend (growth rate, growth rate: active cases)
Method to obtain the computed outcome	simple computation
Data source	data from (sub)national authorities (China, South Korea, Iran, Italy, France), public (USAFacts: US)
Data availability	data made available by the authors
**Intervention**	
Terminology for interventions	policies
Terminology for the specific type of non-pharmaceutical interventions	anti-contagion
Exposure types	multiple separate interventions (individual and aggregated country-specific measures (includes travel restrictions school closure, business closure, religious closure, work bans, event bans gathering bans, home isolation, emergency declaration, etc))

– continued on next page –

Types of single interventions	international travel restrictions, workplace closure, venue closure, bans of large gatherings, stay-at-home order, declaration of a state of emergency
Coding of interventions	necessary
Source of intervention data	coding done by authors
Availability of data on exposure	coded data made available by the authors
**Methodological approach**	
Empirical approach	parametric
Use of exposure variation	both variation over time and between populations
Method	non-mechanistic model (generalized linear model)
Code availability	publicly available
**Effectiveness assessment**	
Reporting of intervention effectiveness	quantification of change in outcome values
Measure of effectiveness	growth rate, growth rate: active cases
Interpretation of results	explicitly causal
Reporting of uncertainty	yes
Sensitivity analysis	different or modified outcome used (bias related to underreporting of cases estimated), model specification varied, epidemiological parameters varied
Subgroup assessment	none

Huang et al. 2020

**Study information**	
Author	Huang X, Li Z, Jiang Y, Li X, Porter D
Title	Twitter Reveals Human Mobility Dynamics during the COVID-19 Pandemic
Year	2020

– continued on next page –

Journal	PLOS ONE
DOI	10.1371/journal.pone.0241957
Date received	2020-06-24
Date accepted	2020-10-23
**Study setting**	
Number of populations included	multiple
Level of populations included	both national and subnational (20 selected countries around the world, US states)
Geographic areas covered	Europe, Middle East and Africa, Asia, Oceania, Central and South America, North America
Number of countries covered	multiple countries
Study period	start and end date span first epidemic wave: behavioral
**Outcome**	
Raw outcome	behavioral outcome (mobility: index based on distances)
Time resolution of raw outcome	daily
Computed outcome	none
Method to obtain the computed outcome	– not applicable –
Data source	mobility data from corporate organizations (Twitter)
Data availability	data made available by the authors
**Intervention**	
Terminology for interventions	measures
Terminology for the specific type of non-pharmaceutical interventions	mitigation
Exposure types	all interventions together
Types of single interventions	– not applicable –

– continued on next page –

Coding of interventions	– not applicable –
Source of intervention data	– not applicable –
Availability of data on exposure	– not applicable –
**Methodological approach**	
Empirical approach	descriptive
Use of exposure variation	only variation over time for multiple populations
Method	description of change over time (description of time course)
Code availability	none
**Effectiveness assessment**	
Reporting of intervention effectiveness	qualitative statement
Measure of effectiveness	mobility: index based on distances
Interpretation of results	associative
Reporting of uncertainty	– not applicable –
Sensitivity analysis	none
Subgroup assessment	none

Huber & Langen 2020 (Analysis 1)

**Study information**	
Author	Huber M, Langen H
Title	Timing Matters: The Impact of Response Measures on COVID-19-related Hospitalization and Death Rates in Germany and Switzerland
Year	2020

– continued on next page –

Journal	Swiss Journal of Economics and Statistics
DOI	10.1186/s41937-020-00054-w
Date received	2020-06-18
Date accepted	2020-07-27
**Study setting**	
Number of populations included	multiple
Level of populations included	both national and subnational (Germany, Switzerland and its states/counties/cantons)
Geographic areas covered	Europe
Number of countries covered	multiple countries
Study period	start and end date span first epidemic wave
**Outcome**	
Raw outcome	epidemiological population-level outcome (hospitalizations, deaths)
Time resolution of raw outcome	daily
Computed outcome	none
Method to obtain the computed outcome	– not applicable –
Data source	data from (sub)national authorities
Data availability	data access via source
**Intervention**	
Terminology for interventions	measures
Terminology for the specific type of non-pharmaceutical interventions	response
Exposure types	start of interventions (start date of lockdown measures (including the closure of non-essential shops, schools, childcare centers, cafes, bars, and restaurants, bans on gatherings))

– continued on next page –

Types of single interventions	– not applicable –
Coding of interventions	not necessary
Source of intervention data	government or news websites
Availability of data on exposure	raw data documented in the manuscript
**Methodological approach**	
Empirical approach	parametric
Use of exposure variation	only variation between populations
Method	non-mechanistic model (generalized linear model)
Code availability	none
**Effectiveness assessment**	
Reporting of intervention effectiveness	quantification of change in outcome values
Measure of effectiveness	hospitalizations, deaths
Interpretation of results	explicitly causal
Reporting of uncertainty	yes
Sensitivity analysis	model specification varied
Subgroup assessment	none

Huber & Langen 2020 (Analysis 2)

**Study information**	
Author	Huber M, Langen H
Title	Timing Matters: The Impact of Response Measures on COVID-19-related Hospitalization and Death Rates in Germany and Switzerland
Year	2020
Journal	Swiss Journal of Economics and Statistics

– continued on next page –

DOI	10.1186/s41937-020-00054-w
Date received	2020-06-18
Date accepted	2020-07-27
**Study setting**	
Number of populations included	multiple
Level of populations included	subnational (2 cantons in Switzerland)
Geographic areas covered	Europe
Number of countries covered	single country (Switzerland)
Study period	start and end date span first epidemic wave
**Outcome**	
Raw outcome	epidemiological population-level outcome (hospitalizations, deaths)
Time resolution of raw outcome	daily
Computed outcome	none
Method to obtain the computed outcome	– not applicable –
Data source	data from (sub)national authorities
Data availability	data access via source
**Intervention**	
Terminology for interventions	measures
Terminology for the specific type of non-pharmaceutical interventions	response
Exposure types	start of interventions (start date of lockdown measures (including the closure of non-essential shops, schools, childcare centers, cafes, bars, and restaurants, bans on gatherings))
Types of single interventions	– not applicable –
Coding of interventions	not necessary

– continued on next page –

Source of intervention data	government or news websites
Availability of data on exposure	raw data documented in the manuscript
**Methodological approach**	
Empirical approach	counterfactual
Use of exposure variation	only variation over time for multiple populations
Method	synthetic controls
Code availability	none
**Effectiveness assessment**	
Reporting of intervention effectiveness	quantification of change in outcome values
Measure of effectiveness	hospitalizations, deaths
Interpretation of results	explicitly causal
Reporting of uncertainty	no
Sensitivity analysis	none
Subgroup assessment	none

Huber & Langen 2020 (Analysis 3)

**Study information**	
Author	Huber M, Langen H
Title	Timing Matters: The Impact of Response Measures on COVID-19-related Hospitalization and Death Rates in Germany and Switzerland
Year	2020
Journal	Swiss Journal of Economics and Statistics
DOI	10.1186/s41937-020-00054-w
Date received	2020-06-18

– continued on next page –

Date accepted	2020-07-27
**Study setting**	
Number of populations included	multiple
Level of populations included	both national and subnational (Germany and its states)
Geographic areas covered	Europe
Number of countries covered	single country (Germany)
Study period	start and end date span first epidemic wave
**Outcome**	
Raw outcome	epidemiological population-level outcome (hospitalizations, deaths)
Time resolution of raw outcome	daily
Computed outcome	none
Method to obtain the computed outcome	– not applicable –
Data source	data from (sub)national authorities
Data availability	data access via source
**Intervention**	
Terminology for interventions	measures
Terminology for the specific type of non-pharmaceutical interventions	response
Exposure types	one single intervention (curfew)
Types of single interventions	stay-at-home order
Coding of interventions	not necessary
Source of intervention data	government or news websites
Availability of data on exposure	raw data documented in the manuscript
**Methodological approach**	
Empirical approach	parametric

– continued on next page –

Huber & Langen 2020 (Analysis 3) – continued from previous page

Use of exposure variation	only variation between populations
Method	non-mechanistic model (generalized linear model)
Code availability	none
**Effectiveness assessment**	
Reporting of intervention effectiveness	quantification of change in outcome values
Measure of effectiveness	hospitalizations, deaths
Interpretation of results	explicitly causal
Reporting of uncertainty	yes
Sensitivity analysis	none
Subgroup assessment	none

Hyafil & Moriña 2021

**Study information**	
Author	Hyafil A, Moriña D
Title	Analysis of the Impact of Lockdown on the Reproduction Number of the SARS-Cov-2 in Spain
Year	2021
Journal	Gaceta Sanitaria
DOI	10.1016/j.gaceta.2020.05.003
Date received	2020-04-18
Date accepted	2020-05-06
**Study setting**	
Number of populations included	single

– continued on next page –

Level of populations included	national (Spain)
Geographic areas covered	Europe
Number of countries covered	single country (Spain)
Study period	start and end date span first epidemic wave
**Outcome**	
Raw outcome	epidemiological population-level outcome (hospitalizations)
Time resolution of raw outcome	daily
Computed outcome	epidemiological parameter (reproduction number)
Method to obtain the computed outcome	compartmental transmission model (single-population)
Data source	data from (sub)national authorities
Data availability	data access via source
**Intervention**	
Terminology for interventions	interventions
Terminology for the specific type of non-pharmaceutical interventions	non-pharmaceutical
Exposure types	multiple separate interventions (emergency declaration, stay-at-home order)
Types of single interventions	declaration of a state of emergency, stay-at-home order
Coding of interventions	not necessary
Source of intervention data	– could not be evaluated –
Availability of data on exposure	raw data documented in the manuscript
**Methodological approach**	
Empirical approach	descriptive
Use of exposure variation	only variation over time for a single population
Method	description of change over time (comparison of time periods)

– continued on next page –

Code availability	none
**Effectiveness assessment**	
Reporting of intervention effectiveness	comparison of outcome values
Measure of effectiveness	reproduction number
Interpretation of results	implicitly causal
Reporting of uncertainty	yes
Sensitivity analysis	different or modified outcome used (use cases and deaths instead of hospitalizations as raw different or modified outcome used), epidemiological parameters varied
Subgroup assessment	none

Islam et al. 2020

**Study information**	
Author	Islam N, Sharp SJ, Chowell G, Shabnam S, Kawachi I, Lacey B, Massaro JM, D'Agostino RB, White M
Title	Physical Distancing Interventions and Incidence of Coronavirus Disease 2019: Natural Experiment in 149 Countries
Year	2020
Journal	BMJ
DOI	10.1136/bmj.m2743
Date received	– could not be evaluated –
Date accepted	2020-07-08
**Study setting**	
Number of populations included	multiple

– continued on next page –

Level of populations included	national (149 countries and regions)
Geographic areas covered	Europe, Middle East and Africa, Asia, Oceania, Central and South America, North America
Number of countries covered	multiple countries
Study period	end date could not be evaluated
**Outcome**	
Raw outcome	epidemiological population-level outcome (cases)
Time resolution of raw outcome	daily
Computed outcome	none
Method to obtain the computed outcome	– not applicable –
Data source	data from publicly available cross-country selections (ECDC)
Data availability	data made available by the authors
**Intervention**	
Terminology for interventions	measures
Terminology for the specific type of non-pharmaceutical interventions	physical distancing
Exposure types	multiple separate interventions (school closure, bans of large gatherings, workplace closure, stay-at-home order, public transport closure)
Types of single interventions	school closure, bans of large gatherings, workplace closure, stay-at-home order, public transport closure
Coding of interventions	necessary
Source of intervention data	use of externally coded data (OxCGRT)
Availability of data on exposure	coded data made available by the authors
**Methodological approach**	
Empirical approach	parametric

– continued on next page –

Use of exposure variation	only variation over time for multiple populations
Method	non-mechanistic model (generalized linear model)
Code availability	none
**Effectiveness assessment**	
Reporting of intervention effectiveness	quantification of change in outcome values
Measure of effectiveness	incidence rate ratio
Interpretation of results	associative
Reporting of uncertainty	yes
Sensitivity analysis	epidemiological parameters varied, model specification varied, same analysis with (sub)population(s) excluded
Subgroup assessment	none

Jardine et al. 2020

**Study information**	
Author	Jardine R, Wright J, Samad Z, Bhutta ZA
Title	Analysis of COVID-19 Burden, Epidemiology and Mitigation Strategies in Muslim Majority Countries
Year	2020
Journal	Eastern Mediterranean Health Journal
DOI	10.26719/emhj.20.120
Date received	2020-06-26
Date accepted	2020-09-14
**Study setting**	
Number of populations included	multiple

– continued on next page –

Level of populations included	national (44 countries with Muslim majority)
Geographic areas covered	Middle East and Africa
Number of countries covered	multiple countries
Study period	same end date for several populations with diverse epidemic trajectories
**Outcome**	
Raw outcome	epidemiological population-level outcome (cases)
Time resolution of raw outcome	daily
Computed outcome	measure of epidemic trend (doubling time)
Method to obtain the computed outcome	simple computation
Data source	data from publicly available cross-country selections (JHU)
Data availability	data access via source
**Intervention**	
Terminology for interventions	interventions
Terminology for the specific type of non-pharmaceutical interventions	non-pharmaceutical
Exposure types	one combination of interventions (strict movement restrictions (visa restrictions, domestic travel restrictions and curfew))
Types of single interventions	– not applicable –
Coding of interventions	necessary
Source of intervention data	use of externally coded data (ACAPS Government Measures Dataset)
Availability of data on exposure	access to externally coded data via source
**Methodological approach**	
Empirical approach	descriptive
Use of exposure variation	only variation between populations
Method	comparison of populations

– continued on next page –

Code availability	none
**Effectiveness assessment**	
Reporting of intervention effectiveness	quantification of change in outcome values
Measure of effectiveness	cases, doubling time
Interpretation of results	associative
Reporting of uncertainty	yes
Sensitivity analysis	none
Subgroup assessment	none

**Jarvis et al. 2020**	
**Study information**	
Author	Jarvis CI, Van Zandvoort AK, Gimma A, Prem K, Klepac P, Rubin GJ, Edmunds WJ
Title	Quantifying the Impact of Physical Distance Measures on the Transmission of COVID-19 in the UK
Year	2020
Journal	BMC Medicine
DOI	10.1186/s12916-020-01597-8
Date received	2020-04-03
Date accepted	2020-04-17
**Study setting**	
Number of populations included	single
Level of populations included	national (UK)
Geographic areas covered	Europe

– continued on next page –

Number of countries covered	single country (UK)
Study period	late start
**Outcome**	
Raw outcome	behavioral outcome (survey: number of contacts & adherence to NPIs)
Time resolution of raw outcome	– not applicable –
Computed outcome	epidemiological parameter (reproduction number)
Method to obtain the computed outcome	statistical estimation of reproduction number from individual data (Diekmann et al.)
Data source	data collected by authors
Data availability	data made available by the authors
**Intervention**	
Terminology for interventions	measures
Terminology for the specific type of non-pharmaceutical interventions	physical distancing
Exposure types	one combination of interventions (lockdown on March 23)
Types of single interventions	– not applicable –
Coding of interventions	not necessary
Source of intervention data	– could not be evaluated –
Availability of data on exposure	raw data documented in the manuscript
**Methodological approach**	
Empirical approach	descriptive
Use of exposure variation	only variation over time for a single population
Method	description of change over time (comparison of time periods)
Code availability	publicly available
**Effectiveness assessment**	

– continued on next page –

Reporting of intervention effectiveness	quantification of change in outcome values
Measure of effectiveness	number of contacts, adherence to NPIs, reproduction number
Interpretation of results	implicitly causal
Reporting of uncertainty	yes
Sensitivity analysis	epidemiological parameters varied
Subgroup assessment	socioeconomic indicators (age, gender, household size)

Jefferies et al. 2020

**Study information**	
Author	Jefferies S, French N, Gilkison C, Graham G, Hope V, Marshall J, McElnay C, McNeill A, Muellner P, Paine S, Prasad N, Scott J, Sherwood J, Yang L, Priest P
Title	COVID-19 in New Zealand and the Impact of the National Response: A Descriptive Epidemiological Study
Year	2020
Journal	The Lancet Public Health
DOI	10.1016/s2468-2667(20)30225-5
Date received	– could not be evaluated –
Date accepted	2020-10-13 (published)
**Study setting**	
Number of populations included	single
Level of populations included	national (New Zealand)
Geographic areas covered	Oceania
Number of countries covered	single country (New Zealand)

– continued on next page –

Study period	start and end date span first epidemic wave
**Outcome**	
Raw outcome	epidemiological individual-level outcome (individual cases)
Time resolution of raw outcome	daily
Computed outcome	epidemiological parameter (time from symptom onset to isolation, isolation interval)
Method to obtain the computed outcome	simple computation
Data source	data from (sub)national authorities
Data availability	data not accessible
**Intervention**	
Terminology for interventions	responses
Terminology for the specific type of non-pharmaceutical interventions	government
Exposure types	phases of interventions (five phases of different nonpharmaceutical interventions which ronly oughly correspond to different alert levels)
Types of single interventions	– not applicable –
Coding of interventions	not necessary
Source of intervention data	– could not be evaluated –
Availability of data on exposure	raw data documented in the manuscript
**Methodological approach**	
Empirical approach	descriptive
Use of exposure variation	only variation over time for a single population
Method	description of change over time (comparison of time periods)
Code availability	none
**Effectiveness assessment**	

– continued on next page –

Reporting of intervention effectiveness	comparison of outcome values
Measure of effectiveness	cases, hospitalizations, ICU hospitalizations, deaths, time from symptom onset to isolation, isolation interval
Interpretation of results	associative
Reporting of uncertainty	yes
Sensitivity analysis	same analysis with (sub)population(s) excluded
Subgroup assessment	socioeconomic indicators (age, gender, income, health care occupation, ethnicity), epidemiological indicators (source of infection)

Jeffrey et al. 2020 (Analysis 1)

**Study information**	
Author	Jeffrey B, Walters CE, Ainslie KE, Eales O, Ciavarella C, Bhatia S, Hayes S, Baguelin M, Boonyasiri A, Brazeau NF, Cuomo-Dannenburg G, FitzJohn RG, Gaythorpe K, Green W, Imai N, Mellan TA, Mishra S, Nouvellet P, Unwin HJ, Verity R, Vollmer M, Whittaker C, Ferguson NM, Donnelly CA, Riley S
Title	Anonymised and Aggregated Crowd Level Mobility Data from Mobile Phones Suggests That Initial Compliance with COVID-19 Social Distancing Interventions Was High and Geographically Consistent across the UK
Year	2020
Journal	Wellcome Open Research
DOI	10.12688/wellcomeopenres.15997.1
Date received	– could not be evaluated –
Date accepted	2020-07-17 (published)

– continued on next page –

**Study setting**	
Number of populations included	multiple
Level of populations included	both national and subnational (UK countries, local authority districts, cities)
Geographic areas covered	Europe
Number of countries covered	single country (UK)
Study period	start and end date span first epidemic wave: behavioral
**Outcome**	
Raw outcome	epidemiological population-level outcome (cases), behavioral outcome (mobility: transit station mobility index & subway validations)
Time resolution of raw outcome	daily
Computed outcome	none
Method to obtain the computed outcome	– not applicable –
Data source	mobility data from corporate organizations (Facebook (number of trips and trip lengths), O2 (number of trips and trip lengths))
Data availability	data made available by the authors
**Intervention**	
Terminology for interventions	interventions
Terminology for the specific type of non-pharmaceutical interventions	social distancing
Exposure types	multiple separate interventions (school closure, venue closure, stay-at-home order)
Types of single interventions	school closure, venue closure, stay-at-home order
Coding of interventions	not necessary
Source of intervention data	government or news websites

– continued on next page –

Availability of data on exposure	raw data documented in the manuscript
**Methodological approach**	
Empirical approach	descriptive
Use of exposure variation	only variation over time for multiple populations
Method	description of change over time (comparison of time periods)
Code availability	publicly available
**Effectiveness assessment**	
Reporting of intervention effectiveness	comparison of outcome values
Measure of effectiveness	mobility: percentage difference from baseline number of trips and trip lengths
Interpretation of results	implicitly causal
Reporting of uncertainty	no
Sensitivity analysis	different or modified outcome used (different mobility data used)
Subgroup assessment	socioeconomic indicators (population density)

Jeffrey et al. 2020 (Analysis 2, mobility as exposure)

**Study information**	
Author	Jeffrey B, Walters CE, Ainslie KE, Eales O, Ciavarella C, Bhatia S, Hayes S, Baguelin M, Boonyasiri A, Brazeau NF, Cuomo-Dannenburg G, FitzJohn RG, Gaythorpe K, Green W, Imai N, Mellan TA, Mishra S, Nouvellet P, Unwin HJ, Verity R, Vollmer M, Whittaker C, Ferguson NM, Donnelly CA, Riley S
Title	Anonymised and Aggregated Crowd Level Mobility Data from Mobile Phones Suggests That Initial Compliance with COVID-19 Social Distancing Interventions Was High and Geographically Consistent across the UK

– continued on next page –

Year	2020
Journal	Wellcome Open Research
DOI	10.12688/wellcomeopenres.15997.1
Date received	– could not be evaluated –
Date accepted	17.07.2020 (published)
**Study setting**	
Number of populations included	multiple
Level of populations included	subnational (local authority districts in UK)
Geographic areas covered	Europe
Number of countries covered	single country (UK)
Study period	start and end date span first epidemic wave
**Outcome**	
Raw outcome	behavioral outcome (mobility: number of trips)
Time resolution of raw outcome	daily
Computed outcome	none
Method to obtain the computed outcome	– not applicable –
Data source	mobility data by corporate organizations (Facebook)
Data availability	data made available by the authors
**Intervention**	
Terminology for interventions	interventions
Terminology for the specific type of non-pharmaceutical interventions	social distancing
Exposure types	change points (mobility: number of trips)
Types of single interventions	– not applicable –
Coding of interventions	– not applicable –

– continued on next page –

Source of intervention data	data collected by authors
Availability of data on exposure	data made available by the authors
**Methodological approach**	
Empirical approach	descriptive
Use of exposure variation	only variation over time for multiple populations
Method	description of change over time (comparison of time periods)
Code availability	publicly available
**Effectiveness assessment**	
Reporting of intervention effectiveness	quantification of change in outcome values
Measure of effectiveness	mobility: percentage difference from baseline number of trips
Interpretation of results	implicitly causal
Reporting of uncertainty	yes
Sensitivity analysis	model specification varied
Subgroup assessment	none

Ji et al. 2020

**Study information**	
Author	Ji T, Chen HL, Xu J, Wu LN, Li JJ, Chen K, Qin G
Title	Lockdown Contained the Spread of 2019 Novel Coronavirus Disease in Huangshi City, China: Early Epidemiological Findings
Year	2020
Journal	Clinical Infectious Diseases
DOI	10.1093/cid/ciaa390

– continued on next page –

Date received	2020-02-24
Date accepted	2020-04-03
**Study setting**	
Number of populations included	single
Level of populations included	subnational (Huangshi (China))
Geographic areas covered	Asia
Number of countries covered	single country (China)
Study period	start and end date span first epidemic wave
**Outcome**	
Raw outcome	epidemiological population-level outcome (cases)
Time resolution of raw outcome	daily
Computed outcome	none
Method to obtain the computed outcome	– not applicable –
Data source	data from (sub)national authorities
Data availability	data access via source
**Intervention**	
Terminology for interventions	interventions
Terminology for the specific type of non-pharmaceutical interventions	non-pharmaceutical
Exposure types	one combination of interventions (Wuhan City Lockdown)
Types of single interventions	– not applicable –
Coding of interventions	not necessary
Source of intervention data	another study (Rubin et al.)
Availability of data on exposure	raw data documented in the manuscript
**Methodological approach**	

– continued on next page –

Empirical approach	descriptive
Use of exposure variation	only variation over time for a single population
Method	description of change over time (description of time course)
Code availability	none
**Effectiveness assessment**	
Reporting of intervention effectiveness	qualitative statement
Measure of effectiveness	cases
Interpretation of results	implicitly causal
Reporting of uncertainty	– not applicable –
Sensitivity analysis	none
Subgroup assessment	none

Jia et al. 2020

**Study information**	
Author	Jia JS, Lu X, Yuan Y, Xu G, Jia J, Christakis NA
Title	Population Flow Drives Spatio-Temporal Distribution of COVID-19 in China
Year	2020
Journal	Nature
DOI	10.1038/s41586-020-2284-y
Date received	2020-02-18
Date accepted	2020-04-21
**Study setting**	

– continued on next page –

Number of populations included	multiple
Level of populations included	both national and subnational (China and its provinces)
Geographic areas covered	Asia
Number of countries covered	single country (China)
Study period	start and end date span first epidemic wave: behavioral
**Outcome**	
Raw outcome	behavioral outcome (mobility: migration from Wuhan)
Time resolution of raw outcome	daily
Computed outcome	none
Method to obtain the computed outcome	– not applicable –
Data source	mobility data from corporate organizations (major national carrier)
Data availability	data made available by the authors
**Intervention**	
Terminology for interventions	interventions
Terminology for the specific type of non-pharmaceutical interventions	public health
Exposure types	one combination of interventions (Wuhan City Lockdown)
Types of single interventions	– not applicable –
Coding of interventions	not necessary
Source of intervention data	– could not be evaluated –
Availability of data on exposure	raw data documented in the manuscript
**Methodological approach**	
Empirical approach	descriptive
Use of exposure variation	only variation over time for multiple populations
Method	description of change over time (description of time course)

– continued on next page –

Code availability	publicly available
**Effectiveness assessment**	
Reporting of intervention effectiveness	quantification of change in outcome values
Measure of effectiveness	mobility: migration from Wuhan
Interpretation of results	implicitly causal
Reporting of uncertainty	no
Sensitivity analysis	none
Subgroup assessment	none

**Jüni et al. 2020**	
**Study information**	
Author	Jüni P, Rothenbühler M, Bobos P, Thorpe KE, da Costa BR, Fisman DN, Slutsky AS, Gesink D
Title	Impact of Climate and Public Health Interventions on the COVID-19 Pandemic: A Prospective Cohort Study
Year	2020
Journal	Canadian Medical Association Journal
DOI	10.1503/cmaj.200920
Date received	– could not be evaluated –
Date accepted	2020-05-05
**Study setting**	
Number of populations included	multiple
Level of populations included	both national and subnational (144 "geopolitical areas")

– continued on next page –

Geographic areas covered	Europe, Middle East and Africa, Asia, Oceania, Central and South America, North America
Number of countries covered	multiple countries
Study period	late start
**Outcome**	
Raw outcome	epidemiological population-level outcome (cases)
Time resolution of raw outcome	– not applicable –
Computed outcome	measure of epidemic trend (growth rate)
Method to obtain the computed outcome	simple computation
Data source	data from publicly available cross-country selections (JHU)
Data availability	data access via source
**Intervention**	
Terminology for interventions	interventions
Terminology for the specific type of non-pharmaceutical interventions	public health
Exposure types	multiple combinations of interventions (bans of small gatherings, school closure, other social distancing measures (there was variation in measures of social distancing reported by different geopolitical areas, including recommendations or requirements regarding social distancing, closure of sit-in restaurants and bars, or closure of nongrocery stores))
Types of single interventions	– not applicable –
Coding of interventions	necessary
Source of intervention data	coding done by authors
Availability of data on exposure	coded data not available
**Methodological approach**	
Empirical approach	parametric

– continued on next page –

Use of exposure variation	only variation between populations
Method	non-mechanistic model (generalized linear model)
Code availability	none
**Effectiveness assessment**	
Reporting of intervention effectiveness	quantification of change in outcome values
Measure of effectiveness	growth rate
Interpretation of results	associative
Reporting of uncertainty	yes
Sensitivity analysis	model specification varied
Subgroup assessment	none

**Kang & Kim 2020**	
**Study information**	
Author	Kang N, Kim B
Title	The Effects of Border Shutdowns on the Spread of COVID-19
Year	2020
Journal	Journal of Preventive Medicine and Public Health
DOI	10.3961/jpmph.20.332
Date received	2020-07-17
Date accepted	2020-07-20
**Study setting**	
Number of populations included	multiple
Level of populations included	national (Australia, Singapore, USA, Vietnam, Taiwan, Hong Kong)

– continued on next page –

Geographic areas covered	Europe, Middle East and Africa, Asia, Oceania, Central and South America, North America
Number of countries covered	multiple countries
Study period	end date in growth phase of wave
**Outcome**	
Raw outcome	epidemiological population-level outcome (cases)
Time resolution of raw outcome	daily
Computed outcome	none
Method to obtain the computed outcome	– not applicable –
Data source	data from publicly available cross-country selections (WHO)
Data availability	data access via source
**Intervention**	
Terminology for interventions	– not applicable –
Terminology for the specific type of non-pharmaceutical interventions	border shutdown
Exposure types	one single intervention (travel restrictions (entry bans against China))
Types of single interventions	international travel restrictions
Coding of interventions	not necessary
Source of intervention data	government or news websites
Availability of data on exposure	raw data documented in the manuscript
**Methodological approach**	
Empirical approach	counterfactual
Use of exposure variation	only variation over time for multiple populations
Method	synthetic controls
Code availability	none

– continued on next page –

**Effectiveness assessment**	
Reporting of intervention effectiveness	quantification of change in outcome values
Measure of effectiveness	cases
Interpretation of results	implicitly causal
Reporting of uncertainty	no
Sensitivity analysis	different or modified outcome used (new cases used instead of cumulative cases), model specification varied
Subgroup assessment	none

Karnakov et al. 2020 (Analysis 1)

**Study information**	
Author	Karnakov P, Arampatzis G, Kii I, Wermelinger F, Wlchli D, Papadimitriou C, Koumoutsakos P
Title	Data-Driven Inference of the Reproduction Number for COVID-19 before and after Interventions for 51 European Countries
Year	2020
Journal	Swiss Medical Weekly
DOI	10.4414/smw.2020.20313
Date received	– could not be evaluated –
Date accepted	2020-07-17 (published)
**Study setting**	
Number of populations included	multiple
Level of populations included	national (51 countries)

– continued on next page –

Geographic areas covered	Europe
Number of countries covered	multiple countries
Study period	start and end date span first epidemic wave
**Outcome**	
Raw outcome	epidemiological population-level outcome (cases)
Time resolution of raw outcome	daily
Computed outcome	none
Method to obtain the computed outcome	– not applicable –
Data source	data from publicly available open-source projects (data from publicly available open-source projects by the Humanistic GIS Lab at University of Washington)
Data availability	data made available by the authors
**Intervention**	
Terminology for interventions	interventions
Terminology for the specific type of non-pharmaceutical interventions	non-pharmaceutical
Exposure types	start of interventions (estimated change points when interventions change R (roughly corresponding to the day of the first intervention leading to lockdown))
Types of single interventions	– not applicable –
Coding of interventions	– not applicable –
Source of intervention data	– not applicable –
Availability of data on exposure	– not applicable –
**Methodological approach**	
Empirical approach	parametric
Use of exposure variation	only variation over time for multiple populations

– continued on next page –

Method	mechanistic model (compartmental single-population transmission model)
Latent variable functionally linked to intervention	reproduction number
Code availability	publicly available
**Effectiveness assessment**	
Reporting of intervention effectiveness	comparison of outcome values
Measure of effectiveness	reproduction number
Interpretation of results	associative
Reporting of uncertainty	yes
Sensitivity analysis	model specification varied
Subgroup assessment	none

Karnakov et al. 2020 (Analysis 2)

**Study information**	
Author	Karnakov P, Arampatzis G, Kii I, Wermelinger F, Wlchli D, Papadimitriou C, Koumoutsakos P
Title	Data-Driven Inference of the Reproduction Number for COVID-19 before and after Interventions for 51 European Countries
Year	2020
Journal	Swiss Medical Weekly
DOI	10.4414/smw.2020.20313
Date received	– could not be evaluated –
Date accepted	2020-07-17 (published)
**Study setting**	

– continued on next page –

Number of populations included	multiple
Level of populations included	national (51 countries)
Geographic areas covered	Europe
Number of countries covered	multiple countries
Study period	start and end date span first epidemic wave
**Outcome**	
Raw outcome	epidemiological population-level outcome (cases)
Time resolution of raw outcome	daily
Computed outcome	change points (change points: reproduction number)
Method to obtain the computed outcome	compartmental transmission model (compartmental single-population transmission model, with change points)
Data source	data from publicly available open-source projects (data from publicly available open-source projects by the Humanistic GIS Lab at University of Washington)
Data availability	data made available by the authors
**Intervention**	
Terminology for interventions	interventions
Terminology for the specific type of non-pharmaceutical interventions	non-pharmaceutical
Exposure types	start of interventions (start date of non-pharmaceutical interventions (various))
Types of single interventions	– not applicable –
Coding of interventions	necessary
Source of intervention data	use of externally coded data (Wikipedia)
Availability of data on exposure	access to externally coded data via source
**Methodological approach**	

– continued on next page –

Empirical approach	descriptive
Use of exposure variation	only variation over time for multiple populations
Method	comparison of change points with intervention
Code availability	publicly available
**Effectiveness assessment**	
Reporting of intervention effectiveness	quantification of change in outcome values
Measure of effectiveness	change points: reproduction number
Interpretation of results	associative
Reporting of uncertainty	yes
Sensitivity analysis	none
Subgroup assessment	none

**Kaufman et al. 2020**	
**Study information**	
Author	Kaufman BG, Whitaker R, Mahendraratnam N, Smith VA, McClellan MB
Title	Comparing Associations of State Reopening Strategies with COVID-19 Burden
Year	2020
Journal	Journal of General Internal Medicine
DOI	10.1007/s11606-020-06277-0
Date received	2020-09-12
Date accepted	2020-09-28
**Study setting**	

– continued on next page –

Number of populations included	multiple
Level of populations included	both national and subnational (50 US states and Washington D.C.)
Geographic areas covered	North America
Number of countries covered	single country (US)
Study period	same end date for several populations with diverse epidemic trajectories
**Outcome**	
Raw outcome	epidemiological population-level outcome (cases, deaths)
Time resolution of raw outcome	daily
Computed outcome	none
Method to obtain the computed outcome	– not applicable –
Data source	data from public media outlets (New York Times)
Data availability	data access via source
**Intervention**	
Terminology for interventions	measures
Terminology for the specific type of non-pharmaceutical interventions	mitigation
Exposure types	one single intervention (mask mandate (statewide))
Types of single interventions	mask mandate
Coding of interventions	necessary
Source of intervention data	use of externally coded data (Boston University COVID-19 US State Policy Database)
Availability of data on exposure	coded data made available by the authors
**Methodological approach**	
Empirical approach	parametric
Use of exposure variation	both variation over time and between populations

– continued on next page –

Method	non-mechanistic model (generalized linear model)
Code availability	none
**Effectiveness assessment**	
Reporting of intervention effectiveness	quantification of change in outcome values
Measure of effectiveness	cases, deaths
Interpretation of results	implicitly causal
Reporting of uncertainty	yes
Sensitivity analysis	model specification varied, same analysis with (sub)population(s) excluded
Subgroup assessment	none

Kendall et al. 2020 (Analysis 1)

**Study information**	
Author	Kendall M, Milsom L, Abeler-Dörner L, Wymant C, Ferretti L, Briers M, Holmes C, Bonsall D, Abeler J, Fraser C
Title	Epidemiological Changes on the Isle of Wight after the Launch of the NHS Test and Trace Programme: A Preliminary Analysis
Year	2020
Journal	The Lancet Digital Health
DOI	10.1016/s2589-7500(20)30241-7
Date received	– could not be evaluated –
Date accepted	2020-10-14 (published)
**Study setting**	
Number of populations included	single

– continued on next page –

Level of populations included	national (Isle of Wight (UK))
Geographic areas covered	Europe
Number of countries covered	single country (UK)
Study period	start and end date span first epidemic wave
**Outcome**	
Raw outcome	epidemiological population-level outcome (cases)
Time resolution of raw outcome	daily
Computed outcome	epidemiological parameter (reproduction number)
Method to obtain the computed outcome	back projection, statistical estimation of reproduction number (Cori et al.)
Data source	data collected by authors (hospitals: cases), data from (sub)national authorities (cases)
Data availability	data made available by the authors
**Intervention**	
Terminology for interventions	interventions
Terminology for the specific type of non-pharmaceutical interventions	non-pharmaceutical
Exposure types	one combination of interventions (NHS Test and Trace programme)
Types of single interventions	– not applicable –
Coding of interventions	not necessary
Source of intervention data	– could not be evaluated –
Availability of data on exposure	raw data documented in the manuscript
**Methodological approach**	
Empirical approach	descriptive
Use of exposure variation	only variation over time for a single population
Method	description of change over time (comparison of time periods)

– continued on next page –

Code availability	publicly available
**Effectiveness assessment**	
Reporting of intervention effectiveness	comparison of outcome values
Measure of effectiveness	reproduction number, infections
Interpretation of results	associative
Reporting of uncertainty	no
Sensitivity analysis	none
Subgroup assessment	none

**Kendall et al. 2020 (Analysis 2)**	
**Study information**	
Author	Kendall M, Milsom L, Abeler-Dörner L, Wymant C, Ferretti L, Briers M, Holmes C, Bonsall D, Abeler J, Fraser C
Title	Epidemiological Changes on the Isle of Wight after the Launch of the NHS Test and Trace Programme: A Preliminary Analysis
Year	2020
Journal	The Lancet Digital Health
DOI	10.1016/s2589-7500(20)30241-7
Date received	– could not be evaluated –
Date accepted	2020-10-14 (published)
**Study setting**	
Number of populations included	single
Level of populations included	national (Isle of Wight (UK))
Geographic areas covered	Europe

– continued on next page –

Number of countries covered	single country (UK)
Study period	start and end date span first epidemic wave
**Outcome**	
Raw outcome	epidemiological population-level outcome (cases)
Time resolution of raw outcome	daily
Computed outcome	epidemiological parameter (reproduction number)
Method to obtain the computed outcome	back projection, statistical estimation of reproduction number (Cori et al.)
Data source	data collected by authors (hospitals: cases), data from (sub)national authorities (cases)
Data availability	data made available by the authors
**Intervention**	
Terminology for interventions	interventions
Terminology for the specific type of non-pharmaceutical interventions	non-pharmaceutical
Exposure types	one combination of interventions (NHS Test and Trace programme)
Types of single interventions	– not applicable –
Coding of interventions	not necessary
Source of intervention data	– could not be evaluated –
Availability of data on exposure	raw data documented in the manuscript
**Methodological approach**	
Empirical approach	counterfactual
Use of exposure variation	only variation over time for a single population
Method	synthetic controls
Code availability	publicly available
**Effectiveness assessment**	

– continued on next page –

Reporting of intervention effectiveness	quantification of change in outcome values
Measure of effectiveness	reproduction number
Interpretation of results	associative
Reporting of uncertainty	yes
Sensitivity analysis	none
Subgroup assessment	none

**Khan et al. 2020**	
**Study information**	
Author	Khan NM, Soobhug AD, Khan MH
Title	Studying the Trend of the Novel Coronavirus Series in Mauritius and Its Implications
Year	2020
Journal	PLOS ONE
DOI	10.1371/journal.pone.0235730
Date received	2020-05-04
Date accepted	2020-06-10
**Study setting**	
Number of populations included	single
Level of populations included	national (Mauritius)
Geographic areas covered	Middle East and Africa
Number of countries covered	single country (Mauritius)
Study period	start and end date span first epidemic wave

– continued on next page –

**Outcome**	
Raw outcome	epidemiological population-level outcome (active cases)
Time resolution of raw outcome	daily
Computed outcome	none
Method to obtain the computed outcome	– not applicable –
Data source	data from publicly available cross-country selections (ECDC)
Data availability	data made available by the authors
**Intervention**	
Terminology for interventions	measures
Terminology for the specific type of non-pharmaceutical interventions	confinement
Exposure types	one combination of interventions (sanitary curfew on March 20 (work bans, non-essential business restrictions, social distancing in public places))
Types of single interventions	– not applicable –
Coding of interventions	not necessary
Source of intervention data	– could not be evaluated –
Availability of data on exposure	raw data documented in the manuscript
**Methodological approach**	
Empirical approach	parametric
Use of exposure variation	only variation over time for a single population
Method	non-mechanistic model (generalized linear model)
Code availability	none
**Effectiveness assessment**	
Reporting of intervention effectiveness	quantification of change in outcome values

– continued on next page –

Measure of effectiveness	active cases
Interpretation of results	implicitly causal
Reporting of uncertainty	yes
Sensitivity analysis	model specification varied
Subgroup assessment	none

**Kharroubi & Saleh 2020**	
**Study information**	
Author	Kharroubi S, Saleh F
Title	Are Lockdown Measures Effective against COVID-19?
Year	2020
Journal	Frontiers in Public Health
DOI	10.3389/fpubh.2020.549692
Date received	2020-04-09
Date accepted	2020-09-09
**Study setting**	
Number of populations included	single
Level of populations included	national (Lebanon)
Geographic areas covered	Middle East and Africa
Number of countries covered	single country (Lebanon)
Study period	start and end date span first epidemic wave
**Outcome**	
Raw outcome	epidemiological population-level outcome (cases)
Time resolution of raw outcome	daily

– continued on next page –

Computed outcome	none
Method to obtain the computed outcome	– not applicable –
Data source	data from (sub)national authorities
Data availability	data access via source
**Intervention**	
Terminology for interventions	measures
Terminology for the specific type of non-pharmaceutical interventions	containment
Exposure types	one combination of interventions (lockdown decreed on March 15 (full lockdown, shutting down airport, imposing travel restrictions and completely sealing the borders))
Types of single interventions	– not applicable –
Coding of interventions	not necessary
Source of intervention data	– could not be evaluated –
Availability of data on exposure	raw data documented in the manuscript
**Methodological approach**	
Empirical approach	parametric
Use of exposure variation	only variation over time for a single population
Method	non-mechanistic model (generalized linear model)
Code availability	none
**Effectiveness assessment**	
Reporting of intervention effectiveness	quantification of change in outcome values
Measure of effectiveness	cases
Interpretation of results	implicitly causal

– continued on next page –

Reporting of uncertainty	no
Sensitivity analysis	none
Subgroup assessment	none

**Kim et al. 2020a**	
**Study information**	
Author	Kim YJ, Seo MH, Yeom HE
Title	Estimating a Breakpoint in the Pattern of Spread of COVID-19 in South Korea
Year	2020
Journal	International Journal of Infectious Diseases
DOI	10.1016/j.ijid.2020.06.055
Date received	2020-05-05
Date accepted	2020-06-16
**Study setting**	
Number of populations included	single
Level of populations included	national (South Korea)
Geographic areas covered	Asia
Number of countries covered	single country (South Korea)
Study period	start and end date span first epidemic wave
**Outcome**	
Raw outcome	epidemiological population-level outcome (cases)
Time resolution of raw outcome	daily
Computed outcome	none

– continued on next page –

Method to obtain the computed outcome	– not applicable –
Data source	data from (sub)national authorities
Data availability	data access via source
**Intervention**	
Terminology for interventions	interventions
Terminology for the specific type of non-pharmaceutical interventions	public health
Exposure types	all interventions together
Types of single interventions	– not applicable –
Coding of interventions	– not applicable –
Source of intervention data	– not applicable –
Availability of data on exposure	– not applicable –
**Methodological approach**	
Empirical approach	parametric
Use of exposure variation	only variation over time for a single population
Method	mechanistic model (compartmental single-population transmission model)
Latent variable functionally linked to intervention	transmission rate
Code availability	none
**Effectiveness assessment**	
Reporting of intervention effectiveness	quantification of change in outcome values
Measure of effectiveness	transmission rate
Interpretation of results	associative

– continued on next page –

Reporting of uncertainty	no
Sensitivity analysis	epidemiological parameters varied
Subgroup assessment	none

**Kim et al. 2020b**	
**Study information**	
Author	Kim S, Ko Y, Kim YJ, Jung E
Title	The Impact of Social Distancing and Public Behavior Changes on COVID-19 Transmission Dynamics in the Republic of Korea
Year	2020
Journal	PLOS ONE
DOI	10.1371/journal.pone.0238684
Date received	2020-05-01
Date accepted	2020-08-22
**Study setting**	
Number of populations included	single
Level of populations included	national (Korea)
Geographic areas covered	Asia
Number of countries covered	single country (Korea)
Study period	start and end date span first epidemic wave
**Outcome**	
Raw outcome	epidemiological population-level outcome (cases)
Time resolution of raw outcome	daily
Computed outcome	none

– continued on next page –

Method to obtain the computed outcome	– not applicable –
Data source	data from (sub)national authorities
Data availability	data access via source
**Intervention**	
Terminology for interventions	interventions
Terminology for the specific type of non-pharmaceutical interventions	non-pharmaceutical
Exposure types	all interventions together
Types of single interventions	– not applicable –
Coding of interventions	– not applicable –
Source of intervention data	– not applicable –
Availability of data on exposure	– not applicable –
**Methodological approach**	
Empirical approach	parametric
Use of exposure variation	only variation over time for a single population
Method	mechanistic model (compartmental single-population transmission model)
Latent variable functionally linked to intervention	other (behavior change rate: transition of susceptibles to compartment with lower transmission rate)
Code availability	none
**Effectiveness assessment**	
Reporting of intervention effectiveness	quantification of change in outcome values
Measure of effectiveness	behavior change rate
Interpretation of results	implicitly causal

– continued on next page –

Reporting of uncertainty	no
Sensitivity analysis	none
Subgroup assessment	none

**Klimek-Tulwin & Tulwin 2020**	
**Study information**	
Author	Klimek-Tulwin M, Tulwin T
Title	Early School Closures Can Reduce the First-Wave of the COVID-19 Pandemic Development
Year	2020
Journal	Journal of Public Health
DOI	10.1007/s10389-020-01391-z
Date received	2020-05-30
Date accepted	2020-10-01
**Study setting**	
Number of populations included	multiple
Level of populations included	national (15 countries)
Geographic areas covered	Asia, Central and South America, Europe
Number of countries covered	multiple countries
Study period	end date could not be evaluated
**Outcome**	
Raw outcome	epidemiological population-level outcome (cases)
Time resolution of raw outcome	daily
Computed outcome	none

– continued on next page –

Method to obtain the computed outcome	– not applicable –
Data source	data from publicly available cross-country selections (Worldometer)
Data availability	data access via source
**Intervention**	
Terminology for interventions	interventions
Terminology for the specific type of non-pharmaceutical interventions	government
Exposure types	one single intervention (school closure (relative timing of school closure, i.e. incidence at date of closure))
Types of single interventions	school closure
Coding of interventions	necessary
Source of intervention data	use of externally coded data (UNESCO Institute for Statistics Database)
Availability of data on exposure	access to externally coded data via source
**Methodological approach**	
Empirical approach	parametric
Use of exposure variation	only variation between populations
Method	non-mechanistic model (correlation)
Code availability	none
**Effectiveness assessment**	
Reporting of intervention effectiveness	quantification of change in outcome values
Measure of effectiveness	cases
Interpretation of results	associative
Reporting of uncertainty	yes

– continued on next page –

Sensitivity analysis	none
Subgroup assessment	none

**Koh et al. 2020 (Analysis 1)**	
**Study information**	
Author	Koh WC, Naing L, Wong J
Title	Estimating the Impact of Physical Distancing Measures in Containing COVID-19: An Empirical Analysis
Year	2020
Journal	International Journal of Infectious Diseases
DOI	10.1016/j.ijid.2020.08.026
Date received	2020-06-16
Date accepted	2020-08-08
**Study setting**	
Number of populations included	multiple
Level of populations included	national (142 countries worldwide)
Geographic areas covered	Europe, Middle East and Africa, Asia, Oceania, Central and South America, North America
Number of countries covered	multiple countries
Study period	end date could not be evaluated, other (start at date of 100th case)
**Outcome**	
Raw outcome	epidemiological population-level outcome (cases)
Time resolution of raw outcome	daily

– continued on next page –

Computed outcome	measure of epidemic trend (growth rate), epidemiological parameter (reproduction number)
Method to obtain the computed outcome	statistical estimation of reproduction number (Cori et al.), simple computation (growth rate)
Data source	data from publicly available cross-country selections (ECDC (cases), JHU (cases))
Data availability	data access via source
**Intervention**	
Terminology for interventions	measures
Terminology for the specific type of non-pharmaceutical interventions	social distancing
Exposure types	multiple separate interventions (school closure, bans of large gatherings, bans of small gatherings, workplace closure, stay-at-home order, international travel restrictions, internal movement restrictions, public transport closure)
Types of single interventions	school closure, bans of large gatherings, bans of small gatherings, workplace closure, stay-at-home order, international travel restrictions, internal movement restrictions, public transport closure
Coding of interventions	necessary
Source of intervention data	use of externally coded data (OxCGRT)
Availability of data on exposure	access to externally coded data via source
**Methodological approach**	
Empirical approach	parametric
Use of exposure variation	only variation between populations
Method	non-mechanistic model (generalized linear model)
Code availability	none
**Effectiveness assessment**	

– continued on next page –

Reporting of intervention effectiveness	quantification of change in outcome values
Measure of effectiveness	reproduction number, growth rate
Interpretation of results	implicitly causal
Reporting of uncertainty	yes
Sensitivity analysis	model specification varied
Subgroup assessment	none

Koh et al. 2020 (Analysis 2)

**Study information**	
Author	Koh WC, Naing L, Wong J
Title	Estimating the Impact of Physical Distancing Measures in Containing COVID-19: An Empirical Analysis
Year	2020
Journal	International Journal of Infectious Diseases
DOI	10.1016/j.ijid.2020.08.026
Date received	2020-06-16
Date accepted	2020-08-08
**Study setting**	
Number of populations included	multiple
Level of populations included	national (142 countries worldwide)
Geographic areas covered	Europe, Middle East and Africa, Asia, Oceania, Central and South America, North America
Number of countries covered	multiple countries
Study period	end date could not be evaluated, other (start at date of 100th case)

– continued on next page –

**Outcome**	
Raw outcome	epidemiological population-level outcome (cases)
Time resolution of raw outcome	daily
Computed outcome	measure of epidemic trend (growth rate), epidemiological parameter (reproduction number)
Method to obtain the computed outcome	statistical estimation of reproduction number (Cori et al.), simple computation (growth rate)
Data source	data from publicly available cross-country selections (ECDC (cases), JHU (cases))
Data availability	data access via source
**Intervention**	
Terminology for interventions	measures
Terminology for the specific type of non-pharmaceutical interventions	social distancing
Exposure types	stringency index (OxCGRT Stringency Index)
Types of single interventions	– not applicable –
Coding of interventions	necessary
Source of intervention data	use of externally coded data (OxCGRT)
Availability of data on exposure	access to externally coded data via source
**Methodological approach**	
Empirical approach	parametric
Use of exposure variation	only variation between populations
Method	non-mechanistic model (generalized linear model)
Code availability	none
**Effectiveness assessment**	

– continued on next page –

Reporting of intervention effectiveness	quantification of change in outcome values
Measure of effectiveness	reproduction number, growth rate
Interpretation of results	implicitly causal
Reporting of uncertainty	yes
Sensitivity analysis	model specification varied
Subgroup assessment	none

Koh et al. 2020 (Analysis 3, mobility as exposure)

**Study information**	
Author	Koh WC, Naing L, Wong J
Title	Estimating the Impact of Physical Distancing Measures in Containing COVID-19: An Empirical Analysis
Year	2020
Journal	International Journal of Infectious Diseases
DOI	10.1016/j.ijid.2020.08.026
Date received	16.06.2020
Date accepted	08.08.2020
**Study setting**	
Number of populations included	multiple
Level of populations included	national (142 countries worldwide)
Geographic areas covered	Europe, Middle East and Africa, Asia, Oceania, Central and South America, North America
Number of countries covered	multiple countries
Study period	end date could not be evaluated, other (start at date of 100th case)

– continued on next page –

**Outcome**	
Raw outcome	epidemiological population-level outcome (cases)
Time resolution of raw outcome	daily
Computed outcome	epidemiological parameter (reproduction number), measure of epidemic trend (growth rate)
Method to obtain the computed outcome	statistical estimation of reproduction number (Cori et al.), simple computation (growth rate)
Data source	data from publicly available cross-country selections (ECDC, JHU)
Data availability	data access via source
**Intervention**	
Terminology for interventions	measures
Terminology for the specific type of non-pharmaceutical interventions	social distancing
Exposure types	mobility (time spent in various locations)
Types of single interventions	– not applicable –
Coding of interventions	– not applicable –
Source of intervention data	mobility data by corporate organizations (Google)
Availability of data on exposure	data access via source
**Methodological approach**	
Empirical approach	parametric
Use of exposure variation	only variation between populations
Method	non-mechanistic model (generalized linear model)
Code availability	none
**Effectiveness assessment**	
Reporting of intervention effectiveness	quantification of change in outcome values

– continued on next page –

Measure of effectiveness	reproduction number, growth rate
Interpretation of results	implicitly causal
Reporting of uncertainty	yes
Sensitivity analysis	none
Subgroup assessment	none

Kraemer et al. 2020 (Analysis 1)

**Study information**	
Author	Kraemer MU, Yang CH, Gutierrez B, Wu CH, Klein B, Pigott DM, du Plessis L, Faria NR, Li R, Hanage WP, Brownstein JS, Layan M, Vespignani A, Tian H, Dye C, Pybus OG, And SV
Title	The Effect of Human Mobility and Control Measures on the COVID-19 Epidemic in China
Year	2020
Journal	Science
DOI	10.1126/science.abb4218
Date received	2020-03-03
Date accepted	2020-03-24
**Study setting**	
Number of populations included	multiple
Level of populations included	both national and subnational (China and its provinces)
Geographic areas covered	Asia
Number of countries covered	single country (China)
Study period	start and end date span first epidemic wave: behavioral

– continued on next page –

**Outcome**	
Raw outcome	behavioral outcome (mobility: migration from Wuhan)
Time resolution of raw outcome	daily
Computed outcome	none
Method to obtain the computed outcome	– not applicable –
Data source	mobility data from corporate organizations (Baidu)
Data availability	data made available by the authors
**Intervention**	
Terminology for interventions	measures
Terminology for the specific type of non-pharmaceutical interventions	control
Exposure types	one combination of interventions (Wuhan City Lockdown)
Types of single interventions	– not applicable –
Coding of interventions	not necessary
Source of intervention data	– could not be evaluated –
Availability of data on exposure	raw data documented in the manuscript
**Methodological approach**	
Empirical approach	descriptive
Use of exposure variation	only variation over time for multiple populations
Method	description of change over time (description of time course)
Code availability	publicly available
**Effectiveness assessment**	
Reporting of intervention effectiveness	qualitative statement
Measure of effectiveness	mobility: migration from Wuhan

– continued on next page –

Interpretation of results	implicitly causal
Reporting of uncertainty	– not applicable –
Sensitivity analysis	none
Subgroup assessment	none

**Kraemer et al. 2020 (Analysis 2)**	
**Study information**	
Author	Kraemer MU, Yang CH, Gutierrez B, Wu CH, Klein B, Pigott DM, du Plessis L, Faria NR, Li R, Hanage WP, Brownstein JS, Layan M, Vespignani A, Tian H, Dye C, Pybus OG, And SV
Title	The Effect of Human Mobility and Control Measures on the COVID-19 Epidemic in China
Year	2020
Journal	Science
DOI	10.1126/science.abb4218
Date received	2020-03-03
Date accepted	2020-03-24
**Study setting**	
Number of populations included	multiple
Level of populations included	both national and subnational (China and its provinces)
Geographic areas covered	Asia
Number of countries covered	single country (China)
Study period	start and end date span first epidemic wave
**Outcome**	
Raw outcome	epidemiological population-level outcome (cases)

– continued on next page –

Time resolution of raw outcome	daily
Computed outcome	measure of epidemic trend (growth rate)
Method to obtain the computed outcome	other (time series model)
Data source	data from (sub)national authorities
Data availability	data made available by the authors
**Intervention**	
Terminology for interventions	measures
Terminology for the specific type of non-pharmaceutical interventions	control
Exposure types	one combination of interventions (Wuhan City Lockdown)
Types of single interventions	– not applicable –
Coding of interventions	not necessary
Source of intervention data	– could not be evaluated –
Availability of data on exposure	raw data documented in the manuscript
**Methodological approach**	
Empirical approach	descriptive
Use of exposure variation	only variation over time for multiple populations
Method	description of change over time (comparison of time periods)
Code availability	publicly available
**Effectiveness assessment**	
Reporting of intervention effectiveness	comparison of outcome values
Measure of effectiveness	growth rate
Interpretation of results	implicitly causal
Reporting of uncertainty	yes

– continued on next page –

**Kraemer et al. 2020 (Analysis 2)** – continued from previous page	
Sensitivity analysis	none
Subgroup assessment	none

**Kraemer et al. 2020 (Analysis 3, mobility as exposure)**	
**Study information**	
Author	Kraemer MU, Yang CH, Gutierrez B, Wu CH, Klein B, Pigott DM, du Plessis L, Faria NR, Li R, Hanage WP, Brownstein JS, Layan M, Vespignani A, Tian H, Dye C, Pybus OG, And SV
Title	The Effect of Human Mobility and Control Measures on the COVID-19 Epidemic in China
Year	2020
Journal	Science
DOI	10.1126/science.abb4218
Date received	03.03.2020
Date accepted	24.03.2020
**Study setting**	
Number of populations included	multiple
Level of populations included	both national and subnational (China and its provinces)
Geographic areas covered	Asia
Number of countries covered	single country (China)
Study period	start and end date span first epidemic wave
**Outcome**	
Raw outcome	epidemiological population-level outcome (cases)
Time resolution of raw outcome	daily
Computed outcome	none

– continued on next page –

Method to obtain the computed outcome	– not applicable –
Data source	data from (sub)national authorities
Data availability	data made available by the authors
**Intervention**	
Terminology for interventions	measures
Terminology for the specific type of non-pharmaceutical interventions	control
Exposure types	mobility (migration from Wuhan)
Types of single interventions	– not applicable –
Coding of interventions	– not applicable –
Source of intervention data	mobility data by corporate organizations (Baidu)
Availability of data on exposure	data access via source
**Methodological approach**	
Empirical approach	descriptive
Use of exposure variation	only variation over time for multiple populations
Method	non-mechanistic model (correlation)
Code availability	publicly available
**Effectiveness assessment**	
Reporting of intervention effectiveness	quantification of change in outcome values
Measure of effectiveness	cases
Interpretation of results	implicitly causal
Reporting of uncertainty	no
Sensitivity analysis	none
Subgroup assessment	none

Krishna 2020	
**Study information**	
Author	Krishna MV
Title	Mathematical Modelling on Diffusion and Control of COVID–19
Year	2020
Journal	Infectious Disease Modelling
DOI	10.1016/j.idm.2020.08.009
Date received	2020-06-26
Date accepted	2020-08-14
**Study setting**	
Number of populations included	single
Level of populations included	subnational (Wuhan)
Geographic areas covered	Asia
Number of countries covered	single country (China)
Study period	end date in growth phase of wave
**Outcome**	
Raw outcome	epidemiological population-level outcome (cases)
Time resolution of raw outcome	daily
Computed outcome	epidemiological parameter (reproduction number)
Method to obtain the computed outcome	compartmental transmission model (single-population)
Data source	– could not be evaluated –
Data availability	data not accessible
**Intervention**	
Terminology for interventions	measures

– continued on next page –

Terminology for the specific type of non-pharmaceutical interventions	control
Exposure types	one combination of interventions (Wuhan City Lockdown)
Types of single interventions	– not applicable –
Coding of interventions	not necessary
Source of intervention data	– could not be evaluated –
Availability of data on exposure	raw data documented in the manuscript
**Methodological approach**	
Empirical approach	descriptive
Use of exposure variation	only variation over time for a single population
Method	description of change over time (comparison of time periods)
Code availability	none
**Effectiveness assessment**	
Reporting of intervention effectiveness	comparison of outcome values
Measure of effectiveness	reproduction number
Interpretation of results	associative
Reporting of uncertainty	yes
Sensitivity analysis	none
Subgroup assessment	none

Kucharski et al. 2020

Study information

– continued on next page –

Author	Kucharski AJ, Russell TW, Diamond C, Liu Y, Edmunds J, Funk S, Eggo RM, Sun F, Jit M, Munday JD, Davies N, Gimma A, van Zandvoort K, Gibbs H, Hellewell J, Jarvis CI, Clifford S, Quilty BJ, Bosse NI, Abbott S, Klepac P, Flasche S
Title	Early Dynamics of Transmission and Control of COVID-19: A Mathematical Modelling Study
Year	2020
Journal	The Lancet Infectious Diseases
DOI	10.1016/s1473-3099(20)30144-4
Date received	– could not be evaluated –
Date accepted	2020-03-11 (published)
**Study setting**	
Number of populations included	single
Level of populations included	subnational (Wuhan)
Geographic areas covered	Asia
Number of countries covered	single country (China)
Study period	start and end date span first epidemic wave
**Outcome**	
Raw outcome	epidemiological population-level outcome (cases)
Time resolution of raw outcome	daily
Computed outcome	epidemiological parameter (reproduction number)
Method to obtain the computed outcome	compartmental transmission model (metapopulation)
Data source	data from publicly available cross-country selections (WHO: exported cases), data from other research projects (study by Huang et al. 2020: cases in Wuhan, study by Liu et al. 2020: cases in China), data from public media outlets (varous data from public media outletss: prevalence of infection on evacuation flights)

– continued on next page –

Data availability	data made available by the authors
**Intervention**	
Terminology for interventions	measures
Terminology for the specific type of non-pharmaceutical interventions	travel control
Exposure types	one single intervention (travel restrictions (introduced on Jan 23))
Types of single interventions	international travel restrictions
Coding of interventions	not necessary
Source of intervention data	– could not be evaluated –
Availability of data on exposure	raw data documented in the manuscript
**Methodological approach**	
Empirical approach	descriptive
Use of exposure variation	only variation over time for a single population
Method	description of change over time (comparison of time periods)
Code availability	publicly available
**Effectiveness assessment**	
Reporting of intervention effectiveness	comparison of outcome values
Measure of effectiveness	reproduction number
Interpretation of results	associative
Reporting of uncertainty	yes
Sensitivity analysis	different or modified outcome used (different confirmed case data), epidemiological parameters varied
Subgroup assessment	none

Kumar et al. 2020	
**Study information**	
Author	Kumar V, Sood A, Gupta S, Sood N
Title	Prevention- versus Promotion-Focus Regulatory Efforts on the Disease Incidence and Mortality of COVID-19: A Multinational Diffusion Study Using Functional Data Analysis
Year	2020
Journal	Journal of International Marketing
DOI	10.1177/1069031x20966563
Date received	– could not be evaluated –
Date accepted	2020-12-14 (published)
**Study setting**	
Number of populations included	multiple
Level of populations included	national (70 countries around the globe)
Geographic areas covered	Europe, Middle East and Africa, Asia, Oceania, Central and South America, North America
Number of countries covered	multiple countries
Study period	start and end date span first epidemic wave
**Outcome**	
Raw outcome	epidemiological population-level outcome (cases)
Time resolution of raw outcome	daily
Computed outcome	none
Method to obtain the computed outcome	– not applicable –
Data source	data from publicly available cross-country selections (ECDC)
Data availability	data access via source
**Intervention**	

– continued on next page –

Terminology for interventions	interventions
Terminology for the specific type of non-pharmaceutical interventions	non-pharmaceutical
Exposure types	categories of interventions (containment and closure policies aggregated via principal component analysis based on individual measures (shelter-in-place orders, workplace closure, mobility restrictions, gathering bans, school closure, mask-mandates, time-based curfews, partial lockdown))
Types of single interventions	– not applicable –
Coding of interventions	necessary
Source of intervention data	use of externally coded data (OxCGRT)
Availability of data on exposure	access to externally coded data via source
**Methodological approach**	
Empirical approach	parametric
Use of exposure variation	both variation over time and between populations
Method	non-mechanistic model (generalized linear model)
Code availability	none
**Effectiveness assessment**	
Reporting of intervention effectiveness	quantification of change in outcome values
Measure of effectiveness	cases
Interpretation of results	implicitly causal
Reporting of uncertainty	yes
Sensitivity analysis	none
Subgroup assessment	epidemiological indicators (pre-intervention epidemic trend), socioeconomic indicators (population density, age, health care occupation), public health response (subject to other measures)

Kuo & Fu 2021	
**Study information**	
Author	Kuo CP, Fu JS
Title	Evaluating the Impact of Mobility on COVID-19 Pandemic with Machine Learning Hybrid Predictions
Year	2021
Journal	Science of The Total Environment
DOI	10.1016/j.scitotenv.2020.144151
Date received	2020-09-07
Date accepted	2020-11-24
**Study setting**	
Number of populations included	multiple
Level of populations included	both national and subnational (172 US counties)
Geographic areas covered	North America
Number of countries covered	single country (US)
Study period	start and end date span first epidemic wave: behavioral
**Outcome**	
Raw outcome	behavioral outcome (mobility: mobility and engagement index)
Time resolution of raw outcome	daily
Computed outcome	none
Method to obtain the computed outcome	– not applicable –
Data source	data from (sub)national authorities (Federal Reserve Bank of Dallas)
Data availability	data access via source
**Intervention**	
Terminology for interventions	measures

– continued on next page –

Terminology for the specific type of non-pharmaceutical interventions	lockdown, control
Exposure types	one single intervention (stay-at-home order)
Types of single interventions	stay-at-home order
Coding of interventions	necessary
Source of intervention data	use of externally coded data (New York Times)
Availability of data on exposure	access to externally coded data via source
**Methodological approach**	
Empirical approach	descriptive
Use of exposure variation	only variation over time for multiple populations
Method	description of change over time (comparison of time periods)
Code availability	none
**Effectiveness assessment**	
Reporting of intervention effectiveness	quantification of change in outcome values
Measure of effectiveness	mobility: mobility and engagement index
Interpretation of results	associative
Reporting of uncertainty	no
Sensitivity analysis	none
Subgroup assessment	none

Lau et al. 2020 (Analysis 1)

**Study information**	

– continued on next page –

Author	Lau H, Khosrawipour V, Kocbach P, Mikolajczyk A, Schubert J, Bania J, Khosrawipour T
Title	The Positive Impact of Lockdown in Wuhan on Containing the COVID-19 Outbreak in China
Year	2020
Journal	Journal of Travel Medicine
DOI	10.1093/jtm/taaa037
Date received	2020-02-25
Date accepted	2020-03-12
**Study setting**	
Number of populations included	single
Level of populations included	national (China)
Geographic areas covered	Asia
Number of countries covered	single country (China)
Study period	end date at peak of wave
**Outcome**	
Raw outcome	epidemiological population-level outcome (cases)
Time resolution of raw outcome	daily
Computed outcome	measure of epidemic trend (doubling time)
Method to obtain the computed outcome	exponential growth model
Data source	data from publicly available cross-country selections (WHO)
Data availability	data access via source
**Intervention**	
Terminology for interventions	measures

– continued on next page –

Terminology for the specific type of non-pharmaceutical interventions	lockdown
Exposure types	one combination of interventions (Wuhan City Lockdown)
Types of single interventions	– not applicable –
Coding of interventions	not necessary
Source of intervention data	– could not be evaluated –
Availability of data on exposure	raw data documented in the manuscript
**Methodological approach**	
Empirical approach	descriptive
Use of exposure variation	only variation over time for a single population
Method	description of change over time (comparison of time periods)
Code availability	none
**Effectiveness assessment**	
Reporting of intervention effectiveness	comparison of outcome values
Measure of effectiveness	doubling time
Interpretation of results	implicitly causal
Reporting of uncertainty	yes
Sensitivity analysis	none
Subgroup assessment	none

Lau et al. 2020 (Analysis 2)

**Study information**	
Author	Lau H, Khosrawipour V, Kocbach P, Mikolajczyk A, Schubert J, Bania J, Khosrawipour T

– continued on next page –

Title	The Positive Impact of Lockdown in Wuhan on Containing the COVID-19 Outbreak in China
Year	2020
Journal	Journal of Travel Medicine
DOI	10.1093/jtm/taaa037
Date received	2020-02-25
Date accepted	2020-03-12
**Study setting**	
Number of populations included	single
Level of populations included	national (China)
Geographic areas covered	Asia
Number of countries covered	single country (China)
Study period	end date at peak of wave
**Outcome**	
Raw outcome	epidemiological population-level outcome (cases), behavioral outcome (mobility: domestic air traffic)
Time resolution of raw outcome	daily
Computed outcome	other (correlation between cases and mobility)
Method to obtain the computed outcome	other (correlation analysis)
Data source	data from publicly available cross-country selections (WHO: cases), data from (sub)national authorities (Civil Aviation Administration of China: flight data)
Data availability	data access via source
**Intervention**	
Terminology for interventions	measures

– continued on next page –

Terminology for the specific type of non-pharmaceutical interventions	lockdown
Exposure types	one combination of interventions (Wuhan City Lockdown)
Types of single interventions	– not applicable –
Coding of interventions	not necessary
Source of intervention data	– could not be evaluated –
Availability of data on exposure	raw data documented in the manuscript
**Methodological approach**	
Empirical approach	descriptive
Use of exposure variation	only variation over time for a single population
Method	description of change over time (comparison of time periods)
Code availability	none
**Effectiveness assessment**	
Reporting of intervention effectiveness	comparison of outcome values
Measure of effectiveness	correlation between cases and mobility
Interpretation of results	implicitly causal
Reporting of uncertainty	yes
Sensitivity analysis	none
Subgroup assessment	none

Lee et al. 2020

**Study information**	
Author	Lee M, Zhao J, Sun Q, Pan Y, Zhou W, Xiong C, Zhang L

– continued on next page –

Title	Human Mobility Trends during the Early Stage of the COVID-19 Pandemic in the United States
Year	2020
Journal	PLOS ONE
DOI	10.1371/journal.pone.0241468
Date received	2020-05-29
Date accepted	2020-10-15
**Study setting**	
Number of populations included	multiple
Level of populations included	both national and subnational (50 US states)
Geographic areas covered	North America
Number of countries covered	single country (US)
Study period	start and end date span first epidemic wave: behavioral
**Outcome**	
Raw outcome	behavioral outcome (mobility: percentage staying home & distance traveled & percentage out-of-county trips)
Time resolution of raw outcome	daily
Computed outcome	none
Method to obtain the computed outcome	– not applicable –
Data source	data from other research projects (University of Maryland COVID-19 Impact Analysis Platform)
Data availability	data access via source
**Intervention**	
Terminology for interventions	interventions

– continued on next page –

Terminology for the specific type of non-pharmaceutical interventions	non-pharmaceutical
Exposure types	one single intervention (national analysis: national emergency declaration, state analysis: stay-at-home order)
Types of single interventions	declaration of a state of emergency, stay-at-home order
Coding of interventions	necessary
Source of intervention data	– could not be evaluated –
Availability of data on exposure	coded data not available
**Methodological approach**	
Empirical approach	descriptive
Use of exposure variation	only variation over time for multiple populations
Method	description of change over time (comparison of time periods)
Code availability	none
**Effectiveness assessment**	
Reporting of intervention effectiveness	quantification of change in outcome values
Measure of effectiveness	mobility: percentage staying home & distance traveled & percentage out-of-county trips
Interpretation of results	associative
Reporting of uncertainty	no
Sensitivity analysis	none
Subgroup assessment	socioeconomic indicators (income)

Lei et al. 2020	
**Study information**	
Author	Lei H, Wu X, Wang X, Xu M, Xie Y, Du X, Cowling BJ, Li Y, Shu Y
Title	Different Transmission Dynamics of Coronavirus Disease 2019 (COVID-19) and Influenza Suggest the Relative Efficiency of Isolation/Quarantine and Social Distancing against COVID-19 in China
Year	2020
Journal	Clinical Infectious Diseases
DOI	10.1093/cid/ciaa1584
Date received	2020-09-04
Date accepted	2020-10-12
**Study setting**	
Number of populations included	single
Level of populations included	national (China)
Geographic areas covered	Asia
Number of countries covered	single country (China)
Study period	start and end date span first epidemic wave
**Outcome**	
Raw outcome	epidemiological population-level outcome (cases, surrogate disease: cases influenza)
Time resolution of raw outcome	daily
Computed outcome	epidemiological parameter (reproduction number, reproduction number: influenza)
Method to obtain the computed outcome	statistical estimation of reproduction number (Cori et al.)
Data source	data from publicly available cross-country selections (WHO (provided by Chinese National Influenza Center))
Data availability	data access via source

– continued on next page –

**Intervention**	
Terminology for interventions	interventions
Terminology for the specific type of non-pharmaceutical interventions	non-pharmaceutical
Exposure types	one combination of interventions (Wuhan City Lockdown)
Types of single interventions	– not applicable –
Coding of interventions	not necessary
Source of intervention data	– could not be evaluated –
Availability of data on exposure	raw data documented in the manuscript
**Methodological approach**	
Empirical approach	parametric
Use of exposure variation	only variation over time for a single population
Method	non-mechanistic model (generalized linear model)
Code availability	none
**Effectiveness assessment**	
Reporting of intervention effectiveness	quantification of change in outcome values
Measure of effectiveness	reproduction number, reproduction number: influenza
Interpretation of results	associative
Reporting of uncertainty	yes
Sensitivity analysis	none
Subgroup assessment	none

Lemaitre et al. 2020 (Analysis 1)	
**Study information**	
Author	Lemaitre JC, Perez-Saez J, Azman AS, Rinaldo A, Fellay J
Title	Assessing the Impact of Non-Pharmaceutical Interventions on SARS-CoV-2 Transmission in Switzerland
Year	2020
Journal	Swiss Medical Weekly
DOI	10.4414/smw.2020.20295
Date received	– could not be evaluated –
Date accepted	2020-05-30 (published)
**Study setting**	
Number of populations included	multiple
Level of populations included	both national and subnational (Swiss cantons)
Geographic areas covered	Europe
Number of countries covered	single country (Switzerland)
Study period	start and end date span first epidemic wave
**Outcome**	
Raw outcome	epidemiological population-level outcome (hospitalizations, deaths), behavioral outcome (mobility: time spent in various locations)
Time resolution of raw outcome	daily
Computed outcome	epidemiological parameter (reproduction number), change points (change points: reproduction number, change points: mobility)
Method to obtain the computed outcome	compartmental transmission model (single-population), change point analysis
Data source	mobility data from corporate organizations (Google)
Data availability	data made available by the authors
**Intervention**	

– continued on next page –

Terminology for interventions	interventions
Terminology for the specific type of non-pharmaceutical interventions	non-pharmaceutical
Exposure types	one single intervention (school closure)
Types of single interventions	school closure
Coding of interventions	not necessary
Source of intervention data	– could not be evaluated –
Availability of data on exposure	raw data documented in the manuscript
**Methodological approach**	
Empirical approach	descriptive
Use of exposure variation	only variation over time for multiple populations
Method	comparison of change points with intervention
Code availability	publicly available
**Effectiveness assessment**	
Reporting of intervention effectiveness	quantification of change in outcome values
Measure of effectiveness	change points: reproduction number, change points: mobility
Interpretation of results	associative
Reporting of uncertainty	no
Sensitivity analysis	none
Subgroup assessment	none

Lemaitre et al. 2020 (Analysis 2)

**Study information**	
Author	Lemaitre JC, Perez-Saez J, Azman AS, Rinaldo A, Fellay J

– continued on next page –

Title	Assessing the Impact of Non-Pharmaceutical Interventions on SARS-CoV-2 Transmission in Switzerland
Year	2020
Journal	Swiss Medical Weekly
DOI	10.4414/smw.2020.20295
Date received	– could not be evaluated –
Date accepted	2020-05-30 (published)
**Study setting**	
Number of populations included	multiple
Level of populations included	both national and subnational (Swiss cantons)
Geographic areas covered	Europe
Number of countries covered	single country (Switzerland)
Study period	start and end date span first epidemic wave
**Outcome**	
Raw outcome	epidemiological population-level outcome (hospitalizations, deaths)
Time resolution of raw outcome	daily
Computed outcome	epidemiological parameter (reproduction number), change points (change points: reproduction number)
Method to obtain the computed outcome	compartmental transmission model (single-population), change point analysis
Data source	data from publicly available open-source projects (openZH)
Data availability	data made available by the authors
**Intervention**	
Terminology for interventions	interventions
Terminology for the specific type of non-pharmaceutical interventions	non-pharmaceutical

– continued on next page –

Exposure types	multiple separate interventions (bans of small gatherings, school closure)
Types of single interventions	bans of small gatherings, school closure
Coding of interventions	not necessary
Source of intervention data	– could not be evaluated –
Availability of data on exposure	raw data documented in the manuscript
**Methodological approach**	
Empirical approach	descriptive
Use of exposure variation	only variation over time for multiple populations
Method	comparison of change points with intervention
Code availability	publicly available
**Effectiveness assessment**	
Reporting of intervention effectiveness	comparison of outcome values
Measure of effectiveness	reproduction number
Interpretation of results	associative
Reporting of uncertainty	yes
Sensitivity analysis	none
Subgroup assessment	none

Lemaitre et al. 2020 (Analysis 3, mobility as exposure)

**Study information**	
Author	Lemaitre JC, Perez-Saez J, Azman AS, Rinaldo A, Fellay J
Title	Assessing the Impact of Non-Pharmaceutical Interventions on SARS-CoV-2 Transmission in Switzerland
Year	2020

– continued on next page –

Journal	Swiss Medical Weekly
DOI	10.4414/smw.2020.20295
Date received	– could not be evaluated –
Date accepted	30.05.2020 (published)
**Study setting**	
Number of populations included	multiple
Level of populations included	both national and subnational (Swiss cantons)
Geographic areas covered	Europe
Number of countries covered	single country (Switzerland)
Study period	start and end date span first epidemic wave
**Outcome**	
Raw outcome	epidemiological population-level outcome (hospitalized, deaths)
Time resolution of raw outcome	daily
Computed outcome	epidemiological parameter (reproduction number)
Method to obtain the computed outcome	compartmental transmission model (single-population)
Data source	data from publicly available open-source projects (openZH)
Data availability	data made available by the authors
**Intervention**	
Terminology for interventions	interventions
Terminology for the specific type of non-pharmaceutical interventions	non-pharmaceutical
Exposure types	mobility (time spent in various locations)
Types of single interventions	– not applicable –
Coding of interventions	– not applicable –
Source of intervention data	mobility data by corporate organizations (Google)

– continued on next page –

Availability of data on exposure	data access via source
**Methodological approach**	
Empirical approach	descriptive
Use of exposure variation	only variation over time for multiple populations
Method	non-mechanistic model (correlation)
Code availability	publicly available
**Effectiveness assessment**	
Reporting of intervention effectiveness	quantification of change in outcome values
Measure of effectiveness	reproduction number
Interpretation of results	associative
Reporting of uncertainty	no
Sensitivity analysis	none
Subgroup assessment	none

Li et al. 2020a

**Study information**	
Author	Li Y, Zhang R, Zhao J, Molina MJ
Title	Understanding Transmission and Intervention for the COVID-19 Pandemic in the United States
Year	2020
Journal	Science of The Total Environment
DOI	10.1016/j.scitotenv.2020.141560
Date received	2020-07-18

– continued on next page –

Date accepted	2020-08-15
**Study setting**	
Number of populations included	multiple
Level of populations included	subnational (15 US states)
Geographic areas covered	North America
Number of countries covered	single country (US)
Study period	same end date for several populations with diverse epidemic trajectories
**Outcome**	
Raw outcome	epidemiological population-level outcome (cases)
Time resolution of raw outcome	daily
Computed outcome	none
Method to obtain the computed outcome	– not applicable –
Data source	data from (sub)national authorities
Data availability	data access via source
**Intervention**	
Terminology for interventions	measures
Terminology for the specific type of non-pharmaceutical interventions	mitigation
Exposure types	one single intervention (mask mandate)
Types of single interventions	mask mandate
Coding of interventions	necessary
Source of intervention data	coding done by authors
Availability of data on exposure	coded data made available by the authors
**Methodological approach**	
Empirical approach	counterfactual

– continued on next page –

Use of exposure variation	only variation over time for multiple populations
Method	non-mechanistic model (exponential growth model)
Code availability	none
**Effectiveness assessment**	
Reporting of intervention effectiveness	quantification of change in outcome values
Measure of effectiveness	cases
Interpretation of results	implicitly causal
Reporting of uncertainty	no
Sensitivity analysis	none
Subgroup assessment	none

Li et al. 2020b

**Study information**	
Author	Li BZ, Cao NW, Zhou HY, Chu XJ, Ye DQ
Title	Strong Policies Control the Spread of COVID-19 in China
Year	2020
Journal	Journal of Medical Virology
DOI	10.1002/jmv.25934
Date received	2020-03-26
Date accepted	2020-04-21
**Study setting**	
Number of populations included	multiple
Level of populations included	subnational (Hubei, Guangdong, henan, Zhejiang, Hunan, Anhui)

– continued on next page –

Geographic areas covered	Asia
Number of countries covered	single country (China)
Study period	start and end date span first epidemic wave
**Outcome**	
Raw outcome	epidemiological population-level outcome (cases)
Time resolution of raw outcome	daily
Computed outcome	none
Method to obtain the computed outcome	– not applicable –
Data source	data from (sub)national authorities
Data availability	data access via source
**Intervention**	
Terminology for interventions	policies
Terminology for the specific type of non-pharmaceutical interventions	intervention
Exposure types	all interventions together
Types of single interventions	– not applicable –
Coding of interventions	– not applicable –
Source of intervention data	– not applicable –
Availability of data on exposure	– not applicable –
**Methodological approach**	
Empirical approach	counterfactual
Use of exposure variation	only variation over time for multiple populations
Method	mechanistic model (compartmental single-population transmission model)
Code availability	none
**Effectiveness assessment**	

– continued on next page –

Reporting of intervention effectiveness	comparison of outcome values
Measure of effectiveness	cases
Interpretation of results	implicitly causal
Reporting of uncertainty	no
Sensitivity analysis	none
Subgroup assessment	none

**Li et al. 2020c (Analysis 1)**	
**Study information**	
Author	Li Y, Wang LW, Peng ZH, Shen HB
Title	Basic Reproduction Number and Predicted Trends of Coronavirus Disease 2019 Epidemic in the Mainland of China
Year	2020
Journal	Infectious Diseases of Poverty
DOI	10.1186/s40249-020-00704-4
Date received	2020-03-06
Date accepted	2020-06-18
**Study setting**	
Number of populations included	multiple
Level of populations included	both national and subnational (China Hubei and outside Hubei)
Geographic areas covered	Asia
Number of countries covered	single country (China)

– continued on next page –

Study period	start and end date span first epidemic wave
**Outcome**	
Raw outcome	epidemiological population-level outcome (cases)
Time resolution of raw outcome	daily
Computed outcome	epidemiological parameter (reproduction number, transmission rate, migration rate)
Method to obtain the computed outcome	compartmental transmission model (single-population)
Data source	data from (sub)national authorities
Data availability	data access via source
**Intervention**	
Terminology for interventions	measures
Terminology for the specific type of non-pharmaceutical interventions	control
Exposure types	multiple combinations of interventions (Wuhan lockdown, Hubei lockdown, large-scale case screening)
Types of single interventions	– not applicable –
Coding of interventions	not necessary
Source of intervention data	– could not be evaluated –
Availability of data on exposure	raw data documented in the manuscript
**Methodological approach**	
Empirical approach	descriptive
Use of exposure variation	both variation over time and between populations
Method	description of change over time (comparison of time periods)
Code availability	none
**Effectiveness assessment**	

– continued on next page –

Reporting of intervention effectiveness	comparison of outcome values
Measure of effectiveness	reproduction number, transmission rate, migration rate
Interpretation of results	associative
Reporting of uncertainty	no
Sensitivity analysis	none
Subgroup assessment	none

**Li et al. 2020c (Analysis 2)**	
**Study information**	
Author	Li Y, Wang LW, Peng ZH, Shen HB
Title	Basic Reproduction Number and Predicted Trends of Coronavirus Disease 2019 Epidemic in the Mainland of China
Year	2020
Journal	Infectious Diseases of Poverty
DOI	10.1186/s40249-020-00704-4
Date received	2020-03-06
Date accepted	2020-06-18
**Study setting**	
Number of populations included	multiple
Level of populations included	both national and subnational (China Hubei and outside Hubei)
Geographic areas covered	Asia
Number of countries covered	single country (China)
Study period	start and end date span first epidemic wave
**Outcome**	

– continued on next page –

Raw outcome	epidemiological population-level outcome (cases)
Time resolution of raw outcome	daily
Computed outcome	none
Method to obtain the computed outcome	– not applicable –
Data source	data from (sub)national authorities
Data availability	data access via source
**Intervention**	
Terminology for interventions	measures
Terminology for the specific type of non-pharmaceutical interventions	control
Exposure types	multiple combinations of interventions (Wuhan lockdown, Hubei lockdown, large-scale case screening)
Types of single interventions	– not applicable –
Coding of interventions	not necessary
Source of intervention data	– could not be evaluated –
Availability of data on exposure	raw data documented in the manuscript
**Methodological approach**	
Empirical approach	counterfactual
Use of exposure variation	both variation over time and between populations
Method	mechanistic model (compartmental metapopulation transmission model)
Code availability	none
**Effectiveness assessment**	
Reporting of intervention effectiveness	comparison of outcome values
Measure of effectiveness	cases

– continued on next page –

Interpretation of results	associative
Reporting of uncertainty	no
Sensitivity analysis	none
Subgroup assessment	none

Li et al. 2021

**Study information**	
Author	Li Y, Campbell H, Kulkarni D, Harpur A, Nundy M, Wang X, Nair H
Title	The Temporal Association of Introducing and Lifting Non-Pharmaceutical Interventions with the Time-Varying Reproduction Number (R) of SARS-CoV-2: A Modelling Study across 131 Countries
Year	2021
Journal	The Lancet Infectious Diseases
DOI	10.1016/s1473-3099(20)30785-4
Date received	– could not be evaluated –
Date accepted	2020-10-22 (published)
**Study setting**	
Number of populations included	multiple
Level of populations included	national (133 countries)
Geographic areas covered	Europe, Middle East and Africa, Asia, Oceania, Central and South America, North America
Number of countries covered	multiple countries
Study period	start and end date span first epidemic wave
**Outcome**	
Raw outcome	epidemiological population-level outcome (effective reproduction number)

– continued on next page –

Time resolution of raw outcome	daily
Computed outcome	epidemiological parameter (reproduction number ratio)
Method to obtain the computed outcome	simple computation
Data source	data from other research projects (EpiForecasts project by the London School of Hygiene & Tropical Medicine or study by Abbott et al.)
Data availability	data made available by the authors
**Intervention**	
Terminology for interventions	interventions
Terminology for the specific type of non-pharmaceutical interventions	non-pharmaceutical
Exposure types	multiple separate interventions (school closure, bans of large gatherings, bans of small gatherings, workplace closure, stay-at-home order, international travel restrictions, internal movement restrictions, public transport closure)
Types of single interventions	school closure, bans of large gatherings, bans of small gatherings, workplace closure, stay-at-home order, international travel restrictions, internal movement restrictions, public transport closure
Coding of interventions	necessary
Source of intervention data	use of externally coded data (OxCGRT)
Availability of data on exposure	coded data made available by the authors
**Methodological approach**	
Empirical approach	parametric
Use of exposure variation	both variation over time and between populations
Method	non-mechanistic model (generalized linear model)
Code availability	publicly available
**Effectiveness assessment**	

– continued on next page –

Reporting of intervention effectiveness	quantification of change in outcome values
Measure of effectiveness	reproduction number ratio
Interpretation of results	associative
Reporting of uncertainty	yes
Sensitivity analysis	different coding of interventions used (mean effective reproduction number of the last day considered instead of effective reproduction number of the last day), same analysis with (sub)population(s) excluded
Subgroup assessment	none

Lim et al. 2020a (Analysis 1)

**Study information**	
Author	Lim JT, Dickens BS, Choo EL, Chew LZ, Koo JR, Tam C, Park M, Cook AR
Title	Revealing Regional Disparities in the Transmission Potential of SARS-CoV-2 from Interventions in Southeast Asia
Year	2020
Journal	Proceedings of the Royal Society B: Biological Sciences
DOI	10.1098/rspb.2020.1173
Date received	2020-05-20
Date accepted	2020-08-03
**Study setting**	
Number of populations included	multiple
Level of populations included	national (9 South-east Asian countries)

– continued on next page –

Geographic areas covered	Asia
Number of countries covered	multiple countries
Study period	same end date for several populations with diverse epidemic trajectories
**Outcome**	
Raw outcome	epidemiological population-level outcome (cases), behavioral outcome (mobility: time spent in various locations)
Time resolution of raw outcome	daily
Computed outcome	epidemiological parameter (reproduction number)
Method to obtain the computed outcome	statistical estimation of reproduction number (Cori et al., extended with regression on mobility)
Data source	data from publicly available cross-country selections (JHU: cases), mobility data from corporate organizations (Google: mobility)
Data availability	data access via source
**Intervention**	
Terminology for interventions	measures
Terminology for the specific type of non-pharmaceutical interventions	social distancing
Exposure types	all interventions together (timeline of measures)
Types of single interventions	– not applicable –
Coding of interventions	– not applicable –
Source of intervention data	– not applicable –
Availability of data on exposure	– not applicable –
**Methodological approach**	
Empirical approach	descriptive
Use of exposure variation	only variation over time for multiple populations
Method	description of change over time (comparison of time periods)

– continued on next page –

Code availability	none
**Effectiveness assessment**	
Reporting of intervention effectiveness	comparison of outcome values
Measure of effectiveness	reproduction number, cases, mobility: time spent in various locations
Interpretation of results	implicitly causal
Reporting of uncertainty	yes
Sensitivity analysis	none
Subgroup assessment	none

Lim et al. 2020a (Analysis 2, mobility as exposure)

**Study information**	
Author	Lim JT, Dickens BS, Choo EL, Chew LZ, Koo JR, Tam C, Park M, Cook AR
Title	Revealing Regional Disparities in the Transmission Potential of SARS-CoV-2 from Interventions in Southeast Asia
Year	2020
Journal	Proceedings of the Royal Society B: Biological Sciences
DOI	10.1098/rspb.2020.1173
Date received	20.05.2020
Date accepted	03.08.2020
**Study setting**	
Number of populations included	multiple
Level of populations included	national (9 South-east Asian countries)
Geographic areas covered	Asia

– continued on next page –

Number of countries covered	multiple countries
Study period	same end date for several populations with diverse epidemic trajectories
**Outcome**	
Raw outcome	epidemiological population-level outcome (cases)
Time resolution of raw outcome	daily
Computed outcome	none
Method to obtain the computed outcome	– not applicable –
Data source	data from publicly available cross-country selections (JHU)
Data availability	data access via source
**Intervention**	
Terminology for interventions	measures
Terminology for the specific type of non-pharmaceutical interventions	social distancing
Exposure types	mobility (time spent in various locations)
Types of single interventions	– not applicable –
Coding of interventions	– not applicable –
Source of intervention data	mobility data by corporate organizations (Google)
Availability of data on exposure	data access via source
**Methodological approach**	
Empirical approach	parametric
Use of exposure variation	only variation over time for multiple populations
Method	mechanistic model (semi-mechanistic Bayesian transmission model)
Code availability	none
**Effectiveness assessment**	

– continued on next page –

Reporting of intervention effectiveness	quantification of change in outcome values
Measure of effectiveness	reproduction number
Interpretation of results	implicitly causal
Reporting of uncertainty	yes
Sensitivity analysis	epidemiological parameters varied
Subgroup assessment	none

**Lim et al. 2020b**	
**Study information**	
Author	Lim JT, Dickens BS, Chew LZ, Choo EL, Koo JR, Aik J, Ng LC, Cook AR
Title	Impact of Sars-Cov-2 Interventions on Dengue Transmission
Year	2020
Journal	PLOS Neglected Tropical Diseases
DOI	10.1371/journal.pntd.0008719
Date received	2020-06-09
Date accepted	2020-08-16
**Study setting**	
Number of populations included	multiple
Level of populations included	national (Thailand, Malaysia, Singapore)
Geographic areas covered	Asia
Number of countries covered	multiple countries
Study period	end date could not be evaluated
**Outcome**	

– continued on next page –

Raw outcome	epidemiological population-level outcome (surrogate disease: cases dengue)
Time resolution of raw outcome	weekly, monthly
Computed outcome	none
Method to obtain the computed outcome	– not applicable –
Data source	data from (sub)national authorities (health authorities: Thailand and Singapore, Administrative Modernisation and Management Planning Unit: Malaysia), data from publicly available cross-country selections (WHO: all)
Data availability	data made available by the authors
**Intervention**	
Terminology for interventions	measures
Terminology for the specific type of non-pharmaceutical interventions	social distancing
Exposure types	all interventions together (social distancing policy implemented or not, timeline of measures provided)
Types of single interventions	– not applicable –
Coding of interventions	– not applicable –
Source of intervention data	– not applicable –
Availability of data on exposure	– not applicable –
**Methodological approach**	
Empirical approach	parametric
Use of exposure variation	only variation over time for multiple populations
Method	non-mechanistic model (generalized linear model)
Code availability	publicly available
**Effectiveness assessment**	
Reporting of intervention effectiveness	quantification of change in outcome values

– continued on next page –

Measure of effectiveness	cases: dengue
Interpretation of results	implicitly causal
Reporting of uncertainty	yes
Sensitivity analysis	model specification varied
Subgroup assessment	none

Linka et al. 2020 (Analysis 1)

**Study information**	
Author	Linka K, Peirlinck M, Kuhl E
Title	The Reproduction Number of COVID-19 and Its Correlation with Public Health Interventions
Year	2020
Journal	Computational Mechanics
DOI	10.1007/s00466-020-01880-8
Date received	2020-05-04
Date accepted	2020-07-06
**Study setting**	
Number of populations included	multiple
Level of populations included	national (all 27 EU countries)
Geographic areas covered	Europe
Number of countries covered	multiple countries
Study period	start and end date span first epidemic wave
**Outcome**	

– continued on next page –

Raw outcome	epidemiological population-level outcome (cases)
Time resolution of raw outcome	daily
Computed outcome	epidemiological parameter (reproduction number)
Method to obtain the computed outcome	compartmental transmission model (single-population)
Data source	data from publicly available cross-country selections (ECDC)
Data availability	data access via source
**Intervention**	
Terminology for interventions	interventions
Terminology for the specific type of non-pharmaceutical interventions	public health
Exposure types	all interventions together
Types of single interventions	– not applicable –
Coding of interventions	– not applicable –
Source of intervention data	– not applicable –
Availability of data on exposure	– not applicable –
**Methodological approach**	
Empirical approach	descriptive
Use of exposure variation	only variation over time for multiple populations
Method	description of change over time (comparison of time periods)
Code availability	none
**Effectiveness assessment**	
Reporting of intervention effectiveness	comparison of outcome values
Measure of effectiveness	reproduction number
Interpretation of results	implicitly causal

– continued on next page –

Reporting of uncertainty	yes
Sensitivity analysis	model specification varied
Subgroup assessment	none

**Linka et al. 2020 (Analysis 2, mobility as exposure)**	
**Study information**	
Author	Linka K, Peirlinck M, Kuhl E
Title	The Reproduction Number of COVID-19 and Its Correlation with Public Health Interventions
Year	2020
Journal	Computational Mechanics
DOI	10.1007/s00466-020-01880-8
Date received	04.05.2020
Date accepted	06.07.2020
**Study setting**	
Number of populations included	multiple
Level of populations included	national (all 27 EU countries)
Geographic areas covered	Europe
Number of countries covered	multiple countries
Study period	start and end date span first epidemic wave
**Outcome**	
Raw outcome	epidemiological population-level outcome (cases)
Time resolution of raw outcome	daily
Computed outcome	epidemiological parameter (reproduction number)

– continued on next page –

Method to obtain the computed outcome	compartmental transmission model (single-population)
Data source	data from publicly available cross-country selections (ECDC)
Data availability	data access via source
**Intervention**	
Terminology for interventions	interventions
Terminology for the specific type of non-pharmaceutical interventions	public health
Exposure types	mobility (air travel data, number of requests for directions in Maps)
Types of single interventions	– not applicable –
Coding of interventions	– not applicable –
Source of intervention data	mobility data by corporate organizations (Eurocontrol: air travel data, Apple mobility: number of trips by mode)
Availability of data on exposure	data access via source
**Methodological approach**	
Empirical approach	descriptive
Use of exposure variation	only variation over time for multiple populations
Method	non-mechanistic model (correlation)
Code availability	none
**Effectiveness assessment**	
Reporting of intervention effectiveness	quantification of change in outcome values
Measure of effectiveness	reproduction number
Interpretation of results	implicitly causal
Reporting of uncertainty	no

– continued on next page –

Sensitivity analysis	none
Subgroup assessment	none

**Liu et al. 2020a**	
**Study information**	
Author	Liu PY, He S, Rong LB, Tang SY
Title	The Effect of Control Measures on COVID-19 Transmission in Italy: Comparison with Guangdong Province in China
Year	2020
Journal	Infectious Diseases of Poverty
DOI	10.1186/s40249-020-00730-2
Date received	2020-04-01
Date accepted	2020-07-22
**Study setting**	
Number of populations included	single
Level of populations included	national (Italy)
Geographic areas covered	Europe
Number of countries covered	single country (Italy)
Study period	end date in growth phase of wave
**Outcome**	
Raw outcome	epidemiological population-level outcome (cases, deaths, recovered cases)
Time resolution of raw outcome	daily
Computed outcome	summary statistic (initial contact rate, prevalence ratio: cases, cure ratio: recovered, time until 1000 cases)

– continued on next page –

Method to obtain the computed outcome	simple computation (ratios), compartmental transmission model (compartmental single-population transmission model: initial contact rate)
Data source	data from (sub)national authorities (Italy and Guangdong)
Data availability	data access via source
**Intervention**	
Terminology for interventions	measures
Terminology for the specific type of non-pharmaceutical interventions	control
Exposure types	all interventions together (timeline of measures)
Types of single interventions	– not applicable –
Coding of interventions	– not applicable –
Source of intervention data	– not applicable –
Availability of data on exposure	– not applicable –
**Methodological approach**	
Empirical approach	descriptive
Use of exposure variation	only variation over time for a single population
Method	comparison of populations
Code availability	none
**Effectiveness assessment**	
Reporting of intervention effectiveness	comparison of outcome values
Measure of effectiveness	contact rate: initial contact rate, cases, recovered, time until 1000 cases
Interpretation of results	implicitly causal
Reporting of uncertainty	no
Sensitivity analysis	none
Subgroup assessment	none

**Liu et al. 2020b (Analysis 1)**	
**Study information**	
Author	Liu K, Ai S, Song S, Zhu G, Tian F, Li H, Gao Y, Wu Y, Zhang S, Shao Z, Liu Q, Lin H
Title	Population Movement, City Closure in Wuhan, and Geographical Expansion of the COVID-19 Infection in China in January 2020
Year	2020
Journal	Clinical Infectious Diseases
DOI	10.1093/cid/ciaa422
Date received	2020-02-11
Date accepted	2020-04-17
**Study setting**	
Number of populations included	multiple
Level of populations included	both national and subnational (Chinese provinces)
Geographic areas covered	Asia
Number of countries covered	single country (China)
Study period	start and end date span first epidemic wave: behavioral
**Outcome**	
Raw outcome	behavioral outcome (mobility: population outflow from Wuhan)
Time resolution of raw outcome	daily
Computed outcome	none
Method to obtain the computed outcome	– not applicable –
Data source	mobility data from corporate organizations (Baidu)

– continued on next page –

Data availability	data access via source
**Intervention**	
Terminology for interventions	measures
Terminology for the specific type of non-pharmaceutical interventions	prevention, control
Exposure types	one combination of interventions (Wuhan City Lockdown)
Types of single interventions	– not applicable –
Coding of interventions	not necessary
Source of intervention data	– could not be evaluated –
Availability of data on exposure	raw data documented in the manuscript
**Methodological approach**	
Empirical approach	descriptive
Use of exposure variation	only variation over time for multiple populations
Method	description of change over time (comparison of time periods)
Code availability	none
**Effectiveness assessment**	
Reporting of intervention effectiveness	comparison of outcome values
Measure of effectiveness	mobility: population outflow from Wuhan
Interpretation of results	implicitly causal
Reporting of uncertainty	no
Sensitivity analysis	none
Subgroup assessment	none

Liu et al. 2020b (Analysis 2, mobility as exposure)	
**Study information**	
Author	Liu K, Ai S, Song S, Zhu G, Tian F, Li H, Gao Y, Wu Y, Zhang S, Shao Z, Liu Q, Lin H
Title	Population Movement, City Closure in Wuhan, and Geographical Expansion of the COVID-19 Infection in China in January 2020
Year	2020
Journal	Clinical Infectious Diseases
DOI	10.1093/cid/ciaa422
Date received	11.02.2020
Date accepted	17.04.2020
**Study setting**	
Number of populations included	multiple
Level of populations included	both national and subnational (Chinese provinces)
Geographic areas covered	Asia
Number of countries covered	single country (China)
Study period	end date in growth phase of wave
**Outcome**	
Raw outcome	epidemiological population-level outcome (cases)
Time resolution of raw outcome	daily
Computed outcome	none
Method to obtain the computed outcome	– not applicable –
Data source	data from (sub)national authorities
Data availability	data access via source
**Intervention**	
Terminology for interventions	measures

– continued on next page –

Terminology for the specific type of non-pharmaceutical interventions	prevention, control
Exposure types	mobility (population outflow from Wuhan)
Types of single interventions	– not applicable –
Coding of interventions	– not applicable –
Source of intervention data	mobility data by corporate organizations (Baidu)
Availability of data on exposure	data access via source
**Methodological approach**	
Empirical approach	counterfactual
Use of exposure variation	only variation between populations
Method	other
Code availability	none
**Effectiveness assessment**	
Reporting of intervention effectiveness	quantification of change in outcome values
Measure of effectiveness	cases
Interpretation of results	implicitly causal
Reporting of uncertainty	yes
Sensitivity analysis	none
Subgroup assessment	none

Loeffler-Wirth et al. 2020

**Study information**	
Author	Loeffler-Wirth H, Schmidt M, Binder H

– continued on next page –

Title	Covid-19 Transmission Trajectories–Monitoring the Pandemic in the World-wide Context
Year	2020
Journal	Viruses
DOI	10.3390/v12070777
Date received	2020-06-30
Date accepted	2020-07-14
**Study setting**	
Number of populations included	multiple
Level of populations included	both national and subnational (187 countries, 17 German states)
Geographic areas covered	Europe, Middle East and Africa, Asia, Oceania, Central and South America, North America
Number of countries covered	multiple countries
Study period	same end date for several populations with diverse epidemic trajectories
**Outcome**	
Raw outcome	epidemiological population-level outcome (cases)
Time resolution of raw outcome	daily
Computed outcome	epidemiological parameter (reproduction number)
Method to obtain the computed outcome	compartmental transmission model (single-population)
Data source	data from publicly available cross-country selections (JHU: all other countries), data from (sub)national authorities (Germany)
Data availability	data access via source
**Intervention**	
Terminology for interventions	interventions

– continued on next page –

Terminology for the specific type of non-pharmaceutical interventions	non-pharmaceutical
Exposure types	all interventions together
Types of single interventions	– not applicable –
Coding of interventions	– not applicable –
Source of intervention data	– not applicable –
Availability of data on exposure	– not applicable –
**Methodological approach**	
Empirical approach	descriptive
Use of exposure variation	only variation over time for multiple populations
Method	description of change over time (description of time course)
Code availability	none
**Effectiveness assessment**	
Reporting of intervention effectiveness	qualitative statement
Measure of effectiveness	cases, reproduction number
Interpretation of results	implicitly causal
Reporting of uncertainty	– not applicable –
Sensitivity analysis	none
Subgroup assessment	none

Lurie et al. 2020 (Analysis 1)

Study information

– continued on next page –

Author	Lurie MN, Silva J, Yorlets RR, Tao J, Chan PA
Title	Coronavirus Disease 2019 Epidemic Doubling Time in the United States before and during Stay-at-Home Restrictions
Year	2020
Journal	The Journal of Infectious Diseases
DOI	10.1093/infdis/jiaa491
Date received	2020-06-15
Date accepted	2020-07-30
**Study setting**	
Number of populations included	multiple
Level of populations included	both national and subnational (US and its states)
Geographic areas covered	North America
Number of countries covered	single country (US)
Study period	same end date for several populations with diverse epidemic trajectories
**Outcome**	
Raw outcome	epidemiological population-level outcome (cases)
Time resolution of raw outcome	daily
Computed outcome	measure of epidemic trend (doubling time)
Method to obtain the computed outcome	simple computation
Data source	data from publicly available cross-country selections (JHU)
Data availability	data access via source
**Intervention**	
Terminology for interventions	strategies
Terminology for the specific type of non-pharmaceutical interventions	mitigation

– continued on next page –

Exposure types	one single intervention (stay-at-home order)
Types of single interventions	stay-at-home order
Coding of interventions	necessary
Source of intervention data	use of externally coded data (New York Times, OxCGRT (for national level))
Availability of data on exposure	access to externally coded data via source
**Methodological approach**	
Empirical approach	descriptive
Use of exposure variation	only variation over time for multiple populations
Method	description of change over time (comparison of time periods)
Code availability	none
**Effectiveness assessment**	
Reporting of intervention effectiveness	quantification of change in outcome values
Measure of effectiveness	doubling time
Interpretation of results	associative
Reporting of uncertainty	yes
Sensitivity analysis	none
Subgroup assessment	none

Lurie et al. 2020 (Analysis 2)

**Study information**	
Author	Lurie MN, Silva J, Yorlets RR, Tao J, Chan PA
Title	Coronavirus Disease 2019 Epidemic Doubling Time in the United States before and during Stay-at-Home Restrictions

– continued on next page –

Year	2020
Journal	The Journal of Infectious Diseases
DOI	10.1093/infdis/jiaa491
Date received	2020-06-15
Date accepted	2020-07-30
**Study setting**	
Number of populations included	single
Level of populations included	national (US)
Geographic areas covered	North America
Number of countries covered	single country (US)
Study period	start and end date span first epidemic wave
**Outcome**	
Raw outcome	epidemiological population-level outcome (cases)
Time resolution of raw outcome	daily
Computed outcome	measure of epidemic trend (doubling time)
Method to obtain the computed outcome	simple computation
Data source	data from publicly available cross-country selections (JHU)
Data availability	data access via source
**Intervention**	
Terminology for interventions	strategies
Terminology for the specific type of non-pharmaceutical interventions	mitigation
Exposure types	stringency index (OxCGRT Stringency Index)
Types of single interventions	– not applicable –
Coding of interventions	necessary

– continued on next page –

Source of intervention data	use of externally coded data (OxCGRT)
Availability of data on exposure	access to externally coded data via source
**Methodological approach**	
Empirical approach	descriptive
Use of exposure variation	only variation over time for a single population
Method	description of change over time (comparison of time periods)
Code availability	none
**Effectiveness assessment**	
Reporting of intervention effectiveness	quantification of change in outcome values
Measure of effectiveness	doubling time
Interpretation of results	associative
Reporting of uncertainty	yes
Sensitivity analysis	none
Subgroup assessment	none

Maier & Brockmann 2020

**Study information**	
Author	Maier BF, Brockmann D
Title	Effective Containment Explains Subexponential Growth in Recent Confirmed COVID-19 Cases in China
Year	2020
Journal	Science
DOI	10.1126/science.abb4557

– continued on next page –

Date received	2020-02-25
Date accepted	2020-04-04
**Study setting**	
Number of populations included	multiple
Level of populations included	both national and subnational (Hubei + 28 other provinces (China))
Geographic areas covered	Asia
Number of countries covered	single country (China)
Study period	end date at peak of wave
**Outcome**	
Raw outcome	epidemiological population-level outcome (cases)
Time resolution of raw outcome	daily
Computed outcome	none
Method to obtain the computed outcome	– not applicable –
Data source	data from publicly available cross-country selections (JHU)
Data availability	data made available by the authors
**Intervention**	
Terminology for interventions	measures
Terminology for the specific type of non-pharmaceutical interventions	containment
Exposure types	categories of interventions (containment and quarantining)
Types of single interventions	– not applicable –
Coding of interventions	– not applicable –
Source of intervention data	– not applicable –
Availability of data on exposure	– not applicable –
**Methodological approach**	

– continued on next page –

Empirical approach	parametric
Use of exposure variation	only variation over time for multiple populations
Method	mechanistic model (compartmental single-population transmission model)
Latent variable functionally linked to intervention	susceptible decay
Code availability	publicly available
**Effectiveness assessment**	
Reporting of intervention effectiveness	quantification of change in outcome values
Measure of effectiveness	reproduction number
Interpretation of results	implicitly causal
Reporting of uncertainty	no
Sensitivity analysis	epidemiological parameters varied
Subgroup assessment	none

**Malheiro et al. 2020**	
**Study information**	
Author	Malheiro R, Figueiredo AL, Magalhães JP, Teixeira P, Moita I, Moutinho MC, Mansilha RB, Gonçalves LM, Ferreira E
Title	Effectiveness of Contact Tracing and Quarantine on Reducing COVID-19 Transmission: A Retrospective Cohort Study
Year	2020
Journal	Public Health
DOI	10.1016/j.puhe.2020.09.012
Date received	2020-07-03

– continued on next page –

Date accepted	2020-09-16
**Study setting**	
Number of populations included	single
Level of populations included	subnational (Eastern Porto (Portugal))
Geographic areas covered	Europe
Number of countries covered	single country (Portugal)
Study period	start and end date span first epidemic wave
**Outcome**	
Raw outcome	epidemiological individual-level outcome (individual cases and transmission chains)
Time resolution of raw outcome	daily
Computed outcome	epidemiological parameter (secondary cases per index case, proportion of index cases with secondary cases, time between symptom onset and testing, number of close contacts)
Method to obtain the computed outcome	simple computation
Data source	data from (sub)national authorities (National Epidemiological Surveillance System and protected databases used for the daily monitoring and registration of close contacts for follow-up)
Data availability	data not accessible
**Intervention**	
Terminology for interventions	measures
Terminology for the specific type of non-pharmaceutical interventions	lockdown
Exposure types	one combination of interventions (state of emergency as decreed by the Portugese government on March 18 (lockdown measures, including constraints in public circulation, closure of commercial activities, and resort to homeworking))

– continued on next page –

Types of single interventions	– not applicable –
Coding of interventions	not necessary
Source of intervention data	government or news websites
Availability of data on exposure	raw data documented in the manuscript
**Methodological approach**	
Empirical approach	descriptive
Use of exposure variation	only variation over time for a single population
Method	description of change over time (comparison of time periods)
Code availability	none
**Effectiveness assessment**	
Reporting of intervention effectiveness	quantification of change in outcome values
Measure of effectiveness	secondary cases per index case, proportion of index cases with secondary cases, time between symptom onset and testing, number of contacts
Interpretation of results	implicitly causal
Reporting of uncertainty	yes
Sensitivity analysis	none
Subgroup assessment	public health response (subject to other measures: contact tracing and quarantine measures)

Manevski et al. 2020

**Study information**	
Author	Manevski D, Gorenjec NR, Kejžar N, Blagus R
Title	Modeling COVID-19 Pandemic Using Bayesian Analysis with Application to Slovene Data

– continued on next page –

Year	2020
Journal	Mathematical Biosciences
DOI	10.1016/j.mbs.2020.108466
Date received	2020-07-08
Date accepted	2020-09-05
**Study setting**	
Number of populations included	single
Level of populations included	national (Slovenia)
Geographic areas covered	Europe
Number of countries covered	single country (Slovenia)
Study period	start and end date span first epidemic wave
**Outcome**	
Raw outcome	epidemiological population-level outcome (cases, deaths, hospitalizations, ICU hospitalizations)
Time resolution of raw outcome	daily
Computed outcome	none
Method to obtain the computed outcome	– not applicable –
Data source	data from publicly available open-source projects (COVID-19 Tracker Slovenia)
Data availability	data made available by the authors
**Intervention**	
Terminology for interventions	interventions
Terminology for the specific type of non-pharmaceutical interventions	non-pharmaceutical

– continued on next page –

Exposure types	all interventions together (event bans, lockdown, prohibition of movement outside municipality of residence)
Types of single interventions	– not applicable –
Coding of interventions	– not applicable –
Source of intervention data	– not applicable –
Availability of data on exposure	– not applicable –
**Methodological approach**	
Empirical approach	parametric
Use of exposure variation	only variation over time for a single population
Method	mechanistic model (semi-mechanistic Bayesian transmission model)
Latent variable functionally linked to intervention	reproduction number
Code availability	publicly available
**Effectiveness assessment**	
Reporting of intervention effectiveness	comparison of outcome values
Measure of effectiveness	reproduction number
Interpretation of results	associative
Reporting of uncertainty	yes
Sensitivity analysis	different or modified outcome used (only deaths used), model specification varied, epidemiological parameters varied
Subgroup assessment	none

Marschner 2020	
**Study information**	
Author	Marschner IC
Title	Back-Projection of COVID-19 Diagnosis Counts to Assess Infection Incidence and Control Measures: Analysis of Australian Data
Year	2020
Journal	Epidemiology and Infection
DOI	10.1017/s0950268820001065
Date received	2020-04-26
Date accepted	2020-05-11
**Study setting**	
Number of populations included	single
Level of populations included	national (Australia)
Geographic areas covered	Oceania
Number of countries covered	single country (Australia)
Study period	start and end date span first epidemic wave
**Outcome**	
Raw outcome	epidemiological population-level outcome (cases)
Time resolution of raw outcome	daily
Computed outcome	other (infections)
Method to obtain the computed outcome	back projection
Data source	data from (sub)national authorities
Data availability	data made available by the authors
**Intervention**	
Terminology for interventions	measures

– continued on next page –

Terminology for the specific type of non-pharmaceutical interventions	control
Exposure types	phases of interventions (border closure and three stages of social distancing measures)
Types of single interventions	– not applicable –
Coding of interventions	not necessary
Source of intervention data	government or news websites
Availability of data on exposure	raw data documented in the manuscript
**Methodological approach**	
Empirical approach	descriptive
Use of exposure variation	only variation over time for a single population
Method	description of change over time (description of time course)
Code availability	publicly available
**Effectiveness assessment**	
Reporting of intervention effectiveness	qualitative statement
Measure of effectiveness	infections
Interpretation of results	associative
Reporting of uncertainty	– not applicable –
Sensitivity analysis	different or modified outcome used (undetected cases adjusted for), epidemiological parameters varied
Subgroup assessment	none

McCarthy et al. 2020	
**Study information**	
Author	McCarthy Z, Xiao Y, Scarabel F, Tang B, Bragazzi NL, Nah K, Heffernan JM, Asgary A, Murty VK, Ogden NH, Wu J
Title	Quantifying the Shift in Social Contact Patterns in Response to Non-Pharmaceutical Interventions
Year	2020
Journal	Journal of Mathematics in Industry
DOI	10.1186/s13362-020-00096-y
Date received	2020-10-15
Date accepted	2020-11-25
**Study setting**	
Number of populations included	single
Level of populations included	subnational (Ontario (Canada))
Geographic areas covered	North America
Number of countries covered	single country (Canada)
Study period	start and end date span first epidemic wave
**Outcome**	
Raw outcome	epidemiological population-level outcome (cases)
Time resolution of raw outcome	daily
Computed outcome	none
Method to obtain the computed outcome	– not applicable –
Data source	data from other research projects (publicly funded research project: contact matrices), data from (sub)national authorities (cases)
Data availability	data access via source
**Intervention**	

– continued on next page –

Terminology for interventions	measures
Terminology for the specific type of non-pharmaceutical interventions	physical distancing
Exposure types	multiple combinations of interventions (public school closure (from March 14), declaration of state of emergency, with closure of public venues and events and physical distancing advisories (from March 18), and closure of non-essential establishments (from March 24))
Types of single interventions	– not applicable –
Coding of interventions	not necessary
Source of intervention data	government or news websites
Availability of data on exposure	raw data documented in the manuscript
**Methodological approach**	
Empirical approach	parametric
Use of exposure variation	only variation over time for a single population
Method	mechanistic model (compartmental single-population transmission model)
Latent variable functionally linked to intervention	contact matrix
Code availability	none
**Effectiveness assessment**	
Reporting of intervention effectiveness	quantification of change in outcome values
Measure of effectiveness	reproduction number, contact rate, contact matrix
Interpretation of results	associative
Reporting of uncertainty	no
Sensitivity analysis	epidemiological parameters varied
Subgroup assessment	socioeconomic indicators (age), epidemiological indicators (infection setting)

McGrail et al. 2020	
**Study information**	
Author	McGrail DJ, Dai J, McAndrews KM, Kalluri R
Title	Enacting National Social Distancing Policies Corresponds with Dramatic Reduction in COVID19 Infection Rates
Year	2020
Journal	PLOS ONE
DOI	10.1371/journal.pone.0236619
Date received	2020-05-01
Date accepted	2020-07-10
**Study setting**	
Number of populations included	multiple
Level of populations included	both national and subnational (50 US states, 120 countries)
Geographic areas covered	Europe, Middle East and Africa, Asia, Oceania, Central and South America, North America
Number of countries covered	multiple countries
Study period	same end date for several populations with diverse epidemic trajectories
**Outcome**	
Raw outcome	epidemiological population-level outcome (cases), behavioral outcome (mobility: average reduction in time spent in various locations)
Time resolution of raw outcome	daily
Computed outcome	measure of epidemic trend (growth rate)
Method to obtain the computed outcome	exponential growth model
Data source	data from publicly available cross-country selections (JHU)
Data availability	data access via source

– continued on next page –

**Intervention**	
Terminology for interventions	measures
Terminology for the specific type of non-pharmaceutical interventions	social distancing
Exposure types	one single intervention (workplace closure)
Types of single interventions	workplace closure
Coding of interventions	necessary
Source of intervention data	use of externally coded data (Aura Vision Global Covid-19 Lockdown Tracker)
Availability of data on exposure	coded data made available by the authors
**Methodological approach**	
Empirical approach	descriptive
Use of exposure variation	both variation over time and between populations
Method	description of change over time (comparison of time periods)
Code availability	none
**Effectiveness assessment**	
Reporting of intervention effectiveness	quantification of change in outcome values
Measure of effectiveness	growth rate, mobility: average reduction in time spent in various locations
Interpretation of results	implicitly causal
Reporting of uncertainty	yes
Sensitivity analysis	start or end date of study period varied
Subgroup assessment	none

McKee et al. 2020	
**Study information**	
Author	McKee KL, Crandell IC, Hanlon AL
Title	County-Level Social Distancing and Policy Impact in the United States: A Dynamical Systems Model
Year	2020
Journal	JMIR Public Health and Surveillance
DOI	10.2196/23902
Date received	2020-09-10
Date accepted	2020-11-27
**Study setting**	
Number of populations included	multiple
Level of populations included	both national and subnational (3050 counties in the US)
Geographic areas covered	North America
Number of countries covered	single country (US)
Study period	start and end date span first epidemic wave: behavioral
**Outcome**	
Raw outcome	behavioral outcome (mobility: distance traveled & visitation to key sites & log number of interpersonal encounters)
Time resolution of raw outcome	daily
Computed outcome	none
Method to obtain the computed outcome	– not applicable –
Data source	mobility data from corporate organizations (Unacast)
Data availability	data access via source
**Intervention**	
Terminology for interventions	measures

– continued on next page –

Terminology for the specific type of non-pharmaceutical interventions	social distancing
Exposure types	multiple separate interventions (stay-at-home order, bans of small gatherings, school closure, workplace closure)
Types of single interventions	stay-at-home order, bans of small gatherings, school closure, workplace closure
Coding of interventions	necessary
Source of intervention data	use of externally coded data (Kaiser Fung Family)
Availability of data on exposure	access to externally coded data via source
**Methodological approach**	
Empirical approach	parametric
Use of exposure variation	both variation over time and between populations
Method	other (differential equation-based state-space model to estimate effects of interventions on mobility)
Code availability	none
**Effectiveness assessment**	
Reporting of intervention effectiveness	quantification of change in outcome values
Measure of effectiveness	mobility: distance traveled & visitation to key sites & log number of interpersonal encounters
Interpretation of results	associative
Reporting of uncertainty	yes
Sensitivity analysis	none
Subgroup assessment	none

McKenzie & Adams 2020	
**Study information**	
Author	McKenzie G, Adams B
Title	A Country Comparison of Place-Based Activity Response to COVID-19 Policies
Year	2020
Journal	Applied Geography
DOI	10.1016/j.apgeog.2020.102363
Date received	2020-05-18
Date accepted	2020-10-26
**Study setting**	
Number of populations included	multiple
Level of populations included	both national and subnational (108 countries around the globe, 46 with subnational regions)
Geographic areas covered	Europe, Middle East and Africa, Asia, Oceania, Central and South America, North America
Number of countries covered	multiple countries
Study period	start and end date span first epidemic wave: behavioral
**Outcome**	
Raw outcome	behavioral outcome (mobility: time spent in various locations)
Time resolution of raw outcome	daily
Computed outcome	none
Method to obtain the computed outcome	– not applicable –
Data source	mobility data from corporate organizations (Google)
Data availability	data made available by the authors
**Intervention**	

– continued on next page –

Terminology for interventions	responses
Terminology for the specific type of non-pharmaceutical interventions	government
Exposure types	stringency index (OxCGRT Stringency Index)
Types of single interventions	– not applicable –
Coding of interventions	necessary
Source of intervention data	use of externally coded data (OxCGRT)
Availability of data on exposure	coded data made available by the authors
**Methodological approach**	
Empirical approach	parametric
Use of exposure variation	only variation over time for multiple populations
Method	non-mechanistic model (correlation)
Code availability	publicly available
**Effectiveness assessment**	
Reporting of intervention effectiveness	quantification of change in outcome values
Measure of effectiveness	mobility: time spent in various locations
Interpretation of results	associative
Reporting of uncertainty	yes
Sensitivity analysis	none
Subgroup assessment	geographic areas (regions)

Medline et al. 2020

Study information

– continued on next page –

Author	Medline A, Hayes L, Valdez K, Hayashi A, Vahedi F, Capell W, Sonnenberg J, Glick Z, Klausner JD
Title	Evaluating the Impact of Stay-at-Home Orders on the Time to Reach the Peak Burden of COVID-19 Cases and Deaths: Does Timing Matter?
Year	2020
Journal	BMC Public Health
DOI	10.1186/s12889-020-09817-9
Date received	2020-06-20
Date accepted	2020-11-02
**Study setting**	
Number of populations included	multiple
Level of populations included	both national and subnational (41 countries, US and its states)
Geographic areas covered	Europe, Middle East and Africa, Asia, Oceania, Central and South America, North America
Number of countries covered	multiple countries
Study period	end date could not be evaluated
**Outcome**	
Raw outcome	epidemiological population-level outcome (cases, deaths)
Time resolution of raw outcome	daily
Computed outcome	summary statistic (time from first case to peak of cases/deaths)
Method to obtain the computed outcome	simple computation
Data source	data from publicly available cross-country selections (WHO: all countries, Worldometer: all countries), data from (sub)national authorities (US)
Data availability	data access via source
**Intervention**	

– continued on next page –

Terminology for interventions	interventions
Terminology for the specific type of non-pharmaceutical interventions	non-pharmaceutical
Exposure types	one single intervention (stay-at-home order)
Types of single interventions	stay-at-home order
Coding of interventions	necessary
Source of intervention data	government or news websites (countries), New York Times (US)
Availability of data on exposure	access to externally coded data via source
**Methodological approach**	
Empirical approach	parametric
Use of exposure variation	only variation between populations
Method	non-mechanistic model (generalized linear model)
Code availability	none
**Effectiveness assessment**	
Reporting of intervention effectiveness	quantification of change in outcome values
Measure of effectiveness	time to peak: cases/deaths
Interpretation of results	associative
Reporting of uncertainty	yes
Sensitivity analysis	none
Subgroup assessment	none

Miller et al. 2020

**Study information**	

– continued on next page –

Author	Miller D, Martin MA, Harel N, Tirosh O, Kustin T, Meir M, Sorek N, Gefen-Halevi S, Amit S, Vorontsov O, Shaag A, Wolf D, Peretz A, Shemer-Avni Y, Roif-Kaminsky D, Kopelman NM, Huppert A, Koelle K, Stern A
Title	Full Genome Viral Sequences Inform Patterns of SARS-CoV-2 Spread into and within Israel
Year	2020
Journal	Nature Communications
DOI	10.1038/s41467-020-19248-0
Date received	2020-06-12
Date accepted	2020-10-02
**Study setting**	
Number of populations included	single
Level of populations included	national (Israel)
Geographic areas covered	Asia
Number of countries covered	single country (Israel)
Study period	start and end date span first epidemic wave
**Outcome**	
Raw outcome	epidemiological individual-level outcome (genome sequence data)
Time resolution of raw outcome	daily
Computed outcome	none
Method to obtain the computed outcome	– not applicable –
Data source	data collected by authors, data from publicly available cross-country selections (GISAID)
Data availability	data made available by the authors
**Intervention**	

– continued on next page –

Terminology for interventions	measures
Terminology for the specific type of non-pharmaceutical interventions	social distancing
Exposure types	one combination of interventions (interventions implemented on March 19)
Types of single interventions	– not applicable –
Coding of interventions	not necessary
Source of intervention data	– could not be evaluated –
Availability of data on exposure	raw data documented in the manuscript
**Methodological approach**	
Empirical approach	parametric
Use of exposure variation	only variation over time for a single population
Method	mechanistic model (phylodynamic model)
Latent variable functionally linked to intervention	reproduction number
Code availability	publicly available
**Effectiveness assessment**	
Reporting of intervention effectiveness	quantification of change in outcome values
Measure of effectiveness	reproduction number
Interpretation of results	associative
Reporting of uncertainty	yes
Sensitivity analysis	model specification varied, epidemiological parameters varied
Subgroup assessment	none

**Mitra et al. 2020**	
**Study information**	
Author	Mitra A, Pakhare AP, Roy A, Joshi A
Title	Impact of COVID-19 Epidemic Curtailment Strategies in Selected Indian States: An Analysis by Reproduction Number and Doubling Time with Incidence Modelling
Year	2020
Journal	PLOS ONE
DOI	10.1371/journal.pone.0239026
Date received	2020-05-18
Date accepted	2020-08-28
**Study setting**	
Number of populations included	multiple
Level of populations included	subnational (Top 10 Indian states with highest incidence)
Geographic areas covered	Asia
Number of countries covered	single country (India)
Study period	end date in growth phase of wave
**Outcome**	
Raw outcome	epidemiological population-level outcome (cases)
Time resolution of raw outcome	daily
Computed outcome	measure of epidemic trend (growth rate, doubling time), epidemiological parameter (reproduction number)
Method to obtain the computed outcome	exponential growth model (growth rate), exponential growth model (doubling time), statistical estimation of reproduction number (White et al.)
Data source	data from publicly available open-source projects (covid19India.org)
Data availability	data made available by the authors
**Intervention**	

– continued on next page –

Terminology for interventions	strategies
Terminology for the specific type of non-pharmaceutical interventions	curtailment
Exposure types	one combination of interventions (Indian National Lockdown)
Types of single interventions	– not applicable –
Coding of interventions	not necessary
Source of intervention data	– could not be evaluated –
Availability of data on exposure	raw data documented in the manuscript
**Methodological approach**	
Empirical approach	descriptive
Use of exposure variation	only variation over time for multiple populations
Method	description of change over time (comparison of time periods)
Code availability	publicly available
**Effectiveness assessment**	
Reporting of intervention effectiveness	comparison of outcome values
Measure of effectiveness	growth rate, doubling time, reproduction number
Interpretation of results	associative
Reporting of uncertainty	yes
Sensitivity analysis	none
Subgroup assessment	none

Moon et al. 2020

Study information

– continued on next page –

Author	Moon SG, Kim YK, Son WS, Kim JH, Choi J, Na BJ, Park B
Title	Time Variant Reproductive Number of COVID-19 in Seoul, Korea
Year	2020
Journal	Epidemiology and Health
DOI	10.4178/epih.e2020047
Date received	2020-04-01
Date accepted	2020-06-20
**Study setting**	
Number of populations included	single
Level of populations included	subnational (Seoul (Korea))
Geographic areas covered	Asia
Number of countries covered	single country (South Korea)
Study period	start and end date span first epidemic wave
**Outcome**	
Raw outcome	epidemiological population-level outcome (cases)
Time resolution of raw outcome	daily
Computed outcome	epidemiological parameter (reproduction number)
Method to obtain the computed outcome	statistical estimation of reproduction number (Cori et al.)
Data source	data from (sub)national authorities
Data availability	data access via source
**Intervention**	
Terminology for interventions	policies
Terminology for the specific type of non-pharmaceutical interventions	quarantine
Exposure types	all interventions together

– continued on next page –

Types of single interventions	– not applicable –
Coding of interventions	– not applicable –
Source of intervention data	– not applicable –
Availability of data on exposure	– not applicable –
**Methodological approach**	
Empirical approach	descriptive
Use of exposure variation	only variation over time for a single population
Method	description of change over time (description of time course)
Code availability	none
**Effectiveness assessment**	
Reporting of intervention effectiveness	qualitative statement
Measure of effectiveness	reproduction number
Interpretation of results	associative
Reporting of uncertainty	– not applicable –
Sensitivity analysis	different or modified outcome used (use effective reproduction number based on cases by date of confirmation and by date of onset), epidemiological parameters varied
Subgroup assessment	none

Moreno et al. 2020

Study information

– continued on next page –

Author	Moreno GK, Braun KM, Riemersma KK, Martin MA, Halfmann PJ, Crooks CM, Prall T, Baker D, Baczenas JJ, Heffron AS, Ramuta M, Khubbar M, Weiler AM, Accola MA, Rehrauer WM, O'Connor SL, Safdar N, Pepperell CS, Dasu T, Bhattacharyya S, Kawaoka Y, Koelle K, O'Connor DH, Friedrich TC
Title	Revealing Fine-Scale Spatiotemporal Differences in SARS-CoV-2 Introduction and Spread
Year	2020
Journal	Nature Communications
DOI	10.1038/s41467-020-19346-z
Date received	2020-06-30
Date accepted	2020-10-06
**Study setting**	
Number of populations included	multiple
Level of populations included	subnational (2 US counties (Dane and Milwaukee))
Geographic areas covered	North America
Number of countries covered	single country (US)
Study period	end date in growth phase of wave
**Outcome**	
Raw outcome	epidemiological individual-level outcome (genome sequence data)
Time resolution of raw outcome	daily
Computed outcome	none
Method to obtain the computed outcome	– not applicable –
Data source	data collected by authors (hospitals and laboratories in the counties)
Data availability	data made available by the authors
**Intervention**	

– continued on next page –

Terminology for interventions	interventions
Terminology for the specific type of non-pharmaceutical interventions	public health
Exposure types	one single intervention (stay-at-home order (business closure, stay-at-home order, gathering bans))
Types of single interventions	stay-at-home order
Coding of interventions	not necessary
Source of intervention data	government or news websites
Availability of data on exposure	raw data documented in the manuscript
**Methodological approach**	
Empirical approach	parametric
Use of exposure variation	only variation over time for multiple populations
Method	mechanistic model (phylodynamic model)
Latent variable functionally linked to intervention	reproduction number
Code availability	publicly available
**Effectiveness assessment**	
Reporting of intervention effectiveness	quantification of change in outcome values
Measure of effectiveness	reproduction number
Interpretation of results	associative
Reporting of uncertainty	yes
Sensitivity analysis	epidemiological parameters varied
Subgroup assessment	none

Moshammer et al. 2020	
**Study information**	
Author	Moshammer H, Poteser M, Lemmerer K, Wallner P, Hutter HP
Title	Time Course of COVID-19 Cases in Austria
Year	2020
Journal	International Journal of Environmental Research and Public Health
DOI	10.3390/ijerph17093270
Date received	2020-04-15
Date accepted	2020-05-06
**Study setting**	
Number of populations included	single
Level of populations included	national (Austria)
Geographic areas covered	Europe
Number of countries covered	single country (Austria)
Study period	start and end date span first epidemic wave
**Outcome**	
Raw outcome	epidemiological population-level outcome (cases)
Time resolution of raw outcome	daily
Computed outcome	epidemiological parameter (reproduction number)
Method to obtain the computed outcome	back projection, statistical estimation of reproduction number (other, very simplistic approach)
Data source	data from publicly available cross-country selections (JHU)
Data availability	data access via source
**Intervention**	
Terminology for interventions	measures
Terminology for the specific type of non-pharmaceutical interventions	public health

– continued on next page –

Exposure types	all interventions together (closure of schools, restraurants, shops, cancellation of conferences etc)
Types of single interventions	– not applicable –
Coding of interventions	– not applicable –
Source of intervention data	– not applicable –
Availability of data on exposure	– not applicable –
**Methodological approach**	
Empirical approach	descriptive
Use of exposure variation	only variation over time for a single population
Method	description of change over time (description of time course)
Code availability	none
**Effectiveness assessment**	
Reporting of intervention effectiveness	qualitative statement
Measure of effectiveness	cases, reproduction number
Interpretation of results	implicitly causal
Reporting of uncertainty	– not applicable –
Sensitivity analysis	none
Subgroup assessment	none

Mukandavire et al. 2020

**Study information**	
Author	Mukandavire Z, Nyabadza F, Malunguza NJ, Cuadros DF, Shiri T, Musuka G

– continued on next page –

Title	Quantifying Early COVID-19 Outbreak Transmission in South Africa and Exploring Vaccine Efficacy Scenarios
Year	2020
Journal	PLOS ONE
DOI	10.1371/journal.pone.0236003
Date received	2020-04-23
Date accepted	2020-06-27
**Study setting**	
Number of populations included	single
Level of populations included	national (South Africa)
Geographic areas covered	Central and South America
Number of countries covered	single country (South Africa)
Study period	end date in growth phase of wave
**Outcome**	
Raw outcome	epidemiological population-level outcome (cases)
Time resolution of raw outcome	daily
Computed outcome	epidemiological parameter (transmission rate)
Method to obtain the computed outcome	compartmental transmission model (single-population)
Data source	data from (sub)national authorities
Data availability	data made available by the authors
**Intervention**	
Terminology for interventions	measures
Terminology for the specific type of non-pharmaceutical interventions	social distancing
Exposure types	one combination of interventions (lockdown issued on March 26)

– continued on next page –

Types of single interventions	– not applicable –
Coding of interventions	not necessary
Source of intervention data	– could not be evaluated –
Availability of data on exposure	raw data documented in the manuscript
**Methodological approach**	
Empirical approach	descriptive
Use of exposure variation	only variation over time for a single population
Method	description of change over time (comparison of time periods)
Code availability	none
**Effectiveness assessment**	
Reporting of intervention effectiveness	quantification of change in outcome values
Measure of effectiveness	transmission rate
Interpretation of results	implicitly causal
Reporting of uncertainty	yes
Sensitivity analysis	none
Subgroup assessment	none

Munayco et al. 2020

Study information

– continued on next page –

Author	Munayco CV, Tariq A, Rothenberg R, Soto-Cabezas GG, Reyes MF, Valle A, Rojas-Mezarina L, Cabezas C, Loayza M, Chowell G, Garro DC, Vasquez KM, Castro ES, Ordinola IS, Mimbela JM, Cornejo KM, Quijano FC, Rosillo LT, Ibarguen LO, Dominguez MV, Seminario RV, Silva MC, Dreyfus MS, Pineda ML, Durand M, Janampa N, Chuquihuaccha J, Lizarbe SM, Cusi DE, Pilco IM, Jaramillo A, Vargas K, Cabanillas O, Arrasco J, Vargas M, Ramos W
Title	Early Transmission Dynamics of COVID-19 in a Southern Hemisphere Setting: Lima-Peru: February 29th–March 30th, 2020
Year	2020
Journal	Infectious Disease Modelling
DOI	10.1016/j.idm.2020.05.001
Date received	2020-04-24
Date accepted	2020-05-07
**Study setting**	
Number of populations included	single
Level of populations included	subnational (Lima (Peru))
Geographic areas covered	Central and South America
Number of countries covered	single country (Peru)
Study period	start and end date span first epidemic wave
**Outcome**	
Raw outcome	epidemiological population-level outcome (cases)
Time resolution of raw outcome	daily
Computed outcome	none
Method to obtain the computed outcome	– not applicable –
Data source	data from (sub)national authorities

– continued on next page –

Data availability	data access via source
**Intervention**	
Terminology for interventions	measures
Terminology for the specific type of non-pharmaceutical interventions	social distancing
Exposure types	all interventions together (school closure, emergency declaration (national), other social distancing interventions)
Types of single interventions	– not applicable –
Coding of interventions	– not applicable –
Source of intervention data	– not applicable –
Availability of data on exposure	– not applicable –
**Methodological approach**	
Empirical approach	counterfactual
Use of exposure variation	only variation over time for a single population
Method	non-mechanistic model (exponential growth model)
Code availability	none
**Effectiveness assessment**	
Reporting of intervention effectiveness	qualitative statement
Measure of effectiveness	cases
Interpretation of results	implicitly causal
Reporting of uncertainty	– not applicable –
Sensitivity analysis	none
Subgroup assessment	none

Murillo-Zamora et al. 2020	
**Study information**	
Author	Murillo-Zamora E, Guzmán-Esquivel J, Sánchez-Piña RA, Cedeño-Laurent G, Delgado-Enciso I, Mendoza-Cano O
Title	Physical Distancing Reduced the Incidence of Influenza and Supports a Favorable Impact on SARS-CoV-2 Spread in Mexico
Year	2020
Journal	The Journal of Infection in Developing Countries
DOI	10.3855/jidc.13250
Date received	2020-06-12
Date accepted	2020-08-11
**Study setting**	
Number of populations included	multiple
Level of populations included	subnational (Maxican states)
Geographic areas covered	Central and South America
Number of countries covered	single country (Mexico)
Study period	end date in growth phase of wave
**Outcome**	
Raw outcome	epidemiological population-level outcome (cases)
Time resolution of raw outcome	daily
Computed outcome	measure of epidemic trend (growth rate)
Method to obtain the computed outcome	simple computation
Data source	data from (sub)national authorities
Data availability	data access via source
**Intervention**	
Terminology for interventions	measures

– continued on next page –

Terminology for the specific type of non-pharmaceutical interventions	physical distancing
Exposure types	one single intervention (school closure)
Types of single interventions	school closure
Coding of interventions	not necessary
Source of intervention data	government or news websites
Availability of data on exposure	raw data documented in the manuscript
**Methodological approach**	
Empirical approach	descriptive
Use of exposure variation	both variation over time and between populations
Method	comparison of populations
Code availability	none
**Effectiveness assessment**	
Reporting of intervention effectiveness	quantification of change in outcome values
Measure of effectiveness	growth rate
Interpretation of results	implicitly causal
Reporting of uncertainty	yes
Sensitivity analysis	none
Subgroup assessment	socioeconomic indicators (age), public health response (response time)

Ng et al. 2020

**Study information**	
Author	Ng Y, Li Z, Chua YX, Chaw WL, Zhao Z, Er B, Pung R, Chiew CJ, Lye DC, Heng D, Lee VJ

– continued on next page –

Title	Evaluation of the Effectiveness of Surveillance and Containment Measures for the First 100 Patients with COVID-19 in Singapore —January 2–February 29, 2020
Year	2020
Journal	Morbidity and Mortality Weekly Report
DOI	10.15585/mmwr.mm6911e1
Date received	– could not be evaluated –
Date accepted	2020-03-20 (published)
**Study setting**	
Number of populations included	single
Level of populations included	national (Singapore)
Geographic areas covered	Asia
Number of countries covered	single country (Singapore)
Study period	end date in growth phase of wave
**Outcome**	
Raw outcome	epidemiological individual-level outcome (individual cases)
Time resolution of raw outcome	daily
Computed outcome	epidemiological parameter (time from symptom onset to isolation)
Method to obtain the computed outcome	simple computation
Data source	data from (sub)national authorities
Data availability	data not accessible
**Intervention**	
Terminology for interventions	measures
Terminology for the specific type of non-pharmaceutical interventions	surveillance, containment

– continued on next page –

Exposure types	all interventions together
Types of single interventions	– not applicable –
Coding of interventions	– not applicable –
Source of intervention data	– not applicable –
Availability of data on exposure	– not applicable –
**Methodological approach**	
Empirical approach	descriptive
Use of exposure variation	only variation over time for a single population
Method	description of change over time (comparison of time periods)
Code availability	none
**Effectiveness assessment**	
Reporting of intervention effectiveness	quantification of change in outcome values
Measure of effectiveness	time from symptom onset to isolation
Interpretation of results	implicitly causal
Reporting of uncertainty	no
Sensitivity analysis	none
Subgroup assessment	epidemiological indicators (source of infection)

Niazkar et al. 2020 (Analysis 1)

**Study information**	
Author	Niazkar M, Türkkan GE, Niazkar HR, Türkkan YA

– continued on next page –

Title	Assessment of Three Mathematical Prediction Models for Forecasting the COVID-19 Outbreak in Iran and Turkey
Year	2020
Journal	Computational and Mathematical Methods in Medicine
DOI	10.1155/2020/7056285
Date received	2020-07-27
Date accepted	2020-11-03
**Study setting**	
Number of populations included	single
Level of populations included	national (Iran)
Geographic areas covered	Middle East and Africa
Number of countries covered	single country (Iran)
Study period	start and end date span first epidemic wave
**Outcome**	
Raw outcome	epidemiological population-level outcome (cases)
Time resolution of raw outcome	daily
Computed outcome	none
Method to obtain the computed outcome	– not applicable –
Data source	data from publicly available cross-country selections (WHO)
Data availability	data access via source
**Intervention**	
Terminology for interventions	measures
Terminology for the specific type of non-pharmaceutical interventions	control
Exposure types	one single intervention (workplace closure (reopening of low-risk businesses))

– continued on next page –

Types of single interventions	workplace closure
Coding of interventions	not necessary
Source of intervention data	government or news websites
Availability of data on exposure	raw data documented in the manuscript
**Methodological approach**	
Empirical approach	counterfactual
Use of exposure variation	only variation over time for a single population
Method	non-mechanistic model (exponential growth model)
Code availability	none
**Effectiveness assessment**	
Reporting of intervention effectiveness	quantification of change in outcome values
Measure of effectiveness	cases
Interpretation of results	implicitly causal
Reporting of uncertainty	no
Sensitivity analysis	none
Subgroup assessment	none

**Niazkar et al. 2020 (Analysis 2)**	
**Study information**	
Author	Niazkar M, Türkkan GE, Niazkar HR, Türkkan YA
Title	Assessment of Three Mathematical Prediction Models for Forecasting the COVID-19 Outbreak in Iran and Turkey
Year	2020
Journal	Computational and Mathematical Methods in Medicine

– continued on next page –

DOI	10.1155/2020/7056285
Date received	2020-07-27
Date accepted	2020-11-03
**Study setting**	
Number of populations included	single
Level of populations included	national (Turkey)
Geographic areas covered	Middle East and Africa
Number of countries covered	single country (Turkey)
Study period	end date in growth phase of wave
**Outcome**	
Raw outcome	epidemiological population-level outcome (cases)
Time resolution of raw outcome	daily
Computed outcome	none
Method to obtain the computed outcome	– not applicable –
Data source	data from (sub)national authorities
Data availability	data access via source
**Intervention**	
Terminology for interventions	measures
Terminology for the specific type of non-pharmaceutical interventions	control
Exposure types	one combination of interventions (curfew, bans of large gatherings (canelling of sport events), venue closure (closure of mosques))
Types of single interventions	– not applicable –
Coding of interventions	not necessary
Source of intervention data	government or news websites

– continued on next page –

Availability of data on exposure	raw data documented in the manuscript
**Methodological approach**	
Empirical approach	counterfactual
Use of exposure variation	only variation over time for a single population
Method	non-mechanistic model (exponential growth model)
Code availability	none
**Effectiveness assessment**	
Reporting of intervention effectiveness	quantification of change in outcome values
Measure of effectiveness	cases
Interpretation of results	implicitly causal
Reporting of uncertainty	no
Sensitivity analysis	none
Subgroup assessment	none

Nie et al. 2020

**Study information**	
Author	Nie L, Guo AX, Yi C, And RW
Title	Analyzing the Effects of Public Interventions on Reducing Public Gatherings in China during the COVID-19 Epidemic via Mobile Terminals Positioning Data
Year	2020
Journal	Mathematical Biosciences and Engineering
DOI	10.3934/mbe.2020265
Date received	2020-05-07

– continued on next page –

Date accepted	2020-07-09
**Study setting**	
Number of populations included	multiple
Level of populations included	both national and subnational (China and its 31 provinces)
Geographic areas covered	Asia
Number of countries covered	single country (China)
Study period	start and end date span first epidemic wave: behavioral
**Outcome**	
Raw outcome	behavioral outcome (mobility: number of mobile terminals with specific points of interest)
Time resolution of raw outcome	daily
Computed outcome	none
Method to obtain the computed outcome	– not applicable –
Data source	– could not be evaluated –
Data availability	data not accessible
**Intervention**	
Terminology for interventions	interventions
Terminology for the specific type of non-pharmaceutical interventions	public health
Exposure types	multiple combinations of interventions (five strategies involving work-frome-home orders and venue closure as well as their partial resumption)
Types of single interventions	– not applicable –
Coding of interventions	not necessary
Source of intervention data	– could not be evaluated –
Availability of data on exposure	raw data documented in the manuscript

– continued on next page –

**Methodological approach**	
Empirical approach	parametric
Use of exposure variation	only variation over time for a single population
Method	non-mechanistic model (generalized linear model)
Code availability	none
**Effectiveness assessment**	
Reporting of intervention effectiveness	quantification of change in outcome values
Measure of effectiveness	mobility: number of people in workplace or consumption areas
Interpretation of results	implicitly causal
Reporting of uncertainty	yes
Sensitivity analysis	none
Subgroup assessment	none

**Niwa et al. 2020**	
**Study information**	
Author	Niwa M, Hara Y, Sengoku S, Kodama K
Title	Effectiveness of Social Measures against COVID-19 Outbreaks in Selected Japanese Regions Analyzed by System Dynamic Modeling
Year	2020
Journal	International Journal of Environmental Research and Public Health
DOI	10.3390/ijerph17176238
Date received	2020-07-17
Date accepted	2020-08-19

– continued on next page –

**Study setting**	
Number of populations included	multiple
Level of populations included	subnational (3 regions in Japan (Tokyo metropolitan area, Osaka prefecture, Hokkaido prefecture))
Geographic areas covered	Asia
Number of countries covered	single country (Japan)
Study period	start and end date span first epidemic wave
**Outcome**	
Raw outcome	epidemiological population-level outcome (cases)
Time resolution of raw outcome	daily
Computed outcome	epidemiological parameter (other: transmission efficiency)
Method to obtain the computed outcome	other (stock-and-flow model based on system dynamics)
Data source	data from (sub)national authorities
Data availability	data made available by the authors
**Intervention**	
Terminology for interventions	measures
Terminology for the specific type of non-pharmaceutical interventions	control
Exposure types	all interventions together
Types of single interventions	– not applicable –
Coding of interventions	– not applicable –
Source of intervention data	– not applicable –
Availability of data on exposure	– not applicable –
**Methodological approach**	
Empirical approach	descriptive

– continued on next page –

Use of exposure variation	only variation over time for multiple populations
Method	description of change over time (comparison of time periods)
Code availability	none
**Effectiveness assessment**	
Reporting of intervention effectiveness	quantification of change in outcome values
Measure of effectiveness	transmission efficiency
Interpretation of results	explicitly causal
Reporting of uncertainty	no
Sensitivity analysis	none
Subgroup assessment	none

Odagaki 2020

**Study information**	
Author	Odagaki T
Title	Analysis of the Outbreak of COVID-19 in Japan by SIQR Model
Year	2020
Journal	Infectious Disease Modelling
DOI	10.1016/j.idm.2020.08.013
Date received	2020-06-11
Date accepted	2020-08-28
**Study setting**	
Number of populations included	single
Level of populations included	national (Japan)

– continued on next page –

Geographic areas covered	Asia
Number of countries covered	single country (Japan)
Study period	start and end date span first epidemic wave
**Outcome**	
Raw outcome	epidemiological population-level outcome (cases)
Time resolution of raw outcome	daily
Computed outcome	epidemiological parameter (transmission rate)
Method to obtain the computed outcome	compartmental transmission model (single-population)
Data source	data from (sub)national authorities
Data availability	data access via source
**Intervention**	
Terminology for interventions	measures
Terminology for the specific type of non-pharmaceutical interventions	lockdown, quarantine
Exposure types	one single intervention (emergency declaration)
Types of single interventions	declaration of a state of emergency
Coding of interventions	not necessary
Source of intervention data	– could not be evaluated –
Availability of data on exposure	raw data documented in the manuscript
**Methodological approach**	
Empirical approach	descriptive
Use of exposure variation	only variation over time for a single population
Method	description of change over time (comparison of time periods)
Code availability	none
**Effectiveness assessment**	

– continued on next page –

Reporting of intervention effectiveness	quantification of change in outcome values
Measure of effectiveness	transmission rate, reproduction number
Interpretation of results	implicitly causal
Reporting of uncertainty	no
Sensitivity analysis	none
Subgroup assessment	none

Ouchetto et al. 2020

**Study information**	
Author	Ouchetto O, Bourhanbour AD, Boumhamdi M
Title	Effectiveness of Containment Measures to Control the Spread of COVID-19 in North Africa
Year	2020
Journal	Disaster Medicine and Public Health Preparedness
DOI	10.1017/dmp.2020.314
Date received	– could not be evaluated –
Date accepted	2020-09-03 (published)
**Study setting**	
Number of populations included	multiple
Level of populations included	national (Morocco, Algeria, Tunisia)
Geographic areas covered	Middle East and Africa
Number of countries covered	multiple countries
Study period	end date at peak of wave

– continued on next page –

**Outcome**	
Raw outcome	epidemiological population-level outcome (cases)
Time resolution of raw outcome	daily
Computed outcome	epidemiological parameter (reproduction number)
Method to obtain the computed outcome	statistical estimation of reproduction number (Wallinga and Teunis)
Data source	data from publicly available cross-country selections (ECDC)
Data availability	data access via source
**Intervention**	
Terminology for interventions	measures
Terminology for the specific type of non-pharmaceutical interventions	containment
Exposure types	one single intervention (stay-at-home order)
Types of single interventions	stay-at-home order
Coding of interventions	not necessary
Source of intervention data	– could not be evaluated –
Availability of data on exposure	raw data documented in the manuscript
**Methodological approach**	
Empirical approach	descriptive
Use of exposure variation	only variation over time for multiple populations
Method	description of change over time (comparison of time periods)
Code availability	none
**Effectiveness assessment**	
Reporting of intervention effectiveness	comparison of outcome values
Measure of effectiveness	reproduction number

– continued on next page –

Interpretation of results	implicitly causal
Reporting of uncertainty	no
Sensitivity analysis	none
Subgroup assessment	none

Pan et al. 2020a

**Study information**	
Author	Pan Y, Darzi A, Kabiri A, Zhao G, Luo W, Xiong C, Zhang L
Title	Quantifying Human Mobility Behaviour Changes during the COVID-19 Outbreak in the United States
Year	2020
Journal	Scientific Reports
DOI	10.1038/s41598-020-77751-2
Date received	2020-06-18
Date accepted	2020-11-03
**Study setting**	
Number of populations included	multiple
Level of populations included	both national and subnational (US and all states)
Geographic areas covered	North America
Number of countries covered	single country (US)
Study period	start and end date span first epidemic wave: behavioral
**Outcome**	
Raw outcome	behavioral outcome (mobility: percentage staying at home & daily work trips & non-work trips & distance travelled & out-of-county trips)

– continued on next page –

Time resolution of raw outcome	daily
Computed outcome	none
Method to obtain the computed outcome	– not applicable –
Data source	public (Maryland Transportation Institute led by the University of Maryland)
Data availability	data made available by the authors
**Intervention**	
Terminology for interventions	orders
Terminology for the specific type of non-pharmaceutical interventions	government
Exposure types	multiple separate interventions (stay-at-home order, emergency declaration (national))
Types of single interventions	stay-at-home order, declaration of a state of emergency
Coding of interventions	necessary
Source of intervention data	– could not be evaluated –
Availability of data on exposure	coded data not available
**Methodological approach**	
Empirical approach	descriptive
Use of exposure variation	only variation over time for multiple populations
Method	description of change over time (description of time course)
Code availability	publicly available
**Effectiveness assessment**	
Reporting of intervention effectiveness	qualitative statement
Measure of effectiveness	mobility: social distancing index

– continued on next page –

Interpretation of results	implicitly causal
Reporting of uncertainty	– not applicable –
Sensitivity analysis	none
Subgroup assessment	none

**Pan et al. 2020b**	
**Study information**	
Author	Pan A, Liu L, Wang C, Guo H, Hao X, Wang Q, Huang J, He N, Yu H, Lin X, Wei S, Wu T
Title	Association of Public Health Interventions with the Epidemiology of the COVID-19 Outbreak in Wuhan, China
Year	2020
Journal	JAMA
DOI	10.1001/jama.2020.6130
Date received	– could not be evaluated –
Date accepted	2020-04-10 (published)
**Study setting**	
Number of populations included	single
Level of populations included	subnational (Wuhan)
Geographic areas covered	Asia
Number of countries covered	single country (China)
Study period	start and end date span first epidemic wave
**Outcome**	
Raw outcome	epidemiological individual-level outcome (individual cases)

– continued on next page –

Time resolution of raw outcome	daily
Computed outcome	epidemiological parameter (reproduction number)
Method to obtain the computed outcome	statistical estimation of reproduction number (Cori et al.)
Data source	data from (sub)national authorities
Data availability	data not accessible
**Intervention**	
Terminology for interventions	interventions
Terminology for the specific type of non-pharmaceutical interventions	public health
Exposure types	one combination of interventions (Wuhan City Lockdown, centralized quarantine and treatment, universal symptom survey)
Types of single interventions	– not applicable –
Coding of interventions	not necessary
Source of intervention data	– could not be evaluated –
Availability of data on exposure	raw data documented in the manuscript
**Methodological approach**	
Empirical approach	descriptive
Use of exposure variation	only variation over time for a single population
Method	description of change over time (comparison of time periods)
Code availability	none
**Effectiveness assessment**	
Reporting of intervention effectiveness	comparison of outcome values
Measure of effectiveness	cases, reproduction number
Interpretation of results	associative

– continued on next page –

Reporting of uncertainty	yes
Sensitivity analysis	none
Subgroup assessment	socioeconomic indicators (age, gender, health care occupation), epidemiological indicators (disease severity)

**Pang et al. 2020**	
**Study information**	
Author	Pang L, Liu S, Zhang X, Tian T, Zhao Z
Title	Transmission Dynamics and Control Strategies of COVID-19 in Wuhan, China
Year	2020
Journal	Journal of Biological Systems
DOI	10.1142/s0218339020500096
Date received	2020-02-23
Date accepted	2020-04-14
**Study setting**	
Number of populations included	single
Level of populations included	subnational (Wuhan)
Geographic areas covered	Asia
Number of countries covered	single country (China)
Study period	start and end date span first epidemic wave
**Outcome**	
Raw outcome	epidemiological population-level outcome (cases)
Time resolution of raw outcome	daily

– continued on next page –

Computed outcome	epidemiological parameter (reproduction number, transmission rate)
Method to obtain the computed outcome	compartmental transmission model (single-population)
Data source	data from (sub)national authorities
Data availability	data access via source
**Intervention**	
Terminology for interventions	measures
Terminology for the specific type of non-pharmaceutical interventions	control
Exposure types	one combination of interventions (Wuhan City Lockdown and strict quarantine measures)
Types of single interventions	– not applicable –
Coding of interventions	not necessary
Source of intervention data	– could not be evaluated –
Availability of data on exposure	raw data documented in the manuscript
**Methodological approach**	
Empirical approach	descriptive
Use of exposure variation	only variation over time for a single population
Method	description of change over time (comparison of time periods)
Code availability	none
**Effectiveness assessment**	
Reporting of intervention effectiveness	comparison of outcome values
Measure of effectiveness	reproduction number, transmission rate
Interpretation of results	implicitly causal
Reporting of uncertainty	yes

– continued on next page –

Sensitivity analysis	none
Subgroup assessment	none

**Pei et al. 2020**	
**Study information**	
Author	Pei S, Kandula S, Shaman J
Title	Differential Effects of Intervention Timing on COVID-19 Spread in the United States
Year	2020
Journal	Science Advances
DOI	10.1126/sciadv.abd6370
Date received	2020-07-02
Date accepted	2020-10-20
**Study setting**	
Number of populations included	multiple
Level of populations included	both national and subnational (US counties)
Geographic areas covered	North America
Number of countries covered	single country (US)
Study period	same end date for several populations with diverse epidemic trajectories
**Outcome**	
Raw outcome	epidemiological population-level outcome (cases, deaths)
Time resolution of raw outcome	daily
Computed outcome	epidemiological parameter (reproduction number)

– continued on next page –

Method to obtain the computed outcome	compartmental transmission model (single-population)
Data source	public (USAFacts)
Data availability	data made available by the authors
**Intervention**	
Terminology for interventions	measures
Terminology for the specific type of non-pharmaceutical interventions	social distancing, control
Exposure types	all interventions together
Types of single interventions	– not applicable –
Coding of interventions	– not applicable –
Source of intervention data	– not applicable –
Availability of data on exposure	– not applicable –
**Methodological approach**	
Empirical approach	descriptive
Use of exposure variation	only variation over time for multiple populations
Method	description of change over time (description of time course)
Code availability	publicly available
**Effectiveness assessment**	
Reporting of intervention effectiveness	comparison of outcome values
Measure of effectiveness	reproduction number
Interpretation of results	associative
Reporting of uncertainty	yes
Sensitivity analysis	model specification varied, epidemiological parameters varied
Subgroup assessment	none

Peixoto et al. 2020	
**Study information**	
Author	Peixoto VR, Vieira A, Aguiar P, Carvalho C, Thomas DR, Abrantes A
Title	Initial Assessment of the Impact of the Emergency State Lockdown Measures on the 1st Wave of the COVID-19 Epidemic in Portugal
Year	2020
Journal	Acta Médica Portuguesa
DOI	10.20344/amp.14129
Date received	2020-05-13
Date accepted	2020-07-31
**Study setting**	
Number of populations included	single
Level of populations included	national (Portugal)
Geographic areas covered	Europe
Number of countries covered	single country (Portugal)
Study period	end date at peak of wave
**Outcome**	
Raw outcome	epidemiological population-level outcome (cases, deaths, hospitalizations, ICU hospitalizations)
Time resolution of raw outcome	daily
Computed outcome	none
Method to obtain the computed outcome	– not applicable –
Data source	data from (sub)national authorities
Data availability	data access via source

– continued on next page –

**Intervention**	
Terminology for interventions	measures
Terminology for the specific type of non-pharmaceutical interventions	containment
Exposure types	one combination of interventions (lockdown measures decreed on March 16)
Types of single interventions	– not applicable –
Coding of interventions	not necessary
Source of intervention data	– could not be evaluated –
Availability of data on exposure	raw data documented in the manuscript
**Methodological approach**	
Empirical approach	counterfactual
Use of exposure variation	only variation over time for a single population
Method	non-mechanistic model (time series model)
Code availability	none
**Effectiveness assessment**	
Reporting of intervention effectiveness	quantification of change in outcome values
Measure of effectiveness	cases, deaths, hospitalizations, ICU hospitalizations
Interpretation of results	implicitly causal
Reporting of uncertainty	no
Sensitivity analysis	none
Subgroup assessment	none

**Peng et al. 2020**	
**Study information**	
Author	Peng Z, Song W, Ding Z, Guan Q, Yang X, Xu Q, Wang X, Xia Y
Title	Linking Key Intervention Timings to Rapid Declining Effective Reproduction Number to Quantify Lessons against COVID-19
Year	2020
Journal	Frontiers of Medicine
DOI	10.1007/s11684-020-0788-3
Date received	2020-03-20
Date accepted	2020-04-08
**Study setting**	
Number of populations included	multiple
Level of populations included	national (China, Italy, Iran, South Korea, Japan)
Geographic areas covered	Asia, Europe, Middle East and Africa
Number of countries covered	multiple countries
Study period	end date in growth phase of wave
**Outcome**	
Raw outcome	epidemiological population-level outcome (cases)
Time resolution of raw outcome	daily
Computed outcome	epidemiological parameter (reproduction number)
Method to obtain the computed outcome	statistical estimation of reproduction number (White et al., Walinga et al., Cori et al.)
Data source	data from (sub)national authorities (China), data from publicly available cross-country selections (WHO: other countries)
Data availability	data access via source
**Intervention**	
Terminology for interventions	measures

– continued on next page –

Terminology for the specific type of non-pharmaceutical interventions	prevention, control
Exposure types	all interventions together
Types of single interventions	– not applicable –
Coding of interventions	– not applicable –
Source of intervention data	– not applicable –
Availability of data on exposure	– not applicable –
**Methodological approach**	
Empirical approach	descriptive
Use of exposure variation	only variation over time for multiple populations
Method	description of change over time (description of time course)
Code availability	none
**Effectiveness assessment**	
Reporting of intervention effectiveness	qualitative statement
Measure of effectiveness	reproduction number
Interpretation of results	associative
Reporting of uncertainty	– not applicable –
Sensitivity analysis	none
Subgroup assessment	none

Pépin et al. 2020

**Study information**	
Author	Pépin JL, Bruno RM, Yang RY, Vercamer V, Jouhaud P, Escourrou P, Boutouyrie P

– continued on next page –

Title	Wearable Activity Trackers for Monitoring Adherence to Home Confinement During the COVID-19 Pandemic Worldwide: Data Aggregation and Analysis
Year	2020
Journal	Journal of Medical Internet Research
DOI	10.2196/19787
Date received	2020-05-01
Date accepted	2020-06-04
**Study setting**	
Number of populations included	multiple
Level of populations included	both national and subnational (Multiple countries around the world including some subnational regions)
Geographic areas covered	Asia, Oceania, Europe, North America
Number of countries covered	multiple countries
Study period	start and end date span first epidemic wave: behavioral
**Outcome**	
Raw outcome	behavioral outcome (mobility: number of steps made)
Time resolution of raw outcome	daily
Computed outcome	none
Method to obtain the computed outcome	– not applicable –
Data source	mobility data from corporate organizations (Withings)
Data availability	data not accessible
**Intervention**	
Terminology for interventions	policies

– continued on next page –

Terminology for the specific type of non-pharmaceutical interventions	confinement
Exposure types	multiple combinations of interventions (lockdown: stay-at-home order, partial lockdown: e.g. school closures, venue closures, bans of gatherings)
Types of single interventions	– not applicable –
Coding of interventions	necessary
Source of intervention data	– could not be evaluated –
Availability of data on exposure	coded data made available by the authors
**Methodological approach**	
Empirical approach	descriptive
Use of exposure variation	only variation over time for multiple populations
Method	description of change over time (comparison of time periods)
Code availability	none
**Effectiveness assessment**	
Reporting of intervention effectiveness	quantification of change in outcome values
Measure of effectiveness	mobility: number of steps made
Interpretation of results	associative
Reporting of uncertainty	yes
Sensitivity analysis	none
Subgroup assessment	none

Piovani et al. 2021

Study information

– continued on next page –

Author	Piovani D, Christodoulou MN, Hadjidemetriou A, Pantavou K, Zaza P, Bagos PG, Bonovas S, Nikolopoulos GK
Title	Effect of Early Application of Social Distancing Interventions on COVID-19 Mortality over the First Pandemic Wave: An Analysis of Longitudinal Data from 37 Countries
Year	2021
Journal	Journal of Infection
DOI	10.1016/j.jinf.2020.11.033
Date received	– could not be evaluated –
Date accepted	2020-11-28
**Study setting**	
Number of populations included	multiple
Level of populations included	national (37 OECD countries)
Geographic areas covered	Europe, Middle East and Africa, Asia, Oceania, Central and South America, North America
Number of countries covered	multiple countries
Study period	same end date for several populations with diverse epidemic trajectories
**Outcome**	
Raw outcome	epidemiological population-level outcome (deaths)
Time resolution of raw outcome	daily
Computed outcome	none
Method to obtain the computed outcome	– not applicable –
Data source	data from publicly available cross-country selections (WHO)
Data availability	data access via source
**Intervention**	

– continued on next page –

Terminology for interventions	measures
Terminology for the specific type of non-pharmaceutical interventions	social distancing
Exposure types	one single intervention (bans of large gatherings, school closure)
Types of single interventions	bans of large gatherings, school closure
Coding of interventions	necessary
Source of intervention data	use of externally coded data (OxCGRT)
Availability of data on exposure	access to externally coded data via source
**Methodological approach**	
Empirical approach	parametric
Use of exposure variation	both variation over time and between populations
Method	non-mechanistic model (generalized linear model)
Code availability	none
**Effectiveness assessment**	
Reporting of intervention effectiveness	quantification of change in outcome values
Measure of effectiveness	deaths
Interpretation of results	associative
Reporting of uncertainty	yes
Sensitivity analysis	epidemiological parameters varied
Subgroup assessment	socioeconomic indicators (age, population density), epidemiological indicators (pre-intervention epidemic trend)

Price et al. 2020	
**Study information**	
Author	Price DJ, Shearer FM, Meehan MT, McBryde E, Moss R, Golding N, Conway EJ, Dawson P, Cromer D, Wood J, Abbott S, McVernon J, McCaw JM
Title	Early Analysis of the Australian COVID-19 Epidemic
Year	2020
Journal	eLife
DOI	10.7554/elife.58785
Date received	2020-05-11
Date accepted	2020-08-12
**Study setting**	
Number of populations included	multiple
Level of populations included	both national and subnational (Australia and its states)
Geographic areas covered	Oceania
Number of countries covered	single country (Australia)
Study period	start and end date span first epidemic wave
**Outcome**	
Raw outcome	epidemiological population-level outcome (cases, deaths)
Time resolution of raw outcome	daily
Computed outcome	epidemiological parameter (reproduction number)
Method to obtain the computed outcome	statistical estimation of reproduction number (Cori et al.)
Data source	data from (sub)national authorities
Data availability	data made available by the authors
**Intervention**	
Terminology for interventions	interventions

– continued on next page –

Terminology for the specific type of non-pharmaceutical interventions	public health
Exposure types	all interventions together (timeline of measures)
Types of single interventions	– not applicable –
Coding of interventions	– not applicable –
Source of intervention data	– not applicable –
Availability of data on exposure	– not applicable –
**Methodological approach**	
Empirical approach	descriptive
Use of exposure variation	only variation over time for multiple populations
Method	description of change over time (description of time course)
Code availability	publicly available
**Effectiveness assessment**	
Reporting of intervention effectiveness	qualitative statement
Measure of effectiveness	cases, reproduction number
Interpretation of results	associative
Reporting of uncertainty	– not applicable –
Sensitivity analysis	different or modified outcome used (percentage of imported cases varied)
Subgroup assessment	none

Pullano et al. 2020 (Analysis 1)

Study information

– continued on next page –

Author	Pullano G, Valdano E, Scarpa N, Rubrichi S, Colizza V
Title	Evaluating the Effect of Demographic Factors, Socioeconomic Factors, and Risk Aversion on Mobility during the COVID-19 Epidemic in France under Lockdown: A Population-Based Study
Year	2020
Journal	The Lancet Digital Health
DOI	10.1016/s2589-7500(20)30243-0
Date received	– could not be evaluated –
Date accepted	2020-10-28 (published)
**Study setting**	
Number of populations included	multiple
Level of populations included	both national and subnational (France, regions)
Geographic areas covered	Europe
Number of countries covered	single country (France)
Study period	start and end date span first epidemic wave: behavioral
**Outcome**	
Raw outcome	behavioral outcome (mobility: number of trips)
Time resolution of raw outcome	daily
Computed outcome	none
Method to obtain the computed outcome	– not applicable –
Data source	mobility data from corporate organizations (Orange Business Service Flux Vision)
Data availability	data not accessible
**Intervention**	
Terminology for interventions	– not applicable –

– continued on next page –

Terminology for the specific type of non-pharmaceutical interventions	lockdown
Exposure types	phases of interventions (phase 2: school closure, gathering bans, public transport bans, phase 3: nationwide restrictions culminating in lockdown)
Types of single interventions	– not applicable –
Coding of interventions	not necessary
Source of intervention data	– could not be evaluated –
Availability of data on exposure	raw data documented in the manuscript
**Methodological approach**	
Empirical approach	descriptive
Use of exposure variation	only variation over time for multiple populations
Method	description of change over time (comparison of time periods)
Code availability	none
**Effectiveness assessment**	
Reporting of intervention effectiveness	quantification of change in outcome values
Measure of effectiveness	mobility: number of trips
Interpretation of results	implicitly causal
Reporting of uncertainty	no
Sensitivity analysis	none
Subgroup assessment	socioeconomic indicators (age)

Pullano et al. 2020 (Analysis 2)

**Study information**	
Author	Pullano G, Valdano E, Scarpa N, Rubrichi S, Colizza V

– continued on next page –

Title	Evaluating the Effect of Demographic Factors, Socioeconomic Factors, and Risk Aversion on Mobility during the COVID-19 Epidemic in France under Lockdown: A Population-Based Study
Year	2020
Journal	The Lancet Digital Health
DOI	10.1016/s2589-7500(20)30243-0
Date received	– could not be evaluated –
Date accepted	2020-10-28 (published)
**Study setting**	
Number of populations included	multiple
Level of populations included	both national and subnational (France, regions)
Geographic areas covered	Europe
Number of countries covered	single country (France)
Study period	start and end date span first epidemic wave: behavioral
**Outcome**	
Raw outcome	behavioral outcome (mobility: number of trips)
Time resolution of raw outcome	daily
Computed outcome	none
Method to obtain the computed outcome	– not applicable –
Data source	mobility data from corporate organizations (Orange Business Service Flux Vision)
Data availability	data not accessible
**Intervention**	
Terminology for interventions	– not applicable –

– continued on next page –

Terminology for the specific type of non-pharmaceutical interventions	lockdown
Exposure types	phases of interventions (Combining phase 2: school closure, gathering bans, public transport bans, and phase 3: nationwide restrictions culminating in lockdown)
Types of single interventions	– not applicable –
Coding of interventions	not necessary
Source of intervention data	– could not be evaluated –
Availability of data on exposure	raw data documented in the manuscript
**Methodological approach**	
Empirical approach	counterfactual
Use of exposure variation	only variation over time for multiple populations
Method	non-mechanistic model (time series model)
Code availability	none
**Effectiveness assessment**	
Reporting of intervention effectiveness	quantification of change in outcome values
Measure of effectiveness	mobility: number of trips
Interpretation of results	implicitly causal
Reporting of uncertainty	yes
Sensitivity analysis	none
Subgroup assessment	socioeconomic indicators (age)

Pullano et al. 2020 (Analysis 3)

Study information

– continued on next page –

Author	Pullano G, Valdano E, Scarpa N, Rubrichi S, Colizza V
Title	Evaluating the Effect of Demographic Factors, Socioeconomic Factors, and Risk Aversion on Mobility during the COVID-19 Epidemic in France under Lockdown: A Population-Based Study
Year	2020
Journal	The Lancet Digital Health
DOI	10.1016/s2589-7500(20)30243-0
Date received	– could not be evaluated –
Date accepted	2020-10-28 (published)
**Study setting**	
Number of populations included	multiple
Level of populations included	both national and subnational (France, regions)
Geographic areas covered	Europe
Number of countries covered	single country (France)
Study period	start and end date span first epidemic wave: behavioral
**Outcome**	
Raw outcome	behavioral outcome (mobility: number of mobility connections between locations)
Time resolution of raw outcome	daily
Computed outcome	none
Method to obtain the computed outcome	– not applicable –
Data source	mobility data from corporate organizations (Orange Business Service Flux Vision)
Data availability	data not accessible
**Intervention**	

– continued on next page –

Terminology for interventions	– not applicable –
Terminology for the specific type of non-pharmaceutical interventions	lockdown
Exposure types	phases of interventions (phase 2: school closure, gathering bans, public transport bans, phase 3: nationwide restrictions culminating in lockdown)
Types of single interventions	– not applicable –
Coding of interventions	not necessary
Source of intervention data	– could not be evaluated –
Availability of data on exposure	raw data documented in the manuscript
**Methodological approach**	
Empirical approach	descriptive
Use of exposure variation	only variation over time for multiple populations
Method	description of change over time (comparison of time periods)
Code availability	none
**Effectiveness assessment**	
Reporting of intervention effectiveness	comparison of outcome values
Measure of effectiveness	mobility: number of mobility connections between locations
Interpretation of results	implicitly causal
Reporting of uncertainty	no
Sensitivity analysis	none
Subgroup assessment	none

Pung et al. 2020	
**Study information**	
Author	Pung R, Cook AR, Chiew CJ, Clapham HE, Sun Y, Li Z, Dickens BL, Ma S, Mak K, Tan CC, Heng D, Chen MI, Lee VJ
Title	Effectiveness of Containment Measures against COVID-19 in Singapore
Year	2020
Journal	Epidemiology
DOI	10.1097/ede.0000000000001257
Date received	2020-05-09
Date accepted	2020-09-03
**Study setting**	
Number of populations included	single
Level of populations included	national (Singapore)
Geographic areas covered	Asia
Number of countries covered	single country (Singapore)
Study period	start and end date span first epidemic wave
**Outcome**	
Raw outcome	epidemiological individual-level outcome (individual cases and transmission chains)
Time resolution of raw outcome	daily
Computed outcome	epidemiological parameter (reproduction number)
Method to obtain the computed outcome	statistical estimation of reproduction number from individual data (Wallinga and Teunis, extended version with Bayesian data augmentation of transmission chains, incorporating individual data)
Data source	data from (sub)national authorities
Data availability	data not accessible
**Intervention**	

– continued on next page –

Terminology for interventions	measures
Terminology for the specific type of non-pharmaceutical interventions	containment
Exposure types	all interventions together
Types of single interventions	– not applicable –
Coding of interventions	– not applicable –
Source of intervention data	– not applicable –
Availability of data on exposure	– not applicable –
**Methodological approach**	
Empirical approach	descriptive
Use of exposure variation	only variation over time for a single population
Method	description of change over time (description of time course)
Code availability	none
**Effectiveness assessment**	
Reporting of intervention effectiveness	qualitative statement
Measure of effectiveness	reproduction number
Interpretation of results	implicitly causal
Reporting of uncertainty	– not applicable –
Sensitivity analysis	none
Subgroup assessment	none

Qiu et al. 2020 (Analysis 1)	
**Study information**	
Author	Qiu Y, Chen X, Shi W
Title	Impacts of Social and Economic Factors on the Transmission of Coronavirus Disease 2019 (COVID-19) in China
Year	2020
Journal	Journal of Population Economics
DOI	10.1007/s00148-020-00778-2
Date received	– could not be evaluated –
Date accepted	2020-05-09 (published)
**Study setting**	
Number of populations included	multiple
Level of populations included	both national and subnational (304 prefecture-level cities (China), excluding Wuhan)
Geographic areas covered	Asia
Number of countries covered	single country (China)
Study period	start and end date span first epidemic wave
**Outcome**	
Raw outcome	epidemiological population-level outcome (cases)
Time resolution of raw outcome	daily
Computed outcome	epidemiological parameter (reproduction number)
Method to obtain the computed outcome	other (fit regression model with lagged cases as predictor and interpret coefficient as transmission rate/reproduction number)
Data source	data from (sub)national authorities
Data availability	data access via source
**Intervention**	
Terminology for interventions	measures

– continued on next page –

Terminology for the specific type of non-pharmaceutical interventions	public health
Exposure types	one combination of interventions (Wuhan City Lockdown)
Types of single interventions	– not applicable –
Coding of interventions	not necessary
Source of intervention data	– could not be evaluated –
Availability of data on exposure	raw data documented in the manuscript
**Methodological approach**	
Empirical approach	descriptive
Use of exposure variation	only variation over time for multiple populations
Method	description of change over time (comparison of time periods)
Code availability	none
**Effectiveness assessment**	
Reporting of intervention effectiveness	comparison of outcome values
Measure of effectiveness	reproduction number
Interpretation of results	explicitly causal
Reporting of uncertainty	no
Sensitivity analysis	model specification varied
Subgroup assessment	none

Qiu et al. 2020 (Analysis 2)

**Study information**	
Author	Qiu Y, Chen X, Shi W

– continued on next page –

Title	Impacts of Social and Economic Factors on the Transmission of Coronavirus Disease 2019 (COVID-19) in China
Year	2020
Journal	Journal of Population Economics
DOI	10.1007/s00148-020-00778-2
Date received	– could not be evaluated –
Date accepted	2020-05-09 (published)
**Study setting**	
Number of populations included	multiple
Level of populations included	both national and subnational (<300 prefecture-level cities (China), excluded cities in Hubei)
Geographic areas covered	Asia
Number of countries covered	single country (China)
Study period	other (starts with start of NPIs)
**Outcome**	
Raw outcome	epidemiological population-level outcome (cases)
Time resolution of raw outcome	daily
Computed outcome	none
Method to obtain the computed outcome	– not applicable –
Data source	data from (sub)national authorities
Data availability	data access via source
**Intervention**	
Terminology for interventions	measures
Terminology for the specific type of non-pharmaceutical interventions	public health

– continued on next page –

Qiu et al. 2020 (Analysis 2) – continued from previous page

Exposure types	multiple separate interventions (bans of small gatherings (closed management of communities), stay-at-home order)
Types of single interventions	bans of small gatherings, stay-at-home order
Coding of interventions	not necessary
Source of intervention data	– could not be evaluated –
Availability of data on exposure	raw data documented in the manuscript
**Methodological approach**	
Empirical approach	parametric
Use of exposure variation	both variation over time and between populations
Method	non-mechanistic model (generalized linear model)
Code availability	none
**Effectiveness assessment**	
Reporting of intervention effectiveness	quantification of change in outcome values
Measure of effectiveness	cases
Interpretation of results	explicitly causal
Reporting of uncertainty	yes
Sensitivity analysis	model specification varied
Subgroup assessment	none

Quaife et al. 2020

Study information

– continued on next page –

Author	Quaife M, van Zandvoort K, Gimma A, Shah K, McCreesh N, Prem K, Barasa E, Mwanga D, Kangwana B, Pinchoff J, Bosse NI, Medley G, O'Reilly K, Leclerc QJ, Jit M, Lowe R, Davies NG, Deol AK, Knight GM, Auzenbergs M, Edmunds WJ, Atkins KE, Jombart T, Eggo RM, Foss AM, Flasche S, Pearson CA, Nightingale ES, Munday JD, Abbott S, Quilty BJ, Gibbs HP, Simons D, Emery JC, Hué S, Liu Y, Tully DC, Rosello A, Procter SR, Endo A, Russell TW, Rees EM, Diamond C, Meakin SR, Kucharski AJ, Gore-Langton GR, Klepac P, Hellewell J, Sun FY, Houben RM, Villabona-Arenas CJ, Funk S, Clifford S, Brady O, Edmunds WJ, Jarvis CI, And KA
Title	The Impact of COVID-19 Control Measures on Social Contacts and Transmission in Kenyan Informal Settlements
Year	2020
Journal	BMC Medicine
DOI	10.1186/s12916-020-01779-4
Date received	2020-06-09
Date accepted	2020-09-09
**Study setting**	
Number of populations included	single
Level of populations included	subnational (informal settlements around Nairobi, Kenia)
Geographic areas covered	Middle East and Africa
Number of countries covered	single country (Kenia)
Study period	other (surveys conducted only in May)
**Outcome**	
Raw outcome	behavioral outcome (survey: number of physical and non-physical contacts)
Time resolution of raw outcome	– not applicable –
Computed outcome	epidemiological parameter (reproduction number)

– continued on next page –

Method to obtain the computed outcome	statistical estimation of reproduction number from individual data (Diekmann et al., contact matrices estimated from survey responses)
Data source	data collected by authors
Data availability	data made available by the authors
**Intervention**	
Terminology for interventions	measures
Terminology for the specific type of non-pharmaceutical interventions	control
Exposure types	all interventions together
Types of single interventions	– not applicable –
Coding of interventions	– not applicable –
Source of intervention data	– not applicable –
Availability of data on exposure	– not applicable –
**Methodological approach**	
Empirical approach	descriptive
Use of exposure variation	only variation over time for a single population
Method	description of change over time (comparison of time periods)
Code availability	publicly available
**Effectiveness assessment**	
Reporting of intervention effectiveness	comparison of outcome values
Measure of effectiveness	reproduction number
Interpretation of results	implicitly causal
Reporting of uncertainty	yes
Sensitivity analysis	model specification varied
Subgroup assessment	none

Rahman et al. 2020 (Analysis 1)	
**Study information**	
Author	Rahman MM, Thill JC, Paul KC
Title	COVID-19 Pandemic Severity, Lockdown Regimes, and People's Mobility: Early Evidence from 88 Countries
Year	2020
Journal	Sustainability
DOI	10.3390/su12219101
Date received	2020-10-02
Date accepted	2020-10-30
**Study setting**	
Number of populations included	multiple
Level of populations included	national (88 countries around the globe)
Geographic areas covered	Europe, Middle East and Africa, Asia, Oceania, Central and South America, North America
Number of countries covered	multiple countries
Study period	start and end date span first epidemic wave: behavioral
**Outcome**	
Raw outcome	behavioral outcome (mobility: time spent in various locations)
Time resolution of raw outcome	daily
Computed outcome	none
Method to obtain the computed outcome	– not applicable –
Data source	mobility data from corporate organizations (Google)

– continued on next page –

Data availability	data access via source
**Intervention**	
Terminology for interventions	– not applicable –
Terminology for the specific type of non-pharmaceutical interventions	lockdown
Exposure types	one combination of interventions (lockdown measures (e.g., restriction on public gathering, workplace closing, and stay-at-home order) with specific dates)
Types of single interventions	– not applicable –
Coding of interventions	necessary
Source of intervention data	use of externally coded data (OxCGRT, and same as in Dunford et al.)
Availability of data on exposure	access to externally coded data via source
**Methodological approach**	
Empirical approach	parametric
Use of exposure variation	both variation over time and between populations
Method	non-mechanistic model (other: structural equation model)
Code availability	none
**Effectiveness assessment**	
Reporting of intervention effectiveness	quantification of change in outcome values
Measure of effectiveness	mobility: time spent in various locations
Interpretation of results	explicitly causal
Reporting of uncertainty	yes
Sensitivity analysis	none
Subgroup assessment	none

Rahman et al. 2020 (Analysis 2, mobility as exposure)	
**Study information**	
Author	Rahman MM, Thill JC, Paul KC
Title	COVID-19 Pandemic Severity, Lockdown Regimes, and People's Mobility: Early Evidence from 88 Countries
Year	2020
Journal	Sustainability
DOI	10.3390/su12219101
Date received	02.10.2020
Date accepted	30.10.2020
**Study setting**	
Number of populations included	multiple
Level of populations included	national (88 countries around the globe)
Geographic areas covered	Europe, Middle East and Africa, Asia, Oceania, Central and South America, North America
Number of countries covered	multiple countries
Study period	same end date for several populations with diverse epidemic trajectories
**Outcome**	
Raw outcome	epidemiological population-level outcome (cases, deaths)
Time resolution of raw outcome	daily
Computed outcome	none
Method to obtain the computed outcome	– not applicable –
Data source	data from publicly available cross-country selections (Worldometer)
Data availability	data access via source
**Intervention**	
Terminology for interventions	– not applicable –

– continued on next page –

Terminology for the specific type of non-pharmaceutical interventions	lockdown
Exposure types	mobility (time spent in various locations)
Types of single interventions	– not applicable –
Coding of interventions	– not applicable –
Source of intervention data	mobility data by corporate organizations (Google)
Availability of data on exposure	data access via source
**Methodological approach**	
Empirical approach	parametric
Use of exposure variation	both variation over time and between populations
Method	non-mechanistic model (generalized linear model)
Code availability	none
**Effectiveness assessment**	
Reporting of intervention effectiveness	quantification of change in outcome values
Measure of effectiveness	cases, deaths
Interpretation of results	explicitly causal
Reporting of uncertainty	yes
Sensitivity analysis	none
Subgroup assessment	none

Reis et al. 2020

**Study information**	
Author	Reis RF, de Melo Quintela B, de Oliveira Campos J, Gomes JM, Rocha BM, Lobosco M, dos Santos RW

– continued on next page –

Title	Characterization of the COVID-19 Pandemic and the Impact of Uncertainties, Mitigation Strategies, and Underreporting of Cases in South Korea, Italy, and Brazil
Year	2020
Journal	Chaos, Solitons & Fractals
DOI	10.1016/j.chaos.2020.109888
Date received	2020-04-11
Date accepted	2020-05-10
**Study setting**	
Number of populations included	multiple
Level of populations included	national (Brazil, Italy, South Korea)
Geographic areas covered	Asia, Central and South America, Europe
Number of countries covered	multiple countries
Study period	end date in growth phase of wave
**Outcome**	
Raw outcome	epidemiological population-level outcome (cases, deaths)
Time resolution of raw outcome	daily
Computed outcome	none
Method to obtain the computed outcome	– not applicable –
Data source	data from publicly available cross-country selections (JHU)
Data availability	data made available by the authors
**Intervention**	
Terminology for interventions	measures
Terminology for the specific type of non-pharmaceutical interventions	mitigation

– continued on next page –

Exposure types	all interventions together
Types of single interventions	– not applicable –
Coding of interventions	– not applicable –
Source of intervention data	– not applicable –
Availability of data on exposure	– not applicable –
**Methodological approach**	
Empirical approach	parametric
Use of exposure variation	only variation over time for multiple populations
Method	mechanistic model (compartmental single-population transmission model)
Latent variable functionally linked to intervention	transmission rate
Code availability	publicly available
**Effectiveness assessment**	
Reporting of intervention effectiveness	quantification of change in outcome values
Measure of effectiveness	transmission rate
Interpretation of results	implicitly causal
Reporting of uncertainty	yes
Sensitivity analysis	none
Subgroup assessment	none

Riccardo et al. 2020

**Study information**	

– continued on next page –

Author	Riccardo F, Ajelli M, Andrianou XD, Bella A, Manso M, Fabiani M, Bellino S, Boros S, Urdiales AM, Marziano V, Rota MC, Filia A, DAncona F, Siddu A, Punzo O, Trentini F, Guzzetta G, Poletti P, Stefanelli P, Castrucci MR, Ciervo A, Benedetto CD, Tallon M, Piccioli A, Brusaferro S, Rezza G, Merler S, And PP
Title	Epidemiological Characteristics of COVID-19 Cases and Estimates of the Reproductive Numbers 1 Month into the Epidemic, Italy, 28 January to 31 March 2020
Year	2020
Journal	Eurosurveillance
DOI	10.2807/1560-7917.es.2020.25.49.2000790
Date received	2020-05-03
Date accepted	2020-08-14
**Study setting**	
Number of populations included	multiple
Level of populations included	both national and subnational (Italy and its regions)
Geographic areas covered	Europe
Number of countries covered	single country (Italy)
Study period	start and end date span first epidemic wave
**Outcome**	
Raw outcome	epidemiological population-level outcome (cases)
Time resolution of raw outcome	daily
Computed outcome	epidemiological parameter (reproduction number)
Method to obtain the computed outcome	statistical estimation of reproduction number (Cori et al.)
Data source	data from (sub)national authorities
Data availability	data access via source

– continued on next page –

**Intervention**	
Terminology for interventions	measures
Terminology for the specific type of non-pharmaceutical interventions	non-pharmacological
Exposure types	all interventions together (timeline of measures)
Types of single interventions	– not applicable –
Coding of interventions	– not applicable –
Source of intervention data	– not applicable –
Availability of data on exposure	– not applicable –
**Methodological approach**	
Empirical approach	descriptive
Use of exposure variation	only variation over time for multiple populations
Method	description of change over time (description of time course)
Code availability	none
**Effectiveness assessment**	
Reporting of intervention effectiveness	qualitative statement
Measure of effectiveness	reproduction number
Interpretation of results	associative
Reporting of uncertainty	– not applicable –
Sensitivity analysis	none
Subgroup assessment	none

Rieger & Wang 2020	
**Study information**	
Author	Rieger MO, Wang M
Title	Secret Erosion of the "Lockdown"? Patterns in Daily Activities during the SARS-Cov2 Pandemics around the World
Year	2020
Journal	Review of Behavioral Economics
DOI	10.1561/105.00000124
Date received	– could not be evaluated –
Date accepted	2020-08-10 (published)
**Study setting**	
Number of populations included	multiple
Level of populations included	national (Germany, France, UK, US)
Geographic areas covered	Europe, North America
Number of countries covered	multiple countries
Study period	start and end date span first epidemic wave: behavioral
**Outcome**	
Raw outcome	behavioral outcome (mobility: number of trips by mode & time spent in various locations & intention)
Time resolution of raw outcome	daily
Computed outcome	none
Method to obtain the computed outcome	– not applicable –
Data source	mobility data from corporate organizations (Apple: number of trips by mode, Google: time spent in various locations), data from other research projects (study by Fetzer et al.: intention)
Data availability	data access via source

– continued on next page –

**Intervention**	
Terminology for interventions	– not applicable –
Terminology for the specific type of non-pharmaceutical interventions	lockdown
Exposure types	one combination of interventions (lockdown with specific date for each country)
Types of single interventions	– not applicable –
Coding of interventions	not necessary
Source of intervention data	– could not be evaluated –
Availability of data on exposure	raw data documented in the manuscript
**Methodological approach**	
Empirical approach	parametric
Use of exposure variation	only variation over time for multiple populations
Method	non-mechanistic model (correlation)
Code availability	none
**Effectiveness assessment**	
Reporting of intervention effectiveness	quantification of change in outcome values
Measure of effectiveness	mobility: number of trips by mode & time spent in various locations & intention
Interpretation of results	associative
Reporting of uncertainty	yes
Sensitivity analysis	none
Subgroup assessment	none

Ryu et al. 2020	
**Study information**	
Author	Ryu S, Ali ST, Jang C, Kim B, Cowling BJ
Title	Effect of Nonpharmaceutical Interventions on Transmission of Severe Acute Respiratory Syndrome Coronavirus 2, South Korea, 2020
Year	2020
Journal	Emerging Infectious Diseases
DOI	10.3201/eid2610.201886
Date received	– could not be evaluated –
Date accepted	2020-10-15 (published)
**Study setting**	
Number of populations included	single
Level of populations included	national (South Korea)
Geographic areas covered	Asia
Number of countries covered	single country (South Korea)
Study period	start and end date span first epidemic wave
**Outcome**	
Raw outcome	epidemiological individual-level outcome (individual cases and transmission chains)
Time resolution of raw outcome	daily
Computed outcome	epidemiological parameter (reproduction number)
Method to obtain the computed outcome	statistical estimation of reproduction number (Cori et al.)
Data source	data from (sub)national authorities
Data availability	data not accessible
**Intervention**	
Terminology for interventions	interventions

– continued on next page –

Terminology for the specific type of non-pharmaceutical interventions	non-pharmaceutical
Exposure types	one single intervention (emergency declaration (declaration of highest public alert))
Types of single interventions	declaration of a state of emergency
Coding of interventions	not necessary
Source of intervention data	another study (Kim et al.)
Availability of data on exposure	raw data documented in the manuscript
**Methodological approach**	
Empirical approach	descriptive
Use of exposure variation	only variation over time for a single population
Method	description of change over time (comparison of time periods)
Code availability	none
**Effectiveness assessment**	
Reporting of intervention effectiveness	quantification of change in outcome values
Measure of effectiveness	reproduction number
Interpretation of results	associative
Reporting of uncertainty	yes
Sensitivity analysis	none
Subgroup assessment	none

Saez et al. 2020

**Study information**	
Author	Saez M, Tobias A, Varga D, Barceló MA

– continued on next page –

Title	Effectiveness of the Measures to Flatten the Epidemic Curve of COVID-19. The Case of Spain
Year	2020
Journal	Science of The Total Environment
DOI	10.1016/j.scitotenv.2020.138761
Date received	2020-04-06
Date accepted	2020-04-15
**Study setting**	
Number of populations included	single
Level of populations included	national (Spain)
Geographic areas covered	Europe
Number of countries covered	single country (Spain)
Study period	start and end date span first epidemic wave
**Outcome**	
Raw outcome	epidemiological population-level outcome (cases)
Time resolution of raw outcome	daily
Computed outcome	measure of epidemic trend (growth rate)
Method to obtain the computed outcome	simple computation
Data source	data from (sub)national authorities
Data availability	data access via source
**Intervention**	
Terminology for interventions	measures
Terminology for the specific type of non-pharmaceutical interventions	physical distancing

– continued on next page –

Exposure types	one combination of interventions (confinement of the population by decree of the Spanish Government on March 14)
Types of single interventions	– not applicable –
Coding of interventions	not necessary
Source of intervention data	– could not be evaluated –
Availability of data on exposure	raw data documented in the manuscript
**Methodological approach**	
Empirical approach	parametric
Use of exposure variation	only variation over time for a single population
Method	non-mechanistic model (generalized linear model)
Code availability	none
**Effectiveness assessment**	
Reporting of intervention effectiveness	quantification of change in outcome values
Measure of effectiveness	growth rate
Interpretation of results	implicitly causal
Reporting of uncertainty	yes
Sensitivity analysis	none
Subgroup assessment	none

Salje et al. 2020

**Study information**	
Author	Salje H, Kiem CT, Lefrancq N, Courtejoie N, Bosetti P, Paireau J, Andronico A, Hozé N, Richet J, Dubost CL, Strat YL, Lessler J, Levy-Bruhl D, Fontanet A, Opatowski L, Boelle PY, Cauchemez S

– continued on next page –

Title	Estimating the Burden of SARS-CoV-2 in France
Year	2020
Journal	Science
DOI	10.1126/science.abc3517
Date received	2020-04-20
Date accepted	2020-05-11
**Study setting**	
Number of populations included	single
Level of populations included	national (France)
Geographic areas covered	Europe
Number of countries covered	single country (France)
Study period	start and end date span first epidemic wave
**Outcome**	
Raw outcome	epidemiological population-level outcome (hospitalizations, deaths)
Time resolution of raw outcome	daily
Computed outcome	none
Method to obtain the computed outcome	– not applicable –
Data source	data from (sub)national authorities
Data availability	data made available by the authors
**Intervention**	
Terminology for interventions	– not applicable –
Terminology for the specific type of non-pharmaceutical interventions	lockdown
Exposure types	one single intervention (stay-at-home order)
Types of single interventions	stay-at-home order

– continued on next page –

Coding of interventions	not necessary
Source of intervention data	– could not be evaluated –
Availability of data on exposure	raw data documented in the manuscript
**Methodological approach**	
Empirical approach	parametric
Use of exposure variation	only variation over time for a single population
Method	mechanistic model (compartmental single-population transmission model)
Latent variable functionally linked to intervention	transmission rate
Code availability	publicly available
**Effectiveness assessment**	
Reporting of intervention effectiveness	quantification of change in outcome values
Measure of effectiveness	transmission rate, reproduction number
Interpretation of results	implicitly causal
Reporting of uncertainty	yes
Sensitivity analysis	epidemiological parameters varied
Subgroup assessment	none

Salvatore et al. 2020

**Study information**	
Author	Salvatore M, Basu D, Ray D, Kleinsasser M, Purkayastha S, Bhattacharyya R, Mukherjee B

– continued on next page –

Title	Comprehensive Public Health Evaluation of Lockdown as a Non-Pharmaceutical Intervention on COVID-19 Spread in India: National Trends Masking State-Level Variations
Year	2020
Journal	BMJ Open
DOI	10.1136/bmjopen-2020-041778
Date received	2020-06-17
Date accepted	2020-11-11
**Study setting**	
Number of populations included	multiple
Level of populations included	both national and subnational (India and 20 states of India)
Geographic areas covered	Asia
Number of countries covered	single country (India)
Study period	end date in growth phase of wave
**Outcome**	
Raw outcome	epidemiological population-level outcome (cases)
Time resolution of raw outcome	daily
Computed outcome	measure of epidemic trend (doubling time), epidemiological parameter (reproduction number)
Method to obtain the computed outcome	statistical estimation of reproduction number (Cori et al.), exponential growth model (doubling time)
Data source	data from publicly available open-source projects (covid19india.org: cases), data from publicly available cross-country selections (OurWorldInData: cases)
Data availability	data made available by the authors
**Intervention**	
Terminology for interventions	interventions

– continued on next page –

Terminology for the specific type of non-pharmaceutical interventions	non-pharmaceutical
Exposure types	one combination of interventions (Indian National Lockdown)
Types of single interventions	– not applicable –
Coding of interventions	not necessary
Source of intervention data	government or news websites
Availability of data on exposure	raw data documented in the manuscript
**Methodological approach**	
Empirical approach	descriptive
Use of exposure variation	only variation over time for multiple populations
Method	description of change over time (comparison of time periods)
Code availability	publicly available
**Effectiveness assessment**	
Reporting of intervention effectiveness	comparison of outcome values
Measure of effectiveness	reproduction number, doubling time
Interpretation of results	implicitly causal
Reporting of uncertainty	yes
Sensitivity analysis	none
Subgroup assessment	none

Santamaria et al. 2020

**Study information**	
Author	Santamaria C, Sermi F, Spyratos S, Iacus SM, Annunziato A, Tarchi D, Vespe M

– continued on next page –

Title	Measuring the Impact of COVID-19 Confinement Measures on Human Mobility Using Mobile Positioning Data. A European Regional Analysis
Year	2020
Journal	Safety Science
DOI	10.1016/j.ssci.2020.104925
Date received	2020-07-09
Date accepted	2020-07-17
**Study setting**	
Number of populations included	multiple
Level of populations included	national (14 European countries)
Geographic areas covered	Europe
Number of countries covered	multiple countries
Study period	start and end date span first epidemic wave: behavioral
**Outcome**	
Raw outcome	behavioral outcome (mobility: incoming and outgoing travels in a region)
Time resolution of raw outcome	daily
Computed outcome	none
Method to obtain the computed outcome	– not applicable –
Data source	mobility data from corporate organizations (European Mobile Network Operators)
Data availability	data not accessible
**Intervention**	
Terminology for interventions	measures
Terminology for the specific type of non-pharmaceutical interventions	confinement

– continued on next page –

Exposure types	multiple separate interventions (school closure, bans of large gatherings, bans of small gatherings, workplace closure, stay-at-home order, international travel restrictions, internal movement restrictions, public transport closure)
Types of single interventions	school closure, bans of large gatherings, bans of small gatherings, workplace closure, stay-at-home order, international travel restrictions, internal movement restrictions, public transport closure
Coding of interventions	necessary
Source of intervention data	use of externally coded data (OxCGRT)
Availability of data on exposure	access to externally coded data via source
**Methodological approach**	
Empirical approach	parametric
Use of exposure variation	both variation over time and between populations
Method	non-mechanistic model (generalized linear model)
Code availability	none
**Effectiveness assessment**	
Reporting of intervention effectiveness	quantification of change in outcome values
Measure of effectiveness	mobility: mobility index of number of movements
Interpretation of results	associative
Reporting of uncertainty	yes
Sensitivity analysis	none
Subgroup assessment	none

Saurabh et al. 2020	
**Study information**	
Author	Saurabh S, Verma MK, Gautam V, Kumar N, Goel AD, Gupta MK, Bhardwaj P, Misra S
Title	Transmission Dynamics of the COVID-19 Epidemic at the District Level in India: Prospective Observational Study
Year	2020
Journal	JMIR Public Health and Surveillance
DOI	10.2196/22678
Date received	2020-07-21
Date accepted	2020-09-09
**Study setting**	
Number of populations included	single
Level of populations included	subnational (Jodhpur district (India))
Geographic areas covered	Asia
Number of countries covered	single country (India)
Study period	end date in growth phase of wave
**Outcome**	
Raw outcome	epidemiological individual-level outcome (individual cases and transmission chains, cases)
Time resolution of raw outcome	daily
Computed outcome	epidemiological parameter (reproduction number)
Method to obtain the computed outcome	statistical estimation of reproduction number from individual data (Simplified version of Cori et al., Wallinga and Teunis)
Data source	data from publicly available open-source projects (covid19india.org)
Data availability	data access via source
**Intervention**	

– continued on next page –

Terminology for interventions	measures
Terminology for the specific type of non-pharmaceutical interventions	control
Exposure types	all interventions together
Types of single interventions	– not applicable –
Coding of interventions	– not applicable –
Source of intervention data	– not applicable –
Availability of data on exposure	– not applicable –
**Methodological approach**	
Empirical approach	descriptive
Use of exposure variation	only variation over time for a single population
Method	description of change over time (description of time course)
Code availability	none
**Effectiveness assessment**	
Reporting of intervention effectiveness	qualitative statement
Measure of effectiveness	reproduction number
Interpretation of results	implicitly causal
Reporting of uncertainty	– not applicable –
Sensitivity analysis	none
Subgroup assessment	none

Scarabel et al. 2020

Study information

– continued on next page –

Author	Scarabel F, Pellis L, Bragazzi NL, Wu J
Title	Canada Needs to Rapidly Escalate Public Health Interventions for Its COVID-19 Mitigation Strategies
Year	2020
Journal	Infectious Disease Modelling
DOI	10.1016/j.idm.2020.03.004
Date received	2020-03-25
Date accepted	2020-03-30
**Study setting**	
Number of populations included	single
Level of populations included	national (Italy)
Geographic areas covered	Europe
Number of countries covered	single country (Italy)
Study period	end date in growth phase of wave
**Outcome**	
Raw outcome	epidemiological population-level outcome (cases)
Time resolution of raw outcome	daily
Computed outcome	measure of epidemic trend (growth rate)
Method to obtain the computed outcome	exponential growth model
Data source	data from publicly available cross-country selections (ECDC)
Data availability	data access via source
**Intervention**	
Terminology for interventions	interventions
Terminology for the specific type of non-pharmaceutical interventions	public health

– continued on next page –

Exposure types	multiple combinations of interventions (lockdown (movement restrictions), closure of non-essential businesses)
Types of single interventions	– not applicable –
Coding of interventions	not necessary
Source of intervention data	– could not be evaluated –
Availability of data on exposure	raw data documented in the manuscript
**Methodological approach**	
Empirical approach	descriptive
Use of exposure variation	only variation over time for a single population
Method	description of change over time (comparison of time periods)
Code availability	none
**Effectiveness assessment**	
Reporting of intervention effectiveness	comparison of outcome values
Measure of effectiveness	growth rate
Interpretation of results	implicitly causal
Reporting of uncertainty	no
Sensitivity analysis	none
Subgroup assessment	none

Sebastian et al. 2020 (Analysis 1)

**Study information**	
Author	Sebastian S, Paul A, Joby J, Saijan S, Joseph J, Kunjumon J

– continued on next page –

Title	Impact of Lockdown in India: A Case Study on Karnataka with International Model
Year	2020
Journal	Kesmas: National Public Health Journal
DOI	10.21109/kesmas.v15i2.3978
Date received	2020-07-02
Date accepted	2020-07-03
**Study setting**	
Number of populations included	multiple
Level of populations included	both national and subnational (Karnataka and Pubjab (India), Australia, UK)
Geographic areas covered	Asia, Europe, Oceania
Number of countries covered	multiple countries
Study period	end date could not be evaluated
**Outcome**	
Raw outcome	epidemiological population-level outcome (cases, recovered cases, deaths)
Time resolution of raw outcome	daily
Computed outcome	measure of epidemic trend (growth rate, growth rate: recovered, growth rate: deaths, doubling time)
Method to obtain the computed outcome	simple computation (growth rate), exponential growth model (doubling time)
Data source	data from publicly available cross-country selections (WHO)
Data availability	data access via source
**Intervention**	
Terminology for interventions	measures

– continued on next page –

Terminology for the specific type of non-pharmaceutical interventions	social distancing
Exposure types	one combination of interventions (Indian National Lockdown)
Types of single interventions	– not applicable –
Coding of interventions	not necessary
Source of intervention data	– could not be evaluated –
Availability of data on exposure	raw data documented in the manuscript
**Methodological approach**	
Empirical approach	descriptive
Use of exposure variation	only variation over time for multiple populations
Method	description of change over time (comparison of time periods)
Code availability	none
**Effectiveness assessment**	
Reporting of intervention effectiveness	quantification of change in outcome values
Measure of effectiveness	growth rate, growth rate: recovered, growth rate: deaths, doubling time
Interpretation of results	implicitly causal
Reporting of uncertainty	no
Sensitivity analysis	none
Subgroup assessment	none

Sebastian et al. 2020 (Analysis 2)

**Study information**	
Author	Sebastian S, Paul A, Joby J, Saijan S, Joseph J, Kunjumon J

– continued on next page –

Title	Impact of Lockdown in India: A Case Study on Karnataka with International Model
Year	2020
Journal	Kesmas: National Public Health Journal
DOI	10.21109/kesmas.v15i2.3978
Date received	2020-07-02
Date accepted	2020-07-03
**Study setting**	
Number of populations included	multiple
Level of populations included	both national and subnational (Karnataka and Pubjab (India), Australia, UK)
Geographic areas covered	Asia, Europe, Oceania
Number of countries covered	multiple countries
Study period	end date could not be evaluated
**Outcome**	
Raw outcome	epidemiological population-level outcome (cases)
Time resolution of raw outcome	daily
Computed outcome	none
Method to obtain the computed outcome	– not applicable –
Data source	data from publicly available cross-country selections (WHO)
Data availability	data access via source
**Intervention**	
Terminology for interventions	measures
Terminology for the specific type of non-pharmaceutical interventions	social distancing

– continued on next page –

Exposure types	one combination of interventions (Indian National Lockdown)
Types of single interventions	– not applicable –
Coding of interventions	not necessary
Source of intervention data	– could not be evaluated –
Availability of data on exposure	raw data documented in the manuscript
**Methodological approach**	
Empirical approach	counterfactual
Use of exposure variation	only variation over time for multiple populations
Method	non-mechanistic model (exponential growth model)
Code availability	none
**Effectiveness assessment**	
Reporting of intervention effectiveness	quantification of change in outcome values
Measure of effectiveness	cases
Interpretation of results	implicitly causal
Reporting of uncertainty	no
Sensitivity analysis	none
Subgroup assessment	none

Sebastiani et al. 2020

**Study information**	
Author	Sebastiani G, Massa M, Riboli E
Title	COVID-19 Epidemic in Italy: Evolution, Projections and Impact of Government Measures

– continued on next page –

Year	2020
Journal	European Journal of Epidemiology
DOI	10.1007/s10654-020-00631-6
Date received	2020-04-06
Date accepted	2020-04-08
**Study setting**	
Number of populations included	multiple
Level of populations included	both national and subnational (Italy and 107 provinces)
Geographic areas covered	Europe
Number of countries covered	single country (Italy)
Study period	end date at peak of wave
**Outcome**	
Raw outcome	epidemiological population-level outcome (cases)
Time resolution of raw outcome	daily
Computed outcome	none
Method to obtain the computed outcome	– not applicable –
Data source	data from (sub)national authorities
Data availability	data access via source
**Intervention**	
Terminology for interventions	measures
Terminology for the specific type of non-pharmaceutical interventions	government
Exposure types	all interventions together (lockdown (quarantining, movement restrictions, school and venue closure etc.))
Types of single interventions	– not applicable –

– continued on next page –

Coding of interventions	– not applicable –
Source of intervention data	– not applicable –
Availability of data on exposure	– not applicable –
**Methodological approach**	
Empirical approach	counterfactual
Use of exposure variation	only variation over time for multiple populations
Method	non-mechanistic model (exponential growth model)
Code availability	none
**Effectiveness assessment**	
Reporting of intervention effectiveness	qualitative statement
Measure of effectiveness	cases
Interpretation of results	implicitly causal
Reporting of uncertainty	– not applicable –
Sensitivity analysis	none
Subgroup assessment	none

Seemann et al. 2020

**Study information**	
Author	Seemann T, Lane CR, Sherry NL, Duchene S, Gonçalves da Silva A, Caly L, Sait M, Ballard SA, Horan K, Schultz MB, Hoang T, Easton M, Dougall S, Stinear TP, Druce J, Catton M, Sutton B, van Diemen A, Alpren C, Williamson DA, Howden BP
Title	Tracking the COVID-19 Pandemic in Australia Using Genomics
Year	2020

– continued on next page –

Journal	Nature Communications
DOI	10.1038/s41467-020-18314-x
Date received	2020-07-06
Date accepted	2020-08-17
**Study setting**	
Number of populations included	single
Level of populations included	subnational (Victoria (Australia))
Geographic areas covered	Oceania
Number of countries covered	single country (Australia)
Study period	start and end date span first epidemic wave
**Outcome**	
Raw outcome	epidemiological individual-level outcome (genome sequence data)
Time resolution of raw outcome	daily
Computed outcome	epidemiological parameter (reproduction number)
Method to obtain the computed outcome	phylodynamic model
Data source	data from (sub)national authorities
Data availability	data made available by the authors
**Intervention**	
Terminology for interventions	– not applicable –
Terminology for the specific type of non-pharmaceutical interventions	travel restrictions, physical distancing
Exposure types	one combination of interventions (estimated change point around March 27 roughly corresponding to the implementation of social distancing restrictions)
Types of single interventions	– not applicable –

– continued on next page –

Coding of interventions	– not applicable –
Source of intervention data	– not applicable –
Availability of data on exposure	– not applicable –
**Methodological approach**	
Empirical approach	descriptive
Use of exposure variation	only variation over time for a single population
Method	description of change over time (comparison of time periods)
Code availability	none
**Effectiveness assessment**	
Reporting of intervention effectiveness	comparison of outcome values
Measure of effectiveness	reproduction number
Interpretation of results	associative
Reporting of uncertainty	yes
Sensitivity analysis	none
Subgroup assessment	none

Sehra et al. 2020 (Analysis 1)

**Study information**	
Author	Sehra ST, George M, Wiebe DJ, Fundin S, Baker JF
Title	Cell Phone Activity in Categories of Places and Associations with Growth in Cases of COVID-19 in the US
Year	2020

– continued on next page –

Journal	JAMA Internal Medicine
DOI	10.1001/jamainternmed.2020.4288
Date received	– could not be evaluated –
Date accepted	2020-08-31 (published)
**Study setting**	
Number of populations included	multiple
Level of populations included	both national and subnational (US counties)
Geographic areas covered	North America
Number of countries covered	single country (US)
Study period	start and end date span first epidemic wave: behavioral
**Outcome**	
Raw outcome	behavioral outcome (mobility: time spent in various locations)
Time resolution of raw outcome	daily
Computed outcome	none
Method to obtain the computed outcome	– not applicable –
Data source	mobility data from corporate organizations (Google)
Data availability	data access via source
**Intervention**	
Terminology for interventions	measures
Terminology for the specific type of non-pharmaceutical interventions	social distancing
Exposure types	one single intervention (stay-at-home order)
Types of single interventions	stay-at-home order
Coding of interventions	necessary
Source of intervention data	coding done by authors

– continued on next page –

Availability of data on exposure	coded data not available
**Methodological approach**	
Empirical approach	descriptive
Use of exposure variation	only variation over time for multiple populations
Method	description of change over time (comparison of time periods)
Code availability	none
**Effectiveness assessment**	
Reporting of intervention effectiveness	quantification of change in outcome values
Measure of effectiveness	mobility: time spent in various locations
Interpretation of results	associative
Reporting of uncertainty	yes
Sensitivity analysis	none
Subgroup assessment	none

Sehra et al. 2020 (Analysis 2, mobility as exposure)

**Study information**	
Author	Sehra ST, George M, Wiebe DJ, Fundin S, Baker JF
Title	Cell Phone Activity in Categories of Places and Associations with Growth in Cases of COVID-19 in the US
Year	2020
Journal	JAMA Internal Medicine
DOI	10.1001/jamainternmed.2020.4288
Date received	– could not be evaluated –
Date accepted	31.08.2020 (published)

– continued on next page –

**Study setting**	
Number of populations included	multiple
Level of populations included	both national and subnational (US counties)
Geographic areas covered	North America
Number of countries covered	single country (US)
Study period	same end date for several populations with diverse epidemic trajectories
**Outcome**	
Raw outcome	epidemiological population-level outcome (cases)
Time resolution of raw outcome	daily
Computed outcome	measure of epidemic trend (growth rate)
Method to obtain the computed outcome	simple computation
Data source	data from publicly available cross-country selections (JHU)
Data availability	data access via source
**Intervention**	
Terminology for interventions	measures
Terminology for the specific type of non-pharmaceutical interventions	social distancing
Exposure types	mobility (time spent in various locations)
Types of single interventions	– not applicable –
Coding of interventions	– not applicable –
Source of intervention data	mobility data by corporate organizations (Google)
Availability of data on exposure	data access via source
**Methodological approach**	
Empirical approach	parametric
Use of exposure variation	only variation over time for multiple populations

– continued on next page –

**Sehra et al. 2020 (Analysis 2, mobility as exposure)** – continued from previous page	
Method	non-mechanistic model (generalized linear model)
Code availability	none
**Effectiveness assessment**	
Reporting of intervention effectiveness	quantification of change in outcome values
Measure of effectiveness	growth rate
Interpretation of results	associative
Reporting of uncertainty	yes
Sensitivity analysis	model specification varied, epidemiological parameters varied
Subgroup assessment	none

**Sekine et al. 2020**	
**Study information**	
Author	Sekine I, Uojima H, Koyama H, Kamio T, Sato M, Yamamoto T, Fukaguchi K, Fukui H, Yamagami H
Title	Impact of Non-Pharmaceutical Interventions for the COVID-19 Pandemic on Emergency Department Patient Trends in Japan: A Retrospective Analysis
Year	2020
Journal	Acute Medicine & Surgery
DOI	10.1002/ams2.603
Date received	2020-08-11
Date accepted	2020-10-26
**Study setting**	
Number of populations included	single

– continued on next page –

Level of populations included	subnational (Shonan Kamakura General Hospital in Kanagawa, Japan)
Geographic areas covered	Asia
Number of countries covered	single country (Japan)
Study period	start and end date span first epidemic wave
**Outcome**	
Raw outcome	epidemiological population-level outcome (emergency room visits)
Time resolution of raw outcome	daily
Computed outcome	none
Method to obtain the computed outcome	– not applicable –
Data source	data collected by authors (Shonan Kamakura General Hospital in Kanagawa)
Data availability	data access via source
**Intervention**	
Terminology for interventions	interventions
Terminology for the specific type of non-pharmaceutical interventions	non-pharmaceutical
Exposure types	multiple separate interventions (school closure, emergency declaration)
Types of single interventions	school closure, declaration of a state of emergency
Coding of interventions	not necessary
Source of intervention data	– could not be evaluated –
Availability of data on exposure	raw data documented in the manuscript
**Methodological approach**	
Empirical approach	parametric
Use of exposure variation	only variation over time for a single population
Method	non-mechanistic model (generalized linear model)

– continued on next page –

Code availability	none
**Effectiveness assessment**	
Reporting of intervention effectiveness	quantification of change in outcome values
Measure of effectiveness	emergency room visits
Interpretation of results	associative
Reporting of uncertainty	yes
Sensitivity analysis	none
Subgroup assessment	socioeconomic indicators (age, gender)

Sewell & And 2020 (Analysis 1)

**Study information**	
Author	Sewell DK, And AM
Title	Simulation-Free Estimation of an Individual-Based SEIR Model for Evaluating Nonpharmaceutical Interventions with an Application to COVID-19 in the District of Columbia
Year	2020
Journal	PLOS ONE
DOI	10.1371/journal.pone.0241949
Date received	2020-05-28
Date accepted	2020-09-26
**Study setting**	
Number of populations included	single
Level of populations included	subnational (Washingston D.C.)

– continued on next page –

Geographic areas covered	North America
Number of countries covered	single country (US)
Study period	start and end date span first epidemic wave: behavioral
**Outcome**	
Raw outcome	behavioral outcome (mobility: time spent in locations for retail and recreation)
Time resolution of raw outcome	daily
Computed outcome	none
Method to obtain the computed outcome	– not applicable –
Data source	mobility data from corporate organizations (Google)
Data availability	data made available by the authors
**Intervention**	
Terminology for interventions	interventions
Terminology for the specific type of non-pharmaceutical interventions	non-pharmaceutical
Exposure types	one single intervention (emergency declaration)
Types of single interventions	declaration of a state of emergency
Coding of interventions	not necessary
Source of intervention data	use of externally coded data (Boston University COVID-19 US State Policy Database)
Availability of data on exposure	raw data documented in the manuscript
**Methodological approach**	
Empirical approach	descriptive
Use of exposure variation	only variation over time for a single population
Method	description of change over time (description of time course)

– continued on next page –

Code availability	publicly available
**Effectiveness assessment**	
Reporting of intervention effectiveness	qualitative statement
Measure of effectiveness	mobility: time spent in locations for retail and recreation
Interpretation of results	implicitly causal
Reporting of uncertainty	– not applicable –
Sensitivity analysis	none
Subgroup assessment	none

Sewell & And 2020 (Analysis 2)

**Study information**	
Author	Sewell DK, And AM
Title	Simulation-Free Estimation of an Individual-Based SEIR Model for Evaluating Nonpharmaceutical Interventions with an Application to COVID-19 in the District of Columbia
Year	2020
Journal	PLOS ONE
DOI	10.1371/journal.pone.0241949
Date received	2020-05-28
Date accepted	2020-09-26
**Study setting**	
Number of populations included	single
Level of populations included	subnational (Washingston D.C.)
Geographic areas covered	North America

– continued on next page –

Number of countries covered	single country (US)
Study period	start and end date span first epidemic wave
**Outcome**	
Raw outcome	epidemiological population-level outcome (cases)
Time resolution of raw outcome	daily
Computed outcome	none
Method to obtain the computed outcome	– not applicable –
Data source	data from public media outlets (COVID Tracking Project by the Atlantic)
Data availability	data made available by the authors
**Intervention**	
Terminology for interventions	interventions
Terminology for the specific type of non-pharmaceutical interventions	non-pharmaceutical
Exposure types	one single intervention (mask mandate)
Types of single interventions	mask mandate
Coding of interventions	not necessary
Source of intervention data	use of externally coded data (Boston University COVID-19 US State Policy Database)
Availability of data on exposure	raw data documented in the manuscript
**Methodological approach**	
Empirical approach	parametric
Use of exposure variation	only variation over time for a single population
Method	mechanistic model (individual-based transmission model)
Latent variable functionally linked to intervention	probability of transmission upon contact

– continued on next page –

Code availability	publicly available
**Effectiveness assessment**	
Reporting of intervention effectiveness	quantification of change in outcome values
Measure of effectiveness	probability of transmission upon contact
Interpretation of results	implicitly causal
Reporting of uncertainty	no
Sensitivity analysis	none
Subgroup assessment	none

Shanmugam 2020

**Study information**	
Author	Shanmugam R
Title	Restricted Prevalence Rates of COVID-19's Infectivity, Hospitalization, Recovery, Mortality in the USA and Their Implications
Year	2020
Journal	Journal of Healthcare Informatics Research
DOI	10.1007/s41666-020-00078-0
Date received	2020-05-28
Date accepted	2020-09-25
**Study setting**	
Number of populations included	multiple
Level of populations included	both national and subnational (4 regions in the US: eastern, central, mountain, and pacific time zone states)
Geographic areas covered	North America

– continued on next page –

Number of countries covered	single country (US)
Study period	end date could not be evaluated
**Outcome**	
Raw outcome	epidemiological population-level outcome (cases, deaths, hospitalizations, recovered cases)
Time resolution of raw outcome	– not applicable –
Computed outcome	none
Method to obtain the computed outcome	– not applicable –
Data source	public (Kaiser Family Foundation)
Data availability	data access via source
**Intervention**	
Terminology for interventions	measures
Terminology for the specific type of non-pharmaceutical interventions	social distancing
Exposure types	complete set of interventions
Types of single interventions	– not applicable –
Coding of interventions	– not applicable –
Source of intervention data	– not applicable –
Availability of data on exposure	– not applicable –
**Methodological approach**	
Empirical approach	parametric
Use of exposure variation	only variation over time for multiple populations
Method	non-mechanistic model (generalized linear model)
Code availability	none
**Effectiveness assessment**	

– continued on next page –

Reporting of intervention effectiveness	quantification of change in outcome values
Measure of effectiveness	cases, deaths, hospitalizations, recovered
Interpretation of results	implicitly causal
Reporting of uncertainty	no
Sensitivity analysis	none
Subgroup assessment	geographic areas (regions)

**Shi et al. 2020a (Analysis 1)**	
**Study information**	
Author	Shi Q, Hu Y, Peng B, Tang XJ, Wang W, Su K, Luo C, Wu B, Zhang F, Zhang Y, Anderson B, Zhong XN, Qiu JF, Yang CY, Huang AL
Title	Effective Control of SARS-CoV-2 Transmission in Wanzhou, China
Year	2020
Journal	Nature Medicine
DOI	10.1038/s41591-020-01178-5
Date received	2020-06-22
Date accepted	2020-11-13
**Study setting**	
Number of populations included	single
Level of populations included	subnational (Wanzhou (China))
Geographic areas covered	Asia
Number of countries covered	single country (China)

– continued on next page –

Study period	start and end date span first epidemic wave
**Outcome**	
Raw outcome	epidemiological individual-level outcome (individual cases and transmission chains)
Time resolution of raw outcome	daily
Computed outcome	epidemiological parameter (reproduction number)
Method to obtain the computed outcome	statistical estimation of reproduction number from individual data (Lloyd and Smith, between generations)
Data source	data from (sub)national authorities
Data availability	data not accessible
**Intervention**	
Terminology for interventions	measures
Terminology for the specific type of non-pharmaceutical interventions	control
Exposure types	one combination of interventions (Wuhan City Lockdown)
Types of single interventions	– not applicable –
Coding of interventions	not necessary
Source of intervention data	– could not be evaluated –
Availability of data on exposure	raw data documented in the manuscript
**Methodological approach**	
Empirical approach	descriptive
Use of exposure variation	only variation over time for a single population
Method	description of change over time (comparison of time periods)
Code availability	publicly available
**Effectiveness assessment**	

– continued on next page –

Reporting of intervention effectiveness	comparison of outcome values
Measure of effectiveness	reproduction number
Interpretation of results	associative
Reporting of uncertainty	yes
Sensitivity analysis	none
Subgroup assessment	none

Shi et al. 2020a (Analysis 2)

**Study information**	
Author	Shi Q, Hu Y, Peng B, Tang XJ, Wang W, Su K, Luo C, Wu B, Zhang F, Zhang Y, Anderson B, Zhong XN, Qiu JF, Yang CY, Huang AL
Title	Effective Control of SARS-CoV-2 Transmission in Wanzhou, China
Year	2020
Journal	Nature Medicine
DOI	10.1038/s41591-020-01178-5
Date received	2020-06-22
Date accepted	2020-11-13
**Study setting**	
Number of populations included	single
Level of populations included	subnational (Wanzhou (China))
Geographic areas covered	Asia
Number of countries covered	single country (China)
Study period	start and end date span first epidemic wave
**Outcome**	

– continued on next page –

Raw outcome	epidemiological population-level outcome (cases)
Time resolution of raw outcome	daily
Computed outcome	none
Method to obtain the computed outcome	– not applicable –
Data source	data from (sub)national authorities
Data availability	data access via source
**Intervention**	
Terminology for interventions	measures
Terminology for the specific type of non-pharmaceutical interventions	control
Exposure types	one combination of interventions (Wuhan City Lockdown)
Types of single interventions	– not applicable –
Coding of interventions	not necessary
Source of intervention data	– could not be evaluated –
Availability of data on exposure	raw data documented in the manuscript
**Methodological approach**	
Empirical approach	counterfactual
Use of exposure variation	only variation over time for a single population
Method	mechanistic model (individual-based transmission model)
Code availability	publicly available
**Effectiveness assessment**	
Reporting of intervention effectiveness	comparison of outcome values
Measure of effectiveness	cases
Interpretation of results	associative

– continued on next page –

Reporting of uncertainty	no
Sensitivity analysis	none
Subgroup assessment	none

Shi et al. 2020b

**Study information**	
Author	Shi S, Tanaka S, Ueno R, Gilmour S, Tanoue Y, Kawashima T, Nomura S, Eguchi A, Miyata H, Yoneoka D
Title	Travel Restrictions and SARS-CoV-2 Transmission: An Effective Distance Approach to Estimate Impact
Year	2020
Journal	Bulletin of the World Health Organization
DOI	10.2471/blt.20.255679
Date received	2020-03-18
Date accepted	2020-05-12
**Study setting**	
Number of populations included	multiple
Level of populations included	both national and subnational (80 countries around the globe with their airports)
Geographic areas covered	Europe, Middle East and Africa, Asia, Oceania, Central and South America, North America
Number of countries covered	multiple countries
Study period	same end date for several populations with diverse epidemic trajectories
**Outcome**	

– continued on next page –

Raw outcome	epidemiological population-level outcome (dates of first reported case in country), behavioral outcome (mobility: transportation network data)
Time resolution of raw outcome	daily
Computed outcome	none
Method to obtain the computed outcome	– not applicable –
Data source	public (Automatic Dependent Surveillance-Broadcast exchange data: mobility), – could not be evaluated – (first case date)
Data availability	data access via source
**Intervention**	
Terminology for interventions	– not applicable –
Terminology for the specific type of non-pharmaceutical interventions	travel restrictions
Exposure types	one single intervention (travel restrictions (day of introduction of travel restrictions: entry bans, visa restrictions, flight suspensions))
Types of single interventions	international travel restrictions
Coding of interventions	necessary
Source of intervention data	use of externally coded data (WHO, Think Global Health)
Availability of data on exposure	coded data made available by the authors
**Methodological approach**	
Empirical approach	counterfactual
Use of exposure variation	both variation over time and between populations
Method	other (hazard-based survival model for countries' risk of case importation)
Code availability	publicly available
**Effectiveness assessment**	
Reporting of intervention effectiveness	quantification of change in outcome values

– continued on next page –

Measure of effectiveness	countries' risk of case importation
Interpretation of results	associative
Reporting of uncertainty	no
Sensitivity analysis	none
Subgroup assessment	none

Shim et al. 2021

**Study information**	
Author	Shim E, Tariq A, Chowell G
Title	Spatial Variability in Reproduction Number and Doubling Time across Two Waves of the COVID-19 Pandemic in South Korea, February to July, 2020
Year	2021
Journal	International Journal of Infectious Diseases
DOI	10.1016/j.ijid.2020.10.007
Date received	2020-08-30
Date accepted	2020-10-03
**Study setting**	
Number of populations included	multiple
Level of populations included	subnational (Seoul, Gyeonggi Province, Gyeongbuk Province, and Daegu (South Korea))
Geographic areas covered	Asia
Number of countries covered	single country (South Korea)
Study period	includes second wave (beginning of second wave for some regions)
**Outcome**	

– continued on next page –

Raw outcome	epidemiological population-level outcome (cases)
Time resolution of raw outcome	daily
Computed outcome	measure of epidemic trend (doubling time), epidemiological parameter (reproduction number)
Method to obtain the computed outcome	statistical estimation of reproduction number, simple computation (doubling time)
Data source	data from (sub)national authorities
Data availability	data access via source
**Intervention**	
Terminology for interventions	measures
Terminology for the specific type of non-pharmaceutical interventions	control
Exposure types	all interventions together
Types of single interventions	– not applicable –
Coding of interventions	– not applicable –
Source of intervention data	– not applicable –
Availability of data on exposure	– not applicable –
**Methodological approach**	
Empirical approach	descriptive
Use of exposure variation	only variation over time for multiple populations
Method	description of change over time (description of time course)
Code availability	none
**Effectiveness assessment**	
Reporting of intervention effectiveness	qualitative statement
Measure of effectiveness	reproduction number, doubling time

– continued on next page –

Interpretation of results	implicitly causal
Reporting of uncertainty	– not applicable –
Sensitivity analysis	none
Subgroup assessment	none

Siedner et al. 2020

**Study information**	
Author	Siedner MJ, Harling G, Reynolds Z, Gilbert RF, Haneuse S, Venkataramani AS, Tsai AC
Title	Social Distancing to Slow the US COVID-19 Epidemic: Longitudinal Pretest–Posttest Comparison Group Study
Year	2020
Journal	PLOS Medicine
DOI	10.1371/journal.pmed.1003244
Date received	2020-04-27
Date accepted	2020-07-02
**Study setting**	
Number of populations included	multiple
Level of populations included	both national and subnational (US states)
Geographic areas covered	North America
Number of countries covered	single country (US)
Study period	same end date for several populations with diverse epidemic trajectories
**Outcome**	
Raw outcome	epidemiological population-level outcome (cases, deaths)

– continued on next page –

Time resolution of raw outcome	daily
Computed outcome	measure of epidemic trend (growth rate, growth rate: deaths)
Method to obtain the computed outcome	simple computation
Data source	data from public media outlets (New York Times)
Data availability	data access via source
**Intervention**	
Terminology for interventions	measures
Terminology for the specific type of non-pharmaceutical interventions	social distancing
Exposure types	start of interventions (days since first social distancing measure (closure of schools, closure of workplaces, cancellations of public events, stay-at-home order, closure of state borders))
Types of single interventions	– not applicable –
Coding of interventions	necessary
Source of intervention data	coding done by authors
Availability of data on exposure	coded data made available by the authors
**Methodological approach**	
Empirical approach	parametric
Use of exposure variation	both variation over time and between populations
Method	non-mechanistic model (generalized linear model)
Code availability	none
**Effectiveness assessment**	
Reporting of intervention effectiveness	quantification of change in outcome values
Measure of effectiveness	growth rate, growth rate: deaths

– continued on next page –

Interpretation of results	associative
Reporting of uncertainty	yes
Sensitivity analysis	model specification varied, epidemiological parameters varied
Subgroup assessment	none

Signorelli et al. 2020

**Study information**	
Author	Signorelli C, Scognamiglio T, Odone A
Title	COVID-19 in Italy: Impact of Containment Measures and Prevalence Estimates of Infection in the General Population
Year	2020
Journal	Acta Bio Medica Atenei Parmensis
DOI	10.23750/abm.v91i3-S.9511
Date received	2020-04-07
Date accepted	2020-04-08
**Study setting**	
Number of populations included	multiple
Level of populations included	both national and subnational (Italy and regions)
Geographic areas covered	Europe
Number of countries covered	single country (Italy)
Study period	start and end date span first epidemic wave
**Outcome**	
Raw outcome	epidemiological population-level outcome (cases)
Time resolution of raw outcome	daily

– continued on next page –

Computed outcome	none
Method to obtain the computed outcome	– not applicable –
Data source	data from (sub)national authorities
Data availability	data access via source
**Intervention**	
Terminology for interventions	measures
Terminology for the specific type of non-pharmaceutical interventions	containment
Exposure types	all interventions together (timeline of measures)
Types of single interventions	– not applicable –
Coding of interventions	– not applicable –
Source of intervention data	– not applicable –
Availability of data on exposure	– not applicable –
**Methodological approach**	
Empirical approach	descriptive
Use of exposure variation	only variation over time for multiple populations
Method	description of change over time (description of time course)
Code availability	none
**Effectiveness assessment**	
Reporting of intervention effectiveness	qualitative statement
Measure of effectiveness	cases
Interpretation of results	implicitly causal
Reporting of uncertainty	– not applicable –

– continued on next page –

Sensitivity analysis	none
Subgroup assessment	none

**Silva et al. 2020**	
**Study information**	
Author	Silva L, Filho DF, Fernandes A
Title	The Effect of Lockdown on the COVID-19 Epidemic in Brazil: Evidence from an Interrupted Time Series Design
Year	2020
Journal	Cadernos de Saúde Pública
DOI	10.1590/0102-311x00213920
Date received	2020-07-21
Date accepted	2020-08-31
**Study setting**	
Number of populations included	multiple
Level of populations included	subnational (4 cities (Brazil))
Geographic areas covered	Central and South America
Number of countries covered	single country (Brazil)
Study period	end date in growth phase of wave
**Outcome**	
Raw outcome	epidemiological population-level outcome (cases, deaths)
Time resolution of raw outcome	daily
Computed outcome	none

– continued on next page –

Method to obtain the computed outcome	– not applicable –
Data source	data from (sub)national authorities
Data availability	data made available by the authors
**Intervention**	
Terminology for interventions	– not applicable –
Terminology for the specific type of non-pharmaceutical interventions	lockdown
Exposure types	one combination of interventions (lockdown)
Types of single interventions	– not applicable –
Coding of interventions	not necessary
Source of intervention data	– could not be evaluated –
Availability of data on exposure	raw data documented in the manuscript
**Methodological approach**	
Empirical approach	parametric
Use of exposure variation	only variation over time for multiple populations
Method	non-mechanistic model (generalized linear model)
Code availability	publicly available
**Effectiveness assessment**	
Reporting of intervention effectiveness	quantification of change in outcome values
Measure of effectiveness	cases, deaths
Interpretation of results	implicitly causal
Reporting of uncertainty	yes
Sensitivity analysis	none
Subgroup assessment	none

Singh & Singh 2020	
**Study information**	
Author	Singh BP, Singh G
Title	Modeling Tempo ofCOVID-19 Pandemic in India and Significance of Lock-down
Year	2020
Journal	Journal of Public Affairs
DOI	10.1002/pa.2257
Date received	2020-06-24
Date accepted	2020-06-27
**Study setting**	
Number of populations included	multiple
Level of populations included	both national and subnational (India and its states)
Geographic areas covered	Asia
Number of countries covered	single country (India)
Study period	end date in growth phase of wave
**Outcome**	
Raw outcome	epidemiological population-level outcome (cases)
Time resolution of raw outcome	daily
Computed outcome	measure of epidemic trend (growth rate)
Method to obtain the computed outcome	exponential growth model
Data source	data from publicly available open-source projects (covid19india.org)
Data availability	data access via source
**Intervention**	

– continued on next page –

Terminology for interventions	strategies
Terminology for the specific type of non-pharmaceutical interventions	physical distancing
Exposure types	one combination of interventions (Indian National Lockdown)
Types of single interventions	– not applicable –
Coding of interventions	not necessary
Source of intervention data	– could not be evaluated –
Availability of data on exposure	raw data documented in the manuscript
**Methodological approach**	
Empirical approach	descriptive
Use of exposure variation	only variation over time for a single population
Method	description of change over time (comparison of time periods)
Code availability	none
**Effectiveness assessment**	
Reporting of intervention effectiveness	quantification of change in outcome values
Measure of effectiveness	growth rate
Interpretation of results	implicitly causal
Reporting of uncertainty	yes
Sensitivity analysis	none
Subgroup assessment	none

Singh et al. 2020

Study information

– continued on next page –

Author	Singh BB, Lowerison M, Lewinson RT, Vallerand IA, Deardon R, Gill JP, Singh B, Barkema HW
Title	Public Health Interventions Slowed but Did Not Halt the Spread of COVID-19 in India
Year	2020
Journal	Transboundary and Emerging Diseases
DOI	10.1111/tbed.13868
Date received	2020-06-20
Date accepted	2020-09-26
**Study setting**	
Number of populations included	multiple
Level of populations included	both national and subnational (India and 12 states)
Geographic areas covered	Asia
Number of countries covered	single country (India)
Study period	end date in growth phase of wave
**Outcome**	
Raw outcome	epidemiological population-level outcome (cases), behavioral outcome (mobility: time spent in various locations)
Time resolution of raw outcome	daily
Computed outcome	epidemiological parameter (reproduction number)
Method to obtain the computed outcome	statistical estimation of reproduction number (Cori et al.)
Data source	data from publicly available cross-country selections (WHO: cases), mobility data from corporate organizations (Google: mobility)
Data availability	data access via source
**Intervention**	

– continued on next page –

Terminology for interventions	interventions
Terminology for the specific type of non-pharmaceutical interventions	public health
Exposure types	one combination of interventions (Indian National Lockdown)
Types of single interventions	– not applicable –
Coding of interventions	not necessary
Source of intervention data	government or news websites
Availability of data on exposure	raw data documented in the manuscript
**Methodological approach**	
Empirical approach	descriptive
Use of exposure variation	only variation over time for multiple populations
Method	description of change over time (comparison of time periods)
Code availability	none
**Effectiveness assessment**	
Reporting of intervention effectiveness	comparison of outcome values
Measure of effectiveness	cases, reproduction number, mobility: time spent in various locations
Interpretation of results	implicitly causal
Reporting of uncertainty	yes
Sensitivity analysis	none
Subgroup assessment	none

Sinha 2020

Study information

– continued on next page –

Author	Sinha S
Title	Epidemiological Dynamics of the COVID-19 Pandemic in India: An Interim Assessment
Year	2020
Journal	Statistics and Applications
DOI	– not applicable –
Date received	2020-07-20
Date accepted	2020-07-30
**Study setting**	
Number of populations included	single
Level of populations included	national (India)
Geographic areas covered	Asia
Number of countries covered	single country (India)
Study period	end date in growth phase of wave
**Outcome**	
Raw outcome	epidemiological population-level outcome (cases, deaths, recovered cases)
Time resolution of raw outcome	daily
Computed outcome	epidemiological parameter (reproduction number)
Method to obtain the computed outcome	exponential growth model
Data source	data from publicly available cross-country selections (JHU)
Data availability	data access via source
**Intervention**	
Terminology for interventions	interventions
Terminology for the specific type of non-pharmaceutical interventions	non-pharmaceutical

– continued on next page –

Exposure types	one combination of interventions (Indian National Lockdown)
Types of single interventions	– not applicable –
Coding of interventions	not necessary
Source of intervention data	– could not be evaluated –
Availability of data on exposure	raw data documented in the manuscript
**Methodological approach**	
Empirical approach	descriptive
Use of exposure variation	only variation over time for a single population
Method	description of change over time (comparison of time periods)
Code availability	none
**Effectiveness assessment**	
Reporting of intervention effectiveness	comparison of outcome values
Measure of effectiveness	reproduction number
Interpretation of results	associative
Reporting of uncertainty	yes
Sensitivity analysis	none
Subgroup assessment	none

Spaccaferri et al. 2020

**Study information**	
Author	Spaccaferri G, Larrieu S, Pouey J, Calba C, Benet T, Sommen C, Lévy-Bruhl D, Smaili S, Che D, Filleul L, Caserio-Schönemann C, Ait-El-Belghiti F, Haeghebaert S, Desenclos JC, Huiart L, Laporte A, Rolland P

– continued on next page –

Title	Early Assessment of the Impact of Mitigation Measures to Control COVID-19 in 22 French Metropolitan Areas, October to November 2020
Year	2020
Journal	Eurosurveillance
DOI	10.2807/1560-7917.es.2020.25.50.2001974
Date received	2020-11-21
Date accepted	2020-12-17
**Study setting**	
Number of populations included	multiple
Level of populations included	both national and subnational (22 French metropolitan areas)
Geographic areas covered	Europe
Number of countries covered	single country (France)
Study period	includes second wave (focus on second wave: start date October 28, 2020, end date November 15, 2020)
**Outcome**	
Raw outcome	epidemiological population-level outcome (cases, hospitalizations, test positivity rate)
Time resolution of raw outcome	daily
Computed outcome	measure of epidemic trend (growth rate, growth rate: hospitalizations, growth rate: test positivity rate)
Method to obtain the computed outcome	simple computation
Data source	data from (sub)national authorities
Data availability	data access via source
**Intervention**	
Terminology for interventions	measures

– continued on next page –

Terminology for the specific type of non-pharmaceutical interventions	mitigation
Exposure types	one combination of interventions (Wuhan City Lockdown)
Types of single interventions	– not applicable –
Coding of interventions	not necessary
Source of intervention data	– could not be evaluated –
Availability of data on exposure	raw data documented in the manuscript
**Methodological approach**	
Empirical approach	descriptive
Use of exposure variation	only variation over time for multiple populations
Method	description of change over time (comparison of time periods)
Code availability	none
**Effectiveness assessment**	
Reporting of intervention effectiveness	comparison of outcome values
Measure of effectiveness	growth rate, growth rate: hospitalizations, growth rate: test positivity rate
Interpretation of results	associative
Reporting of uncertainty	no
Sensitivity analysis	none
Subgroup assessment	public health response (response time)

Sun et al. 2020

Study information

– continued on next page –

Author	Sun S, Folarin AA, Ranjan Y, Rashid Z, Conde P, Stewart C, Cummins N, Matcham F, Dalla Costa G, Simblett S, Leocani L, Lamers F, Sørensen PS, Buron M, Zabalza A, Guerrero Pérez AI, Penninx BW, Siddi S, Haro JM, Myin-Germeys I, Rintala A, Wykes T, Narayan VA, Comi G, Hotopf M, Dobson RJ, RADAR-CNS Consortium
Title	Using Smartphones and Wearable Devices to Monitor Behavioral Changes During COVID-19
Year	2020
Journal	Journal of Medical Internet Research
DOI	10.2196/19992
Date received	2020-05-08
Date accepted	2020-07-26
**Study setting**	
Number of populations included	multiple
Level of populations included	national (Italy, Spain, Denmark, UK, Netherlands)
Geographic areas covered	Europe
Number of countries covered	multiple countries
Study period	start and end date span first epidemic wave: behavioral
**Outcome**	
Raw outcome	behavioral outcome (mobility: time spent at home, maximum distance from place of residence, steps, maximum number of nearby devices)
Time resolution of raw outcome	daily
Computed outcome	none
Method to obtain the computed outcome	– not applicable –
Data source	data collected by authors (RADAR CNS study using wearable devices and smartphones)

– continued on next page –

Data availability	data not accessible
**Intervention**	
Terminology for interventions	interventions
Terminology for the specific type of non-pharmaceutical interventions	non-pharmaceutical
Exposure types	start of interventions (date of first restrictive measure introducing national lockdown)
Types of single interventions	– not applicable –
Coding of interventions	not necessary
Source of intervention data	use of externally coded data (OxCGRT)
Availability of data on exposure	access to externally coded data via source
**Methodological approach**	
Empirical approach	descriptive
Use of exposure variation	only variation over time for multiple populations
Method	description of change over time (comparison of time periods)
Code availability	none
**Effectiveness assessment**	
Reporting of intervention effectiveness	quantification of change in outcome values
Measure of effectiveness	mobility: time spent at home & maximum distance from place of residence & steps & maximum number of nearby devices
Interpretation of results	associative
Reporting of uncertainty	yes
Sensitivity analysis	none
Subgroup assessment	none

Supino et al. 2020	
**Study information**	
Author	Supino M, d'Onofrio A, Luongo F, Occhipinti G, Co AD
Title	The Effects of Containment Measures in the Italian Outbreak of COVID-19
Year	2020
Journal	BMC Public Health
DOI	10.1186/s12889-020-09913-w
Date received	2020-04-03
Date accepted	2020-11-18
**Study setting**	
Number of populations included	multiple
Level of populations included	both national and subnational (Italy (excluding regions with saturated ICU capacity), Lombardy)
Geographic areas covered	Europe
Number of countries covered	single country (Italy)
Study period	start and end date span first epidemic wave
**Outcome**	
Raw outcome	epidemiological population-level outcome (ICU hospitalizations, deaths)
Time resolution of raw outcome	daily
Computed outcome	none
Method to obtain the computed outcome	– not applicable –
Data source	data from (sub)national authorities
Data availability	data access via source
**Intervention**	
Terminology for interventions	measures

– continued on next page –

Terminology for the specific type of non-pharmaceutical interventions	containment
Exposure types	multiple combinations of interventions (lockdown on March 9, full lockdown on March 21 involving work ban)
Types of single interventions	– not applicable –
Coding of interventions	not necessary
Source of intervention data	– could not be evaluated –
Availability of data on exposure	raw data documented in the manuscript
**Methodological approach**	
Empirical approach	counterfactual
Use of exposure variation	only variation over time for a single population
Method	non-mechanistic model (exponential growth model)
Code availability	none
**Effectiveness assessment**	
Reporting of intervention effectiveness	qualitative statement
Measure of effectiveness	ICU hospitalizations, deaths
Interpretation of results	implicitly causal
Reporting of uncertainty	– not applicable –
Sensitivity analysis	none
Subgroup assessment	none

Suraya et al. 2020 (Analysis 1)	
**Study information**	
Author	Suraya I, Nurmansyah MI, Rachmawati E, Aufa BA, Koire II
Title	The Impact of Large-Scale Social Restriction on COVID-19 Incidence: A Case Study of Four Regions in Indonesia
Year	2020
Journal	Kesmas: National Public Health Journal
DOI	10.21109/kesmas.v15i2.3990
Date received	2020-06-19
Date accepted	2020-06-20
**Study setting**	
Number of populations included	multiple
Level of populations included	subnational (4 provinces in Indonesia)
Geographic areas covered	Asia
Number of countries covered	single country (Indonesia)
Study period	end date in growth phase of wave
**Outcome**	
Raw outcome	epidemiological population-level outcome (cases)
Time resolution of raw outcome	daily
Computed outcome	none
Method to obtain the computed outcome	– not applicable –
Data source	– could not be evaluated –
Data availability	data not accessible
**Intervention**	
Terminology for interventions	– not applicable –

– continued on next page –

Terminology for the specific type of non-pharmaceutical interventions	social restrictions
Exposure types	one combination of interventions (large scale social restrictions (school closure, non-essential business closures, restrictions of religious activities, limitations on activities in public places or facilities, limitations on mode of transportations, restrictions on activities related to defense and security))
Types of single interventions	– not applicable –
Coding of interventions	not necessary
Source of intervention data	– could not be evaluated –
Availability of data on exposure	raw data documented in the manuscript
**Methodological approach**	
Empirical approach	descriptive
Use of exposure variation	only variation over time for multiple populations
Method	description of change over time (comparison of time periods)
Code availability	none
**Effectiveness assessment**	
Reporting of intervention effectiveness	comparison of outcome values
Measure of effectiveness	cases
Interpretation of results	implicitly causal
Reporting of uncertainty	yes
Sensitivity analysis	none
Subgroup assessment	none

Suraya et al. 2020 (Analysis 2)	
**Study information**	
Author	Suraya I, Nurmansyah MI, Rachmawati E, Aufa BA, Koire II
Title	The Impact of Large-Scale Social Restriction on COVID-19 Incidence: A Case Study of Four Regions in Indonesia
Year	2020
Journal	Kesmas: National Public Health Journal
DOI	10.21109/kesmas.v15i2.3990
Date received	2020-06-19
Date accepted	2020-06-20
**Study setting**	
Number of populations included	multiple
Level of populations included	subnational (4 provinces in Indonesia)
Geographic areas covered	Asia
Number of countries covered	single country (Indonesia)
Study period	end date in growth phase of wave
**Outcome**	
Raw outcome	epidemiological population-level outcome (cases)
Time resolution of raw outcome	daily
Computed outcome	none
Method to obtain the computed outcome	– not applicable –
Data source	– could not be evaluated –
Data availability	data not accessible
**Intervention**	
Terminology for interventions	– not applicable –

– continued on next page –

Terminology for the specific type of non-pharmaceutical interventions	social restrictions
Exposure types	one combination of interventions (large scale social restrictions (school closure, non-essential business closures, restrictions of religious activities, limitations on activities in public places or facilities, limitations on mode of transportations, restrictions on activities related to defense and security))
Types of single interventions	– not applicable –
Coding of interventions	not necessary
Source of intervention data	– could not be evaluated –
Availability of data on exposure	raw data documented in the manuscript
**Methodological approach**	
Empirical approach	parametric
Use of exposure variation	only variation over time for multiple populations
Method	non-mechanistic model (generalized linear model)
Code availability	none
**Effectiveness assessment**	
Reporting of intervention effectiveness	quantification of change in outcome values
Measure of effectiveness	incidence rate ratio
Interpretation of results	implicitly causal
Reporting of uncertainty	yes
Sensitivity analysis	none
Subgroup assessment	none

Talmoudi et al. 2020	
**Study information**	
Author	Talmoudi K, Safer M, Letaief H, Hchaichi A, Harizi C, Dhaouadi S, Derouiche S, Bouaziz I, Gharbi D, Najar N, Osman M, Cherif I, Mlallekh R, Ben-Ayed O, Ayedi Y, Bouabid L, Bougatef S, ép Ben-Alaya NB, Chahed MK
Title	Estimating Transmission Dynamics and Serial Interval of the First Wave of COVID-19 Infections under Different Control Measures: A Statistical Analysis in Tunisia from February 29 to May 5, 2020
Year	2020
Journal	BMC Infectious Diseases
DOI	10.1186/s12879-020-05577-4
Date received	2020-05-23
Date accepted	2020-11-03
**Study setting**	
Number of populations included	single
Level of populations included	national (Tunisia)
Geographic areas covered	Middle East and Africa
Number of countries covered	single country (Tunisia)
Study period	start and end date span first epidemic wave
**Outcome**	
Raw outcome	epidemiological individual-level outcome (cases, individual cases)
Time resolution of raw outcome	daily
Computed outcome	epidemiological parameter (reproduction number)
Method to obtain the computed outcome	statistical estimation of reproduction number (Cori et al.)

– continued on next page –

Data source	data from (sub)national authorities (National Notifiable Diseases Reporting System: influenza, National Sentinel Influenza Surveillance Network: influenza-like illnesses)
Data availability	data access via source
**Intervention**	
Terminology for interventions	measures
Terminology for the specific type of non-pharmaceutical interventions	control
Exposure types	multiple combinations of interventions (curfew, lockdown)
Types of single interventions	– not applicable –
Coding of interventions	not necessary
Source of intervention data	– could not be evaluated –
Availability of data on exposure	raw data documented in the manuscript
**Methodological approach**	
Empirical approach	descriptive
Use of exposure variation	only variation over time for a single population
Method	description of change over time (comparison of time periods)
Code availability	none
**Effectiveness assessment**	
Reporting of intervention effectiveness	comparison of outcome values
Measure of effectiveness	reproduction number
Interpretation of results	implicitly causal
Reporting of uncertainty	yes
Sensitivity analysis	none
Subgroup assessment	none

Tamagusko & Ferreira 2020 (Analysis 1)	
**Study information**	
Author	Tamagusko T, Ferreira A
Title	Data-Driven Approach to Understand the Mobility Patterns of the Portuguese Population during the COVID-19 Pandemic
Year	2020
Journal	Sustainability
DOI	10.3390/su12229775
Date received	2020-10-14
Date accepted	2020-11-20
**Study setting**	
Number of populations included	single
Level of populations included	national (Portugal)
Geographic areas covered	Europe
Number of countries covered	single country (Portugal)
Study period	start and end date span first epidemic wave: behavioral
**Outcome**	
Raw outcome	behavioral outcome (mobility: time spent in various locations)
Time resolution of raw outcome	daily
Computed outcome	change points (change points: mobility)
Method to obtain the computed outcome	change point analysis
Data source	mobility data from corporate organizations (Google)
Data availability	data made available by the authors

– continued on next page –

**Intervention**	
Terminology for interventions	measures, policies
Terminology for the specific type of non-pharmaceutical interventions	social distancing
Exposure types	all interventions together (timeline of measures)
Types of single interventions	– not applicable –
Coding of interventions	– not applicable –
Source of intervention data	– not applicable –
Availability of data on exposure	– not applicable –
**Methodological approach**	
Empirical approach	descriptive
Use of exposure variation	only variation over time for a single population
Method	comparison of change points with intervention
Code availability	publicly available
**Effectiveness assessment**	
Reporting of intervention effectiveness	comparison of outcome values
Measure of effectiveness	change points: mobility
Interpretation of results	associative
Reporting of uncertainty	no
Sensitivity analysis	none
Subgroup assessment	none

Tamagusko & Ferreira 2020 (Analysis 2)	
**Study information**	
Author	Tamagusko T, Ferreira A
Title	Data-Driven Approach to Understand the Mobility Patterns of the Portuguese Population during the COVID-19 Pandemic
Year	2020
Journal	Sustainability
DOI	10.3390/su12229775
Date received	2020-10-14
Date accepted	2020-11-20
**Study setting**	
Number of populations included	single
Level of populations included	national (Portugal)
Geographic areas covered	Europe
Number of countries covered	single country (Portugal)
Study period	start and end date span first epidemic wave
**Outcome**	
Raw outcome	epidemiological population-level outcome (cases)
Time resolution of raw outcome	daily
Computed outcome	epidemiological parameter (reproduction number)
Method to obtain the computed outcome	statistical estimation of reproduction number (Cori et al.)
Data source	data from publicly available cross-country selections (OurWorldInData)
Data availability	data made available by the authors
**Intervention**	
Terminology for interventions	measures, policies

– continued on next page –

Terminology for the specific type of non-pharmaceutical interventions	social distancing
Exposure types	all interventions together (timeline of measures)
Types of single interventions	– not applicable –
Coding of interventions	– not applicable –
Source of intervention data	– not applicable –
Availability of data on exposure	– not applicable –
**Methodological approach**	
Empirical approach	descriptive
Use of exposure variation	only variation over time for a single population
Method	description of change over time (description of time course)
Code availability	publicly available
**Effectiveness assessment**	
Reporting of intervention effectiveness	qualitative statement
Measure of effectiveness	reproduction number
Interpretation of results	associative
Reporting of uncertainty	– not applicable –
Sensitivity analysis	none
Subgroup assessment	none

**Tchole et al. 2020**	
**Study information**	
Author	Tchole AI, Li ZW, Wei JT, Ye RZ, Wang WJ, Du WY, Wang HT, Yin CN, Ji XK, Xue FZ, Bachir AM, Zhao L, Cao WC

– continued on next page –

Title	Epidemic and Control of COVID-19 in Niger: Quantitative Analyses in a Least Developed Country
Year	2020
Journal	Journal of Global Health
DOI	10.7189/jogh.10.020513
Date received	– could not be evaluated –
Date accepted	2020-12-06
**Study setting**	
Number of populations included	single
Level of populations included	national (Niger)
Geographic areas covered	Middle East and Africa
Number of countries covered	single country (Niger)
Study period	start and end date span first epidemic wave
**Outcome**	
Raw outcome	epidemiological individual-level outcome (individual cases)
Time resolution of raw outcome	daily
Computed outcome	epidemiological parameter (reproduction number)
Method to obtain the computed outcome	statistical estimation of reproduction number (Cori et al.)
Data source	data from (sub)national authorities
Data availability	data not accessible
**Intervention**	
Terminology for interventions	measures
Terminology for the specific type of non-pharmaceutical interventions	public health
Exposure types	all interventions together (timeline of measures)

– continued on next page –

Types of single interventions	– not applicable –
Coding of interventions	– not applicable –
Source of intervention data	– not applicable –
Availability of data on exposure	– not applicable –
**Methodological approach**	
Empirical approach	descriptive
Use of exposure variation	only variation over time for a single population
Method	description of change over time (description of time course)
Code availability	none
**Effectiveness assessment**	
Reporting of intervention effectiveness	qualitative statement
Measure of effectiveness	reproduction number
Interpretation of results	associative
Reporting of uncertainty	– not applicable –
Sensitivity analysis	none
Subgroup assessment	none

Thai et al. 2020

Study information

– continued on next page –

Author	Thai PQ, Rabaa MA, Luong DH, Tan DQ, Quang TD, Quach HL, Thi NA, Dinh PC, Nghia ND, Tu TA, Quang N, Phuc TM, Chau V, Khanh NC, Anh DD, Duong TN, Thwaites G, van Doorn HR, Choisy M, Chambers M, Choisy M, Day J, Trinh DH, Tam DT, Donovan J, Duc DH, Geskus RB, Chanh HQ, Van HH, Thao HD, Huy Hle, Ha HN, Trieu HT, Yen HX, Kestelyn E, Kesteman T, Nguyet LA, Yen LM, Lawson K, Thanh LK, Nhu LN, Nhat LT, Lan LT, Van TL, Lewycka SO, Tran NB, Nguyet NM, Quyen NT, Ngoc NT, Ny NT, Thuong NT, Trang NT, Tuyen NT, Diep NT, Dung NT, Tam NT, Hong NT, Trang NT, Van VC, Truong NX, Van NT, Khanh PN, Lam PK, Yen PL, Nhat PT, Rabaa M, Thuong TN, Thwaites G, Thwaites L, Phuc TM, Thanh TT, Ngoc TT, Hien TT, Van DH, Van NJ, Chau V, Bich VT, Hang VT, And SY
Title	The First 100 Days of Severe Acute Respiratory Syndrome Coronavirus 2 (SARS-CoV-2) Control in Vietnam
Year	2020
Journal	Clinical Infectious Diseases
DOI	10.1093/cid/ciaa1130
Date received	2020-06-02
Date accepted	2020-07-28
**Study setting**	
Number of populations included	single
Level of populations included	national (Vietnam)
Geographic areas covered	Asia
Number of countries covered	single country (Vietnam)
Study period	start and end date span first epidemic wave
**Outcome**	
Raw outcome	epidemiological population-level outcome (cases), behavioral outcome (mobility: number of trips by mode and time spent in various locations)
Time resolution of raw outcome	daily

– continued on next page –

Computed outcome	epidemiological parameter (reproduction number)
Method to obtain the computed outcome	statistical estimation of reproduction number (Cori et al.)
Data source	data from (sub)national authorities (cases), mobility data from corporate organizations (Apple: number of trips by mode, Google: time spent in various locations)
Data availability	data access via source
**Intervention**	
Terminology for interventions	measures
Terminology for the specific type of non-pharmaceutical interventions	control
Exposure types	all interventions together (timeline of measures)
Types of single interventions	– not applicable –
Coding of interventions	– not applicable –
Source of intervention data	– not applicable –
Availability of data on exposure	– not applicable –
**Methodological approach**	
Empirical approach	descriptive
Use of exposure variation	only variation over time for a single population
Method	description of change over time (description of time course)
Code availability	none
**Effectiveness assessment**	
Reporting of intervention effectiveness	qualitative statement
Measure of effectiveness	cases, reproduction number, mobility: number of trips by mode and and time spent in various locations
Interpretation of results	associative

– continued on next page –

Reporting of uncertainty	– not applicable –
Sensitivity analysis	none
Subgroup assessment	none

Tian et al. 2020 (Analysis 1)

**Study information**	
Author	Tian J, Wu AJ, Bao Y, Weng X, Shi L, Liu B, Yu X, Qi L, And ZL
Title	Modeling Analysis of COVID-19 Based on Morbidity Data in Anhui, China
Year	2020
Journal	Mathematical Biosciences and Engineering
DOI	10.3934/mbe.2020158
Date received	2020-02-22
Date accepted	2020-03-20
**Study setting**	
Number of populations included	single
Level of populations included	subnational (Anhui province (China))
Geographic areas covered	Asia
Number of countries covered	single country (China)
Study period	start and end date span first epidemic wave
**Outcome**	
Raw outcome	epidemiological population-level outcome (deaths)
Time resolution of raw outcome	daily
Computed outcome	none

– continued on next page –

Method to obtain the computed outcome	– not applicable –
Data source	data from (sub)national authorities
Data availability	data access via source
**Intervention**	
Terminology for interventions	measures
Terminology for the specific type of non-pharmaceutical interventions	intervention
Exposure types	one combination of interventions (Wuhan City Lockdown)
Types of single interventions	– not applicable –
Coding of interventions	not necessary
Source of intervention data	– could not be evaluated –
Availability of data on exposure	raw data documented in the manuscript
**Methodological approach**	
Empirical approach	parametric
Use of exposure variation	only variation over time for a single population
Method	non-mechanistic model (generalized linear model)
Code availability	none
**Effectiveness assessment**	
Reporting of intervention effectiveness	quantification of change in outcome values
Measure of effectiveness	deaths
Interpretation of results	implicitly causal
Reporting of uncertainty	yes
Sensitivity analysis	none
Subgroup assessment	none

Tian et al. 2020 (Analysis 2)	
**Study information**	
Author	Tian J, Wu AJ, Bao Y, Weng X, Shi L, Liu B, Yu X, Qi L, And ZL
Title	Modeling Analysis of COVID-19 Based on Morbidity Data in Anhui, China
Year	2020
Journal	Mathematical Biosciences and Engineering
DOI	10.3934/mbe.2020158
Date received	2020-02-22
Date accepted	2020-03-20
**Study setting**	
Number of populations included	single
Level of populations included	subnational (Anhui province (China))
Geographic areas covered	Asia
Number of countries covered	single country (China)
Study period	start and end date span first epidemic wave
**Outcome**	
Raw outcome	epidemiological population-level outcome (deaths)
Time resolution of raw outcome	daily
Computed outcome	epidemiological parameter (reproduction number)
Method to obtain the computed outcome	compartmental transmission model (single-population)
Data source	data from (sub)national authorities
Data availability	data access via source
**Intervention**	
Terminology for interventions	measures

– continued on next page –

Terminology for the specific type of non-pharmaceutical interventions	intervention
Exposure types	one combination of interventions (Wuhan City Lockdown)
Types of single interventions	– not applicable –
Coding of interventions	not necessary
Source of intervention data	– could not be evaluated –
Availability of data on exposure	raw data documented in the manuscript
**Methodological approach**	
Empirical approach	descriptive
Use of exposure variation	only variation over time for a single population
Method	description of change over time (comparison of time periods)
Code availability	none
**Effectiveness assessment**	
Reporting of intervention effectiveness	comparison of outcome values
Measure of effectiveness	reproduction number
Interpretation of results	implicitly causal
Reporting of uncertainty	no
Sensitivity analysis	none
Subgroup assessment	none

Tian et al. 2021

**Study information**	
Author	Tian T, Luo W, Tan J, Jiang Y, Chen M, Pan W, Yang S, Zhao J, Wang X, Zhang H

– continued on next page –

Title	The Timing and Effectiveness of Implementing Mild Interventions of COVID-19 in Large Industrial Regions via a Synthetic Control Method
Year	2021
Journal	Statistics and Its Interface
DOI	10.4310/20-sii634
Date received	2020-07-16
Date accepted	2020-08-30
**Study setting**	
Number of populations included	single
Level of populations included	subnational (Shenzhen)
Geographic areas covered	Asia
Number of countries covered	single country (China)
Study period	end date in growth phase of wave
**Outcome**	
Raw outcome	epidemiological population-level outcome (cases)
Time resolution of raw outcome	daily
Computed outcome	none
Method to obtain the computed outcome	– not applicable –
Data source	data from other research projects (study by Wu et al.)
Data availability	data made available by the authors
**Intervention**	
Terminology for interventions	interventions
Terminology for the specific type of non-pharmaceutical interventions	– not applicable –
Exposure types	one combination of interventions (Wuhan City Lockdown)

– continued on next page –

Types of single interventions	– not applicable –
Coding of interventions	not necessary
Source of intervention data	– could not be evaluated –
Availability of data on exposure	raw data documented in the manuscript
**Methodological approach**	
Empirical approach	counterfactual
Use of exposure variation	only variation over time for a single population
Method	synthetic controls
Code availability	publicly available
**Effectiveness assessment**	
Reporting of intervention effectiveness	quantification of change in outcome values
Measure of effectiveness	cases
Interpretation of results	implicitly causal
Reporting of uncertainty	yes
Sensitivity analysis	none
Subgroup assessment	none

Tiwari et al. 2020

**Study information**	
Author	Tiwari V, Deyal N, Bisht NS
Title	Mathematical Modeling Based Study and Prediction of COVID-19 Epidemic Dissemination under the Impact of Lockdown in India
Year	2020

– continued on next page –

Journal	Frontiers in Physics
DOI	10.3389/fphy.2020.586899
Date received	2020-07-24
Date accepted	2020-09-07
**Study setting**	
Number of populations included	single
Level of populations included	national (India)
Geographic areas covered	Asia
Number of countries covered	single country (India)
Study period	end date in growth phase of wave
**Outcome**	
Raw outcome	epidemiological population-level outcome (cases, deaths, recovered cases)
Time resolution of raw outcome	daily
Computed outcome	epidemiological parameter (reproduction number)
Method to obtain the computed outcome	compartmental transmission model (single-population)
Data source	data from (sub)national authorities
Data availability	data access via source
**Intervention**	
Terminology for interventions	– not applicable –
Terminology for the specific type of non-pharmaceutical interventions	lockdown
Exposure types	one combination of interventions (Indian National Lockdown)
Types of single interventions	– not applicable –
Coding of interventions	not necessary
Source of intervention data	government or news websites

– continued on next page –

Availability of data on exposure	raw data documented in the manuscript
**Methodological approach**	
Empirical approach	descriptive
Use of exposure variation	only variation over time for a single population
Method	description of change over time (comparison of time periods)
Code availability	none
**Effectiveness assessment**	
Reporting of intervention effectiveness	qualitative statement
Measure of effectiveness	cases, reproduction number
Interpretation of results	associative
Reporting of uncertainty	– not applicable –
Sensitivity analysis	none
Subgroup assessment	none

Tsai et al. 2020

**Study information**	
Author	Tsai AC, Harling G, Reynolds Z, Gilbert RF, Siedner MJ
Title	Coronavirus Disease 2019 (COVID-19) Transmission in the United States before versus after Relaxation of Statewide Social Distancing Measures
Year	2020
Journal	Clinical Infectious Diseases
DOI	10.1093/cid/ciaa1502
Date received	2020-08-04

– continued on next page –

Date accepted	2020-09-28
**Study setting**	
Number of populations included	multiple
Level of populations included	both national and subnational (US and its states)
Geographic areas covered	North America
Number of countries covered	single country (US)
Study period	same end date for several populations with diverse epidemic trajectories
**Outcome**	
Raw outcome	epidemiological population-level outcome (effective reproduction number)
Time resolution of raw outcome	daily
Computed outcome	none
Method to obtain the computed outcome	– not applicable –
Data source	data from other research projects (study by Unwin et al.)
Data availability	data access via source
**Intervention**	
Terminology for interventions	measures
Terminology for the specific type of non-pharmaceutical interventions	social distancing
Exposure types	multiple separate interventions (school closure (reopening of schools), workplace closure (easing of working restrictions), venue closure (reopening of businesses, reopening of outdoor recreational facilities), stay-at-home order (rescission of state-wide restrictions on movement: shelter-in-place order or lockdown), travel restrictions (rescission of mandatory quarantines for interstate travel))
Types of single interventions	school closure, stay-at-home order, international travel restrictions, workplace closure, venue closure

– continued on next page –

Coding of interventions	necessary
Source of intervention data	coding done by authors
Availability of data on exposure	coded data made available by the authors
**Methodological approach**	
Empirical approach	parametric
Use of exposure variation	both variation over time and between populations
Method	non-mechanistic model (generalized linear model)
Code availability	none
**Effectiveness assessment**	
Reporting of intervention effectiveness	quantification of change in outcome values
Measure of effectiveness	reproduction number
Interpretation of results	associative
Reporting of uncertainty	yes
Sensitivity analysis	different or modified outcome used (cases and deaths used as raw different or modified outcome used, estimates of effective reproduction number used from other paper), different coding of interventions used (days since relaxation of SIPO used as primary explanatory variable), model specification varied
Subgroup assessment	none

Turk et al. 2020

**Study information**	
Author	Turk PJ, Chou SH, Kowalkowski MA, Palmer PP, Priem JS, Spencer MD, Taylor YJ, McWilliams AD

– continued on next page –

Title	Modeling COVID-19 Latent Prevalence to Assess a Public Health Intervention at a State and Regional Scale: Retrospective Cohort Study
Year	2020
Journal	JMIR Public Health and Surveillance
DOI	10.2196/19353
Date received	2020-04-14
Date accepted	2020-05-18
**Study setting**	
Number of populations included	multiple
Level of populations included	subnational (North Carolina and Charlotte region)
Geographic areas covered	North America
Number of countries covered	single country (US)
Study period	end date in growth phase of wave
**Outcome**	
Raw outcome	epidemiological population-level outcome (cases, deaths)
Time resolution of raw outcome	daily
Computed outcome	measure of epidemic trend (doubling time), epidemiological parameter (reproduction number)
Method to obtain the computed outcome	compartmental transmission model (single-population)
Data source	data from (sub)national authorities
Data availability	data access via source
**Intervention**	
Terminology for interventions	interventions
Terminology for the specific type of non-pharmaceutical interventions	public health

– continued on next page –

Exposure types	one single intervention (stay-at-home order (in Mecklenburg & Co))
Types of single interventions	stay-at-home order
Coding of interventions	not necessary
Source of intervention data	– could not be evaluated –
Availability of data on exposure	raw data documented in the manuscript
**Methodological approach**	
Empirical approach	descriptive
Use of exposure variation	only variation over time for multiple populations
Method	description of change over time (comparison of time periods)
Code availability	none
**Effectiveness assessment**	
Reporting of intervention effectiveness	comparison of outcome values
Measure of effectiveness	reproduction number, doubling time
Interpretation of results	associative
Reporting of uncertainty	yes
Sensitivity analysis	none
Subgroup assessment	none

Tutsoy et al. 2020

**Study information**	
Author	Tutsoy O, Polat A, Colak S, Balikci K
Title	Development of a Multi-Dimensional Parametric Model with Non-Pharmacological Policies for Predicting the COVID-19 Pandemic Casualties

– continued on next page –

Year	2020
Journal	IEEE Access
DOI	10.1109/access.2020.3044929
Date received	2020-12-09
Date accepted	2020-12-11
**Study setting**	
Number of populations included	single
Level of populations included	national (Turkey)
Geographic areas covered	Middle East and Africa
Number of countries covered	single country (Turkey)
Study period	start and end date span first epidemic wave
**Outcome**	
Raw outcome	epidemiological population-level outcome (tests, cases, deaths)
Time resolution of raw outcome	daily
Computed outcome	none
Method to obtain the computed outcome	– not applicable –
Data source	data from (sub)national authorities
Data availability	data access via source
**Intervention**	
Terminology for interventions	policies
Terminology for the specific type of non-pharmaceutical interventions	non-pharmacological
Exposure types	multiple separate interventions (stay-at-home order (curfews for people with chronic diseases, curfews for people over 65, curfews for people under 20, curfews for everyone), school closure)

– continued on next page –

Tutsoy et al. 2020 – continued from previous page

Types of single interventions	stay-at-home order, school closure
Coding of interventions	not necessary
Source of intervention data	– could not be evaluated –
Availability of data on exposure	raw data documented in the manuscript
**Methodological approach**	
Empirical approach	parametric
Use of exposure variation	only variation over time for a single population
Method	other (non-standard, semi-mechanistic transmission model)
Code availability	none
**Effectiveness assessment**	
Reporting of intervention effectiveness	quantification of change in outcome values
Measure of effectiveness	tests, cases, deaths
Interpretation of results	implicitly causal
Reporting of uncertainty	no
Sensitivity analysis	none
Subgroup assessment	none

Utamura et al. 2020

**Study information**	
Author	Utamura M, Koizumi M, Kirikami S
Title	An Epidemiological Model Considering Isolation to Predict COVID-19 Trends in Tokyo, Japan: Numerical Analysis
Year	2020

– continued on next page –

Journal	JMIR Public Health and Surveillance
DOI	10.2196/23624
Date received	2020-08-18
Date accepted	2020-11-30
**Study setting**	
Number of populations included	single
Level of populations included	subnational (Tokyo (Japan))
Geographic areas covered	Asia
Number of countries covered	single country (Japan)
Study period	start and end date span first epidemic wave
**Outcome**	
Raw outcome	epidemiological population-level outcome (cases)
Time resolution of raw outcome	daily
Computed outcome	none
Method to obtain the computed outcome	– not applicable –
Data source	data from (sub)national authorities
Data availability	data access via source
**Intervention**	
Terminology for interventions	interventions
Terminology for the specific type of non-pharmaceutical interventions	public health
Exposure types	one single intervention (stay-at-home order)
Types of single interventions	stay-at-home order
Coding of interventions	not necessary
Source of intervention data	– could not be evaluated –

– continued on next page –

Availability of data on exposure	raw data documented in the manuscript
**Methodological approach**	
Empirical approach	parametric
Use of exposure variation	only variation over time for a single population
Method	mechanistic model (compartmental single-population transmission model)
Latent variable functionally linked to intervention	transmission rate
Code availability	none
**Effectiveness assessment**	
Reporting of intervention effectiveness	quantification of change in outcome values
Measure of effectiveness	transmission rate
Interpretation of results	implicitly causal
Reporting of uncertainty	no
Sensitivity analysis	none
Subgroup assessment	none

Valcarcel et al. 2020

**Study information**	
Author	Valcarcel B, Avilez JL, Torres-Roman JS, Poterico JA, Bazalar-Palacios J, Vecchia C
Title	The Effect of Early-Stage Public Health Policies in the Transmission of COVID-19 for South American Countries
Year	2020
Journal	Revista Panamericana de Salud Pública

– continued on next page –

DOI	10.26633/rpsp.2020.148
Date received	– could not be evaluated –
Date accepted	2020-11-10 (published)
**Study setting**	
Number of populations included	multiple
Level of populations included	national (10 South American countries)
Geographic areas covered	Central and South America
Number of countries covered	multiple countries
Study period	start and end date span first epidemic wave
**Outcome**	
Raw outcome	epidemiological population-level outcome (cases)
Time resolution of raw outcome	daily
Computed outcome	epidemiological parameter (reproduction number)
Method to obtain the computed outcome	statistical estimation of reproduction number (Thompson et al.)
Data source	data from publicly available cross-country selections (ECDC)
Data availability	data made available by the authors
**Intervention**	
Terminology for interventions	measures
Terminology for the specific type of non-pharmaceutical interventions	public health
Exposure types	multiple combinations of interventions (group 1: isolation, quarantine, and social distancing, group 2: community-wide containment)
Types of single interventions	– not applicable –
Coding of interventions	necessary
Source of intervention data	– could not be evaluated –

– continued on next page –

Availability of data on exposure	coded data not available
**Methodological approach**	
Empirical approach	descriptive
Use of exposure variation	only variation over time for multiple populations
Method	comparison of points in time (description of time course)
Code availability	publicly available
**Effectiveness assessment**	
Reporting of intervention effectiveness	comparison of outcome values
Measure of effectiveness	reproduction number
Interpretation of results	associative
Reporting of uncertainty	yes
Sensitivity analysis	model specification varied
Subgroup assessment	public health response (response time)

**Valencia et al. 2020 (Analysis 1)**	
**Study information**	
Author	Valencia M, Becerra JE, Reyes JC, Castro KG
Title	Assessment of Early Mitigation Measures against COVID-19 in Puerto Rico: March 15-May 15, 2020
Year	2020
Journal	PLOS ONE
DOI	10.1371/journal.pone.0240013

– continued on next page –

Date received	2020-06-05
Date accepted	2020-09-17
**Study setting**	
Number of populations included	single
Level of populations included	subnational (Puerto Rico)
Geographic areas covered	North America
Number of countries covered	single country (US)
Study period	start and end date span first epidemic wave
**Outcome**	
Raw outcome	epidemiological population-level outcome (cases, deaths)
Time resolution of raw outcome	daily
Computed outcome	epidemiological parameter (reproduction number)
Method to obtain the computed outcome	exponential growth model
Data source	data from (sub)national authorities
Data availability	data made available by the authors
**Intervention**	
Terminology for interventions	interventions
Terminology for the specific type of non-pharmaceutical interventions	non-pharmaceutical
Exposure types	one combination of interventions (curfew (as part of a state of emergency declaration closing public and private schools and universities, all nonessential businesses and public agencies, and implementing travel restrictions))
Types of single interventions	– not applicable –
Coding of interventions	not necessary
Source of intervention data	government or news websites

– continued on next page –

Availability of data on exposure	raw data documented in the manuscript
**Methodological approach**	
Empirical approach	descriptive
Use of exposure variation	only variation over time for a single population
Method	description of change over time (comparison of time periods)
Code availability	publicly available
**Effectiveness assessment**	
Reporting of intervention effectiveness	comparison of outcome values
Measure of effectiveness	reproduction number
Interpretation of results	associative
Reporting of uncertainty	yes
Sensitivity analysis	none
Subgroup assessment	none

Valencia et al. 2020 (Analysis 2)

**Study information**	
Author	Valencia M, Becerra JE, Reyes JC, Castro KG
Title	Assessment of Early Mitigation Measures against COVID-19 in Puerto Rico: March 15-May 15, 2020
Year	2020
Journal	PLOS ONE
DOI	10.1371/journal.pone.0240013
Date received	2020-06-05
Date accepted	2020-09-17

– continued on next page –

**Study setting**	
Number of populations included	single
Level of populations included	subnational (Puerto Rico)
Geographic areas covered	North America
Number of countries covered	single country (US)
Study period	start and end date span first epidemic wave
**Outcome**	
Raw outcome	epidemiological population-level outcome (cases, deaths)
Time resolution of raw outcome	daily
Computed outcome	none
Method to obtain the computed outcome	– not applicable –
Data source	data from (sub)national authorities
Data availability	data made available by the authors
**Intervention**	
Terminology for interventions	interventions
Terminology for the specific type of non-pharmaceutical interventions	non-pharmaceutical
Exposure types	one combination of interventions (curfew (as part of a state of emergency declaration closing public and private schools and universities, all nonessential businesses and public agencies, and implementing travel restrictions))
Types of single interventions	– not applicable –
Coding of interventions	not necessary
Source of intervention data	government or news websites
Availability of data on exposure	raw data documented in the manuscript
**Methodological approach**	

– continued on next page –

Empirical approach	counterfactual
Use of exposure variation	only variation over time for a single population
Method	non-mechanistic model (exponential growth model)
Code availability	publicly available
**Effectiveness assessment**	
Reporting of intervention effectiveness	comparison of outcome values
Measure of effectiveness	cases, deaths
Interpretation of results	associative
Reporting of uncertainty	no
Sensitivity analysis	none
Subgroup assessment	none

Van Dyke et al. 2020

**Study information**	
Author	Van Dyke ME, Rogers TM, Pevzner E, Satterwhite CL, Shah HB, Beckman WJ, Ahmed F, Hunt DC, Rule J
Title	Trends in County-Level COVID-19 Incidence in Counties with and without a Mask Mandate —Kansas, June 1–August 23, 2020
Year	2020
Journal	Morbidity and Mortality Weekly Report
DOI	10.15585/mmwr.mm6947e2
Date received	– could not be evaluated –
Date accepted	2020-11-20
**Study setting**	

– continued on next page –

Number of populations included	multiple
Level of populations included	subnational (Counties in Kansas)
Geographic areas covered	North America
Number of countries covered	single country (US)
Study period	includes second wave (two waves)
**Outcome**	
Raw outcome	epidemiological population-level outcome (cases)
Time resolution of raw outcome	daily
Computed outcome	none
Method to obtain the computed outcome	– not applicable –
Data source	public (USAFacts)
Data availability	data access via source
**Intervention**	
Terminology for interventions	strategies
Terminology for the specific type of non-pharmaceutical interventions	mitigation
Exposure types	one single intervention (mask mandate)
Types of single interventions	mask mandate
Coding of interventions	necessary
Source of intervention data	use of externally coded data (Kansas Health Institute)
Availability of data on exposure	access to externally coded data via source
**Methodological approach**	
Empirical approach	parametric
Use of exposure variation	both variation over time and between populations
Method	non-mechanistic model (generalized linear model)

– continued on next page –

Code availability	none
**Effectiveness assessment**	
Reporting of intervention effectiveness	quantification of change in outcome values
Measure of effectiveness	cases
Interpretation of results	associative
Reporting of uncertainty	yes
Sensitivity analysis	different coding of interventions used (different NPI coding, i.e., nonmandated counties that included cities mandating masks coded as mandated counties)
Subgroup assessment	public health response (subject to other measures: with and without other NPIs)

**Vannoni et al. 2020**	
**Study information**	
Author	Vannoni M, McKee M, Semenza JC, Bonell C, Stuckler D
Title	Using Volunteered Geographic Information to Assess Mobility in the Early Phases of the COVID-19 Pandemic: A Cross-City Time Series Analysis of 41 Cities in 22 Countries from March 2nd to 26th 2020
Year	2020
Journal	Globalization and Health
DOI	10.1186/s12992-020-00598-9
Date received	2020-04-21
Date accepted	2020-07-13
**Study setting**	

– continued on next page –

Number of populations included	multiple
Level of populations included	subnational (41 cities in 22 countries)
Geographic areas covered	Europe, Middle East and Africa, Asia, Oceania, Central and South America, North America
Number of countries covered	multiple countries
Study period	start and end date span first epidemic wave: behavioral
**Outcome**	
Raw outcome	behavioral outcome (mobility: mobility index based on number of trips planned compared to a reference period)
Time resolution of raw outcome	daily
Computed outcome	none
Method to obtain the computed outcome	– not applicable –
Data source	mobility data from corporate organizations (Citymapper)
Data availability	data access via source
**Intervention**	
Terminology for interventions	interventions
Terminology for the specific type of non-pharmaceutical interventions	non-pharmaceutical
Exposure types	multiple separate interventions (school closure, bans of large gatherings, workplace closure, international travel restrictions, internal movement restrictions, public transport closure)
Types of single interventions	school closure, bans of large gatherings, workplace closure, international travel restrictions, internal movement restrictions, public transport closure
Coding of interventions	necessary
Source of intervention data	use of externally coded data (OxCGRT)
Availability of data on exposure	access to externally coded data via source

– continued on next page –

**Methodological approach**	
Empirical approach	parametric
Use of exposure variation	both variation over time and between populations
Method	non-mechanistic model (generalized linear model)
Code availability	none
**Effectiveness assessment**	
Reporting of intervention effectiveness	quantification of change in outcome values
Measure of effectiveness	mobility: mobility index based on number of trips planned compared to a reference period
Interpretation of results	associative
Reporting of uncertainty	yes
Sensitivity analysis	different or modified outcome used (different mobility data used)
Subgroup assessment	none

**Verma et al. 2020**	
**Study information**	
Author	Verma BK, Verma M, Verma VK, Abdullah RB, Nath DC, Khan HT, Verma A, Vishwakarma RK, Verma V
Title	Global Lockdown: An Effective Safeguard in Responding to the Threat of COVID-19
Year	2020
Journal	Journal of Evaluation in Clinical Practice
DOI	10.1111/jep.13483
Date received	2020-06-22

– continued on next page –

Date accepted	2020-09-01
**Study setting**	
Number of populations included	multiple
Level of populations included	national (United States, Italy, United Kingdom, France, India, Russia)
Geographic areas covered	Asia, Europe, North America
Number of countries covered	multiple countries
Study period	same end date for several populations with diverse epidemic trajectories
**Outcome**	
Raw outcome	epidemiological population-level outcome (cases)
Time resolution of raw outcome	daily
Computed outcome	change points (change points: cases)
Method to obtain the computed outcome	change point analysis
Data source	data from publicly available cross-country selections (OurWorldInData)
Data availability	data access via source
**Intervention**	
Terminology for interventions	– not applicable –
Terminology for the specific type of non-pharmaceutical interventions	lockdown
Exposure types	one combination of interventions (lockdown with specific date for each country)
Types of single interventions	– not applicable –
Coding of interventions	not necessary
Source of intervention data	– could not be evaluated –
Availability of data on exposure	raw data documented in the manuscript
**Methodological approach**	

– continued on next page –

Empirical approach	descriptive
Use of exposure variation	only variation over time for multiple populations
Method	comparison of change points with intervention
Code availability	none
**Effectiveness assessment**	
Reporting of intervention effectiveness	quantification of change in outcome values
Measure of effectiveness	change points: cases
Interpretation of results	implicitly causal
Reporting of uncertainty	yes
Sensitivity analysis	none
Subgroup assessment	none

Vicentini et al. 2020

**Study information**	
Author	Vicentini C, Bordino V, Gardois P, Zotti CM
Title	Early Assessment of the Impact of Mitigation Measures on the COVID-19 Outbreak in Italy
Year	2020
Journal	Public Health
DOI	10.1016/j.puhe.2020.06.028
Date received	2020-04-28
Date accepted	2020-06-10
**Study setting**	

– continued on next page –

Number of populations included	single
Level of populations included	national (Italy)
Geographic areas covered	Europe
Number of countries covered	single country (Italy)
Study period	start and end date span first epidemic wave
**Outcome**	
Raw outcome	epidemiological population-level outcome (ICU hospitalizations)
Time resolution of raw outcome	daily
Computed outcome	none
Method to obtain the computed outcome	– not applicable –
Data source	data from (sub)national authorities
Data availability	data access via source
**Intervention**	
Terminology for interventions	measures
Terminology for the specific type of non-pharmaceutical interventions	mitigation
Exposure types	multiple combinations of interventions (lockdown of the epicenter of the outbreak, school closure and nationwide lockdown)
Types of single interventions	– not applicable –
Coding of interventions	not necessary
Source of intervention data	– could not be evaluated –
Availability of data on exposure	raw data documented in the manuscript
**Methodological approach**	
Empirical approach	counterfactual
Use of exposure variation	only variation over time for a single population

– continued on next page –

Method	non-mechanistic model (exponential growth model)
Code availability	none
**Effectiveness assessment**	
Reporting of intervention effectiveness	comparison of outcome values
Measure of effectiveness	ICU hospitalizations
Interpretation of results	implicitly causal
Reporting of uncertainty	no
Sensitivity analysis	none
Subgroup assessment	none

Wagner et al. 2020

**Study information**	
Author	Wagner AB, Hill EL, Ryan SE, Sun Z, Deng G, Bhadane S, Martinez VH, Wu P, Li D, Anand A, Acharya J, Matteson DS
Title	Social Distancing Merely Stabilized COVID-19 in the United States
Year	2020
Journal	Stat
DOI	10.1002/sta4.302
Date received	2020-05-11
Date accepted	2020-06-28
**Study setting**	
Number of populations included	multiple
Level of populations included	both national and subnational (US and all states)

– continued on next page –

Geographic areas covered	North America
Number of countries covered	single country (US)
Study period	same end date for several populations with diverse epidemic trajectories
**Outcome**	
Raw outcome	epidemiological population-level outcome (cases)
Time resolution of raw outcome	daily
Computed outcome	none
Method to obtain the computed outcome	– not applicable –
Data source	data from public media outlets (New York Times)
Data availability	data made available by the authors
**Intervention**	
Terminology for interventions	measures
Terminology for the specific type of non-pharmaceutical interventions	social distancing
Exposure types	start of interventions (first NPI out of either school closure or venue closure (restaurant closures))
Types of single interventions	– not applicable –
Coding of interventions	necessary
Source of intervention data	use of externally coded data (Education Week, Wida)
Availability of data on exposure	coded data made available by the authors
**Methodological approach**	
Empirical approach	parametric
Use of exposure variation	only variation over time for multiple populations
Method	non-mechanistic model (generalized linear model)
Code availability	publicly available

– continued on next page –

**Effectiveness assessment**	
Reporting of intervention effectiveness	quantification of change in outcome values
Measure of effectiveness	doubling time
Interpretation of results	associative
Reporting of uncertainty	yes
Sensitivity analysis	different or modified outcome used (deaths used instead of confirmed cases), model specification varied
Subgroup assessment	none

Wahaibi et al. 2020

**Study information**	
Author	Wahaibi AA, Manji AA, Maani AA, Rawahi BA, Harthy KA, Alyaquobi F, Al-Jardani A, Petersen E, Abri SA
Title	COVID-19 Epidemic Monitoring after Non-Pharmaceutical Interventions: The Use of Time-Varying Reproduction Number in a Country with a Large Migrant Population
Year	2020
Journal	International Journal of Infectious Diseases
DOI	10.1016/j.ijid.2020.08.039
Date received	2020-07-27
Date accepted	2020-08-16
**Study setting**	
Number of populations included	single
Level of populations included	national (Oman)

– continued on next page –

Geographic areas covered	Middle East and Africa
Number of countries covered	single country (Oman)
Study period	end date in growth phase of wave
**Outcome**	
Raw outcome	epidemiological individual-level outcome (individual cases and transmission chains)
Time resolution of raw outcome	daily
Computed outcome	epidemiological parameter (reproduction number)
Method to obtain the computed outcome	statistical estimation of reproduction number (Cori et al.)
Data source	data from (sub)national authorities
Data availability	data not accessible
**Intervention**	
Terminology for interventions	interventions
Terminology for the specific type of non-pharmaceutical interventions	non-pharmaceutical
Exposure types	phases of interventions (various measures separated into three phases)
Types of single interventions	– not applicable –
Coding of interventions	not necessary
Source of intervention data	– could not be evaluated –
Availability of data on exposure	raw data documented in the manuscript
**Methodological approach**	
Empirical approach	descriptive
Use of exposure variation	only variation over time for a single population
Method	description of change over time (comparison of time periods)
Code availability	none

– continued on next page –

**Effectiveness assessment**	
Reporting of intervention effectiveness	comparison of outcome values
Measure of effectiveness	reproduction number
Interpretation of results	implicitly causal
Reporting of uncertainty	yes
Sensitivity analysis	none
Subgroup assessment	socioeconomic indicators (nationality)

Wan et al. 2020

**Study information**	
Author	Wan H, Cui JA, Yang GJ
Title	Risk Estimation and Prediction of the Transmission of Coronavirus Disease-2019 (COVID-19) in the Mainland of China Excluding Hubei Province
Year	2020
Journal	Infectious Diseases of Poverty
DOI	10.1186/s40249-020-00683-6
Date received	2020-03-02
Date accepted	2020-05-28
**Study setting**	
Number of populations included	single
Level of populations included	national (China exluding Hubei)
Geographic areas covered	Asia
Number of countries covered	single country (China)

– continued on next page –

Study period	start and end date span first epidemic wave
**Outcome**	
Raw outcome	epidemiological population-level outcome (cases, deaths, recovered cases)
Time resolution of raw outcome	daily
Computed outcome	epidemiological parameter (reproduction number)
Method to obtain the computed outcome	compartmental transmission model (single-population)
Data source	data from (sub)national authorities
Data availability	data access via source
**Intervention**	
Terminology for interventions	strategies, measures
Terminology for the specific type of non-pharmaceutical interventions	containment, control, self-protection
Exposure types	all interventions together
Types of single interventions	– not applicable –
Coding of interventions	– not applicable –
Source of intervention data	– not applicable –
Availability of data on exposure	– not applicable –
**Methodological approach**	
Empirical approach	descriptive
Use of exposure variation	only variation over time for a single population
Method	description of change over time (description of time course)
Code availability	none
**Effectiveness assessment**	
Reporting of intervention effectiveness	comparison of outcome values

– continued on next page –

Measure of effectiveness	reproduction number
Interpretation of results	implicitly causal
Reporting of uncertainty	no
Sensitivity analysis	none
Subgroup assessment	none

**Wang et al. 2020a**	
**Study information**	
Author	Wang Q, Xie S, Wang Y, Zeng D
Title	Survival-Convolution Models for Predicting COVID-19 Cases and Assessing Effects of Mitigation Strategies
Year	2020
Journal	Frontiers in Public Health
DOI	10.3389/fpubh.2020.00325
Date received	2020-05-11
Date accepted	2020-06-15
**Study setting**	
Number of populations included	multiple
Level of populations included	national (Italy and US)
Geographic areas covered	Europe, North America
Number of countries covered	multiple countries
Study period	start and end date span first epidemic wave
**Outcome**	
Raw outcome	epidemiological population-level outcome (cases)

– continued on next page –

Time resolution of raw outcome	daily
Computed outcome	none
Method to obtain the computed outcome	– not applicable –
Data source	data from publicly available cross-country selections (Worldometer)
Data availability	data made available by the authors
**Intervention**	
Terminology for interventions	interventions
Terminology for the specific type of non-pharmaceutical interventions	public health
Exposure types	one combination of interventions (nationwide lockdown of March 11 (Italy), emergency declaration (national US))
Types of single interventions	– not applicable –
Coding of interventions	not necessary
Source of intervention data	– could not be evaluated –
Availability of data on exposure	raw data documented in the manuscript
**Methodological approach**	
Empirical approach	parametric
Use of exposure variation	only variation over time for multiple populations
Method	other (discrete, deterministic, non-compartmental transmission model fit via gradient-based optimization)
Latent variable functionally linked to intervention	transmission rate
Code availability	publicly available
**Effectiveness assessment**	
Reporting of intervention effectiveness	quantification of change in outcome values

– continued on next page –

Measure of effectiveness	reproduction number, transmission rate
Interpretation of results	implicitly causal
Reporting of uncertainty	yes
Sensitivity analysis	none
Subgroup assessment	none

**Wang et al. 2020b**	
**Study information**	
Author	Wang T, Wu Y, Lau JY, Yu Y, Liu L, Li J, Zhang K, Tong W, Jiang B
Title	A Four-Compartment Model for the COVID-19 Infection —Implications on Infection Kinetics, Control Measures, and Lockdown Exit Strategies
Year	2020
Journal	Precision Clinical Medicine
DOI	10.1093/pcmedi/pbaa018
Date received	2020-05-21
Date accepted	2020-05-22
**Study setting**	
Number of populations included	multiple
Level of populations included	both national and subnational (Italy, UK, US, Wuhan)
Geographic areas covered	Asia, Europe, North America
Number of countries covered	multiple countries
Study period	end date at peak of wave
**Outcome**	
Raw outcome	epidemiological population-level outcome (cases)

– continued on next page –

Time resolution of raw outcome	daily
Computed outcome	none
Method to obtain the computed outcome	– not applicable –
Data source	– could not be evaluated –
Data availability	data not accessible
**Intervention**	
Terminology for interventions	measures
Terminology for the specific type of non-pharmaceutical interventions	control
Exposure types	one combination of interventions (Wuhan City Lockdown (China), lockdown decreed on March 10 (Italy), lockdown decreed March 23 and strengthened on March 30 (UK), shelter-in-place order (US))
Types of single interventions	– not applicable –
Coding of interventions	not necessary
Source of intervention data	– could not be evaluated –
Availability of data on exposure	raw data documented in the manuscript
**Methodological approach**	
Empirical approach	parametric
Use of exposure variation	only variation over time for multiple populations
Method	mechanistic model (compartmental single-population transmission model)
Latent variable functionally linked to intervention	contact rate, probability of transmission upon contact
Code availability	none
**Effectiveness assessment**	
Reporting of intervention effectiveness	quantification of change in outcome values

– continued on next page –

Measure of effectiveness	contact rate, probability of transmission upon contact
Interpretation of results	implicitly causal
Reporting of uncertainty	no
Sensitivity analysis	none
Subgroup assessment	none

Wang et al. 2020c (Analysis 1)

**Study information**	
Author	Wang S, Yang X, Li L, Nadler P, Arcucci R, Huang Y, Teng Z, Guo Y
Title	A Bayesian Updating Scheme for Pandemics: Estimating the Infection Dynamics of COVID-19
Year	2020
Journal	IEEE Computational Intelligence Magazine
DOI	10.1109/mci.2020.3019874
Date received	– could not be evaluated –
Date accepted	2020-10-15
**Study setting**	
Number of populations included	multiple
Level of populations included	national (14 European countries)
Geographic areas covered	Europe
Number of countries covered	multiple countries
Study period	start and end date span first epidemic wave
**Outcome**	

– continued on next page –

Raw outcome	epidemiological population-level outcome (cases)
Time resolution of raw outcome	daily
Computed outcome	epidemiological parameter (reproduction number, infectious period length, overall infectiousness)
Method to obtain the computed outcome	semi-mechanistic Bayesian transmission model
Data source	data from publicly available cross-country selections (JHU)
Data availability	data access via source
**Intervention**	
Terminology for interventions	measures
Terminology for the specific type of non-pharmaceutical interventions	intervention
Exposure types	stringency index (OxCGRT Stringency Index (categorized into five levels))
Types of single interventions	– not applicable –
Coding of interventions	necessary
Source of intervention data	use of externally coded data (OxCGRT)
Availability of data on exposure	access to externally coded data via source
**Methodological approach**	
Empirical approach	parametric
Use of exposure variation	both variation over time and between populations
Method	non-mechanistic model (generalized linear model)
Code availability	none
**Effectiveness assessment**	
Reporting of intervention effectiveness	quantification of change in outcome values
Measure of effectiveness	reproduction number, infectious period length, overall infectiousness

– continued on next page –

Interpretation of results	associative
Reporting of uncertainty	yes
Sensitivity analysis	none
Subgroup assessment	none

**Wang et al. 2020c (Analysis 2)**	
**Study information**	
Author	Wang S, Yang X, Li L, Nadler P, Arcucci R, Huang Y, Teng Z, Guo Y
Title	A Bayesian Updating Scheme for Pandemics: Estimating the Infection Dynamics of COVID-19
Year	2020
Journal	IEEE Computational Intelligence Magazine
DOI	10.1109/mci.2020.3019874
Date received	– could not be evaluated –
Date accepted	2020-10-15
**Study setting**	
Number of populations included	single
Level of populations included	subnational (Wuhan)
Geographic areas covered	Asia
Number of countries covered	single country (China)
Study period	start and end date span first epidemic wave
**Outcome**	
Raw outcome	epidemiological population-level outcome (cases)
Time resolution of raw outcome	daily

– continued on next page –

Computed outcome	epidemiological parameter (reproduction number, infectious period length, overall infectiousness)
Method to obtain the computed outcome	semi-mechanistic Bayesian transmission model
Data source	data from (sub)national authorities
Data availability	data access via source
**Intervention**	
Terminology for interventions	measures
Terminology for the specific type of non-pharmaceutical interventions	intervention
Exposure types	one combination of interventions (Wuhan City Lockdown)
Types of single interventions	– not applicable –
Coding of interventions	not necessary
Source of intervention data	– could not be evaluated –
Availability of data on exposure	raw data documented in the manuscript
**Methodological approach**	
Empirical approach	descriptive
Use of exposure variation	only variation over time for a single population
Method	description of change over time (comparison of time periods)
Code availability	none
**Effectiveness assessment**	
Reporting of intervention effectiveness	quantification of change in outcome values
Measure of effectiveness	reproduction number, infectious period length, overall infectiousness
Interpretation of results	associative
Reporting of uncertainty	no

– continued on next page –

Sensitivity analysis	none
Subgroup assessment	none

**Wang et al. 2020d**	
**Study information**	
Author	Wang K, Zhao S, Li H, Song Y, Wang L, Wang MH, Peng Z, Li H, He D
Title	Real-Time Estimation of the Reproduction Number of the Novel Coronavirus Disease (COVID-19) in China in 2020 Based on Incidence Data
Year	2020
Journal	Annals of Translational Medicine
DOI	10.21037/atm-20-1944
Date received	2020-02-26
Date accepted	2020-04-21
**Study setting**	
Number of populations included	multiple
Level of populations included	both national and subnational (China, Hubei province and Wuhan)
Geographic areas covered	Asia
Number of countries covered	single country (China)
Study period	start and end date span first epidemic wave
**Outcome**	
Raw outcome	epidemiological individual-level outcome (individual cases)
Time resolution of raw outcome	daily
Computed outcome	epidemiological parameter (reproduction number)

– continued on next page –

Method to obtain the computed outcome	statistical estimation of reproduction number (Wallinga and Teunis)
Data source	data from (sub)national authorities
Data availability	data not accessible
**Intervention**	
Terminology for interventions	– not applicable –
Terminology for the specific type of non-pharmaceutical interventions	lockdown
Exposure types	one combination of interventions (Wuhan City Lockdown)
Types of single interventions	– not applicable –
Coding of interventions	not necessary
Source of intervention data	– could not be evaluated –
Availability of data on exposure	raw data documented in the manuscript
**Methodological approach**	
Empirical approach	descriptive
Use of exposure variation	only variation over time for multiple populations
Method	description of change over time (comparison of time periods)
Code availability	none
**Effectiveness assessment**	
Reporting of intervention effectiveness	comparison of outcome values
Measure of effectiveness	reproduction number
Interpretation of results	associative
Reporting of uncertainty	yes

– continued on next page –

Sensitivity analysis	different or modified outcome used (varying levels of underreporting accounted for when computing effective reproduction number)
Subgroup assessment	none

**Wang et al. 2020e**	
**Study information**	
Author	Wang X, Ren R, Kattan MW, Jehi L, Cheng Z, Fang K
Title	Public Health Interventions' Effect on Hospital Use in Patients with COVID-19: Comparative Study
Year	2020
Journal	JMIR Public Health and Surveillance
DOI	10.2196/25174
Date received	2020-11-12
Date accepted	2020-12-06
**Study setting**	
Number of populations included	multiple
Level of populations included	subnational (New York, Ohio, Hubei)
Geographic areas covered	Asia, North America
Number of countries covered	multiple countries
Study period	start and end date span first epidemic wave
**Outcome**	
Raw outcome	epidemiological population-level outcome (cases, deaths, recovered cases)
Time resolution of raw outcome	daily
Computed outcome	none

– continued on next page –

Method to obtain the computed outcome	– not applicable –
Data source	data from (sub)national authorities (British Columbia and New Zealand), data from public media outlets (COVID Tracking Project by The Atlantic: US)
Data availability	data access via source
**Intervention**	
Terminology for interventions	interventions
Terminology for the specific type of non-pharmaceutical interventions	public health
Exposure types	one combination of interventions (Wuhan City Lockdown)
Types of single interventions	– not applicable –
Coding of interventions	not necessary
Source of intervention data	– could not be evaluated –
Availability of data on exposure	raw data documented in the manuscript
**Methodological approach**	
Empirical approach	parametric
Use of exposure variation	only variation over time for multiple populations
Method	mechanistic model (compartmental single-population transmission model)
Latent variable functionally linked to intervention	transmission rate
Code availability	none
**Effectiveness assessment**	
Reporting of intervention effectiveness	quantification of change in outcome values
Measure of effectiveness	transmission rate, reproduction number
Interpretation of results	implicitly causal

– continued on next page –

Reporting of uncertainty	yes
Sensitivity analysis	none
Subgroup assessment	none

**Wells et al. 2020**	
**Study information**	
Author	Wells CR, Sah P, Moghadas SM, Pandey A, Shoukat A, Wang Y, Wang Z, Meyers LA, Singer BH, Galvani AP
Title	Impact of International Travel and Border Control Measures on the Global Spread of the Novel 2019 Coronavirus Outbreak
Year	2020
Journal	Proceedings of the National Academy of Sciences
DOI	10.1073/pnas.2002616117
Date received	2020-02-12 (sent for review)
Date accepted	2020-03-13
**Study setting**	
Number of populations included	multiple
Level of populations included	national (China and 21 countries with imported cases)
Geographic areas covered	Asia
Number of countries covered	single country (China)
Study period	other (start date in December 2019 to estimate early exportation risk)
**Outcome**	
Raw outcome	epidemiological population-level outcome (cases, exported cases)
Time resolution of raw outcome	daily

– continued on next page –

Computed outcome	none
Method to obtain the computed outcome	– not applicable –
Data source	data collected by authors (from various sources)
Data availability	data made available by the authors
**Intervention**	
Terminology for interventions	measures
Terminology for the specific type of non-pharmaceutical interventions	travel and border control measures
Exposure types	one single intervention (travel restrictions)
Types of single interventions	international travel restrictions
Coding of interventions	not necessary
Source of intervention data	– could not be evaluated –
Availability of data on exposure	raw data documented in the manuscript
**Methodological approach**	
Empirical approach	counterfactual
Use of exposure variation	only variation over time for a single population
Method	other (extrapolate probability and risk of travels outside of China to predict exported cases in absence of intervention)
Code availability	publicly available
**Effectiveness assessment**	
Reporting of intervention effectiveness	quantification of change in outcome values
Measure of effectiveness	exported cases
Interpretation of results	implicitly causal
Reporting of uncertainty	yes

– continued on next page –

Sensitivity analysis	none
Subgroup assessment	none

**Wibbens et al. 2020**	
**Study information**	
Author	Wibbens PD, Koo WW, McGahan AM
Title	Which COVID Policies Are Most Effective? A Bayesian Analysis of COVID-19 by Jurisdiction
Year	2020
Journal	PLOS ONE
DOI	10.1371/journal.pone.0244177
Date received	2020-05-19
Date accepted	2020-12-04
**Study setting**	
Number of populations included	multiple
Level of populations included	both national and subnational (40 countries around the globe and US states)
Geographic areas covered	Europe, Middle East and Africa, Asia, Oceania, Central and South America, North America
Number of countries covered	multiple countries
Study period	start and end date span first epidemic wave
**Outcome**	
Raw outcome	epidemiological population-level outcome (cases, deaths, excess deaths)
Time resolution of raw outcome	daily
Computed outcome	none

– continued on next page –

Method to obtain the computed outcome	– not applicable –
Data source	data from publicly available cross-country selections (JHU)
Data availability	data made available by the authors
**Intervention**	
Terminology for interventions	measures
Terminology for the specific type of non-pharmaceutical interventions	control
Exposure types	multiple separate interventions (school closure, bans of large gatherings, bans of small gatherings, workplace closure, stay-at-home order, international travel restrictions, internal movement restrictions, public transport closure)
Types of single interventions	school closure, bans of large gatherings, bans of small gatherings, workplace closure, stay-at-home order, international travel restrictions, internal movement restrictions, public transport closure
Coding of interventions	necessary
Source of intervention data	use of externally coded data (OxCGRT)
Availability of data on exposure	coded data made available by the authors
**Methodological approach**	
Empirical approach	parametric
Use of exposure variation	both variation over time and between populations
Method	mechanistic model (semi-mechanistic Bayesian transmission model)
Latent variable functionally linked to intervention	other (growth rate of new cases)
Code availability	publicly available
**Effectiveness assessment**	

– continued on next page –

Reporting of intervention effectiveness	quantification of change in outcome values
Measure of effectiveness	growth rate: cases
Interpretation of results	implicitly causal
Reporting of uncertainty	yes
Sensitivity analysis	same analysis with (sub)population(s) excluded, model specification varied
Subgroup assessment	epidemiological indicators (pre-intervention epidemic trend)

Wieland 2020 (Analysis 1)

**Study information**	
Author	Wieland T
Title	A Phenomenological Approach to Assessing the Effectiveness of COVID-19 Related Nonpharmaceutical Interventions in Germany
Year	2020
Journal	Safety Science
DOI	10.1016/j.ssci.2020.104924
Date received	– could not be evaluated –
Date accepted	2020-07-21 (published)
**Study setting**	
Number of populations included	single
Level of populations included	national (Germany)
Geographic areas covered	Europe
Number of countries covered	single country (Germany)

– continued on next page –

Study period	start and end date span first epidemic wave
**Outcome**	
Raw outcome	epidemiological individual-level outcome (individual cases)
Time resolution of raw outcome	daily
Computed outcome	measure of epidemic trend (growth rate: infections), epidemiological parameter (reproduction number), change points (change points: infections)
Method to obtain the computed outcome	back projection, change point analysis, statistical estimation of reproduction number (AD Heiden and Hamouda)
Data source	data from (sub)national authorities
Data availability	data not accessible
**Intervention**	
Terminology for interventions	interventions
Terminology for the specific type of non-pharmaceutical interventions	non-pharmaceutical
Exposure types	multiple combinations of interventions (1. cancellation of mass events after March 8, 2. closure of schools and child day care facilities between March 16 and 18, and 3. a contact ban, bans of gatherings and closures of “nonessential” services from March 23)
Types of single interventions	– not applicable –
Coding of interventions	not necessary
Source of intervention data	– could not be evaluated –
Availability of data on exposure	raw data documented in the manuscript
**Methodological approach**	
Empirical approach	descriptive
Use of exposure variation	only variation over time for a single population
Method	comparison of change points with intervention

– continued on next page –

Wieland 2020 (Analysis 1) – continued from previous page

Code availability	none
**Effectiveness assessment**	
Reporting of intervention effectiveness	comparison of outcome values
Measure of effectiveness	change points: infections, growth rate: infections, reproduction number
Interpretation of results	implicitly causal
Reporting of uncertainty	yes
Sensitivity analysis	none
Subgroup assessment	none

Wieland 2020 (Analysis 2)

**Study information**	
Author	Wieland T
Title	A Phenomenological Approach to Assessing the Effectiveness of COVID-19 Related Nonpharmaceutical Interventions in Germany
Year	2020
Journal	Safety Science
DOI	10.1016/j.ssci.2020.104924
Date received	– could not be evaluated –
Date accepted	2020-07-21 (published)
**Study setting**	
Number of populations included	single
Level of populations included	national (Germany)
Geographic areas covered	Europe
Number of countries covered	single country (Germany)

– continued on next page –

Study period	start and end date span first epidemic wave
**Outcome**	
Raw outcome	epidemiological individual-level outcome (individual cases)
Time resolution of raw outcome	daily
Computed outcome	measure of epidemic trend (growth rate: infections), epidemiological parameter (reproduction number)
Method to obtain the computed outcome	back projection, change point analysis, statistical estimation of reproduction number (AD Heiden and Hamouda)
Data source	data from (sub)national authorities
Data availability	data not accessible
**Intervention**	
Terminology for interventions	interventions
Terminology for the specific type of non-pharmaceutical interventions	non-pharmaceutical
Exposure types	multiple combinations of interventions (estimated change points from time series that correspond with the implementation dates of NPIs to some extent of schools and child day care facilities between March 16 and 18, and 3. a contact ban, bans of gatherings and closures of "nonessential" services from March 23)
Types of single interventions	– not applicable –
Coding of interventions	not necessary
Source of intervention data	– could not be evaluated –
Availability of data on exposure	raw data documented in the manuscript
**Methodological approach**	
Empirical approach	descriptive
Use of exposure variation	only variation over time for a single population
Method	description of change over time (comparison of time periods)

– continued on next page –

Code availability	none
**Effectiveness assessment**	
Reporting of intervention effectiveness	comparison of outcome values
Measure of effectiveness	infections, growth rate: infections, reproduction number
Interpretation of results	implicitly causal
Reporting of uncertainty	yes
Sensitivity analysis	none
Subgroup assessment	none

Wielechowski et al. 2020 (Analysis 1)

**Study information**	
Author	Wielechowski M, Czech K, Grzeda L
Title	Decline in Mobility: Public Transport in Poland in the Time of the COVID-19 Pandemic
Year	2020
Journal	Economies
DOI	10.3390/economies8040078
Date received	2020-08-13
Date accepted	2020-09-15
**Study setting**	
Number of populations included	multiple
Level of populations included	both national and subnational (Poland and its 16 vovoidships)

– continued on next page –

Geographic areas covered	Europe
Number of countries covered	single country (Poland)
Study period	start and end date span first epidemic wave: behavioral
**Outcome**	
Raw outcome	behavioral outcome (mobility: time spent in public transport stations)
Time resolution of raw outcome	daily
Computed outcome	none
Method to obtain the computed outcome	– not applicable –
Data source	mobility data from corporate organizations (Google)
Data availability	data access via source
**Intervention**	
Terminology for interventions	policies
Terminology for the specific type of non-pharmaceutical interventions	government
Exposure types	stringency index (OxCGRT Stringency Index)
Types of single interventions	– not applicable –
Coding of interventions	necessary
Source of intervention data	use of externally coded data (OxCGRT)
Availability of data on exposure	access to externally coded data via source
**Methodological approach**	
Empirical approach	parametric
Use of exposure variation	only variation over time for multiple populations
Method	non-mechanistic model (correlation)
Code availability	none
**Effectiveness assessment**	

– continued on next page –

Reporting of intervention effectiveness	quantification of change in outcome values
Measure of effectiveness	mobility: time spent in public transport stations
Interpretation of results	implicitly causal
Reporting of uncertainty	yes
Sensitivity analysis	none
Subgroup assessment	none

**Wielechowski et al. 2020 (Analysis 2, mobility as exposure)**	
**Study information**	
Author	Wielechowski M, Czech K, Grzeda L
Title	Decline in Mobility: Public Transport in Poland in the Time of the COVID-19 Pandemic
Year	2020
Journal	Economies
DOI	10.3390/economies8040078
Date received	13.08.2020
Date accepted	15.09.2020
**Study setting**	
Number of populations included	multiple
Level of populations included	both national and subnational (Poland and its 16 vovoidships)
Geographic areas covered	Europe
Number of countries covered	single country (Poland)
Study period	start and end date span first epidemic wave
**Outcome**	

– continued on next page –

Raw outcome	epidemiological population-level outcome (cases)
Time resolution of raw outcome	daily
Computed outcome	none
Method to obtain the computed outcome	– not applicable –
Data source	data from (sub)national authorities
Data availability	data access via source
**Intervention**	
Terminology for interventions	policies
Terminology for the specific type of non-pharmaceutical interventions	government
Exposure types	mobility (time spent in public transport stations)
Types of single interventions	– not applicable –
Coding of interventions	– not applicable –
Source of intervention data	mobility data by corporate organizations (Google)
Availability of data on exposure	data access via source
**Methodological approach**	
Empirical approach	descriptive
Use of exposure variation	only variation over time for multiple populations
Method	non-mechanistic model (correlation)
Code availability	none
**Effectiveness assessment**	
Reporting of intervention effectiveness	quantification of change in outcome values
Measure of effectiveness	cases
Interpretation of results	implicitly causal

– continued on next page –

Reporting of uncertainty	yes
Sensitivity analysis	none
Subgroup assessment	none

**Wong et al. 2020a**	
**Study information**	
Author	Wong CK, Wong JY, Tang EH, Au CH, Lau KT, Wai AK
Title	Impact of National Containment Measures on Decelerating the Increase in Daily New Cases of COVID-19 in 54 Countries and 4 Epicenters of the Pandemic: Comparative Observational Study
Year	2020
Journal	Journal of Medical Internet Research
DOI	10.2196/19904
Date received	2020-05-06
Date accepted	2020-07-13
**Study setting**	
Number of populations included	multiple
Level of populations included	both national and subnational (54 countries, Wuhan, New York State, Lombardy, Madrid)
Geographic areas covered	Europe, Middle East and Africa, Asia, Oceania, Central and South America, North America
Number of countries covered	multiple countries
Study period	same end date for several populations with diverse epidemic trajectories
**Outcome**	
Raw outcome	epidemiological population-level outcome (cases)

– continued on next page –

Time resolution of raw outcome	daily
Computed outcome	none
Method to obtain the computed outcome	– not applicable –
Data source	data from publicly available cross-country selections (OurWorldInData)
Data availability	data access via source
**Intervention**	
Terminology for interventions	measures
Terminology for the specific type of non-pharmaceutical interventions	containment
Exposure types	multiple separate interventions (stay-at-home order (limited outdoor movement), curfew (stay-at-home order during specific time periods), lockdown (restriction of population mobility))
Types of single interventions	stay-at-home order, internal movement restrictions
Coding of interventions	necessary
Source of intervention data	coding done by authors
Availability of data on exposure	coded data made available by the authors
**Methodological approach**	
Empirical approach	descriptive
Use of exposure variation	only variation over time for multiple populations
Method	description of change over time (description of time course)
Code availability	none
**Effectiveness assessment**	
Reporting of intervention effectiveness	comparison of outcome values
Measure of effectiveness	cases

– continued on next page –

Interpretation of results	implicitly causal
Reporting of uncertainty	yes
Sensitivity analysis	none
Subgroup assessment	geographic areas (regions), socioeconomic indicators (income), epidemiological indicators (pre-intervention epidemic trend)

Wong et al. 2020b (Analysis 1)

**Study information**	
Author	Wong GN, Weiner ZJ, Tkachenko AV, Elbanna A, Maslov S, Goldenfeld N
Title	Modeling COVID-19 Dynamics in Illinois under Nonpharmaceutical Interventions
Year	2020
Journal	Physical Review X
DOI	10.1103/physrevx.10.041033
Date received	2020-06-15
Date accepted	2020-10-22
**Study setting**	
Number of populations included	single
Level of populations included	subnational (Illinois)
Geographic areas covered	North America
Number of countries covered	single country (US)
Study period	end date at peak of wave
**Outcome**	

– continued on next page –

Raw outcome	epidemiological population-level outcome (hospitalizations, ICU hospitalizations, deaths)
Time resolution of raw outcome	daily
Computed outcome	none
Method to obtain the computed outcome	– not applicable –
Data source	data from (sub)national authorities
Data availability	data made available by the authors
**Intervention**	
Terminology for interventions	interventions
Terminology for the specific type of non-pharmaceutical interventions	non-pharmaceutical
Exposure types	all interventions together
Types of single interventions	– not applicable –
Coding of interventions	– not applicable –
Source of intervention data	– not applicable –
Availability of data on exposure	– not applicable –
**Methodological approach**	
Empirical approach	parametric
Use of exposure variation	only variation over time for a single population
Method	mechanistic model (compartmental single-population transmission model)
Latent variable functionally linked to intervention	reproduction number
Code availability	publicly available
**Effectiveness assessment**	

– continued on next page –

Reporting of intervention effectiveness	quantification of change in outcome values
Measure of effectiveness	reproduction number
Interpretation of results	implicitly causal
Reporting of uncertainty	yes
Sensitivity analysis	none
Subgroup assessment	geographic areas (regions)

**Wong et al. 2020b (Analysis 2, mobility as exposure)**	
**Study information**	
Author	Wong GN, Weiner ZJ, Tkachenko AV, Elbanna A, Maslov S, Goldenfeld N
Title	Modeling COVID-19 Dynamics in Illinois under Nonpharmaceutical Interventions
Year	2020
Journal	Physical Review X
DOI	10.1103/physrevx.10.041033
Date received	15.06.2020
Date accepted	22.10.2020
**Study setting**	
Number of populations included	single
Level of populations included	subnational (Illinois)
Geographic areas covered	North America
Number of countries covered	single country (US)
Study period	end date at peak of wave
**Outcome**	

– continued on next page –

Raw outcome	epidemiological population-level outcome (hospitalizations, ICU hospitalizations, deaths)
Time resolution of raw outcome	daily
Computed outcome	other (mitigation factor)
Method to obtain the computed outcome	compartmental transmission model (single-population)
Data source	data from (sub)national authorities
Data availability	data made available by the authors
**Intervention**	
Terminology for interventions	interventions
Terminology for the specific type of non-pharmaceutical interventions	non-pharmaceutical
Exposure types	mobility (activity, distance, non-essential visits)
Types of single interventions	– not applicable –
Coding of interventions	– not applicable –
Source of intervention data	mobility data by corporate organizations (Google, Unacast)
Availability of data on exposure	data access via source
**Methodological approach**	
Empirical approach	descriptive
Use of exposure variation	only variation over time for a single population
Method	description of change over time (description of time course)
Code availability	publicly available
**Effectiveness assessment**	
Reporting of intervention effectiveness	qualitative statement
Measure of effectiveness	mitigation factor

– continued on next page –

Interpretation of results	implicitly causal
Reporting of uncertainty	– not applicable –
Sensitivity analysis	none
Subgroup assessment	none

Wong et al. 2020c (Analysis 1)

**Study information**	
Author	Wong MC, Ng RW, Chong KC, Lai CK, Huang J, Chen Z, Boon SS, Chan PK
Title	Stringent Containment Measures without Complete City Lockdown to Achieve Low Incidence and Mortality across Two Waves of COVID-19 in Hong Kong
Year	2020
Journal	BMJ Global Health
DOI	10.1136/bmjgh-2020-003573
Date received	2020-07-29
Date accepted	2020-09-07
**Study setting**	
Number of populations included	single
Level of populations included	national (Hong Kong)
Geographic areas covered	Asia
Number of countries covered	single country (Hong Kong)
Study period	includes second wave (two waves)
**Outcome**	

– continued on next page –

Raw outcome	epidemiological individual-level outcome (individual cases)
Time resolution of raw outcome	daily
Computed outcome	epidemiological parameter (reproduction number)
Method to obtain the computed outcome	statistical estimation of reproduction number (Thompson et al.)
Data source	data from (sub)national authorities
Data availability	data not accessible
**Intervention**	
Terminology for interventions	measures
Terminology for the specific type of non-pharmaceutical interventions	containment
Exposure types	all interventions together (timeline of measures)
Types of single interventions	– not applicable –
Coding of interventions	– not applicable –
Source of intervention data	– not applicable –
Availability of data on exposure	– not applicable –
**Methodological approach**	
Empirical approach	descriptive
Use of exposure variation	only variation over time for a single population
Method	description of change over time (description of time course)
Code availability	none
**Effectiveness assessment**	
Reporting of intervention effectiveness	comparison of outcome values
Measure of effectiveness	reproduction number
Interpretation of results	associative

– continued on next page –

Reporting of uncertainty	yes
Sensitivity analysis	none
Subgroup assessment	none

**Wong et al. 2020c (Analysis 2)**	
**Study information**	
Author	Wong MC, Ng RW, Chong KC, Lai CK, Huang J, Chen Z, Boon SS, Chan PK
Title	Stringent Containment Measures without Complete City Lockdown to Achieve Low Incidence and Mortality across Two Waves of COVID-19 in Hong Kong
Year	2020
Journal	BMJ Global Health
DOI	10.1136/bmjgh-2020-003573
Date received	2020-07-29
Date accepted	2020-09-07
**Study setting**	
Number of populations included	single
Level of populations included	national (Hong Kong)
Geographic areas covered	Asia
Number of countries covered	single country (Hong Kong)
Study period	includes second wave (two waves)
**Outcome**	
Raw outcome	epidemiological population-level outcome (cases)
Time resolution of raw outcome	daily

– continued on next page –

Computed outcome	none
Method to obtain the computed outcome	– not applicable –
Data source	data from (sub)national authorities
Data availability	data access via source
**Intervention**	
Terminology for interventions	measures
Terminology for the specific type of non-pharmaceutical interventions	containment
Exposure types	all interventions together (timeline of measures)
Types of single interventions	– not applicable –
Coding of interventions	– not applicable –
Source of intervention data	– not applicable –
Availability of data on exposure	– not applicable –
**Methodological approach**	
Empirical approach	counterfactual
Use of exposure variation	only variation over time for a single population
Method	other (extrapolation of cases by forward application of renewal equation used by Thompson et al.)
Code availability	none
**Effectiveness assessment**	
Reporting of intervention effectiveness	qualitative statement
Measure of effectiveness	cases
Interpretation of results	associative
Reporting of uncertainty	– not applicable –

– continued on next page –

Sensitivity analysis	none
Subgroup assessment	none

Wu et al. 2020 (Analysis 1)

**Study information**	
Author	Wu J, Tang B, Bragazzi NL, Nah K, McCarthy Z
Title	Quantifying the Role of Social Distancing, Personal Protection and Case Detection in Mitigating COVID-19 Outbreak in Ontario, Canada
Year	2020
Journal	Journal of Mathematics in Industry
DOI	10.1186/s13362-020-00083-3
Date received	2020-04-24
Date accepted	2020-05-12
**Study setting**	
Number of populations included	single
Level of populations included	subnational (Ontario)
Geographic areas covered	North America
Number of countries covered	single country (Canada)
Study period	end date in growth phase of wave
**Outcome**	
Raw outcome	epidemiological population-level outcome (cases)
Time resolution of raw outcome	daily
Computed outcome	epidemiological parameter (reproduction number)

– continued on next page –

Method to obtain the computed outcome	compartmental transmission model (single-population)
Data source	data from (sub)national authorities
Data availability	data access via source
**Intervention**	
Terminology for interventions	interventions
Terminology for the specific type of non-pharmaceutical interventions	public health
Exposure types	all interventions together
Types of single interventions	– not applicable –
Coding of interventions	– not applicable –
Source of intervention data	– not applicable –
Availability of data on exposure	– not applicable –
**Methodological approach**	
Empirical approach	descriptive
Use of exposure variation	only variation over time for a single population
Method	description of change over time (description of time course)
Code availability	none
**Effectiveness assessment**	
Reporting of intervention effectiveness	qualitative statement
Measure of effectiveness	reproduction number
Interpretation of results	implicitly causal
Reporting of uncertainty	– not applicable –
Sensitivity analysis	none
Subgroup assessment	none

Wu et al. 2020 (Analysis 2)	
**Study information**	
Author	Wu J, Tang B, Bragazzi NL, Nah K, McCarthy Z
Title	Quantifying the Role of Social Distancing, Personal Protection and Case Detection in Mitigating COVID-19 Outbreak in Ontario, Canada
Year	2020
Journal	Journal of Mathematics in Industry
DOI	10.1186/s13362-020-00083-3
Date received	2020-04-24
Date accepted	2020-05-12
**Study setting**	
Number of populations included	single
Level of populations included	subnational (Ontario)
Geographic areas covered	North America
Number of countries covered	single country (Canada)
Study period	end date in growth phase of wave
**Outcome**	
Raw outcome	epidemiological population-level outcome (cases)
Time resolution of raw outcome	daily
Computed outcome	none
Method to obtain the computed outcome	– not applicable –
Data source	data from (sub)national authorities
Data availability	data access via source
**Intervention**	
Terminology for interventions	interventions

– continued on next page –

Terminology for the specific type of non-pharmaceutical interventions	public health
Exposure types	one single intervention (workplace closure on March 24)
Types of single interventions	workplace closure
Coding of interventions	not necessary
Source of intervention data	– could not be evaluated –
Availability of data on exposure	raw data documented in the manuscript
**Methodological approach**	
Empirical approach	parametric
Use of exposure variation	only variation over time for a single population
Method	mechanistic model (compartmental single-population transmission model)
Latent variable functionally linked to intervention	contact rate, quarantine rate
Code availability	none
**Effectiveness assessment**	
Reporting of intervention effectiveness	quantification of change in outcome values
Measure of effectiveness	contact rate, quarantine rate, reproduction number
Interpretation of results	implicitly causal
Reporting of uncertainty	no
Sensitivity analysis	none
Subgroup assessment	none

Xia et al. 2020	
**Study information**	
Author	Xia F, Xiao AY, Liu P, Cheke RA, Li X, And
Title	Differences in How Interventions Coupled with Effective Reproduction Numbers Account for Marked Variations in COVID-19 Epidemic Outcomes
Year	2020
Journal	Mathematical Biosciences and Engineering
DOI	10.3934/mbe.2020274
Date received	2020-06-05
Date accepted	2020-07-21
**Study setting**	
Number of populations included	multiple
Level of populations included	national (China, South Korea, Japan, Spain)
Geographic areas covered	Asia, Europe
Number of countries covered	multiple countries
Study period	start and end date span first epidemic wave
**Outcome**	
Raw outcome	epidemiological population-level outcome (cases)
Time resolution of raw outcome	daily
Computed outcome	epidemiological parameter (reproduction number)
Method to obtain the computed outcome	compartmental transmission model (single-population)
Data source	data from (sub)national authorities (health authority in each country), data from publicly available cross-country selections (WHO: all countries)
Data availability	data access via source
**Intervention**	
Terminology for interventions	strategies

– continued on next page –

Terminology for the specific type of non-pharmaceutical interventions	containment
Exposure types	all interventions together
Types of single interventions	– not applicable –
Coding of interventions	– not applicable –
Source of intervention data	– not applicable –
Availability of data on exposure	– not applicable –
**Methodological approach**	
Empirical approach	descriptive
Use of exposure variation	only variation over time for multiple populations
Method	description of change over time (description of time course)
Code availability	none
**Effectiveness assessment**	
Reporting of intervention effectiveness	qualitative statement
Measure of effectiveness	reproduction number
Interpretation of results	implicitly causal
Reporting of uncertainty	– not applicable –
Sensitivity analysis	none
Subgroup assessment	none

Xiao et al. 2020

**Study information**	
Author	Xiao Y, Tang B, Wu J, Cheke RA, Tang S

– continued on next page –

Title	Linking Key Intervention Timing to Rapid Decline of the COVID-19 Effective Reproductive Number to Quantify Lessons from Mainland China
Year	2020
Journal	International Journal of Infectious Diseases
DOI	10.1016/j.ijid.2020.06.030
Date received	2020-05-08
Date accepted	2020-06-04
**Study setting**	
Number of populations included	single
Level of populations included	national (China)
Geographic areas covered	Asia
Number of countries covered	single country (China)
Study period	start and end date span first epidemic wave
**Outcome**	
Raw outcome	epidemiological population-level outcome (cases)
Time resolution of raw outcome	daily
Computed outcome	epidemiological parameter (reproduction number)
Method to obtain the computed outcome	statistical estimation of reproduction number (– could not be evaluated –)
Data source	– could not be evaluated –
Data availability	data not accessible
**Intervention**	
Terminology for interventions	measures
Terminology for the specific type of non-pharmaceutical interventions	intervention
Exposure types	all interventions together (timeline of measures)

– continued on next page –

Types of single interventions	– not applicable –
Coding of interventions	– not applicable –
Source of intervention data	– not applicable –
Availability of data on exposure	– not applicable –
**Methodological approach**	
Empirical approach	descriptive
Use of exposure variation	only variation over time for a single population
Method	description of change over time (description of time course)
Code availability	none
**Effectiveness assessment**	
Reporting of intervention effectiveness	qualitative statement
Measure of effectiveness	reproduction number
Interpretation of results	associative
Reporting of uncertainty	– not applicable –
Sensitivity analysis	none
Subgroup assessment	none

Xu et al. 2020 (Analysis 1)

**Study information**	
Author	Xu P, Dredze M, Broniatowski DA
Title	The Twitter Social Mobility Index: Measuring Social Distancing Practices with Geolocated Tweets

– continued on next page –

Year	2020
Journal	Journal of Medical Internet Research
DOI	10.2196/21499
Date received	2020-06-16
Date accepted	2020-10-11
**Study setting**	
Number of populations included	multiple
Level of populations included	both national and subnational (US)
Geographic areas covered	North America
Number of countries covered	single country (US)
Study period	start and end date span first epidemic wave: behavioral
**Outcome**	
Raw outcome	behavioral outcome (mobility: area and frequency of travel)
Time resolution of raw outcome	daily
Computed outcome	none
Method to obtain the computed outcome	– not applicable –
Data source	mobility data from corporate organizations (Twitter)
Data availability	data not accessible
**Intervention**	
Terminology for interventions	policies
Terminology for the specific type of non-pharmaceutical interventions	social distancing
Exposure types	one combination of interventions (social distancing measures, "slow the spread guidelines" by White house)
Types of single interventions	– not applicable –

– continued on next page –

Coding of interventions	not necessary
Source of intervention data	government or news websites
Availability of data on exposure	raw data documented in the manuscript
**Methodological approach**	
Empirical approach	descriptive
Use of exposure variation	only variation over time for multiple populations
Method	description of change over time (comparison of time periods)
Code availability	none
**Effectiveness assessment**	
Reporting of intervention effectiveness	quantification of change in outcome values
Measure of effectiveness	mobility: area and frequency of travel
Interpretation of results	associative
Reporting of uncertainty	no
Sensitivity analysis	none
Subgroup assessment	none

Xu et al. 2020 (Analysis 2)

**Study information**	
Author	Xu P, Dredze M, Broniatowski DA
Title	The Twitter Social Mobility Index: Measuring Social Distancing Practices with Geolocated Tweets
Year	2020
Journal	Journal of Medical Internet Research
DOI	10.2196/21499

– continued on next page –

Date received	2020-06-16
Date accepted	2020-10-11
**Study setting**	
Number of populations included	multiple
Level of populations included	subnational (US)
Geographic areas covered	North America
Number of countries covered	single country (US)
Study period	same end date for several populations with diverse epidemic trajectories
**Outcome**	
Raw outcome	epidemiological population-level outcome (cases)
Time resolution of raw outcome	daily
Computed outcome	none
Method to obtain the computed outcome	– not applicable –
Data source	data from publicly available cross-country selections (JHU)
Data availability	data made available by the authors
**Intervention**	
Terminology for interventions	policies
Terminology for the specific type of non-pharmaceutical interventions	social distancing
Exposure types	multiple separate interventions (state of emergency declaration, stay-at-home order, business closure, and venue closure (in various forms), other (date banned visitors to nursing homes, evictions frozen, day care closed))
Types of single interventions	declaration of a state of emergency, stay-at-home order, workplace closure, venue closure, other
Coding of interventions	necessary

– continued on next page –

**Xu et al. 2020 (Analysis 2)** – continued from previous page	
Source of intervention data	use of externally coded data (COVID-19 US State Policy Database openICPSR)
Availability of data on exposure	coded data made available by the authors
**Methodological approach**	
Empirical approach	parametric
Use of exposure variation	only variation between populations
Method	non-mechanistic model (correlation)
Code availability	none
**Effectiveness assessment**	
Reporting of intervention effectiveness	quantification of change in outcome values
Measure of effectiveness	cases
Interpretation of results	associative
Reporting of uncertainty	yes
Sensitivity analysis	none
Subgroup assessment	none

**Xu et al. 2020 (Analysis 3, mobility as exposure)**	
**Study information**	
Author	Xu P, Dredze M, Broniatowski DA
Title	The Twitter Social Mobility Index: Measuring Social Distancing Practices with Geolocated Tweets
Year	2020
Journal	Journal of Medical Internet Research
DOI	10.2196/21499

– continued on next page –

Date received	16.06.2020
Date accepted	11.10.2020
**Study setting**	
Number of populations included	multiple
Level of populations included	both national and subnational (US)
Geographic areas covered	North America
Number of countries covered	single country (US)
Study period	same end date for several populations with diverse epidemic trajectories
**Outcome**	
Raw outcome	epidemiological population-level outcome (cases)
Time resolution of raw outcome	daily
Computed outcome	none
Method to obtain the computed outcome	– not applicable –
Data source	data from publicly available cross-country selections (JHU)
Data availability	data made available by the authors
**Intervention**	
Terminology for interventions	policies
Terminology for the specific type of non-pharmaceutical interventions	social distancing
Exposure types	mobility (area and frequency of travel)
Types of single interventions	– not applicable –
Coding of interventions	– not applicable –
Source of intervention data	mobility data by corporate organizations (Twitter)
Availability of data on exposure	data not accessible
**Methodological approach**	

– continued on next page –

Empirical approach	descriptive
Use of exposure variation	both variation over time and between populations
Method	non-mechanistic model (correlation)
Code availability	none
**Effectiveness assessment**	
Reporting of intervention effectiveness	quantification of change in outcome values
Measure of effectiveness	cases
Interpretation of results	associative
Reporting of uncertainty	no
Sensitivity analysis	none
Subgroup assessment	none

Yang et al. 2020

**Study information**	
Author	Yang Q, Yi C, Vajdi A, Cohnstaedt LW, Wu H, Guo X, Scoglio CM
Title	Short-Term Forecasts and Long-Term Mitigation Evaluations for the COVID-19 Epidemic in Hubei Province, China
Year	2020
Journal	Infectious Disease Modelling
DOI	10.1016/j.idm.2020.08.001
Date received	2020-03-27
Date accepted	2020-07-05
**Study setting**	

– continued on next page –

Number of populations included	single
Level of populations included	subnational (Hubei province)
Geographic areas covered	Asia
Number of countries covered	single country (China)
Study period	start and end date span first epidemic wave
**Outcome**	
Raw outcome	epidemiological population-level outcome (cases)
Time resolution of raw outcome	daily
Computed outcome	epidemiological parameter (reproduction number, transmission rate)
Method to obtain the computed outcome	compartmental transmission model (metapopulation)
Data source	data from (sub)national authorities
Data availability	data access via source
**Intervention**	
Terminology for interventions	interventions
Terminology for the specific type of non-pharmaceutical interventions	non-pharmaceutical
Exposure types	one combination of interventions (Wuhan City Lockdown)
Types of single interventions	– not applicable –
Coding of interventions	not necessary
Source of intervention data	another study (Du et al., Wu et al.)
Availability of data on exposure	raw data documented in the manuscript
**Methodological approach**	
Empirical approach	descriptive
Use of exposure variation	only variation over time for a single population
Method	description of change over time (comparison of time periods)

– continued on next page –

Code availability	none
**Effectiveness assessment**	
Reporting of intervention effectiveness	comparison of outcome values
Measure of effectiveness	transmission rate, reproduction number
Interpretation of results	associative
Reporting of uncertainty	no
Sensitivity analysis	none
Subgroup assessment	none

Ye & Hu 2020

**Study information**	
Author	Ye L, Hu L
Title	Spatiotemporal Distribution and Trend of COVID-19 in the Yangtze River Delta Region of the People's Republic of China
Year	2020
Journal	Geospatial Health
DOI	10.4081/gh.2020.889
Date received	2020-04-11
Date accepted	2020-04-30
**Study setting**	
Number of populations included	multiple
Level of populations included	subnational (Yangtze river delta region (China) + 3 subregions)
Geographic areas covered	Asia

– continued on next page –

Number of countries covered	single country (China)
Study period	start and end date span first epidemic wave
**Outcome**	
Raw outcome	epidemiological population-level outcome (cases)
Time resolution of raw outcome	daily
Computed outcome	none
Method to obtain the computed outcome	– not applicable –
Data source	data from (sub)national authorities
Data availability	data access via source
**Intervention**	
Terminology for interventions	measures
Terminology for the specific type of non-pharmaceutical interventions	control
Exposure types	one combination of interventions (Wuhan City Lockdown)
Types of single interventions	– not applicable –
Coding of interventions	not necessary
Source of intervention data	– could not be evaluated –
Availability of data on exposure	raw data documented in the manuscript
**Methodological approach**	
Empirical approach	descriptive
Use of exposure variation	only variation over time for multiple populations
Method	description of change over time (description of time course)
Code availability	none
**Effectiveness assessment**	

– continued on next page –

Reporting of intervention effectiveness	qualitative statement
Measure of effectiveness	cases
Interpretation of results	implicitly causal
Reporting of uncertainty	– not applicable –
Sensitivity analysis	none
Subgroup assessment	none

Yehya et al. 2020

**Study information**	
Author	Yehya N, Venkataramani A, Harhay MO
Title	Statewide Interventions and Coronavirus Disease 2019 Mortality in the United States: An Observational Study
Year	2020
Journal	Clinical Infectious Diseases
DOI	10.1093/cid/ciaa923
Date received	2020-05-22
Date accepted	2020-06-26
**Study setting**	
Number of populations included	multiple
Level of populations included	both national and subnational (37 US states)
Geographic areas covered	North America
Number of countries covered	single country (US)
Study period	same end date for several populations with diverse epidemic trajectories

– continued on next page –

**Outcome**	
Raw outcome	epidemiological population-level outcome (deaths)
Time resolution of raw outcome	daily
Computed outcome	summary statistic (mortality rate ratio: mortality rate relative to mortality rate on day 1 in a country)
Method to obtain the computed outcome	simple computation
Data source	data from publicly available cross-country selections (JHU)
Data availability	data access via source
**Intervention**	
Terminology for interventions	measures
Terminology for the specific type of non-pharmaceutical interventions	social distancing
Exposure types	multiple separate interventions (emergency declaration, school closure)
Types of single interventions	declaration of a state of emergency, school closure
Coding of interventions	necessary
Source of intervention data	coding done by authors
Availability of data on exposure	coded data not available
**Methodological approach**	
Empirical approach	parametric
Use of exposure variation	both variation over time and between populations
Method	non-mechanistic model (generalized linear model)
Code availability	none
**Effectiveness assessment**	
Reporting of intervention effectiveness	quantification of change in outcome values

– continued on next page –

Measure of effectiveness	mortality rate ratio: mortality rate relative to mortality rate on day 1 in a country
Interpretation of results	associative
Reporting of uncertainty	yes
Sensitivity analysis	different or modified outcome used (per capita deaths used rather than absolute deaths), start or end date of study period varied, same analysis with (sub)population(s) excluded
Subgroup assessment	none

**You 2020**	
**Study information**	
Author	You JH
Title	Impact of COVID-19 Infection Control Measures on Influenza-Related Outcomes in Hong Kong
Year	2020
Journal	Pathogens and Global Health
DOI	10.1080/20477724.2020.1857492
Date received	– could not be evaluated –
Date accepted	2020-12-15 (published)
**Study setting**	
Number of populations included	single
Level of populations included	national (Hong Kong)
Geographic areas covered	Asia
Number of countries covered	single country (Hong Kong)
Study period	end date could not be evaluated

– continued on next page –

**Outcome**	
Raw outcome	epidemiological population-level outcome (surrogate disease: hospitalizations influenza)
Time resolution of raw outcome	weekly
Computed outcome	other (cases: influenza, deaths: influenza, clinical visits: influenza)
Method to obtain the computed outcome	other (multiply observed cases with ratios for clinical visits, deaths, hospitalizations, perform Monte Carlo simulation)
Data source	data from (sub)national authorities
Data availability	data access via source
**Intervention**	
Terminology for interventions	measures
Terminology for the specific type of non-pharmaceutical interventions	infection control
Exposure types	all interventions together
Types of single interventions	– not applicable –
Coding of interventions	– not applicable –
Source of intervention data	– not applicable –
Availability of data on exposure	– not applicable –
**Methodological approach**	
Empirical approach	descriptive
Use of exposure variation	only variation over time for a single population
Method	description of change over time (comparison of time periods)
Code availability	none
**Effectiveness assessment**	
Reporting of intervention effectiveness	quantification of change in outcome values

– continued on next page –

Measure of effectiveness	hospitalizations: influenza
Interpretation of results	associative
Reporting of uncertainty	yes
Sensitivity analysis	none
Subgroup assessment	none

**You et al. 2020**	
**Study information**	
Author	You C, Deng Y, Hu W, Sun J, Lin Q, Zhou F, Pang CH, Zhang Y, Chen Z, Zhou XH
Title	Estimation of the Time-Varying Reproduction Number of COVID-19 Outbreak in China
Year	2020
Journal	International Journal of Hygiene and Environmental Health
DOI	10.1016/j.ijheh.2020.113555
Date received	2020-03-10
Date accepted	2020-05-07
**Study setting**	
Number of populations included	multiple
Level of populations included	both national and subnational (Hubei, Beijing, Shanghai, Guangdong, Zhejiang, and Henan (cities/provinces))
Geographic areas covered	Asia
Number of countries covered	single country (China)
Study period	start and end date span first epidemic wave
**Outcome**	

– continued on next page –

Raw outcome	epidemiological individual-level outcome (individual cases and transmission chains)
Time resolution of raw outcome	daily
Computed outcome	epidemiological parameter (reproduction number)
Method to obtain the computed outcome	statistical estimation of reproduction number (White et al., Walinga et al.), compartmental transmission model (single-population)
Data source	data from (sub)national authorities
Data availability	data not accessible
**Intervention**	
Terminology for interventions	measures
Terminology for the specific type of non-pharmaceutical interventions	containment
Exposure types	one combination of interventions (Wuhan City Lockdown)
Types of single interventions	– not applicable –
Coding of interventions	not necessary
Source of intervention data	– could not be evaluated –
Availability of data on exposure	raw data documented in the manuscript
**Methodological approach**	
Empirical approach	descriptive
Use of exposure variation	only variation over time for multiple populations
Method	description of change over time (comparison of time periods)
Code availability	none
**Effectiveness assessment**	
Reporting of intervention effectiveness	comparison of outcome values
Measure of effectiveness	reproduction number

– continued on next page –

Interpretation of results	implicitly causal
Reporting of uncertainty	yes
Sensitivity analysis	none
Subgroup assessment	none

**Yuan et al. 2020**	
**Study information**	
Author	Yuan Z, Xiao Y, Dai Z, Huang J, Zhang Z, Chen Y
Title	Modelling the Effects of Wuhan's Lockdown during COVID-19, China
Year	2020
Journal	Bulletin of the World Health Organization
DOI	10.2471/blt.20.254045
Date received	2020-02-29
Date accepted	2020-05-01
**Study setting**	
Number of populations included	multiple
Level of populations included	both national and subnational (China + 44 non-Wuhan provinces, cities)
Geographic areas covered	Asia
Number of countries covered	single country (China)
Study period	start and end date span first epidemic wave
**Outcome**	
Raw outcome	epidemiological population-level outcome (cases)
Time resolution of raw outcome	– not applicable –
Computed outcome	none

– continued on next page –

Method to obtain the computed outcome	– not applicable –
Data source	data from (sub)national authorities
Data availability	data made available by the authors
**Intervention**	
Terminology for interventions	measures
Terminology for the specific type of non-pharmaceutical interventions	control
Exposure types	all interventions together
Types of single interventions	– not applicable –
Coding of interventions	– not applicable –
Source of intervention data	– not applicable –
Availability of data on exposure	– not applicable –
**Methodological approach**	
Empirical approach	counterfactual
Use of exposure variation	both variation over time and between populations
Method	non-mechanistic model (generalized linear model)
Code availability	none
**Effectiveness assessment**	
Reporting of intervention effectiveness	comparison of outcome values
Measure of effectiveness	cases
Interpretation of results	implicitly causal
Reporting of uncertainty	no
Sensitivity analysis	none
Subgroup assessment	none

Zhang et al. 2020a	
**Study information**	
Author	Zhang X, Ji Z, Zheng Y, Ye X, Li D
Title	Evaluating the Effect of City Lockdown on Controlling COVID-19 Propagation through Deep Learning and Network Science Models
Year	2020
Journal	Cities
DOI	10.1016/j.cities.2020.102869
Date received	2020-05-08
Date accepted	2020-07-07
**Study setting**	
Number of populations included	multiple
Level of populations included	both national and subnational (China and its provinces)
Geographic areas covered	Asia
Number of countries covered	single country (China)
Study period	start and end date span first epidemic wave
**Outcome**	
Raw outcome	epidemiological population-level outcome (cases), behavioral outcome (mobility: population flow)
Time resolution of raw outcome	daily
Computed outcome	other (correlation between mobility and infection rate between provinces)
Method to obtain the computed outcome	compartmental transmission model (metapopulation)
Data source	data from publicly available cross-country selections (WHO: cases), data from (sub)national authorities (Civil Aviation Administration of China: flight data)

– continued on next page –

Data availability	data access via source
**Intervention**	
Terminology for interventions	– not applicable –
Terminology for the specific type of non-pharmaceutical interventions	lockdown
Exposure types	one combination of interventions (Wuhan City Lockdown)
Types of single interventions	– not applicable –
Coding of interventions	not necessary
Source of intervention data	– could not be evaluated –
Availability of data on exposure	raw data documented in the manuscript
**Methodological approach**	
Empirical approach	descriptive
Use of exposure variation	both variation over time and between populations
Method	description of change over time (comparison of time periods)
Code availability	none
**Effectiveness assessment**	
Reporting of intervention effectiveness	qualitative statement
Measure of effectiveness	correlation between mobility and infection rate between provinces
Interpretation of results	implicitly causal
Reporting of uncertainty	– not applicable –
Sensitivity analysis	none
Subgroup assessment	none

Zhang et al. 2020b	
**Study information**	
Author	Zhang L, Yang H, Wang K, Zhan Y, Bian L
Title	Measuring Imported Case Risk of COVID-19 from Inbound International Flights —A Case Study on China
Year	2020
Journal	Journal of Air Transport Management
DOI	10.1016/j.jairtraman.2020.101918
Date received	2020-05-29
Date accepted	2020-08-23
**Study setting**	
Number of populations included	multiple
Level of populations included	both national and subnational (China and its provinces)
Geographic areas covered	Asia
Number of countries covered	single country (China)
Study period	start and end date span first epidemic wave
**Outcome**	
Raw outcome	epidemiological population-level outcome (cases, mobility: international flight data)
Time resolution of raw outcome	daily
Computed outcome	other (imported case risk index)
Method to obtain the computed outcome	other (ODE model)
Data source	data from publicly available cross-country selections (JHU: cases), mobility data from corporate organizations (UMETRIP: mobility)
Data availability	data access via source
**Intervention**	

– continued on next page –

Terminology for interventions	– not applicable –
Terminology for the specific type of non-pharmaceutical interventions	travel ban
Exposure types	one single intervention (international travel restrictions)
Types of single interventions	international travel restrictions
Coding of interventions	not necessary
Source of intervention data	– could not be evaluated –
Availability of data on exposure	raw data documented in the manuscript
**Methodological approach**	
Empirical approach	counterfactual
Use of exposure variation	only variation over time for multiple populations
Method	other (simulation of risk index using mobility before intervention)
Code availability	none
**Effectiveness assessment**	
Reporting of intervention effectiveness	comparison of outcome values
Measure of effectiveness	imported case risk index
Interpretation of results	implicitly causal
Reporting of uncertainty	no
Sensitivity analysis	none
Subgroup assessment	none

Zhang et al. 2020c

Study information

– continued on next page –

Author	Zhang J, Dong L, Zhang Y, Chen X, Yao G, Han Z
Title	Investigating Time, Strength, and Duration of Measures in Controlling the Spread of COVID-19 Using a Networked Meta-Population Model
Year	2020
Journal	Nonlinear Dynamics
DOI	10.1007/s11071-020-05769-2
Date received	2020-04-16
Date accepted	2020-06-14
**Study setting**	
Number of populations included	multiple
Level of populations included	both national and subnational (China and some of its large cities)
Geographic areas covered	Asia
Number of countries covered	single country (China)
Study period	start and end date span first epidemic wave
**Outcome**	
Raw outcome	epidemiological population-level outcome (cases)
Time resolution of raw outcome	daily
Computed outcome	none
Method to obtain the computed outcome	– not applicable –
Data source	data from (sub)national authorities
Data availability	data made available by the authors
**Intervention**	
Terminology for interventions	measures
Terminology for the specific type of non-pharmaceutical interventions	intervention

– continued on next page –

Zhang et al. 2020c – continued from previous page

Exposure types	one combination of interventions (Wuhan City Lockdown)
Types of single interventions	– not applicable –
Coding of interventions	not necessary
Source of intervention data	government or news websites
Availability of data on exposure	raw data documented in the manuscript
**Methodological approach**	
Empirical approach	counterfactual
Use of exposure variation	both variation over time and between populations
Method	mechanistic model (compartmental metapopulation transmission model)
Code availability	publicly available
**Effectiveness assessment**	
Reporting of intervention effectiveness	comparison of outcome values
Measure of effectiveness	infections
Interpretation of results	implicitly causal
Reporting of uncertainty	yes
Sensitivity analysis	none
Subgroup assessment	none

Zhang et al. 2020d

**Study information**	
Author	Zhang B, Zhou H, Zhou F
Title	Study on SARS-CoV-2 Transmission and the Effects of Control Measures in China

– continued on next page –

Year	2020
Journal	PLOS ONE
DOI	10.1371/journal.pone.0242649
Date received	2020-02-18
Date accepted	2020-10-16
**Study setting**	
Number of populations included	multiple
Level of populations included	both national and subnational (China, Wuhan, Hubei)
Geographic areas covered	Asia
Number of countries covered	single country (China)
Study period	start and end date span first epidemic wave
**Outcome**	
Raw outcome	epidemiological population-level outcome (cases, deaths)
Time resolution of raw outcome	daily
Computed outcome	epidemiological parameter (reproduction number)
Method to obtain the computed outcome	compartmental transmission model (single-population)
Data source	data from (sub)national authorities
Data availability	data made available by the authors
**Intervention**	
Terminology for interventions	measures
Terminology for the specific type of non-pharmaceutical interventions	control
Exposure types	one combination of interventions (Wuhan City Lockdown)
Types of single interventions	– not applicable –
Coding of interventions	not necessary

– continued on next page –

Source of intervention data	– could not be evaluated –
Availability of data on exposure	raw data documented in the manuscript
**Methodological approach**	
Empirical approach	descriptive
Use of exposure variation	only variation over time for multiple populations
Method	description of change over time (comparison of time periods)
Code availability	none
**Effectiveness assessment**	
Reporting of intervention effectiveness	comparison of outcome values
Measure of effectiveness	reproduction number
Interpretation of results	implicitly causal
Reporting of uncertainty	no
Sensitivity analysis	none
Subgroup assessment	none

Zhao & Chen 2020

**Study information**	
Author	Zhao S, Chen H
Title	Modeling the Epidemic Dynamics and Control of COVID-19 Outbreak in China
Year	2020
Journal	Quantitative Biology
DOI	10.1007/s40484-020-0199-0

– continued on next page –

Date received	2020-02-29
Date accepted	2020-03-04
**Study setting**	
Number of populations included	multiple
Level of populations included	both national and subnational (Wuhan, rest of Hubei, rest of China, four Chinese cities)
Geographic areas covered	Asia
Number of countries covered	single country (China)
Study period	start and end date span first epidemic wave
**Outcome**	
Raw outcome	epidemiological population-level outcome (cases)
Time resolution of raw outcome	daily
Computed outcome	none
Method to obtain the computed outcome	– not applicable –
Data source	data from (sub)national authorities
Data availability	data access via source
**Intervention**	
Terminology for interventions	measures
Terminology for the specific type of non-pharmaceutical interventions	control
Exposure types	phases of interventions (three stages of measures in Wuhan/rest of China)
Types of single interventions	– not applicable –
Coding of interventions	not necessary
Source of intervention data	– could not be evaluated –
Availability of data on exposure	raw data documented in the manuscript

– continued on next page –

**Methodological approach**	
Empirical approach	counterfactual
Use of exposure variation	only variation over time for multiple populations
Method	mechanistic model (compartmental single-population transmission model)
Code availability	none
**Effectiveness assessment**	
Reporting of intervention effectiveness	comparison of outcome values
Measure of effectiveness	cases
Interpretation of results	implicitly causal
Reporting of uncertainty	no
Sensitivity analysis	model specification varied
Subgroup assessment	none

Zhao et al. 2020

**Study information**	
Author	Zhao L, Feng D, Ye RZ, Wang HT, Zhou YH, Wei JT, J. de Vlas S, Cui XM, Jia N, Yin CN, Li SX, Wang ZQ, Cao WC
Title	Outbreak of COVID-19 and SARS in Mainland China: A Comparative Study Based on National Surveillance Data
Year	2020
Journal	BMJ Open
DOI	10.1136/bmjopen-2020-043411
Date received	2020-08-04

– continued on next page –

Date accepted	2020-10-02
**Study setting**	
Number of populations included	single
Level of populations included	subnational (Hubei (China))
Geographic areas covered	Asia
Number of countries covered	single country (China)
Study period	start and end date span first epidemic wave
**Outcome**	
Raw outcome	epidemiological individual-level outcome (individual cases)
Time resolution of raw outcome	daily
Computed outcome	epidemiological parameter (reproduction number)
Method to obtain the computed outcome	statistical estimation of reproduction number (Cori et al.)
Data source	data from (sub)national authorities
Data availability	data not accessible
**Intervention**	
Terminology for interventions	measures
Terminology for the specific type of non-pharmaceutical interventions	control
Exposure types	one combination of interventions (Wuhan City Lockdown)
Types of single interventions	– not applicable –
Coding of interventions	not necessary
Source of intervention data	– could not be evaluated –
Availability of data on exposure	raw data documented in the manuscript
**Methodological approach**	
Empirical approach	descriptive

– continued on next page –

Use of exposure variation	only variation over time for a single population
Method	description of change over time (description of time course)
Code availability	none
**Effectiveness assessment**	
Reporting of intervention effectiveness	qualitative statement
Measure of effectiveness	reproduction number
Interpretation of results	associative
Reporting of uncertainty	no
Sensitivity analysis	none
Subgroup assessment	none

F Excluded studies

Through title and abstract screening, 411 studies were identified as potentially relevant and their full texts evaluated. Of these, 163 studies did not meet the eligibility criteria and were subsequently excluded. In the following, these excluded studies are listed together with the reason for exclusion.

Only simulation of NPI effects in hypothetical scenarios (105 studies) 651

Different research objective than assessment of NPI effects (30 studies) . . 676

Only assessment of effects of mobility or behavior, not of NPIs (17 studies) 683

Use of results from other study (6 studies) 688

Extraction not feasible due to quality issues (4 studies) 690

Not available in English language (1 study) 691

Only simulation of NPI effects in hypothetical scenarios

Only simulation of NPI effects in hypothetical scenarios	
Author	Aleta A, Moreno Y
Title	Evaluation of the potential incidence of COVID-19 and effectiveness of containment measures in Spain: A data-driven approach
Year	2020
Journal	BMC Medicine
DOI	10.1186/s12916-020-01619-5
Author	Alrashed S, Min-Allah N, Saxena A, Ali I, Mehmood R
Title	Impact of lockdowns on the spread of COVID-19 in Saudi Arabia
Year	2020
Journal	Informatics in Medicine Unlocked
DOI	10.1016/j.imu.2020.100420
Author	Alrasheed H, Althnian A, Kurdi H, Al-Mgren H, Alharbi S
Title	COVID-19 spread in Saudi Arabia: Modeling, simulation and analysis
Year	2020
Journal	International Journal of Environmental Research and Public Health
DOI	10.3390/ijerph17217744
Author	Arino J, Bajeux N, Portet S, Watmough J
Title	Quarantine and the risk of COVID-19 importation
Year	2020
Journal	Epidemiology and Infection
DOI	10.1017/S0950268820002988
Author	Awaidy SA, Mahomed O
Title	Impact of non-pharmaceutical interventions on the COVID-19 epidemic: A modelling study

– continued on next page –

Year	2020
Journal	SAGE Open Medicine
DOI	10.1177/2050312120979462
Author	Azanza Ricardo CL, Hernandez-Vargas EA
Title	Epidemiological Characteristics of COVID-19 in Mexico and the Potential Impact of Lifting Confinement Across Regions
Year	2020
Journal	Frontiers in Physics
DOI	10.3389/fphy.2020.573322
Author	Baek YJ, Lee T, Cho Y, Hyun JH, Kim MH, Sohn Y, Kim JH, Ahn JY, Jeong SJ, Ku NS, Yeom JS, Lee J, Choi JY
Title	A mathematical model of COVID-19 transmission in a tertiary hospital and assessment of the effects of different intervention strategies
Year	2020
Journal	PLOS ONE
DOI	10.1371/journal.pone.0241169
Author	Bemanian A, Ahn KW, O'Brien M, Rausch DJ, Weston B, Beyer KMM
Title	Investigating the Trajectory of the COVID-19 Outbreak in Milwaukee County and Projected Effects of Relaxed Distancing
Year	2020
Journal	WMJ
DOI	not available
Author	Bentout S, Tridane A, Djilali S, Touaoula TM
Title	Age-Structured Modeling of COVID-19 Epidemic in the USA, UAE and Algeria
Year	2020

– continued on next page –

Journal	Alexandria Engineering Journal
DOI	10.1016/j.aej.2020.08.053
Author	Bouchnita A, Jebrane A
Title	A hybrid multi-scale model of COVID-19 transmission dynamics to assess the potential of non-pharmaceutical interventions
Year	2020
Journal	Chaos, Solitons and Fractals
DOI	10.1016/j.chaos.2020.109941
Author	Buonomo, B., Della Marca, R.
Title	Effects of information-induced behavioural changes during the COVID-19 lockdowns: The case of Italy: COVID-19 lockdowns and behavioral change
Year	2020
Journal	Royal Society Open Science
DOI	10.1098/rsos.201635rsos201635
Author	Cabore JW, Karamagi HC, Kipruto H, Asamani JA, Droti B, Seydi ABW, Titi-Ofei R, Impouma B, Yao M, Yoti Z, Zawaira F, Tumusiime P, Talisuna A, Kasolo FC, Moeti MR
Title	The potential effects of widespread community transmission of SARS-CoV-2 infection in the World Health Organization African Region: A predictive model
Year	2020
Journal	BMJ Global Health
DOI	10.1136/bmjgh-2020-002647
Author	Cano OB, Morales SC, Bendtsen C
Title	Covid-19 modelling: The effects of social distancing
Year	2020

– continued on next page –

Journal	Interdisciplinary Perspectives on Infectious Diseases
DOI	10.1155/2020/2041743
Author	Casares M, Khan H
Title	The timing and intensity of social distancing to flatten the COVID-19 curve: The case of Spain
Year	2020
Journal	International Journal of Environmental Research and Public Health
DOI	10.3390/ijerph17197283
Author	Castilho C, Gondim JAM, Marchesin M, Sabeti M
Title	Assessing the efficiency of different control strategies for the covid-19 epidemic
Year	2020
Journal	Electronic Journal of Differential Equations
DOI	not available
Author	Chang SL, Harding N, Zachreson C, Cliff OM, Prokopenko M
Title	Modelling transmission and control of the COVID-19 pandemic in Australia
Year	2020
Journal	Nature Communications
DOI	10.1038/s41467-020-19393-6
Author	Chen S, Li Q, Gao S, Kang Y, Shi X
Title	State-specific projection of COVID-19 infection in the United States and evaluation of three major control measures
Year	2020
Journal	Scientific Reports
DOI	10.1038/s41598-020-80044-3

– continued on next page –

Author	Choi S, Ki M
Title	Estimating the reproductive number and the outbreak size of COVID-19 in Korea
Year	2020
Journal	Epidemiology and health
DOI	10.4178/epih.e2020011
Author	Costantino V, Heslop DJ, MacIntyre CR
Title	The effectiveness of full and partial travel bans against COVID-19 spread in Australia for travellers from China during and after the epidemic peak in China
Year	2020
Journal	Journal of travel medicine
DOI	10.1093/jtm/taaa081
Author	DeFries R, Agarwala M, Baquie S, Choksi P, Dogra N, Preetha GS, Khanwilkar S, Mondal P, Nagendra H, Urpelainen J
Title	Post-lockdown spread of COVID-19 from cities to vulnerable forest-fringe villages in Central India
Year	2020
Journal	Current Science
DOI	10.18520/cs/v119/i1/52-58
Author	Faranda D, Alberti T
Title	Modeling the second wave of COVID-19 infections in France and Italy via a stochastic SEIR model
Year	2020
Journal	Chaos
DOI	10.1063/5.0015943

– continued on next page –

Author	Feng LX, Jing SL, Hu SK, Wang DF, Huo HF
Title	Modelling the effects of media coverage and quarantine on the COVID-19 infections in the UK
Year	2020
Journal	Mathematical Biosciences and Engineering
DOI	10.3934/MBE.2020204
Author	Giordano G, Blanchini F, Bruno R, Colaneri P, Di Filippo A, Di Matteo A, Colaneri M
Title	Modelling the COVID-19 epidemic and implementation of population-wide interventions in Italy
Year	2020
Journal	Nature Medicine
DOI	10.1038/s41591-020-0883-7
Author	Girona T
Title	Confinement Time Required to Avoid a Quick Rebound of COVID-19: Predictions From a Monte Carlo Stochastic Model
Year	2020
Journal	Frontiers in Physics
DOI	10.3389/fphy.2020.00186
Author	Godin A, Xia Y, Buckeridge DL, Mishra S, Douwes-Schultz D, Shen Y, Lavigne M, Drolet M, Schmidt AM, Brisson M, Maheu-Giroux M
Title	The role of case importation in explaining differences in early SARS-CoV-2 transmission dynamics in Canada—A mathematical modeling study of surveillance data
Year	2020
Journal	International Journal of Infectious Diseases

– continued on next page –

DOI	10.1016/j.ijid.2020.10.046
Author	Gountas I, Hillas G, Souliotis K
Title	Act early, save lives: managing COVID-19 in Greece
Year	2020
Journal	Public Health
DOI	10.1016/j.puhe.2020.08.016
Author	Gul S, Tuncay K, Binici B, Aydin BB
Title	Transmission dynamics of Covid-19 in Italy, Germany and Turkey considering social distancing, testing and quarantine
Year	2020
Journal	Journal of Infection in Developing Countries
DOI	10.3855/jidc.12844
Author	Gupta SD, Jain R, Bhatnagar S
Title	COVID-19 Pandemic in Rajasthan: Mathematical Modelling and Social Distancing
Year	2020
Journal	Journal of Health Management
DOI	10.1177/0972063420935537
Author	Guzzetta G, Poletti P, Ajelli M, Trentini F, Marziano V, Cereda D, Tirani M, Diurno G, Bodina A, Barone A, Crottogini L, Gramegna M, Melegaro A, Merler S
Title	Potential short-term outcome of an uncontrolled COVID-19 epidemic in Lombardy, Italy, February to March 2020
Year	2020
Journal	Eurosurveillance
DOI	10.2807/1560-7917.ES.2020.25.12.2000293

– continued on next page –

Author	Hammoumi A, Qesmi R
Title	Impact assessment of containment measure against COVID-19 spread in Morocco
Year	2020
Journal	Chaos, Solitons and Fractals
DOI	10.1016/j.chaos.2020.110231
Author	Hogan AB, Jewell BL, Sherrard-Smith E, Vesga JF, Watson OJ, Whittaker C, Hamlet A, Smith JA, Winskill P, Verity R, Baguelin M, Lees JA, Whittles LK, Ainslie KEC, Bhatt S, Boonyasiri A, Brazeau NF, Cattarino L, Cooper LV, Coupland H, Cuomo-Dannenburg G, Dighe A, Djaafara BA, Donnelly CA, Eaton JW, van Elsland SL, FitzJohn RG, Fu H, Gaythorpe KAM, Green W, Haw DJ, Hayes S, Hinsley W, Imai N, Laydon DJ, Mangal TD, Mellan TA, Mishra S, Nedjati-Gilani G, Parag KV, Thompson HA, Unwin HJT, Vollmer MAC, Walters CE, Wang HW, Wang YR, Xi XY, Ferguson NM, Okell LC, Churcher TS, Arinaminpathy N, Ghani AC, Walker PGT, Hallett TB
Title	Potential impact of the COVID-19 pandemic on HIV, tuberculosis, and malaria in low-income and middle-income countries: a modelling study
Year	2020
Journal	Lancet Global Health
DOI	10.1016/S2214-109X(20)30288-6
Author	Hossain MP, Junus A, Zhu X, Jia P, Wen TH, Pfeiffer D, Yuan HY
Title	The effects of border control and quarantine measures on the spread of COVID-19
Year	2020
Journal	Epidemics
DOI	10.1016/j.epidem.2020.100397

– continued on next page –

Author	Hou C, Chen JX, Zhou YQ, Hua L, Yuan JX, He S, Guo Y, Zhang S, Jia QW, Zhao CH, Zhang J, Xu GX, Jia EZ
Title	The effectiveness of quarantine of Wuhan city against the Corona Virus Disease 2019 (COVID-19): A well-mixed SEIR model analysis
Year	2020
Journal	Journal of Medical Virology
DOI	10.1002/jmv.25827
Author	Hu L, Nie LF
Title	Dynamic modeling and analysis of COVID-19 in different transmission process and control strategies
Year	2020
Journal	Mathematical Methods in the Applied Sciences
DOI	10.1002/mma.6839
Author	Huang YB, Wu Y, Zhang WD
Title	Comprehensive identification and isolation policies have effectively suppressed the spread of COVID-19
Year	2020
Journal	Chaos, Solitons and Fractals
DOI	10.1016/j.chaos.2020.110041
Author	Iboi EA, Sharomi O, Ngonghala CN, Gumel AB
Title	Mathematical modeling and analysis of COVID-19 pandemic in Nigeria
Year	2020
Journal	Mathematical Biosciences and Engineering
DOI	10.3934/MBE.2020369
Author	Jackson ML

– continued on next page –

Title	Low-impact social distancing interventions to mitigate local epidemics of SARS-CoV-2
Year	2020
Journal	Microbes and Infection
DOI	10.1016/j.micinf.2020.09.006
Author	Jia JW, Ding J, Liu SY, Liao GD, Li JZ, Duan B, Wang GQ, Zhang R
Title	Modeling the control of Covid-19: Impact of policy interventions and meteorological factors
Year	2020
Journal	Electronic Journal Of Differential Equations
DOI	not available
Author	Jiang S, Li Q, Li C, Liu S, He X, Wang T, Li H, Corpe C, Zhang X, Xu J, Wang J
Title	Mathematical models for devising the optimal SARS-CoV-2 strategy for eradication in China, South Korea, and Italy
Year	2020
Journal	Journal of Translational Medicine
DOI	10.1186/s12967-020-02513-7
Author	Kane GQ, Amba MCO, Mbratana T
Title	Early view of the potential impact of government policy responses on reproductive number of COVID-19 in African countries: Evidence from Cameroon
Year	2020
Journal	Economics Bulletin
DOI	not available
Author	Kassa SM, Njagarah JBH, Terefe YA

– continued on next page –

Title	Analysis of the mitigation strategies for COVID-19: From mathematical modelling perspective
Year	2020
Journal	Chaos, Solitons and Fractals
DOI	10.1016/j.chaos.2020.109968
Author	Khajji B, Kada D, Balatif O, Rachik M
Title	A multi-region discrete time mathematical modeling of the dynamics of Covid-19 virus propagation using optimal control
Year	2020
Journal	Journal of Applied Mathematics and Computing
DOI	10.1007/s12190-020-01354-3
Author	Kheirallah KA, Alsinglawi B, Alzoubi A, Saidan MN, Mubin O, Alorjani MS, Mzayek F
Title	The effect of strict state measures on the epidemiologic curve of covid-19 infection in the context of a developing country: A simulation from Jordan
Year	2020
Journal	International Journal of Environmental Research and Public Health
DOI	10.3390/ijerph17186530
Author	Kim BN, Kim E, Lee S, Oh C
Title	Mathematical model of covid-19 transmission dynamics in South Korea: The impacts of travel restrictions, social distancing, and early detection
Year	2020
Journal	Processes
DOI	10.3390/pr8101304
Author	Kim S, Kim YJ, Peck KR, Jung E

– continued on next page –

Title	School Opening Delay Effect on Transmission Dynamics of Coronavirus Disease 2019 in Korea: Based on Mathematical Modeling and Simulation Study
Year	2020
Journal	Journal of Korean medical science
DOI	10.3346/jkms.2020.35.e143
Author	Kimathi M, Mwalili S, Ojiambo V, Gathungu DK
Title	Age-structured model for COVID-19: Effectiveness of social distancing and contact reduction in Kenya
Year	2020
Journal	Infectious Disease Modelling
DOI	10.1016/j.idm.2020.10.012
Author	Kucharski AJ, Klepac P, Conlan AJK, Kissler SM, Tang ML, Fry H, Gog JR, Edmunds WJ, Emery JC, Medley G, Munday JD, Russell TW, Leclerc QJ, Diamond C, Procter SR, Gimma A, Sun FY, Gibbs HP, Rosello A, van Zandvoort K, Hué S, Meakin SR, Deol AK, Knight G, Jombart T, Foss AM, Bosse NI, Atkins KE, Quilty BJ, Lowe R, Prem K, Flasche S, Pearson CAB, Houben RMGJ, Nightingale ES, Endo A, Tully DC, Liu Y, Villabona-Arenas J, O'Reilly K, Funk S, Eggo RM, Jit M, Rees EM, Hellewell J, Clifford S, Jarvis CI, Abbott S, Auzenbergs M, Davies NG, Simons D
Title	Effectiveness of isolation, testing, contact tracing, and physical distancing on reducing transmission of SARS-CoV-2 in different settings: a mathematical modelling study
Year	2020
Journal	The Lancet Infectious Diseases
DOI	10.1016/S1473-3099(20)30457-6
Author	Kuniya T, Inaba H

– continued on next page –

Title	Possible effects of mixed prevention strategy for COVID-19 epidemic: massive testing, quarantine and social distancing
Year	2020
Journal	AIMS Public Health
DOI	10.3934/publichealth.2020040
Author	Leung K, Wu JT, Liu D, Leung GM
Title	First-wave COVID-19 transmissibility and severity in China outside Hubei after control measures, and second-wave scenario planning: a modelling impact assessment
Year	2020
Journal	The Lancet
DOI	10.1016/S0140-6736(20)30746-7
Author	Lhous M, Zakary O, Rachik M, Magri EM, Tridane A
Title	Optimal containment control strategy of the second phase of the covid-19 lockdown in morocco
Year	2020
Journal	Applied Sciences (Switzerland)
DOI	10.3390/app10217559
Author	Linka K, Rahman P, Goriely A, Kuhl E
Title	Is it safe to lift COVID-19 travel bans? The Newfoundland story
Year	2020
Journal	Computational Mechanics
DOI	10.1007/s00466-020-01899-x
Author	Loewenthal G, Abadi S, Avram O, Halabi K, Ecker N, Nagar N, Mayrose I, Pupko T
Title	An evaluation of COVID-19 in Italy: A data-driven modeling analysis

– continued on next page –

Year	2020
Journal	Infectious Disease Modelling
DOI	10.1016/j.idm.2020.06.007
Author	Mahdizadeh Gharakhanlou N, Hooshangi N
Title	Spatio-temporal simulation of the novel coronavirus (COVID-19) outbreak using the agent-based modeling approach (case study: Urmia, Iran)
Year	2020
Journal	Informatics in Medicine Unlocked
DOI	10.1016/j.imu.2020.100403
Author	Marimuthu Y, Nagappa B, Sharma N, Basu S, Chopra KK
Title	COVID-19 and tuberculosis: A mathematical model based forecasting in Delhi, India
Year	2020
Journal	Indian Journal of Tuberculosis
DOI	10.1016/j.ijtb.2020.05.006
Author	Matrajt L, Leung T
Title	Evaluating the effectiveness of social distancing interventions to delay or flatten the epidemic curve of Coronavirus disease
Year	2020
Journal	Emerging Infectious Diseases
DOI	10.3201/eid2608.201093
Author	McCombs A, Kadelka C
Title	A model-based evaluation of the efficacy of COVID-19 social distancing, testing and hospital triage policies
Year	2020
Journal	PLoS Computational Biology

– continued on next page –

DOI	10.1371/journal.pcbi.1008388
Author	Milhinhos A, Costa PM
Title	On the Progression of COVID-19 in Portugal: A Comparative Analysis of Active Cases Using Non-linear Regression
Year	2020
Journal	Frontiers in Public Health
DOI	10.3389/fpubh.2020.00495
Author	Min KD, Kang H, Lee JY, Jeon S, Cho SI
Title	Estimating the effectiveness of non-pharmaceutical interventions on COVID-19 control in Korea
Year	2020
Journal	Journal of Korean Medical Science
DOI	10.3346/JKMS.2020.35.E321
Author	Murillo MFM, Murillo WAM, Pandey D
Title	Efficient Prediction and Analysis of the possible evolution of the SARS-CoV-2 in COVID-19 applying the SEIR model in Santo Domingo de los Tsachilas, Ecuador
Year	2020
Journal	Enfoque UTE
DOI	10.29019/enfoqueute.v11n4.678
Author	Nawaz A, Su X, Iqbal S, Zahoor H, Asad A, Asghar S, Basit F, Barkat MQ, Souhail A, Raheel Shah SA
Title	Validating a Phenomenological Mathematical Model for Public Health and Safety Interventions Influencing the Evolutionary Stages of Recent Outbreak for Long-Term and Short-Term Domains in Pakistan
Year	2020
Journal	Complexity

– continued on next page –

DOI	10.1155/2020/8866071
Author	Ng WL
Title	To lockdown? When to peak? Will there be an end? A macroeconomic analysis on COVID-19 epidemic in the United States
Year	2020
Journal	Journal of Macroeconomics
DOI	10.1016/j.jmacro.2020.103230
Author	Ngonghala CN, Iboi E, Eikenberry S, Scotch M, MacIntyre CR, Bonds MH, Gumel AB
Title	Mathematical assessment of the impact of non-pharmaceutical interventions on curtailing the 2019 novel Coronavirus
Year	2020
Journal	Mathematical Biosciences
DOI	10.1016/j.mbs.2020.108364
Author	Nkwayep CH, Bowong S, Tewa JJ, Kurths J
Title	Short-term forecasts of the COVID-19 pandemic: a study case of Cameroon
Year	2020
Journal	Chaos, Solitons and Fractals
DOI	10.1016/j.chaos.2020.110106
Author	Nuzzo A, Tan CO, Raskar R, DeSimone DC, Kapa S, Gupta R
Title	Universal Shelter-in-Place Versus Advanced Automated Contact Tracing and Targeted Isolation: A Case for 21st-Century Technologies for SARS-CoV-2 and Future Pandemics
Year	2020
Journal	Mayo Clinic Proceedings
DOI	10.1016/j.mayocp.2020.06.027

– continued on next page –

Author	Olaniyi S, Obabiyi OS, Okosun KO, Oladipo AT, Adewale SO
Title	Mathematical modelling and optimal cost-effective control of COVID-19 transmission dynamics
Year	2020
Journal	European Physical Journal Plus
DOI	10.1140/epjp/s13360-020-00954-z
Author	Olfatifar M, Houri H, Shojaee S, Pourhoseingholi MA, Alali WQ, Busani L, Ashtari S, Shahrokh S, Vahedian A, Aghdaei HA
Title	The Required Confronting Approaches Efficacy and Time to Control COVID-19 Outbreak in Iran
Year	2020
Journal	Archives ff Clinical Infectious Diseases
DOI	10.5812/archcid.102633
Author	Overton CE, Stage HB, Ahmad S, Curran-Sebastian J, Dark P, Das R, Fearon E, Felton T, Fyles M, Gent N, Hall I, House T, Lewkowicz H, Pang X, Pellis L, Sawko R, Ustianowski A, Vekaria B, Webb L
Title	Using statistics and mathematical modelling to understand infectious disease outbreaks: COVID-19 as an example
Year	2020
Journal	Infectious Disease Modelling
DOI	10.1016/j.idm.2020.06.008
Author	Ozair M, Hussain T, Hussain M, Awan AU, Baleanu D, Abro KA
Title	A Mathematical and Statistical Estimation of Potential Transmission and Severity of COVID-19: A Combined Study of Romania and Pakistan
Year	2020
Journal	BioMed Research International

– continued on next page –

DOI	10.1155/2020/5607236
Author	Pais RJ, Taveira N
Title	Predicting the evolution and control of the COVID-19 pandemic in Portugal
Year	2020
Journal	F1000Research
DOI	10.12688/f1000research.23401.2
Author	Pan J, Tian J, Xiong H, Liu Z, Yao Y, Wang Y, Zhu W, Wang Y, Wang W
Title	Risk assessment and evaluation of China's policy to prevent COVID-19 cases imported by plane
Year	2020
Journal	PLoS neglected tropical diseases
DOI	10.1371/journal.pntd.0008908
Author	Peirlinck M, Linka K, Sahli Costabal F, Kuhl E
Title	Outbreak dynamics of COVID-19 in China and the United States
Year	2020
Journal	Biomechanics and Modeling in Mechanobiology
DOI	10.1007/s10237-020-01332-5
Author	Pernice S, Castagno P, Marcotulli L, Maule MM, Richiardi L, Moirano G, Sereno M, Cordero F, Beccuti M
Title	Impacts of reopening strategies for COVID-19 epidemic: a modeling study in Piedmont region
Year	2020
Journal	BMC Infectious Diseases
DOI	10.1186/s12879-020-05490-w
Author	Rădulescu A, Williams C, Cavanagh K

– continued on next page –

Title	Management strategies in a SEIR-type model of COVID 19 community spread
Year	2020
Journal	Scientific Reports
DOI	10.1038/s41598-020-77628-4
Author	Rainisch G, Undurraga EA, Chowell G
Title	A dynamic modeling tool for estimating healthcare demand from the COVID19 epidemic and evaluating population-wide interventions
Year	2020
Journal	International Journal of Infectious Diseases
DOI	10.1016/j.ijid.2020.05.043
Author	Razzaq OA, Rehman DU, Khan NA, Ahmadian A, Ferrara M
Title	Optimal surveillance mitigation of COVID'19 disease outbreak: Fractional order optimal control of compartment model
Year	2020
Journal	Results in Physics
DOI	10.1016/j.rinp.2020.103715
Author	Rivera-Rodriguez C, Urdinola BP
Title	Predicting Hospital Demand During the COVID-19 Outbreak in Bogota, Colombia
Year	2020
Journal	Frontiers in Public Health
DOI	10.3389/fpubh.2020.582706
Author	Ruktanonchai NW, Floyd JR, Lai S, Ruktanonchai CW, Sadilek A, Rente-Lourenco P, Ben X, Carioli A, Gwinn J, Steele JE, Prosper O, Schneider A, Oplinger A, Eastham P, Tatem AJ

– continued on next page –

Title	Assessing the impact of coordinated COVID-19 exit strategies across Europe
Year	2020
Journal	Science
DOI	10.1126/science.abc5096
Author	Saldaña F, Flores-Arguedas H, Camacho-Gutiérrez JA, Barradas I
Title	Modeling the transmission dynamics and the impact of the control interventions for the COVID-19 epidemic outbreak
Year	2020
Journal	Mathematical biosciences and engineering
DOI	10.3934/mbe.2020231
Author	Schneider KA, Ngwa GA, Schwehm M, Eichner L, Eichner M
Title	The COVID-19 pandemic preparedness simulation tool: CovidSIM
Year	2020
Journal	BMC Infectious Diseases
DOI	10.1186/s12879-020-05566-7
Author	Singh A, Chandra SK, Bajpai MK
Title	Study of non-pharmacological interventions on covid-19 spread
Year	2020
Journal	CMES - Computer Modeling in Engineering and Sciences
DOI	10.32604/cmes.2020.011601
Author	Sjödin H, Johansson AF, Brännström Å, Farooq Z, Kriit HK, Wilder-Smith A, Åström C, Thunberg J, Söderquist M, Rocklöv J
Title	COVID-19 healthcare demand and mortality in Sweden in response to non-pharmaceutical mitigation and suppression scenarios
Year	2020
Journal	International Journal of Epidemiology

– continued on next page –

DOI	10.1093/ije/dyaa121
Author	Su L, Hong N, Zhou X, He J, Ma Y, Jiang H, Han L, Chang F, Shan G, Zhu W, Long Y
Title	Evaluation of the Secondary Transmission Pattern and Epidemic Prediction of COVID-19 in the Four Metropolitan Areas of China
Year	2020
Journal	Frontiers in Medicine
DOI	10.3389/fmed.2020.00171
Author	Sy C, Bernardo E, Miguel A, San Juan JL, Mayol AP, Ching PM, Culaba A, Ubando A, Mutuc JE
Title	Policy Development for Pandemic Response Using System Dynamics: a Case Study on COVID-19
Year	2020
Journal	Process Integration and Optimization for Sustainability
DOI	10.1007/s41660-020-00130-x
Author	Taboe HB, Salako KV, Tison JM, Ngonghala CN, Glèlè Kakaï R
Title	Predicting COVID-19 spread in the face of control measures in West Africa
Year	2020
Journal	Mathematical Biosciences
DOI	10.1016/j.mbs.2020.108431
Author	Tang B, Scarabel F, Bragazzi NL, McCarthy Z, Glazer M, Xiao Y, Heffernan JM, Asgary A, Ogden NH, Wu J
Title	De-escalation by reversing the escalation with a stronger synergistic package of contact tracing, quarantine, isolation and personal protection: Feasibility of preventing a covid-19 rebound in Ontario, Canada, as a case study
Year	2020
Journal	Biology

– continued on next page –

DOI	10.3390/biology9050100
Author	Tantrakarnapa K, Bhopdhornangkul B
Title	Challenging the spread of COVID-19 in Thailand
Year	2020
Journal	One Health
DOI	10.1016/j.onehlt.2020.100173
Author	Tatapudi H, Das R, Das TK
Title	Impact assessment of full and partial stay-at-home orders, face mask usage, and contact tracing: An agent-based simulation study of COVID-19 for an urban region
Year	2020
Journal	Global Epidemiology
DOI	10.1016/j.gloepi.2020.100036
Author	Tocto-Erazo MR, Espíndola-Zepeda JA, Montoya-Laos JA, Acuña-Zegarra MA, Olmos-Liceaga D, Reyes-Castro PA, FigueroaPreciado G
Title	Lockdown, relaxation, and acme period in COVID-19: A study of disease dynamics in Hermosillo, Sonora, Mexico
Year	2020
Journal	PLOS ONE
DOI	10.1371/journal.pone.0242957
Author	Traini MC, Caponi C, Ferrari R, De Socio GV
Title	A study of SARS-CoV-2 epidemiology in Italy: from early days to secondary effects after social distancing
Year	2020
Journal	Infectious Diseases
DOI	10.1080/23744235.2020.1797157

– continued on next page –

Author	Tsay C, Lejarza F, Stadtherr MA, Baldea M
Title	Modeling, state estimation, and optimal control for the US COVID-19 outbreak
Year	2020
Journal	Scientific reports
DOI	10.1038/s41598-020-67459-8
Author	Valenti VE, de Lemos Menezes P, de Abreu ACG, Vieira GNA, Garner DM
Title	Social distancing measures could have reduced estimated deaths related to COVID-19 in Brazil
Year	2020
Journal	Journal of Human Growth and Development
DOI	10.7322/JHGD.V30.10360
Author	Varotsos CA, Krapivin VF
Title	A new model for the spread of COVID-19 and the improvement of safety
Year	2020
Journal	Safety Science
DOI	10.1016/j.ssci.2020.104962
Author	Volpert V, Banerjee M, Petrovskii S
Title	On a quarantine model of coronavirus infection and data analysis
Year	2020
Journal	Mathematical Modelling of Natural Phenomena
DOI	10.1051/mmnp/2020006
Author	Wang K, Lu ZZ, Wang XM, Li H, Li HL, Lin DD, Cai YL, Feng X, Song YT, Feng ZW, Ji WD, Wang XY, Yin Y, Wang L, Peng ZH
Title	Current trends and future prediction of novel coronavirus disease (COVID-19) epidemic in China: a dynamical modeling analysis

– continued on next page –

Year	2020
Journal	Mathematical biosciences and engineering
DOI	10.3934/mbe.2020173
Author	Wang X, Du Z, Huang G, Pasco RF, Fox SJ, Galvani AP, Pignone M, Claiborne Johnston S, Meyers LA
Title	Effects of cocooning on coronavirus disease rates after relaxing social distancing
Year	2020
Journal	Emerging Infectious Diseases
DOI	10.3201/EID2612.201930
Author	Wang X, Pasco RF, Du Z, Petty M, Fox SJ, Galvani AP, Pignone M, Claiborne Johnston S, Meyers LA
Title	Impact of social distancing measures on coronavirus disease healthcare demand, central Texas, USA
Year	2020
Journal	Emerging Infectious Diseases
DOI	10.3201/eid2610.201702
Author	Wei Y, Wang J, Song W, Xiu C, Ma L, Pei T
Title	Spread of COVID-19 in China: analysis from a city-based epidemic and mobility model
Year	2020
Journal	Cities
DOI	10.1016/j.cities.2020.103010
Author	Yang JY, Wang GQ, Zhang S
Title	Impact of household quarantine on SARS-Cov-2 infection in mainland China: A mean-field modelling approach

– continued on next page –

Year	2020
Journal	Mathematical biosciences and engineering
DOI	10.3934/mbe.2020248
Author	Yoo S, Managi S
Title	Global mortality benefits of COVID-19 action
Year	2020
Journal	Technological Forecasting And Social Change
DOI	10.1016/j.techfore.2020.120231
Author	Zhang K, Vilches TN, Tariq M, Galvani AP, Moghadas SM
Title	The impact of mask-wearing and shelter-in-place on COVID-19 outbreaks in the United States
Year	2020
Journal	International Journal of Infectious Diseases
DOI	10.1016/j.ijid.2020.10.002
Author	Zhang Y, Yu X, Sun H, Tick GR, Wei W, Jin B
Title	Applicability of time fractional derivative models for simulating the dynamics and mitigation scenarios of COVID-19
Year	2020
Journal	Chaos, Solitons and Fractals
DOI	10.1016/j.chaos.2020.109959
Author	Zhou Y, Xu RZ, Hu DS, Yue Y, Li QQ, Xia JZ
Title	Effects of human mobility restrictions on the spread of COVID-19 in Shenzhen, China: a modelling study using mobile phone data
Year	2020
Journal	Lancet Digital Health

– continued on next page –

Only simulation of NPI effects in hypothetical scenarios – continued from previous page

DOI	10.1016/S2589-7500(20)30165-5
Author	Zhu HJ, Li Y, Jin XL, Huang JP, Liu X, Qian Y, Tan JD
Title	Transmission dynamics and control methodology of COVID-19: A modeling study
Year	2020
Journal	Applied Mathematical Modelling
DOI	10.1016/j.apm.2020.08.056
Author	Zongo P, Zorom M, Mophou G, Dorville R, Beaumont C
Title	A model of covid-19 transmission to understand the effectiveness of the containment measures: Application to data from France
Year	2020
Journal	Epidemiology and Infection
DOI	10.1017/S0950268820002162
Author	Zu J, Li ML, Li ZF, Shen MW, Xiao YN, Ji FP
Title	Transmission patterns of COVID-19 in the mainland of China and the efficacy of different control strategies: a data- And model-driven study
Year	2020
Journal	Infectious Diseases of Poverty
DOI	10.1186/s40249-020-00709-z

Different research objective than assessment of NPI effects

Different research objective than assessment of NPI effects

Author	Bajiya VP, Bugalia S, Tripathi JP
Title	Mathematical modeling of COVID-19: Impact of non-pharmaceutical interventions in India
Year	2020

– continued on next page –

Journal	Chaos
DOI	10.1063/5.0021353
Author	Bianconi A, Marcelli A, Campi G, Perali A
Title	Ostwald Growth Rate in Controlled Covid-19 Epidemic Spreading as in Arrested Growth in Quantum Complex Matter
Year	2020
Journal	Condensed Matter
DOI	10.3390/condmat5020023
Author	Biswas SK, Ghosh JK, Sarkar S, Ghosh U
Title	COVID-19 pandemic in India: a mathematical model study
Year	2020
Journal	Nonlinear Dynamics
DOI	10.1007/s11071-020-05958-z
Author	Bright A, Glynn-Robinson AJ, Kane S, Wright R, Saul N
Title	The effect of COVID-19 public health measures on nationally notifiable diseases in Australia: preliminary analysis
Year	2020
Journal	Communicable diseases intelligence
DOI	10.33321/cdi.2020.44.85
Author	De Visscher A
Title	The COVID-19 pandemic: model-based evaluation of non-pharmaceutical interventions and prognoses
Year	2020
Journal	Nonlinear Dynamics
DOI	10.1007/s11071-020-05861-7

– continued on next page –

Author	Debecker A, Modis T
Title	Poorly known aspects of flattening the curve of COVID-19
Year	2020
Journal	Technological Forecasting and Social Change
DOI	10.1016/j.techfore.2020.120432
Author	Grossman G., Kim S., Rexer J.M., Thirumurthy H.
Title	Political partisanship influences behavioral responses to governors' recommendations for COVID-19 prevention in the United States
Year	2020
Journal	Proceedings of the National Academy of Sciences of the United States of America
DOI	10.1073/pnas.2007835117
Author	Hong HG, Li Y
Title	Estimation of time-varying reproduction numbers underlying epidemiological processes: A new statistical tool for the COVID-19 pandemic
Year	2020
Journal	PLOS ONE
DOI	10.1371/journal.pone.0236464
Author	Kaxiras E, Neofotistos G
Title	Multiple epidemic wave model of the COVID-19 pandemic: Modeling study
Year	2020
Journal	Journal of Medical Internet Research
DOI	10.2196/20912
Author	Kosmidis K, Macheras P
Title	A fractal kinetics SI model can explain the dynamics of COVID-19 epidemics
Year	2020

– continued on next page –

Journal	PLOS ONE
DOI	10.1371/journal.pone.0237304
Author	Lee W, Hwang SS, Song I, Park C, Kim H, Song IK, Choi HM, Prifti K, Kwon Y, Kim J, Oh S, Yang J, Cha M, Kim Y, Bell ML, Kim H
Title	COVID-19 in South Korea: Epidemiological and spatiotemporal patterns of the spread and the role of aggressive diagnostic tests in the early phase
Year	2020
Journal	International Journal of Epidemiology
DOI	10.1093/ije/dyaa119
Author	Lupu D, Maha LG, Viorică ED
Title	Covid-19 incidence in europe: Drivers and government interventions
Year	2020
Journal	Transylvanian Review of Administrative Sciences
DOI	10.24193/tras.SI2020.5
Author	Mahajan P, Kaushal J
Title	Epidemic Trend of COVID-19 Transmission in India During Lockdown-1 Phase
Year	2020
Journal	Journal of community health
DOI	10.1007/s10900-020-00863-3
Author	Maziarz M, Zach M
Title	Agent-based modelling for SARS-CoV-2 epidemic prediction and intervention assessment: A methodological appraisal
Year	2020
Journal	Journal of Evaluation in Clinical Practice
DOI	10.1111/jep.13459

– continued on next page –

Author	Monllor P, Su Z, Gabrielli L, Taltavull de La Paz P
Title	COVID-19 Infection Process in Italy and Spain: Are Data Talking? Evidence From ARMA and Vector Autoregression Models
Year	2020
Journal	Frontiers in Public Health
DOI	10.3389/fpubh.2020.550602
Author	Ponjavić M, Karabegović A, Ferhatbegović E, Tahirović E, Uzunović S, Travar M, Pilav A, Mulić M, Karakaš S, Avdić N, Mulabdić Z, Pavić G, Bičo M, Vasilj I, Mamić D, Hukić M
Title	Spatio-temporal data visualization for monitoring of control measures in the prevention of the spread of COVID-19 in Bosnia and Herzegovina
Year	2020
Journal	Medicinski Glasnik
DOI	10.17392/1215-20
Author	Pratomo H
Title	From Social Distancing to Physical Distancing: A Challenge for Evaluating Public Health Intervention against COVID-19
Year	2020
Journal	Kesmas: National Public Health Journal
DOI	10.21109/kesmas.v15i2.4010
Author	Rajendrakumar AL, Nair ATN, Nangia C, Chourasia PK, Chourasia MK, Syed MG, Nair AS, Nair AB, Koya MSF
Title	Epidemic Landscape and Forecasting of SARS-CoV-2 in India
Year	2020
Journal	Journal of Epidemiology and Global Health
DOI	10.2991/jegh.k.200823.001

– continued on next page –

Author	Ramchandani A, Fan C, Mostafavi A
Title	DeepCOVIDNet: An Interpretable Deep Learning Model for Predictive Surveillance of COVID-19 Using Heterogeneous Features and Their Interactions
Year	2020
Journal	IEEE Access
DOI	10.1109/ACCESS.2020.3019989
Author	Rotejanaprasert C, Lawpoolsri S, Pan-Ngum W, Maude RJ
Title	Preliminary estimation of temporal and spatiotemporal dynamic measures of COVID- 19 transmission in Thailand
Year	2020
Journal	PLOS ONE
DOI	10.1371/journal.pone.0239645
Author	Sun J, Chen X, Zhang Z, Lai S, Zhao B, Liu H, Wang S, Huan W, Zhao R, Ng MTA, Zheng Y
Title	Forecasting the long-term trend of COVID-19 epidemic using a dynamic model
Year	2020
Journal	Scientific Reports
DOI	10.1038/s41598-020-78084-w
Author	Sun ZB, Zhang H, Yang YF, Wan H, Wang YX
Title	Impacts of geographic factors and population density on the COVID-19 spreading under the lockdown policies of China
Year	2020
Journal	Science ff The Total Environment
DOI	10.1016/j.scitotenv.2020.141347

– continued on next page –

Author	Tariq A, Lee Y, Roosa K, Blumberg S, Yan P, Ma S, Chowell G
Title	Real-time monitoring the transmission potential of COVID-19 in Singapore, March 2020
Year	2020
Journal	BMC Medicine
DOI	10.1186/s12916-020-01615-9
Author	Wang KW, Gao J, Wang H, Wu XL, Yuan QF, Guo FY, Zhang ZJ, Cheng Y
Title	Epidemiology of 2019 novel coronavirus in Jiangsu Province, China after wartime control measures: A population-level retrospective study
Year	2020
Journal	Travel Medicine and Infectious Disease
DOI	10.1016/j.tmaid.2020.101654
Author	Wang PP, Zheng XQ, Ai G, Liu DY, Zhu BR
Title	Time series prediction for the epidemic trends of COVID-19 using the improved LSTM deep learning method: Case studies in Russia, Peru and Iran
Year	2020
Journal	Chaos Solitons & Fractals
DOI	10.1016/j.chaos.2020.110214
Author	Ylli A, Wu YY, Burazeri G, Pirkle C, Sentell T
Title	The lower COVID-19 related mortality and incidence rates in Eastern European countries are associated with delayed start of community circulation
Year	2020
Journal	PLOS ONE
DOI	10.1371/journal.pone.0243411

– continued on next page –

Author	Zhang N, Shi T, Zhong H, Guo Y
Title	COVID-19 Prevention and Control Public Health Strategies in Shanghai, China
Year	2020
Journal	Journal of public health management and practice : JPHMP
DOI	10.1097/PHH.0000000000001202
Author	Zhou L, Liu JM, Dong XP, McGoogan JM, Wu ZY
Title	COVID-19 seeding time and doubling time model: An early epidemic risk assessment tool
Year	2020
Journal	Infectious Diseases of Poverty
DOI	10.1186/s40249-020-00685-4
Author	Zhou WK, Wang AL, Xia F, Xiao YN, Tang SY
Title	Effects of media reporting on mitigating spread of COVID-19 in the early phase of the outbreak
Year	2020
Journal	Mathematical biosciences and engineering
DOI	10.3934/mbe.2020147
Author	Zhou YM, Li J, Ye LJ, Chen ZG, Luo QS, Wu XD, Ni HY
Title	Random Network Transmission and Countermeasures in Containing Global Spread of COVID-19-Alike Pandemic: A Hybrid Modelling Approach
Year	2020
Journal	Complexity
DOI	10.1155/2020/6703703

Only assessment of effects of mobility or behavior, not of NPIs

Only assessment of effects of mobility or behavior, not of NPIs	
Author	Borjas GJ
Title	Business closures, stay-at-home restrictions, and COVID-19 testing outcomes in New York City
Year	2020
Journal	Preventing Chronic Disease
DOI	10.5888/PCD17.200264
Author	Bryant P, Elofsson A
Title	Estimating the impact of mobility patterns on COVID-19 infection rates in 11 European countries
Year	2020
Journal	PeerJ
DOI	10.7717/peerj.9879
Author	Delen D, Eryarsoy E, Davazdahemami B
Title	No place like home: Cross-national data analysis of the efficacy of social distancing during the COVID-19 pandemic
Year	2020
Journal	JMIR Public Health and Surveillance
DOI	10.2196/19862
Author	Durmus H, Gökler ME, Metintas S
Title	The effectiveness of community-based social distancing for mitigating the spread of the COVID-19 pandemic in Turkey
Year	2020
Journal	Journal of Preventive Medicine and Public Health
DOI	10.3961/JPMPH.20.381

– continued on next page –

Author	Ferguson NM, Ainslie KEC, Walters CE, Fu H, Bhatia S, Wang H, Xi X, Baguelin M, Bhatt S, Boonyasiri A, Boyd O, Cattarino L, Ciavarella C, Cucunuba Z, Cuomo-Dannenburg G, Dighe A, Dorigatti I, van Elsland SL, FitzJohn R, Gaythorpe K, Ghani AC, Green W, Hamlet A, Hinsley W, Imai N, Jorgensen D, Knock E, Laydon D, Nedjati-Gilani G, Okell LC, Siveroni I, Thompson HA, Unwin HJT, Verity R, Vollmer M, Walker PGT, Wang Y, Watson OJ, Whittaker C, Winskill P, Donnelly CA, Riley S
Title	Evidence of initial success for China exiting COVID-19 social distancing policy after achieving containment
Year	2020
Journal	Wellcome Open Research
DOI	10.12688/wellcomeopenres.15843.1
Author	Iacus SM, Santamaria C, Sermi F, Spyratos S, Tarchi D, Vespe M
Title	Human mobility and COVID-19 initial dynamics
Year	2020
Journal	Nonlinear Dynamics
DOI	10.1007/s11071-020-05854-6
Author	Jang WM, Jang DH, Lee JY
Title	Social Distancing and Transmission-reducing Practices during the 2019 Coronavirus Disease and 2015 Middle East Respiratory Syndrome Coronavirus Outbreaks in Korea
Year	2020
Journal	Journal of Korean Medical Science
DOI	10.3346/jkms.2020.35.e220
Author	Kain MP, Childs ML, Becker AD, Mordecai EA
Title	Chopping the tail: How preventing superspreading can help to maintain COVID-19 control

– continued on next page –

Year	2020
Journal	Epidemics
DOI	10.1016/j.epidem.2020.100430
Author	Lai S, Ruktanonchai NW, Zhou L, Prosper O, Luo W, Floyd JR, Wesolowski A, Santillana M, Zhang C, Du X, Yu H, Tatem AJ
Title	Effect of non-pharmaceutical interventions to contain COVID-19 in China
Year	2020
Journal	Nature
DOI	10.1038/s41586-020-2293-x
Author	Loewenthal G, Abadi S, Avram O, Halabi K, Ecker N, Nagar N, Mayrose I, Pupko T
Title	COVID-19 pandemic-related lockdown: response time is more important than its strictness
Year	2020
Journal	Embo Molecular Medicine
DOI	10.15252/emmm.202013171
Author	Morley CP, Anderson KB, Shaw J, Stewart T, Thomas SJ, Wang D
Title	Social Distancing Metrics and Estimates of SARS-CoV-2 Transmission Rates: Associations Between Mobile Telephone Data Tracking and R
Year	2020
Journal	Journal of Public Health Management and Practice
DOI	10.1097/PHH.0000000000001240
Author	Ogundokun RO, Lukman AF, Kibria GBM, Awotunde JB, Aladeitan BB
Title	Predictive modelling of COVID-19 confirmed cases in Nigeria
Year	2020
Journal	Infectious Disease Modelling

– continued on next page –

DOI	10.1016/j.idm.2020.08.003
Author	Rubin D, Huang J, Fisher BT, Gasparrini A, Tam V, Song L, Wang X, Kaufman J, Fitzpatrick K, Jain A, Griffis H, Crammer K, Morris J, Tasian G
Title	Association of Social Distancing, Population Density, and Temperature with the Instantaneous Reproduction Number of SARS-CoV-2 in Counties across the United States
Year	2020
Journal	JAMA Network Open
DOI	10.1001/jamanetworkopen.2020.16099
Author	Unwin HJT, Mishra S, Bradley VC, Gandy A, Mellan TA, Coupland H, Ish-Horowicz J, Vollmer MAC, Whittaker C, Filippi SL, Xi X, Monod M, Ratmann O, Hutchinson M, Valka F, Zhu H, Hawryluk I, Milton P, Ainslie KEC, Baguelin M, Boonyasiri A, Brazeau NF, Cattarino L, Cucunuba Z, Cuomo-Dannenburg G, Dorigatti I, Eales OD, Eaton JW, van Elsland SL, FitzJohn RG, Gaythorpe KAM, Green W, Hinsley W, Jeffrey B, Knock E, Laydon DJ, Lees J, Nedjati-Gilani G, Nouvellet P, Okell L, Parag KV, Siveroni I, Thompson HA, Walker P, Walters CE, Watson OJ, Whittles LK, Ghani AC, Ferguson NM, Riley S, Donnelly CA, Bhatt S, Flaxman S
Title	State-level tracking of COVID-19 in the United States
Year	2020
Journal	Nature Communications
DOI	10.1038/s41467-020-19652-6
Author	Vokó Z, Pitter JG
Title	The effect of social distance measures on COVID-19 epidemics in Europe: an interrupted time series analysis
Year	2020
Journal	GeroScience

– continued on next page –

DOI	10.1007/s11357-020-00205-0
Author	Yuan X, Hu K, Xu J, Zhang X, Bao W, Lynch CF, Zhang L
Title	State heterogeneity in the associations of human mobility with COVID-19 epidemics in the European Union
Year	2020
Journal	American Journal of Translational Research
DOI	not available
Author	Zhao Y, Wang R, Li J, Zhang Y, Yang H
Title	Analysis of the Transmissibility Change of 2019-Novel Coronavirus Pneumonia and Its Potential Factors in China from 2019 to 2020
Year	2020
Journal	BioMed Research International
DOI	10.1155/2020/3842470

Use of results from other study

**Use of results from other study**	
Author	Dighe A, Cattarino L, Cuomo-Dannenburg G, Skarp J, Imai N, Bhatia S, Gaythorpe KAM, Ainslie KEC, Baguelin M, Bhatt S, Boonyasiri A, Brazeau NF, Cooper LV, Coupland H, Cucunuba Z, Dorigatti I, Eales OD, Van Elsland SL, Fitzjohn RG, Green WD, Haw DJ, Hinsley W, Knock E, Laydon DJ, Mellan T, Mishra S, Nedjati-Gilani G, Nouvellet P, Pons-Salort M, Thompson HA, Unwin HJT, Verity R, Vollmer MAC, Walters CE, Watson OJ, Whittaker C, Whittles LK, Ghani AC, Donnelly CA, Ferguson NM, Riley S
Title	Response to COVID-19 in South Korea and implications for lifting stringent interventions
Year	2020

– continued on next page –

Journal	BMC Medicine
DOI	10.1186/s12916-020-01791-8
Author	Galbán-García E, Más-Bermejo P
Title	COVID-19 in Cuba: Assessing the national response
Year	2020
Journal	MEDICC Review
DOI	10.37757/MR2020.V22.N4.5
Author	Giachino M, Valera CBG, Velásquez SR, Dohrendorf-Wyss MA, Rozanova L, Flahault A
Title	Understanding the dynamics of the covid-19 pandemic: A real-time analysis of switzerland's first wave
Year	2020
Journal	International Journal of Environmental Research and Public Health
DOI	10.3390/ijerph17238825
Author	Hassan R, Dosar AS, Mondol JK, Khan TH, Noman AA, Sayem MS, Hasan M, Juyena NS
Title	Prediction of Epidemics Trend of COVID-19 in Bangladesh
Year	2020
Journal	Frontiers in Public Health
DOI	10.3389/fpubh.2020.559437
Author	Imai N, Gaythorpe KAM, Abbott S, Bhatia S, van Elsland S, Prem K, Liu Y, Ferguson NM
Title	Adoption and impact of non-pharmaceutical interventions for COVID-19
Year	2020
Journal	Wellcome Open Research
DOI	10.12688/wellcomeopenres.15808.1

– continued on next page –

Use of results from other study – continued from previous page

Author	Medina-Ortiz D, Contreras S, Barrera-Saavedra Y, Cabas-Mora G, Olivera-Nappa Á
Title	Country-Wise Forecast Model for the Effective Reproduction Number R<inf>t</inf> of Coronavirus Disease
Year	2020
Journal	Frontiers in Physics
DOI	10.3389/fphy.2020.00304

Extraction not feasible due to quality issues

Extraction not feasible due to quality issues

Author	Adly HM, AlJahdali IA, Garout MA, Khafagy AA, Saati AA, Saleh SAK
Title	Correlation of COVID-19 Pandemic with Healthcare System Response and Prevention Measures in Saudi Arabia
Year	2020
Journal	International Journal of Environmental Research and Public Health
DOI	10.3390/ijerph17186666
Author	Al Zobbi M, Alsinglawi B, Mubin O, Alnajjar F
Title	Measurement Method for Evaluating the Lockdown Policies during the COVID-19 Pandemic
Year	2020
Journal	International Journal of Environmental Research and Public Health
DOI	10.3390/ijerph17155574
Author	Rajendran S, Jayagopal P
Title	Accessing Covid19 epidemic outbreak in Tamilnadu and the impact of lockdown through epidemiological models and dynamic systems
Year	2020

– continued on next page –

Extraction not feasible due to quality issues – continued from previous page

Journal	Measurement
DOI	10.1016/j.measurement.2020.108432
Author	Rui R, Tian M
Title	Joint estimation of case fatality rate of COVID-19 and power of quarantine strategy performed in Wuhan, China
Year	2020
Journal	Biometrical Journal
DOI	10.1002/bimj.202000116

Not available in English language

Not available in English language	
Author	Cao S, Feng P, Shi P
Title	Study on the epidemic development of COVID-19 in Hubei province by a modified SEIR model
Year	2020
Journal	Journal of Zhejiang University
DOI	not available

G PRISMA checklist

Section and Topic	Item #	Checklist Item	Location where item is reported
**TITLE**			
Title	1	Identify the report as a systematic review.	p. 1
**ABSTRACT**			
Abstract	2	See the PRISMA 2020 for Abstracts checklist	p. 1
**INTRODUCTION**			
Rationale	3	Describe the rationale for the review in the context of existing knowledge.	pp. 1–2
Objectives	4	Provide an explicit statement of the objective(s) or question(s) the review addresses.	p. 2
**METHODS**			
Eligibility criteria	5	Specify the inclusion and exclusion criteria for the review and how studies were grouped for the syntheses.	pp. 2–3
Information sources	6	Specify all databases, registers, websites, organisations, reference lists and other sources searched or consulted to identify studies. Specify the date when each source was last searched or consulted.	p. 3
Search strategy	7	Present the full search strategies for all databases, registers and websites, including any filters and limits used.	p. 3, SI Appendix B pp. 3–7
Selection process	8	Specify the methods used to decide whether a study met the inclusion criteria of the review, including how many reviewers screened each record and each report retrieved, whether they worked independently, and if applicable, details of automation tools used in the process.	p. 3
Data collection process	9	Specify the methods used to collect data from reports, including how many reviewers collected data from each report, whether they worked independently, any processes for obtaining or confirming data from study investigators, and if applicable, details of automation tools used in the process.	p. 4

– continued on next page –

Data items	10a	List and define all outcomes for which data were sought. Specify whether all results that were compatible with each outcome domain in each study were sought (e.g. for all measures, time points, analyses), and if not, the methods used to decide which results to collect.	SI Appendix D pp. 12–34
	10b	List and define all other variables for which data were sought (e.g. participant and intervention characteristics, funding sources). Describe any assumptions made about any missing or unclear information.	SI Appendix D pp. 12–34
Study risk of bias assessment	11	Specify the methods used to assess risk of bias in the included studies, including details of the tool(s) used, how many reviewers assessed each study and whether they worked independently, and if applicable, details of automation tools used in the process.	p. 4
Effect measures	12	Specify for each outcome the effect measure(s) (e.g. risk ratio, mean difference) used in the synthesis or presentation of results.	– not applicable –
Synthesis methods	13a	Describe the processes used to decide which studies were eligible for each synthesis (e.g. tabulating the study intervention characteristics and comparing against the planned groups for each synthesis (item 5)).	p. 4
	13b	Describe any methods required to prepare the data for presentation or synthesis, such as handling of missing summary statistics, or data conversions.	– not applicable –
	13c	Describe any methods used to tabulate or visually display results of individual studies and syntheses.	p. 4
	13d	Describe any methods used to synthesize results and provide a rationale for the choice(s). If meta-analysis was performed, describe the model(s), method(s) to identify the presence and extent of statistical heterogeneity, and software package(s) used.	p. 4
	13e	Describe any methods used to explore possible causes of heterogeneity among study results (e.g. subgroup analysis, meta-regression).	– not applicable –
	13f	Describe any sensitivity analyses conducted to assess robustness of the synthesized results.	– not applicable –

– continued on next page –

Reporting bias assessment	14	Describe any methods used to assess risk of bias due to missing results in a synthesis (arising from reporting biases).	– not applicable –
Certainty assessment	15	Describe any methods used to assess certainty (or confidence) in the body of evidence for an outcome.	– not applicable –
**RESULTS**			
Study selection	16a	Describe the results of the search and selection process, from the number of records identified in the search to the number of studies included in the review, ideally using a flow diagram.	pp. 4–5
	16b	Cite studies that might appear to meet the inclusion criteria, but which were excluded, and explain why they were excluded.	pp. 4–5, SI Appendix F pp. 650–691
Study characteristics	17	Cite each included study and present its characteristics.	SI Appendix E pp. 35–649
Risk of bias in studies	18	Present assessments of risk of bias for each included study.	– not applicable –
Results of individual studies	19	For all outcomes, present, for each study: (a) summary statistics for each group (where appropriate) and (b) an effect estimate and its precision (e.g. confidence/credible interval), ideally using structured tables or plots.	– not applicable –
Results of syntheses	20a	For each synthesis, briefly summarise the characteristics and risk of bias among contributing studies.	– not applicable –
	20b	Present results of all statistical syntheses conducted. If meta-analysis was done, present for each the summary estimate and its precision (e.g. confidence/credible interval) and measures of statistical heterogeneity. If comparing groups, describe the direction of the effect.	– not applicable –
	20c	Present results of all investigations of possible causes of heterogeneity among study results.	– not applicable –
	20d	Present results of all sensitivity analyses conducted to assess the robustness of the synthesized results.	– not applicable –
Reporting biases	21	Present assessments of risk of bias due to missing results (arising from reporting biases) for each synthesis assessed.	– not applicable –

– continued on next page –

Certainty of evidence	22	Present assessments of certainty (or confidence) in the body of evidence for each outcome assessed.	– not applicable –
**DISCUSSION**			
Discussion	23a	Provide a general interpretation of the results in the context of other evidence.	pp. 16–18
	23b	Discuss any limitations of the evidence included in the review.	pp. 17–18
	23c	Discuss any limitations of the review processes used.	p. 18
	23d	Discuss implications of the results for practice, policy, and future research.	p. 19
**OTHER INFORMATION**			
Registration and protocol	24a	Provide registration information for the review, including register name and registration number, or state that the review was not registered.	p. 2
	24b	Indicate where the review protocol can be accessed, or state that a protocol was not prepared.	p. 2
	24c	Describe and explain any amendments to information provided at registration or in the protocol.	p. 3
Support	25	Describe sources of financial or non-financial support for the review, and the role of the funders or sponsors in the review.	p. 19
Competing interests	26	Declare any competing interests of review authors.	p. 19
Availability of data, code and other materials	27	Report which of the following are publicly available and where they can be found: template data collection forms; data extracted from included studies; data used for all analyses; analytic code; any other materials used in the review.	p. 19

References

1. Singh BP, Singh G. Modeling tempo of COVID-19 pandemic in India and significance of lockdown. Journal of Public Affairs. 2020;.

2. Cheng Q, Liu Z, Cheng G, Huang J. Heterogeneity and Effectiveness Analysis of COVID-19 Prevention and Control in Major Cities in China through Time-Varying Reproduction Number Estimation. Scientific Reports. 2020;10.

3. Lei H, Wu X, Wang X, Xu M, Xie Y, Du X, et al. Different Transmission Dynamics of Coronavirus Disease 2019 (COVID-19) and Influenza Suggest the Relative Efficiency of Isolation/Quarantine and Social Distancing against COVID-19 in China. Clinical Infectious Diseases. 2020;73(11):e4305–e4311.

4. Loeffler-Wirth H, Schmidt M, Binder H. Covid-19 Transmission Trajectories–Monitoring the Pandemic in the Worldwide Context. Viruses. 2020;12(7):777.

5. Tiwari V, Deyal N, Bisht NS. Mathematical Modeling Based Study and Prediction of COVID-19 Epidemic Dissemination under the Impact of Lockdown in India. Frontiers in Physics. 2020;8.

6. Mukandavire Z, Nyabadza F, Malunguza NJ, Cuadros DF, Shiri T, Musuka G. Quantifying Early COVID-19 Outbreak Transmission in South Africa and Exploring Vaccine Efficacy Scenarios. PLOS ONE. 2020;15(7):e0236003.

7. Gostic KM, McGough L, Baskerville EB, Abbott S, Joshi K, Tedijanto C, et al. Practical Considerations for Measuring the Effective Reproductive Number, Rt. PLOS Computational Biology. 2020;16(12):e1008409.

8. Cori A, Ferguson NM, Fraser C, Cauchemez S. A New Framework and Software to Estimate Time-Varying Reproduction Numbers During Epidemics. American Journal of Epidemiology. 2013;178(9):1505–1512.

9. an der Heiden M, Hamouda O. Schätzung Der Aktuellen Entwicklung Der SARS-CoV-2- Epidemie in Deutschland – Nowcasting. Epidemiologisches Bulletin. 2020;2020(17):10–15.

10. Thompson RN, Stockwin JE, van Gaalen RD, Polonsky JA, Kamvar ZN, Demarsh PA, et al. Improved Inference of Time-Varying Reproduction Numbers during Infectious Disease Outbreaks. Epidemics. 2019;29:100356.

11. Forsberg White L, Pagano M. A Likelihood-Based Method for Real-Time Estimation of the Serial Interval and Reproductive Number of an Epidemic. Statistics in Medicine. 2008;27(16):2999–3016.

12. Wallinga J, Lipsitch M. How Generation Intervals Shape the Relationship between Growth Rates and Reproductive Numbers. Proceedings of the Royal Society B: Biological Sciences. 2007;274(1609):599–604.

13. Wallinga J, Teunis P. Different Epidemic Curves for Severe Acute Respiratory Syndrome Reveal Similar Impacts of Control Measures. American Journal of Epidemiology. 2004;160(6):509–516.

14. Edjoc R, Atchessi N, Lien A, Smith BA, Gabrani-Juma I, Abalos C, et al. Assessing the Progression of the COVID-19 Pandemic in Canada Using Testing Data and Time-Dependent Reproduction Numbers. Canadian Journal of Public Health. 2020;111(6):926–938.

15. Mitra A, Pakhare AP, Roy A, Joshi A. Impact of COVID-19 Epidemic Curtailment Strategies in Selected Indian States: An Analysis by Reproduction Number and Doubling Time with Incidence Modelling. PLOS ONE. 2020;15(9):e0239026.

16. You C, Deng Y, Hu W, Sun J, Lin Q, Zhou F, et al. Estimation of the Time-Varying Reproduction Number of COVID-19 Outbreak in China. International Journal of Hygiene and Environmental Health. 2020;228:113555.

17. Brauner JM, Mindermann S, Sharma M, Johnston D, Salvatier J, Gavenčiak T, et al. Inferring the Effectiveness of Government Interventions against COVID-19. Science. 2021;371(6531):eabd9338.

18. Shen Y, Powell G, Ganser I, Zheng Q, Grundy C, Okhmatovskaia A, et al. Monitoring Non-Pharmaceutical Public Health Interventions during the COVID-19 Pandemic. Scientific Data. 2021;8:225.

19. Our World in Data. Coronavirus Pandemic (COVID-19); 2022. https://ourworldindata.org/coronavirus.

20. Lloyd-Smith JO, Schreiber SJ, Kopp PE, Getz WM. Superspreading and the Effect of Individual Variation on Disease Emergence. Nature. 2005;438(7066):355–359.

21. Diekmann O, Heesterbeek JAP, Metz JAJ. On the Definition and the Computation of the Basic Reproduction Ratio R0 in Models for Infectious Diseases in Heterogeneous Populations. Journal of Mathematical Biology. 1990;28(4):365–382.

22. Johns Hopkins University & Medicine. Coronavirus Resource Center; 2022. https://coronavirus.jhu.edu/.

23. European Centre for Disease Prevention and Control. COVID-19 Datasets; 2022. https://www.ecdc.europa.eu/en/covid-19/data.

24. World Health Organization. WHO Coronavirus (COVID-19) Dashboard; 2022. https://covid19.who.int/.

25. Worldometer. Coronavirus Statistics; 2022. https://www.worldometers.info/coronavirus/.

26. Google. COVID-19 Community Mobility Reports; 2022. https://www.google.com/covid19/mobility/.

27. Apple. COVID-19 Mobility Trends Reports; 2022. https://covid19.apple.com/mobility.

28. Safegraph. Places Data and Foot Traffic Insights; 2022. https://www.safegraph.com/.

29. Hale T, Angrist N, Goldszmidt R, Kira B, Petherick A, Phillips T, et al. A Global Panel Database of Pandemic Policies (Oxford COVID-19 Government Response Tracker). Nature Human Behaviour. 2021;5(4):529–538.

30. Thapa DK, Visentin DC, Hunt GE, Watson R, Cleary M. Being honest with causal language in writing for publication. Journal of Advanced Nursing. 2020;76(6):1285–1288.
